# Supplementary material for: Cardiovascular outcomes of semaglutide and tirzepatide for patients with type 2 diabetes in clinical practice
Source: Nat Med. 2025 Nov 9;32(1):342–52. doi: 10.1038/s41591-025-04102-x (PMC12823426; doi:10.1038/s41591-025-04102-x)
Supplement: Supplementary file 1 — Supplementary Figs. 1–3 and Tables 1−31. [file 41591_2025_4102_MOESM1_ESM.pdf]

nature medicine

Supplementary information https://doi.org/10.1038/s41591-025-04102-x

Cardiovascular outcomes of semaglutide and tirzepatide for patients with type 2 diabetes in clinical practice

In the format provided by the authors and unedited

Supplementary Information for “Cardiovascular outcomes of semaglutide and tirzepatide for patients with type 2 diabetes in clinical practice”

Krüger et al

Supplementary Figure 1. Study design diagrams for the emulation of SUSTAIN-6 and the comparison of semaglutide versus sitagliptin in expanded populations

Supplementary Figure 2. Study design diagrams for the emulation of SURPASS-CVOT and the comparison of tirzepatide versus dulaglutide in expanded populations

Supplementary Figure 3. Study design diagram for the comparison of tirzepatide versus semaglutide in expanded populations

Supplementary Table 1. Baseline characteristics of initiators of semaglutide vs sitagliptin, tirzepatide vs dulaglutide, and tirzepatide vs semaglutide when applying expanded eligibility criteria before and after propensity score matching, pooled across databases. Values are number (percentage) unless otherwise specified.

Supplementary Table 2. Baseline characteristics of initiators of semaglutide vs sitagliptin when applying trial eligibility criteria before and after propensity score matching, pooled across databases. Values are number (percentage) unless otherwise specified.

Supplementary Table 3. Baseline characteristics of initiators of semaglutide vs sitagliptin when applying trial eligibility criteria before and after propensity score matching, Optum database. Values are number (percentage) unless otherwise specified.

Supplementary Table 4. Baseline characteristics of initiators of semaglutide vs sitagliptin when applying trial eligibility criteria before and after propensity score matching, Marketscan databases. Values are number (percentage) unless otherwise specified.

Supplementary Table 5. Baseline characteristics of initiators of semaglutide vs sitagliptin when applying trial eligibility criteria before and after propensity score matching, Medicare database. Values are number (percentage) unless otherwise specified.

Supplementary Table 6. Baseline characteristics of initiators of semaglutide vs sitagliptin when applying expanded eligibility criteria before and after propensity score matching, pooled across databases. Values are number (percentage) unless otherwise specified.

Supplementary Table 7. Baseline characteristics of initiators of semaglutide vs sitagliptin when applying trial eligibility criteria before and after propensity score matching, Optum database. Values are number (percentage) unless otherwise specified.

Supplementary Table 8. Baseline characteristics of initiators of semaglutide vs sitagliptin when applying trial eligibility criteria before and after propensity score matching, Marketscan database. Values are number (percentage) unless otherwise specified.

Supplementary Table 9. Baseline characteristics of initiators of semaglutide vs sitagliptin when applying trial eligibility criteria before and after propensity score matching, Medicare database. Values are number (percentage) unless otherwise specified.

Supplementary Table 10. Baseline characteristics of initiators of tirzepatide vs dulaglutide when applying trial eligibility criteria before and after propensity score matching, pooled across database. Values are number (percentage) unless otherwise specified.

Supplementary Table 11. Baseline characteristics of initiators of tirzepatide vs dulaglutide when applying trial eligibility criteria before and after propensity score matching, Optum database. Values are number (percentage) unless otherwise specified.

Supplementary Table 12. Baseline characteristics of initiators of tirzepatide vs dulaglutide when applying trial eligibility criteria before and after propensity score matching. Marketscan database. Values are number (percentage) unless otherwise specified.

Supplementary Table 13. Baseline characteristics of initiators of tirzepatide vs dulaglutide when applying expanded eligibility criteria before and after propensity score matching, pooled databases. Values are number (percentage) unless otherwise specified.

Supplementary Table 14. Baseline characteristics of initiators of tirzepatide vs dulaglutide when applying expanded eligibility criteria before and after propensity score matching. Optum database. Values are number (percentage) unless otherwise specified.

Supplementary Table 15. Baseline characteristics of initiators of tirzepatide vs dulaglutide when applying expanded eligibility criteria before and after propensity score matching. Marketscan database. Values are number (percentage) unless otherwise specified.

Supplementary Table 16. Baseline characteristics of initiators of tirzepatide vs semaglutide when applying expanded eligibility criteria before and after propensity score matching, across pooled databases. Values are number (percentage) unless otherwise specified.

Supplementary Table 17. Baseline characteristics of initiators of tirzepatide vs semaglutide when applying expanded eligibility criteria before and after propensity score matching. Optum database. Values are number (percentage) unless otherwise specified.

Supplementary Table 18. Baseline characteristics of initiators of tirzepatide vs semaglutide when applying expanded eligibility criteria before and after propensity score matching. MarketScan database. Values are number (percentage) unless otherwise specified.

Supplementary Table 19. Primary end point of all-cause mortality, myocardial infarction or stroke among individuals initiating (A) semaglutide vs sitagliptin, (B) tirzepatide vs dulaglutide, or (C) tirzepatide vs semaglutide, reported by eligibility criteria applied and end point.

Supplementary Table 20. Censoring reason for the primary end point among individuals initiating (A) semaglutide vs sitagliptin, (B) tirzepatide vs dulaglutide, or (C) tirzepatide vs semaglutide, reported by eligibility criteria applied and database.

Supplementary Table 21. Secondary end points among individuals initiating (A) semaglutide vs sitagliptin, (B) tirzepatide vs dulaglutide, or (C) tirzepatide vs semaglutide, reported by eligibility criteria applied and database.

Supplementary Table 22. Safety end points among individuals initiating (A) semaglutide vs sitagliptin, (B) tirzepatide vs dulaglutide, or (C) tirzepatide vs semaglutide, reported by eligibility criteria applied and database.

Supplementary Table 23. Sensitivity analyses among individuals initiating (A) semaglutide vs sitagliptin, (B) tirzepatide vs dulaglutide, or (C) tirzepatide vs semaglutide, reported by eligibility criteria applied and database.

Supplementary Table 24. Subgroups for the primary end point among individuals initiating (A) semaglutide vs sitagliptin, (B) tirzepatide vs dulaglutide, or (C) tirzepatide vs semaglutide, reported by eligibility criteria applied and database.

Supplementary Table 25. 2-year on-treatment causal contrast *post-hoc* analysis

Supplementary Table 26. Overview of cardiovascular risk levels across conducted studies.

Supplementary Table 27. Specification and emulation of the SUSTAIN-6 trial including our expansion study.

Supplementary Table 28. Specification and emulation of the SURPASS-CVOT trial including our expansion study.

Supplementary Table 29. Specification and emulation of the target trial comparing tirzepatide versus semaglutide.

Supplementary Table 30. Primary end points before and after propensity score matching

Supplementary Table 31. Power calculations for superiority and non-inferiority studies

Supplementary Figure 1. Study design diagrams for the emulation of SUSTAIN-6 and the comparison of semaglutide versus sitagliptin in expanded populations

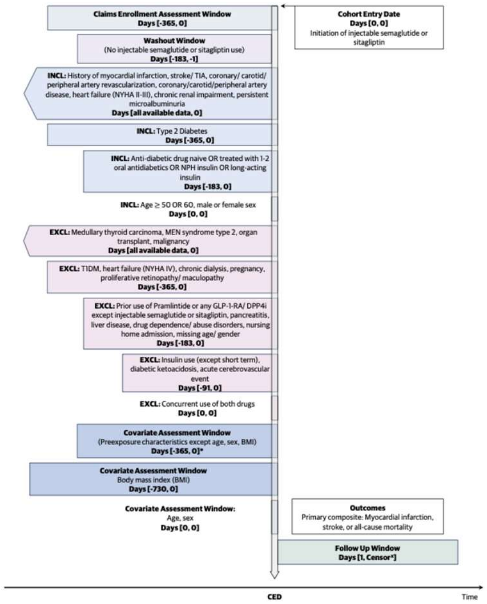

*Censoring criteria: Occurrence of outcomes, end of study period, death, disenrollment, 365 days after CED, discontinuation (45 days grace window and risk window), switch to other GLP-1-RA/ DPPP4i, nursing home admission
*Covariate assessment window periods vary within [-365, 0] for certain items. Details are documented in the study protocol.

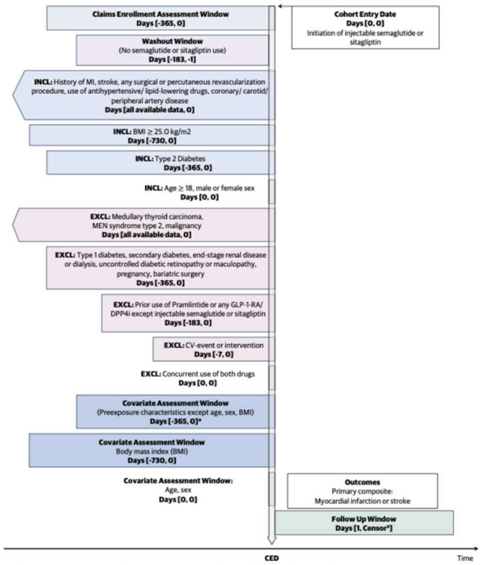

*Censoring criteria: Occurrence of outcomes, end of study period, death, disenrollment, 365 days after CED, discontinuation (45 days grace window and risk window), switch to other GLP-1-RA/ DPP4i, nursing home admission
*Covariate assessment window periods vary within [-365, 0] for certain items. Details are documented in the study protocol.

Supplementary Figure 2. Study design diagrams for the emulation of SURPASS-CVOT and the comparison of tirzepatide versus dulaglutide in expanded populations

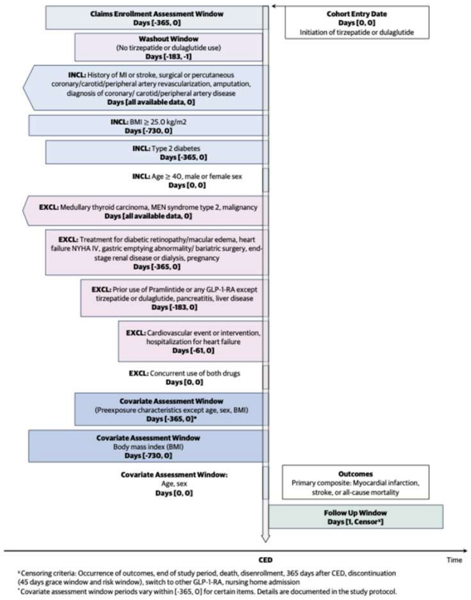

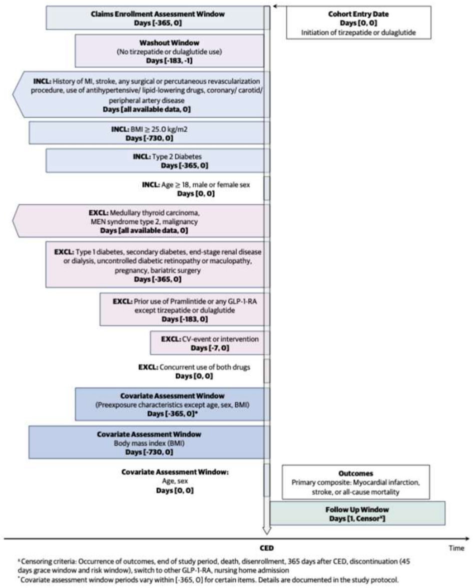

Supplementary Figure 3. Study design diagram for the comparison of tirzepatide versus semaglutide in expanded populations

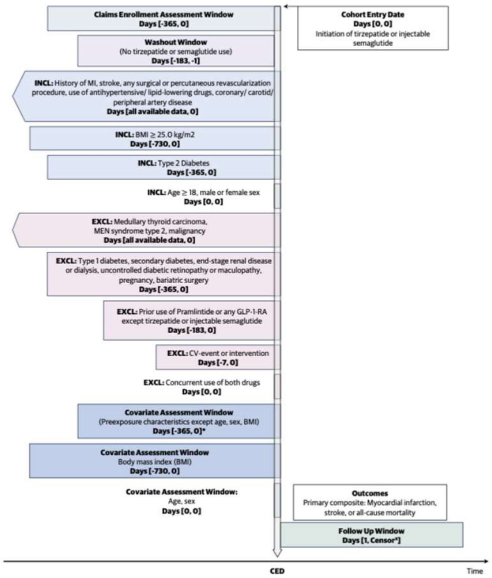

*Censoring criteria: Occurrence of outcomes, end of study period, death, disenrollment, 365 days after CED, discontinuation (45 days grace window and risk window), switch to other GLP-1-RA, nursing home admission

*Covariate assessment window periods vary within [-365, 0] for certain items. Details are documented in the study protocol.

Supplementary Table 26. Overview of cardiovascular risk levels across conducted studies.

Population	Cardiovascular Risk Levels	Population Characteristics
SURPASS-CVOT	High risk	Required established atherosclerotic cardiovascular disease (coronary, cerebrovascular, or peripheral arterial disease)
SUSTAIN-6	Moderate and high risk	Included patients with established CVD or CKD (high risk), and subclinical CV risk (moderate) such as microalbuminuria or LV dysfunction
Expanded populations routinely seen in clinical practice	Low, moderate, and high risk	Included individuals with CV risk factors (e.g., treated hypertension, dyslipidemia) without organ damage, as well as moderate and high-risk individuals

Supplementary Table 27. Specification and emulation of the SUSTAIN-6 trial including our expansion study.

Component	Specification of reference trial	Emulation of reference trial using US claims data	Specification of hypothetical target trial in expanded population	Emulation of hypothetical target trial in expanded population using US claims data
**Eligibility criteria**	SUSTAIN-6 (NCT01720446) enrolled individuals with type 2 diabetes at moderate to high cardiovascular risk.<br>Inclusion: Type 2 diabetes; HbA1c ≥7.0%; anti-diabetic drug naïve or on ≤2 oral agents, basal or premixed insulin; age ≥50 y with established cardiovascular disease or ≥60 y with subclinical cardiovascular disease.<br>Exclusion: Type 1/secondary/gestational diabetes; recent GLP-1 receptor agonist, pramlintide, or DPP4 inhibitor use; recent non-basal/premixed insulin use; recent acute decompensated diabetes, pancreatitis, acute coronary/cerebrovascular event, or planned revascularization; NYHA IV heart failure; medullary thyroid cancer/multiple endocrine neoplasia type 2 or high calcitonin; chronic dialysis or end-stage liver disease; prior/planned transplant; cancer history (except certain skin cancers); acute/uncontrolled conditions; pregnancy; hypersensitivity to study drug; substance abuse; proliferative retinopathy/maculopathy requiring acute treatment.	The emulation of SUSTAIN-6 (NCT06659744) applied the same eligibility criteria, to the extent possible, to individuals in the three claims databases. In addition, at least 12 months of continuous medical and pharmacy enrollment was required prior to cohort entry, and no use of GLP-1 receptor agonists or comparator drugs in the preceding 6 months. Observational analogues of trial eligibility criteria were defined using claims-based definitions measured in a baseline window prior to treatment initiation. Detailed operationalization of the eligibility criteria with coding algorithms and assessment windows are provided in the Supplementary Information.	The expanded population trial would broaden inclusion to reflect patients with type 2 diabetes and overweight at low, moderate, and high cardiovascular risk who are commonly treated in clinical practice.<br>Inclusion: Men and women aged ≥18 years; type 2 diabetes mellitus; BMI ≥25 kg/m²; low, moderate, or high cardiovascular risk, defined as at least one of the following: history of a cardiovascular event; documented history of myocardial infarction or unstable angina; history of ischemic stroke or transient ischemic attack; history of heart failure; prior surgical or percutaneous coronary, carotid, or peripheral revascularization procedure; current use of ≥1 antihypertensive or ≥1 lipid-lowering drug; or documented history of atherosclerotic cardiovascular disease, including coronary, carotid, or peripheral artery disease.<br>Exclusion: History of type 1 or secondary diabetes; personal or family history of multiple endocrine neoplasia type 2 or medullary thyroid carcinoma; end-stage renal disease or dialysis; prior treatment for diabetic retinopathy or macular edema; history of any malignancy; cardiovascular event or intervention within the past 7 days; GLP-1 receptor agonist treatment within the past 6 months; bariatric surgery within the past 12 months; or pregnancy or breastfeeding.	The emulation of the expanded population trial applied the same eligibility using claims-based definitions. Detailed operationalizations with coding algorithms and assessment windows are provided in the Supplementary Information.
**Treatment strategies**	Treatment strategies in SUSTAIN-6 were initiation of injectable semaglutide or placebo.	Treatment strategies in the emulation of SUSTAIN-6 compared new initiation of injectable semaglutide or sitagliptin. Sitagliptin was used to approximate placebo, given its neutral effect on cardiovascular outcomes and prior use as a placebo proxy in observational studies. Treatment exposure in the database studies was identified through outpatient pharmacy dispensing claims using National	Treatment strategies in the expanded population trial would be initiation of injectable semaglutide or sitagliptin in clinical practice.	Treatment strategies in the emulation of the expanded population trial were the same.

		Drug Codes.		
**Assignment procedures**	Assignment procedures in SUSTAIN-6 involved random allocation to treatment groups at baseline, with blinding of both participants and providers. However, because rapid weight loss is an anticipated effect, participants and clinicians may be unintentionally unblinded during the trial.	Assignment procedures in the emulation of SUSTAIN-6 were performed based on first observed dispensing of semaglutide or sitagliptin from outpatient prescription claims that met eligibility criteria. To emulate a new-user design, a 183-day washout period with no dispensing of GLP-1 receptor agonists or comparator drugs prior to treatment initiation was required. Each individual could contribute only once per comparison and was assigned to the strategy consistent with the treatment they received in routine clinical care. Assignment was conducted without knowledge of future outcomes. To mimic randomization, eligible patients who initiated each study drug were matched in a 1:1 ratio to initiators of the comparator drug based on the propensity score, that was estimated with logistic regression, using a comprehensive set of baseline covariates (see Supplementary Information).	Assignment procedures in the expanded population trial would involve random allocation. Patients would be aware of the treatment strategies they receive.	Assignment procedures in the emulation of the expanded population trial were performed based on first qualifying new treatment dispensing of injectable semaglutide or sitagliptin from outpatient prescription claims. Randomization was mimicked using 1:1 propensity score matching on baseline characteristics (see Supplementary Information)
**Follow-up**	Follow-up in SUSTAIN-6 began on the day of treatment initiation. While there were protocolized procedures in place to maximize adherence to randomized therapy assignment, the trial allowed discontinuation for safety reasons. Patients who discontinued the therapy were allowed alternative therapy, but drugs affecting the incretin pathway (including other GLP-1-RA, pramlintide, or DPP4i) were strongly discouraged. Glucose-lowering therapy for each participant was allowed to be modified at the discretion of the site investigator through open-label initiation or adjustment of non–GLP-1 receptor agonist medications, with individualized HbA1c targets set according to local clinical practice and current professional society guidelines.<br>Follow-up in SUSTAIN-6 ended at the date of the last planned follow-up visit for the last participant, once the prespecified number of primary outcome events had occurred, as defined in the trial protocol.	Follow-up in the emulation of SUSTAIN-6 began on the day after first prescription fill.<br>Follow-up in the emulation of SUSTAIN-6 ended at the earliest occurrence of either the outcome, death, disenrollment, nursing home admission, administrative end of follow-up (including 365 day of maximum follow-up or end of data), treatment discontinuation (including 45-day grace period and risk window), or switching of study drugs between exposure and comparator.	Follow-up in the expanded population trial would begin on the day after first prescription fill.<br>Follow-up in the expanded population trial would end at first of outcome occurrence, death, disenrollment, nursing home admission, administrative end of follow-up (including 365 day of maximum follow-up or end of data), treatment discontinuation (including 45-day grace period and risk window), or switching of study drugs between exposure and comparator.	Follow-up start and end in the emulation of the expanded population trial was the same as in the emulation of SUSTAIN-6.

**Outcomes**	In SUSTAIN-6, the primary outcome was major cardiovascular adverse events, a composite defined as cardiovascular mortality, myocardial infarction, or stroke. Secondary outcomes included the individual components of this composite.	In the emulation of SUSTAIN-6, the primary and secondary outcomes were the same, except for capturing all-cause mortality instead of cardiovascular mortality.<br>In addition, we assessed two negative control outcomes, defined as abdominal hernia and lumbar radiculopathy. These outcomes are not expected to differ between treatment strategies, as there is no known biological effect of the study drugs on these outcomes; they serve to detect potential residual confounding when emulating SUSTAIN-6.	In the expanded population trial, the primary outcome would be major cardiovascular adverse events, a composite defined as myocardial infarction, stroke, or all-cause mortality. Additionally, a version of the primary outcome excluding mortality would be assessed.<br>Secondary outcomes would include the individual components of major cardiovascular adverse events as well as a composite of hospitalization for heart failure, urgent heart failure visits, or all-cause mortality. Additionally, a version of the secondary outcome excluding mortality would be assessed.<br>Safety outcomes would include gastrointestinal adverse events, serious bacterial infections, and urinary tract infections.	In the emulation of expanded population trial, all outcomes were the same.<br>In addition, two negative control outcomes abdominal hernia and lumbar radiculopathy were assessed.
**Causal contrast**	In SUSTAIN-6, the causal contrast of interest was the intention-to-treat effect.	In the emulation of SUSTAIN-6, the primary causal contrast was the observational analog of the per-protocol effect, referred to as the on-treatment effect. Given the lower adherence typically observed in clinical practice compared to randomized trials, an analog of the intention-to-treat effect in a database study is likely subject to substantially more exposure misclassification due to discontinuation (diluting the effects toward the null). In such settings, an on-treatment effect may more closely emulate the effect observed among highly adherent trial participants in a trial's intention-to-treat analysis. Therefore, the on-treatment analysis was designated the primary analysis. The observational analog of the intention-to-treat effect, referred to as the as-started effect, was assessed in a sensitivity analysis.	In the expanded population trial, the primary causal contrast would be the per-protocol effect. In addition, the intention-to-treat effect would be evaluated.	In the emulation of the expanded population trial, the causal contrast was the on-treatment effect (per-protocol analog). In addition, the as-started effect (intention-to-treat analog) was also evaluated.
**Identifying assumptions**	Identifying assumptions in SUSTAIN-6 for the intention-to-treat effect assumed random treatment assignment and loss to follow-up, conditional on observed data. The per-protocol effect additionally assumed no unmeasured confounding of adherence and outcome, correct model specification, and nondifferential censoring.	Identifying assumptions in the emulation of SUSTAIN-6 assumed conditional exchangeability at baseline after adjustment for a comprehensive set of pre-treatment characteristics (detailed in the Supplementary Information). For the on-treatment analysis, additional assumption of non-informative censoring at treatment discontinuation or switching, and no unmeasured time-varying	Identifying assumptions in the expanded population trial would be the same as in SUSTAIN-6.	Identifying assumptions in the emulation of the expanded population trial were the same as in the emulation of SUSTAIN-6.

		confounding were made.		
**Data analysis plan**	In SUSTAIN-6, estimates of 1-year risks, risk differences, and hazard ratios comparing the treatment groups were derived from Kaplan–Meier estimates, Aalen-Johansen estimates, and Cox-proportional hazards models.	In the emulation of SUSTAIN-6, estimates of 1-year risks, risk differences, and hazard ratios comparing the treatment groups were derived via Kaplan–Meier estimates, Aalen-Johansen estimates, and Cox-proportional hazards models.	The data analysis plan in the expanded population trial would be the same as in SUSTAIN-6.	The data analysis plan in the emulation of the expanded population trial was the same as in the emulation of SUSTAIN-6.

Supplementary Table 28. Specification and emulation of the SURPASS-CVOT trial including our expansion study.

Component	Specification of reference trial	Emulation of reference trial using US claims data	Specification of hypothetical target trial in expanded population	Emulation of hypothetical target trial in expanded population using US claims data
**Eligibility criteria**	SURPASS-CVOT (NCT04255433) enrolled individuals with type 2 diabetes at high cardiovascular risk.<br>Inclusion: Men and women aged 40 years or older; type 2 diabetes mellitus; HbA1c ≥7% and ≤ 10.5%; BMI ≥ 25kg/m²; established atherosclerotic cardiovascular disease (either of documented history of myocardial infarction, ≥50% stenosis in ≥1 major coronary arteries determined by invasive angiography, ≥50% stenosis in 2 or more major coronary arteries, history of surgical or percutaneous coronary revascularization procedure, documented history of ischemic stroke, carotid arterial disease with ≥50% stenosis, documented by carotid ultrasound, MRI, or angiography, history of carotid stenting or surgical revascularization, intermittent claudication and ankle-brachial index <0.9, or prior nontraumatic amputation or peripheral vascular procedure (e.g., stenting or surgical revascularization), due to peripheral arterial ischemia).<br>Exclusion: HbA1c >10.5%, planned treatment for diabetic retinopathy and/or macular edema, hospitalized for chronic heart failure within 2 months prior to screening, chronic New York Heart Association Functional Classification IV heart failure, planned coronary, carotid, or peripheral artery revascularization, history of chronic or acute pancreatitis, known clinically significant gastric emptying abnormality or bariatric surgery, liver disease (not including non-alcoholic fatty liver disease or ALT level ≥3x the ULN, eGFR <15 mL/Min/1.73 m² or on chronic dialysis, family or personal history of multiple endocrine neoplasia or medullary thyroid carcinoma, elevated serum calcitonin level, cardiovascular event or intervention <60 days prior to screening, GLP-1-RA or	The emulation of SURPASS-CVOT (NCT07088718) applied the same eligibility criteria, to the extent possible, to individuals in the two claims databases. In addition, at least 12 months of continuous medical and pharmacy enrollment was required prior to cohort entry, and no use of GLP-1 receptor agonists in the preceding 6 months. Observational analogues of trial eligibility criteria were defined using claims-based definitions measured in a baseline window prior to treatment initiation. Detailed operationalization of the eligibility criteria with coding algorithms and assessment windows are provided in the Appendix.	The expanded population trial would broaden inclusion to reflect patients with type 2 diabetes and overweight at low, moderate, and high cardiovascular risk who are commonly treated in clinical practice.<br>Inclusion: Men and women aged ≥18 years; type 2 diabetes mellitus; BMI ≥25 kg/m²; low, moderate, or high cardiovascular risk, defined as at least one of the following: history of a cardiovascular event; documented history of myocardial infarction or unstable angina; history of ischemic stroke or transient ischemic attack; history of heart failure; prior surgical or percutaneous coronary, carotid, or peripheral revascularization procedure; current use of ≥1 antihypertensive or ≥1 lipid-lowering drug; or documented history of atherosclerotic cardiovascular disease, including coronary, carotid, or peripheral artery disease.<br>Exclusion: History of type 1 or secondary diabetes; personal or family history of multiple endocrine neoplasia type 2 or medullary thyroid carcinoma; end-stage renal disease or dialysis; prior treatment for diabetic retinopathy or macular edema; history of any malignancy; cardiovascular event or intervention within the past 7 days; GLP-1 receptor agonist or sitagliptin treatment within the past 6 months; bariatric surgery within the past 12 months; or pregnancy or breastfeeding.	The emulation of the expanded population trial applied the same eligibility using claims-based definitions. Detailed operationalizations with coding algorithms and assessment windows are provided in the Appendix.

	pramlintide treatment within 3 months to randomization, women who are pregnant or breastfeeding.			
**Treatment strategies**	Treatment strategies in SURPASS-CVOT were initiation of tirzepatide or dulaglutide.	Treatment strategies in the emulation of SURPASS-CVOT compared new initiation of tirzpeatide or dulaglutide. Treatment exposure in the database studies was identified through outpatient pharmacy dispensing claims using National Drug Codes.	Treatment strategies in the expanded population trial would be initiation of tirzepatide or dulaglutide in clinical practice.	Treatment strategies in the emulation of the expanded population trial were the same.
**Assignment procedures**	Assignment procedures in SURPASS-CVOT involved random allocation to treatment groups at baseline, with blinding of both participants and providers. However, because rapid weight loss is an anticipated effect, participants and clinicians may be unintentionally unblinded during the trial.	Assignment procedures in the emulation of SURPASS-CVOT were performed based on first observed dispensing of tirzepatide or dulaglutide from outpatient prescription claims that met eligibility criteria. To emulate a new-user design, a 183-day washout period with no dispensing of GLP-1 receptor prior to treatment initiation was required. Each individual could contribute only once per comparison and was assigned to the strategy consistent with the treatment they received in routine clinical care. Assignment was conducted without knowledge of future outcomes. To mimic randomization, eligible patients who initiated each study drug were matched in a 1:1 ratio to initiators of the comparator drug based on the propensity score, that was estimated with logistic regression, using a comprehensive set of baseline covariates (see Appendix).	Assignment procedures in the expanded population trial would involve random allocation. Patients would be aware of the treatment strategies they receive.	Assignment procedures in the emulation of the expanded population trial were performed based on first qualifying new treatment dispensing of tirzepatide or dulaglutide from outpatient prescription claims. Randomization was mimicked using 1:1 propensity score matching on baseline characteristics (see Appendix)
**Follow-up**	Follow-up in SURPASS-CVOT began on the day of treatment initiation. While there were protocolized procedures in place to maximize adherence to randomized therapy assignment, the trial allowed discontinuation for safety reasons. Patients who discontinued the therapy were allowed alternative therapy, but drugs affecting the incretin pathway (including other GLP-1-RA, pramlintide, or DPP4i) were strongly discouraged. Glucose-lowering therapy for each participant was allowed to be modified at the discretion of the site investigator through open-label initiation or adjustment of non–GLP-1 receptor agonist medications, with individualized HbA1c targets set according to local clinical practice and current professional society guidelines.	Follow-up in the emulation of SURPASS-CVOT began on the day after first prescription fill. Follow-up in the emulation of SURPASS-CVOT ended at the earliest occurrence of either the outcome, death, disenrollment, nursing home admission, administrative end of follow-up (including 365 day of maximum follow-up or end of data), treatment discontinuation (including 45-day grace period and risk window), or switching of study drugs between exposure and comparator.	Follow-up in the expanded population trial would begin on the day after first prescription fill.<br>Follow-up in the expanded population trial would end at first of outcome occurrence, death, disenrollment, nursing home admission, administrative end of follow-up (including 365 day of maximum follow-up or end of data), treatment discontinuation (including 45-day grace period and risk window), or switching of study drugs between exposure and comparator.	Follow-up start and end in the emulation of the expanded population trial was the same as in the emulation of SURPASS-CVOT.

	Follow-up in SURPASS-CVOT ended once the prespecified number of primary outcome events had occurred, with the trial concluding at the final scheduled follow-up visit fort he last enrolled participant, as defined in the trial design and process paper.			
**Outcomes**	In SURPASS-CVOT, the primary outcome was major cardiovascular adverse events, a composite defined as cardiovascular mortality, myocardial infarction, or stroke. Secondary outcomes included the individual components of this composite.	In the emulation of SURPASS-CVOT, the primary and secondary outcomes were the same, except for capturing all-cause mortality instead of cardiovascular mortality.<br>In addition, we assessed two negative control outcomes, defined as abdominal hernia and lumbar radiculopathy. These outcomes are not expected to differ between treatment strategies, as there is no known biological effect of the study drugs on these outcomes; they serve to detect potential residual confounding when emulating SURPASS-CVOT.	In the expanded population trial, the primary outcome would be major cardiovascular adverse events, a composite defined as myocardial infarction, stroke, or all-cause mortality.<br>Secondary outcomes would include the individual components of major cardiovascular adverse events as well as a composite of hospitalization for heart failure, urgent heart failure visits, or all-cause mortality.<br>Safety outcomes would include gastrointestinal adverse events, serious bacterial infections, and urinary tract infections.	In the emulation of expanded population trial, all outcomes were the same.<br>In addition, two negative control outcomes abdominal hernia and lumbar radiculopathy were assessed.
**Causal contrast**	In SURPASS-CVOT, the causal contrast of interest was the intention-to-treat effect.	In the emulation of SURPASS-CVOT, the primary causal contrast was the observational analog of the per-protocol effect, referred to as the on-treatment effect. Given the lower adherence typically observed in clinical practice compared to randomized trials, an analog of the intention-to-treat effect in a database study is likely subject to substantially more exposure misclassification due to discontinuation (diluting the effects toward the null). In such settings, an on-treatment effect may more closely emulate the effect observed among highly adherent trial participants in a trial's intention-to-treat analysis. Therefore, the on-treatment analysis was designated the primary analysis. The observational analog of the intention-to-treat effect, referred to as the as-started effect, was assessed in a sensitivity analysis.	In the expanded population trial, the primary causal contrast would be the per-protocol effect. In addition, the intention-to-treat effect would be evaluated.	In the emulation of the expanded population trial, the causal contrast was the on-treatment effect (per-protocol analog). In addition, the as-started effect (intention-to-treat analog) was also evaluated.
**Identifying assumptions**	Identifying assumptions in SURPASS-CVOT for the intention-to-treat effect assumed random treatment assignment and loss to follow-up, conditional on observed data. The per-protocol effect additionally assumed no unmeasured confounding of	Identifying assumptions in the emulation of SUPRASS-CVOT assumed conditional exchangeability at baseline after adjustment for a comprehensive set of pre-treatment characteristics (detailed in the Appendix). For the on-treatment analysis, additional	Identifying assumptions in the expanded population trial would be the same as in SURPASS-CVOT.	Identifying assumptions in the emulation of the expanded population trial were the same as in the emulation of SURPASS-CVOT.

	adherence and outcome, correct model specification, and nondifferential censoring.	assumption of non-informative censoring at treatment discontinuation or switching, and no unmeasured time-varying confounding were made.		
**Data analysis plan**	In SURPASS-CVOT, estimates of 1-year risks, risk differences, and hazard ratios comparing the treatment groups were derived from Kaplan–Meier estimates, Aalen-Johansen estimates, and Cox-proportional hazards models.	In the emulation of SURPASS-CVOT, estimates of 1-year risks, risk differences, and hazard ratios comparing the treatment groups were derived via Kaplan–Meier estimates, Aalen-Johansen estimates, and Cox-proportional hazards models.	The data analysis plan in the expanded population trial would be the same as in SURPASS-CVOT.	The data analysis plan in the emulation of the expanded population trial was the same as in the emulation of SURPASS-CVOT.

Supplementary Table 29. Specification and emulation of the target trial comparing tirzepatide versus semaglutide.

Component	Specification of hypothetical target trial in expanded population	Emulation of hypothetical target trial in expanded population using US claims data
**Eligibility criteria**	The expanded population trial (NCT07096063) would enroll patients with type 2 diabetes and overweight or obesity across a broad spectrum of cardiovascular risk, including individuals at low, moderate, and high risk who are commonly treated in clinical practice.<br>Inclusion: Men and women aged ≥18 years; type 2 diabetes mellitus; BMI ≥25 kg/m²; low, moderate, or high cardiovascular risk, defined as at least one of the following: history of a cardiovascular event; documented history of myocardial infarction or unstable angina; history of ischemic stroke or transient ischemic attack; history of heart failure; prior surgical or percutaneous coronary, carotid, or peripheral revascularization procedure; current use of ≥1 antihypertensive or ≥1 lipid-lowering drug; or documented history of atherosclerotic cardiovascular disease, including coronary, carotid, or peripheral artery disease.<br>Exclusion: History of type 1 or secondary diabetes; personal or family history of multiple endocrine neoplasia type 2 or medullary thyroid carcinoma; end-stage renal disease or dialysis; prior treatment for diabetic retinopathy or macular edema; history of any malignancy; cardiovascular event or intervention within the past 7 days; GLP-1 receptor agonist treatment within the past 6 months; bariatric surgery within the past 12 months; or pregnancy or breastfeeding.	The emulation of the expanded population trial (NCT07096063) applied the same eligibility using claims-based definitions. In addition, at least 12 months of continuous medical and pharmacy enrollment was required prior to cohort entry, and no use of GLP-1 receptor agonists in the preceding 6 months. . Observational analogues of trial eligibility criteria were defined using claims-based definitions measured in a baseline window prior to treatment initiation. Detailed operationalizations with coding algorithms and assessment windows are provided in the Appendix.
**Treatment strategies**	Treatment strategies in the expanded population trial would be initiation of tirzepatide or semaglutide in clinical practice.	Treatment strategies in the emulation of the expanded population trial were the same.
**Assignment procedures**	Assignment procedures in the expanded population trial would involve random allocation. Patients would be aware of the treatment strategies they receive.	Assignment procedures in the emulation of the expanded population trial were performed based on first qualifying new treatment dispensing of tirzepatide or semaglutide from outpatient prescription claims. To emulate a new-user design, a 183-day washout period with no dispensing of GLP-1 receptor prior to treatment initiation was required. Each individual could contribute only once per comparison and was assigned to the strategy consistent with the treatment they received in routine clinical care. Assignment was conducted without knowledge of future outcomes. To mimic randomization, eligible patients who initiated each study drug were matched in a 1:1 ratio to initiators of the comparator drug based on the propensity score, that was estimated with logistic regression, using a comprehensive set of baseline covariates (see Appendix).
**Follow-up**	Follow-up in the expanded population trial would begin on the day after first prescription fill.<br>Follow-up in the expanded population trial would end at first of outcome occurrence, death, disenrollment, nursing home admission, administrative end of follow-up (including 365 day of maximum follow-up or end of data), treatment discontinuation (including 45-day grace period and risk window), or switching of study drugs between exposure and comparator.	Follow-up start and end in the emulation of the expanded population trial was the same.
**Outcomes**	In the expanded population trial, the primary outcome would be major cardiovascular adverse events, a composite defined as myocardial infarction, stroke, or all-cause mortality.<br>Secondary outcomes would include the individual components of major cardiovascular adverse events as well as a composite of hospitalization for heart failure, urgent heart failure visits, or all-cause mortality.	In the emulation of expanded population trial, all outcomes were the same.<br>In addition, two negative control outcomes abdominal hernia and lumbar radiculopathy were assessed.

	Safety outcomes would include gastrointestinal adverse events, serious bacterial infections, and urinary tract infections.	
**Causal contrast**	In the expanded population trial, the primary causal contrast would be the per-protocol effect. In addition, the intention-to-treat effect would be evaluated.	In the emulation of the expanded population trial, the causal contrast was the on-treatment effect (per-protocol analog). In addition, the as-started effect (intention-to-treat analog) was also evaluated.
**Identifying assumptions**	Identifying assumptions for the intention-to-treat effect in the expanded population trial would assume random treatment assignment and loss to follow-up, conditional on observed data.<br>The per-protocol effect additionally would assume no unmeasured confounding of adherence and outcome, correct model specification, and nondifferential censoring.	Identifying assumptions in the emulation of the expanded population trial assumed conditional exchangeability at baseline after adjustment for a comprehensive set of pre-treatment characteristics (detailed in the Appendix). For the on-treatment analysis, additional assumption of non-informative censoring at treatment discontinuation or switching, and no unmeasured time-varying confounding were made.
**Data analysis plan**	In the expanded population trial, estimates of 1-year risks, risk differences, and hazard ratios comparing the treatment groups would be derived from Kaplan–Meier estimates, Aalen-Johansen estimates, and Cox-proportional hazards models.	In the emulation of expanded population trial, estimates of 1-year risks, risk differences, and hazard ratios comparing the treatment groups were derived via Kaplan–Meier estimates, Aalen-Johansen estimates, and Cox-proportional hazards models.

Supplementary Table 1. Baseline characteristics of initiators of semaglutide vs sitagliptin, tirzepatide vs dulaglutide, and tirzepatide vs semaglutide when applying expanded eligibility criteria before and after propensity score matching, pooled across databases. Values are number (percentage) unless otherwise specified.

Variable	Semaglutide (n = 79,501)	Sitagliptin (n = 79,501)	SMD	Tirzepatide (n = 39,152)	Dulaglutide (n = 39,152)	SMD	Tirzepatide (n = 86,191)	Semaglutide (n = 86,191)	SMD
**Demographics**									
Age; mean (SD)	63.28 ±11.18	63.32 ±11.89	0.003	60.38 ±11.45	60.38 ±11.72	0.000	59.24 ±11.59	59.26 ±11.72	0.002
Gender									
Male	36,976 (46.5%)	36,844 (46.3%)	0.003	17,995 (46.0%)	17,971 (45.9%)	0.001	38,166 (44.3%)	38,117 (44.2%)	0.001
Female	42,525 (53.5%)	42,657 (53.7%)	0.003	21,157 (54,0%)	21,181 (54,1%)	0.001	48,025 (55.7%)	48,074 (55.8%)	0.001
Race									
White	25,746 (55.6%)	25,740 (55.6%)	0.000	10,954 (44.2%)	10,940 (44.1%)	0.001	27,648 (45.4%)	27,589 (45.3%)	0.001
Black	6,321 (13.6%)	6,254 (13.5%)	0.003	3,483 (14.0%)	3,471 (14.0%)	0.001	7,551 (12.4%)	7,535 (12.4%)	0.001
Unknown / Missing	13,038 (28.2%)	13,114 (28.3%)	0.003	9,991 (40.3%)	10,009 (40.3%)	0.001	24,779 (40.7%)	24,866 (40.9%)	0.002
Others	1,207 (2.6%)	1,204 (2.6%)	0.000	367 (1.5%)	375 (1.5%)	0.002	868 (1.4%)	856 (1.4%)	0.001
Region / State									
Northeast	10,455 (13.2%)	10,520 (13.2%)	0.002	3,689 (9.4%)	3,703 (9.5%)	0.001	6,789 (7.9%)	6,688 (7.8%)	0.004
Midwest / North central	15,841 (19.9%)	15,989 (20.1%)	0.005	9,574 (24.5%)	9,609 (24.5%)	0.002	18,664 (21.7%)	18,693 (21.7%)	0.001
South	43,242 (54.4%)	43,187 (54.3%)	0.001	20,441 (52.2%)	20,342 (52.0%)	0.005	50,849 (59.0%)	50,808 (58.9%)	0.001
West	9,901 (12.5%)	9,742 (12.3%)	0.006	5,430 (13.9%)	5,477 (14.0%)	0.003	9,831 (11.4%)	9,944 (11.5%)	0.004
Missing	62 (0.1%)	63 (0.1%)	0.000	18 (0.0%)	21 (0.1%)	0.003	58 (0.1%)	58 (0.1%)	0.000
Dual status/Low income subsidy	2,843 (3.6%)	2,876 (3.6%)	0.002	N/A	N/A	N/A	N/A	N/A	N/A
**Lifestyle risk factors**									
Smoking/Tobacco use	15,188 (19.1%)	15,201 (19.1%)	0.000	7,498 (19.2%)	7,465 (19.1%)	0.002	16,213 (18.8%)	16,255 (18.9%)	0.001
Weight, BMI class									
25.0-29.9	13,016 (16.4%)	12,821 (16.1%)	0.007	3,601 (9.2%)	3,610 (9.2%)	0.001	5,712 (6.6%)	5,723 (6.6%)	0.001
30.0-34.9	9,831 (12.4%)	9,751 (12.3%)	0.003	6,388 (16.3%)	6,401 (16.3%)	0.001	12,744 (14.8%)	12,713 (14.7%)	0.001
35.0-39.9	5,791 (7.3%)	5,829 (7.3%)	0.002	8,502 (21.7%)	8,501 (21.7%)	0.000	20,622 (23.9%)	20,589 (23.9%)	0.001
40.0 and above	22,425 (28.2%)	22,492 (28.3%)	0.002	9,925 (25.3%)	9,921 (25.3%)	0.000	24,374 (28.3%)	24,478 (28.4%)	0.003
Unspecified Obesity	27,903 (35.1%)	28,065 (35.3%)	0.004	10,736 (27.4%)	10,719 (27.4%)	0.001	22,739 (26.4%)	22,688 (26.3%)	0.001
**Diabetes complications**									
Diabetic retinopathy	7,018 (8.8%)	6,895 (8.7%)	0.005	3,520 (9.0%)	3,540 (9.0%)	0.002	5,979 (6.9%)	5,944 (6.9%)	0.002
Diabetic neuropathy	20,103 (25.3%)	19,893 (25.0%)	0.006	9,816 (25.1%)	9,731 (24.9%)	0.005	17,753 (20.6%)	17,886 (20.8%)	0.004
Diabetic nephropathy	16,382 (20.6%)	16,090 (20.2%)	0.009	7,859 (20.1%)	7,673 (19.6%)	0.012	14,167 (16.4%)	14,227 (16.5%)	0.002
Diabetes with other ophthalmic complications	2,776 (3.5%)	2,701 (3.4%)	0.005	1,150 (2.9%)	1,176 (3.0%)	0.004	2,455 (2.8%)	2,413 (2.8%)	0.003
Diabetes with peripheral circulatory disorders	9,605 (12.1%)	9,583 (12.1%)	0.001	5,044 (12.9%)	4,988 (12.7%)	0.004	9,627 (11.2%)	9,683 (11.2%)	0.002

Diabetic foot	2,096 (2.6%)	2,073 (2.6%)	0.002	1,106 (2.8%)	1,077 (2.8%)	0.004	1,978 (2.3%)	1,957 (2.3%)	0.002
Erectile dysfunction	3,153 (4.0%)	3,084 (3.9%)	0.004	1,811 (4.6%)	1,827 (4.7%)	0.002	3,900 (4.5%)	3,988 (4.6%)	0.005
Hypoglycemia	15,124 (19.0%)	15,177 (19.1%)	0.002	9,112 (23.3%)	9,024 (23.0%)	0.005	19,509 (22.6%)	19,400 (22.5%)	0.003
Hyperglycemia/DKA/HONK	42,188 (53.1%)	41,857 (52.6%)	0.008	21,187 (54.1%)	21,055 (53.8%)	0.007	42,600 (49.4%)	42,539 (49.4%)	0.001
Skin infections	8,090 (10.2%)	8,056 (10.1%)	0.001	4,104 (10.5%)	3,990 (10.2%)	0.010	8,541 (9.9%)	8,566 (9.9%)	0.001
**Cardiovascular-related conditions**									
Coronary atherosclerosis	16,317 (20.5%)	16,130 (20.3%)	0.006	7,030 (18.0%)	6,973 (17.8%)	0.004	14,415 (16.7%)	14,483 (16.8%)	0.002
Stable angina	3,301 (4.2%)	3,251 (4.1%)	0.003	1,464 (3.7%)	1,472 (3.8%)	0.001	3,052 (3.5%)	3,071 (3.6%)	0.001
Unstable angina	2,046 (2.6%)	2,033 (2.6%)	0.001	809 (2.1%)	796 (2.0%)	0.002	1,610 (1.9%)	1,617 (1.9%)	0.001
Hypertension	68,720 (86.4%)	68,763 (86.5%)	0.002	33,542 (85.7%)	33,459 (85.5%)	0.006	73,529 (85.3%)	73,475 (85.2%)	0.002
Hypotension	2,336 (2.9%)	2,296 (2.9%)	0.003	1,010 (2.6%)	983 (2.5%)	0.004	1,914 (2.2%)	1,865 (2.2%)	0.004
Hyperlipidemia	65,752 (82.7%)	65,555 (82.5%)	0.007	32,542 (83.1%)	32,485 (83.0%)	0.004	71,467 (82.9%)	71,458 (82.9%)	0.000
Acute MI	1,008 (1.3%)	990 (1.2%)	0.002	390 (1.0%)	396 (1.0%)	0.002	671 (0.8%)	692 (0.8%)	0.003
Old MI	3,238 (4.1%)	3,192 (4.0%)	0.003	1,296 (3.3%)	1,288 (3.3%)	0.001	2,513 (2.9%)	2,506 (2.9%)	0.000
Ischemic stroke	909 (1.1%)	926 (1.2%)	0.002	263 (0.7%)	274 (0.7%)	0.003	408 (0.5%)	400 (0.5%)	0.001
TIA	1,243 (1.6%)	1,245 (1.6%)	0.000	572 (1.5%)	577 (1.5%)	0.001	1,109 (1.3%)	1,132 (1.3%)	0.002
Cardiac conduction disorder	4,154 (5.2%)	4,115 (5.2%)	0.002	1,862 (4.8%)	1,863 (4.8%)	0.000	3,779 (4.4%)	3,772 (4.4%)	0.000
Previous cardiac procedure (CABG, PTCA, Stent)	1,537 (1.9%)	1,467 (1.8%)	0.006	555 (1.4%)	544 (1.4%)	0.002	1,029 (1.2%)	1,065 (1.2%)	0.004
PVD diagnosis or surgery	7,128 (9.0%)	7,027 (8.8%)	0.004	3,097 (7.9%)	3,099 (7.9%)	0.000	5,935 (6.9%)	5,900 (6.8%)	0.002
Atrial fibrillation	7,657 (9.6%)	7,722 (9.7%)	0.003	3,167 (8.1%)	3,160 (8.1%)	0.001	6,667 (7.7%)	6,755 (7.8%)	0.004
Other cardiac dysrhythmia	14,397 (18.1%)	14,437 (18.2%)	0.001	6,857 (17.5%)	6,842 (17.5%)	0.001	15,194 (17.6%)	15,379 (17.8%)	0.006
Heart failure	9,656 (12.1%)	9,516 (12.0%)	0.005	4,405 (11.3%)	4,289 (11.0%)	0.009	8,641 (10.0%)	8,761 (10.2%)	0.005
Acute heart failure	2,614 (3.3%)	2,582 (3.2%)	0.002	1,061 (2.7%)	1,058 (2.7%)	0.000	1,943 (2.3%)	1,952 (2.3%)	0.001
Cardiomyopathy	3,661 (4.6%)	3,619 (4.6%)	0.003	1,674 (4.3%)	1,671 (4.3%)	0.000	3,290 (3.8%)	3,346 (3.9%)	0.003
Valve disorders	7,803 (9.8%)	7,812 (9.8%)	0.000	3,444 (8.8%)	3,444 (8.8%)	0.000	7,338 (8.5%)	7,343 (8.5%)	0.000
Valve replacement	915 (1.2%)	925 (1.2%)	0.001	341 (0.9%)	347 (0.9%)	0.002	690 (0.8%)	675 (0.8%)	0.002
Edema	9,313 (11.7%)	9,315 (11.7%)	0.000	4,542 (11.6%)	4,452 (11.4%)	0.007	10,018 (11.6%)	10,029 (11.6%)	0.000
Venous thromboembolism / Pulmonary embolism	2,405 (3.0%)	2,434 (3.1%)	0.002	1,159 (3.0%)	1,143 (2.9%)	0.002	2,417 (2.8%)	2,411 (2.8%)	0.000
Pulmonary hypertension	1,977 (2.5%)	2,009 (2.5%)	0.003	911 (2.3%)	906 (2.3%)	0.001	1,972 (2.3%)	1,962 (2.3%)	0.001
Implantable cardioverter defibrillator	234 (0.3%)	225 (0.3%)	0.002	100 (0.3%)	94 (0.2%)	0.003	184 (0.2%)	203 (0.2%)	0.005
Hyperkalemia	2,048 (2.6%)	2,028 (2.6%)	0.002	1,007 (2.6%)	967 (2.5%)	0.007	1,777 (2.1%)	1,719 (2.0%)	0.005
Cerebrovascular procedure	132 (0.2%)	130 (0.2%)	0.001	36 (0.1%)	39 (0.1%)	0.002	64 (0.1%)	52 (0.1%)	0.005
Insertion of pacemakers / removal of cardiac lead	149 (0.2%)	143 (0.2%)	0.002	47 (0.1%)	51 (0.1%)	0.003	98 (0.1%)	98 (0.1%)	0.000
**Renal-related conditions**									
Hypertensive nephropathy	9,474 (11.9%)	9,344 (11.8%)	0.005	4,327 (11.1%)	4,215 (10.8%)	0.009	7,814 (9.1%)	7,817 (9.1%)	0.000
CKD Stage 1-2	3,603 (4.5%)	3,549 (4.5%)	0.003	1,410 (3.6%)	1,409 (3.6%)	0.000	3,521 (4.1%)	3,583 (4.2%)	0.004
CKD Stage 3-4	11,173 (14.1%)	11,117 (14.0%)	0.002	4,366 (11.2%)	4,287 (10.9%)	0.006	9,317 (10.8%)	9,238 (10.7%)	0.003

Unspecified CKD	4,472 (5.6%)	4,462 (5.6%)	0.001	1,953 (5.0%)	1,912 (4.9%)	0.005	3,491 (4.1%)	3,479 (4.0%)	0.001
Microalbuminuria or proteinuria	3,911 (4.9%)	3,842 (4.8%)	0.004	2,342 (6.0%)	2,317 (5.9%)	0.003	4,556 (5.3%)	4,452 (5.2%)	0.005
Acute kidney injury	5,238 (6.6%)	5,236 (6.6%)	0.000	2,165 (5.5%)	2,087 (5.3%)	0.009	3,729 (4.3%)	3,762 (4.4%)	0.002
Urinary tract infections	10,771 (13.5%)	10,818 (13.6%)	0.002	4,630 (11.8%)	4,625 (11.8%)	0.000	10,137 (11.8%)	10,058 (11.7%)	0.003
Genital infections	3,395 (4.3%)	3,340 (4.2%)	0.003	1,376 (3.5%)	1,359 (3.5%)	0.002	2,646 (3.1%)	2,620 (3.0%)	0.002
Urolithiasis (Kidney and urinary stone)	3,510 (4.4%)	3,495 (4.4%)	0.001	1,701 (4.3%)	1,664 (4.3%)	0.005	3,816 (4.4%)	3,805 (4.4%)	0.001
**Other comorbidities**									
COPD	8,914 (11.2%)	8,911 (11.2%)	0.000	3,906 (10.0%)	3,846 (9.8%)	0.005	7,860 (9.1%)	7,909 (9.2%)	0.002
Asthma	8,083 (10.2%)	8,050 (10.1%)	0.001	4,357 (11.1%)	4,290 (11.0%)	0.005	10,140 (11.8%)	10,145 (11.8%)	0.000
Obstructive sleep apnea	20,290 (25.5%)	20,293 (25.5%)	0.000	11,636 (29.7%)	11,597 (29.6%)	0.002	28,394 (32.9%)	28,358 (32.9%)	0.001
Serious bacterial infections	2,798 (3.5%)	2,804 (3.5%)	0.000	1,140 (2.9%)	1,106 (2.8%)	0.005	1,983 (2.3%)	1,973 (2.3%)	0.001
Pneumonia	4,006 (5.0%)	4,048 (5.1%)	0.002	1,604 (4.1%)	1,576 (4.0%)	0.004	3,133 (3.6%)	3,114 (3.6%)	0.001
Liver disease	11,550 (14.5%)	11,473 (14.4%)	0.003	6,351 (16.2%)	6,236 (15.9%)	0.008	14,548 (16.9%)	14,477 (16.8%)	0.002
MASH / MASLD	6,375 (8.0%)	6,278 (7.9%)	0.005	3,692 (9.4%)	3,630 (9.3%)	0.005	9,214 (10.7%)	9,152 (10.6%)	0.002
Fractures / Falls	3,941 (5.0%)	3,945 (5.0%)	0.000	2,065 (5.3%)	2,021 (5.2%)	0.005	4,067 (4.7%)	4,196 (4.9%)	0.007
Osteoporosis	3,177 (4.0%)	3,221 (4.1%)	0.003	1,214 (3.1%)	1,255 (3.2%)	0.006	2,701 (3.1%)	2,734 (3.2%)	0.002
Osteoarthritis	21,376 (26.9%)	21,391 (26.9%)	0.000	10,325 (26.4%)	10,334 (26.4%)	0.001	23,274 (27.0%)	23,372 (27.1%)	0.003
Depression	15,261 (19.2%)	15,175 (19.1%)	0.003	7,749 (19.8%)	7,679 (19.6%)	0.004	17,010 (19.7%)	17,046 (19.8%)	0.001
Dementia	2,955 (3.7%)	2,909 (3.7%)	0.003	1,053 (2.7%)	1,041 (2.7%)	0.002	1,661 (1.9%)	1,662 (1.9%)	0.000
Delirium or psychosis	1,218 (1.5%)	1,205 (1.5%)	0.001	531 (1.4%)	510 (1.3%)	0.005	883 (1.0%)	875 (1.0%)	0.001
Anxiety	13,744 (17.3%)	13,792 (17.3%)	0.002	8,427 (21.5%)	8,179 (20.9%)	0.015	19,929 (23.1%)	19,771 (22.9%)	0.004
Sleep disorders	24,589 (30.9%)	24,449 (30.8%)	0.004	11,988 (30.6%)	11,931 (30.5%)	0.003	28,221 (32.7%)	28,233 (32.8%)	0.000
Anemia	14,705 (18.5%)	14,618 (18.4%)	0.003	6,526 (16.7%)	6,466 (16.5%)	0.004	14,226 (16.5%)	14,207 (16.5%)	0.001
Influenza	1,389 (1.7%)	1,393 (1.8%)	0.000	709 (1.8%)	712 (1.8%)	0.001	1,956 (2.3%)	1,969 (2.3%)	0.001
COVID	4,830 (6.1%)	5,015 (6.3%)	0.010	4,697 (12.0%)	4,693 (12.0%)	0.000	9,847 (11.4%)	9,944 (11.5%)	0.004
Hyperthyroidism and other thyroid gland disorders	19,154 (24.1%)	19,121 (24.1%)	0.001	9,209 (23.5%)	9,094 (23.2%)	0.007	21,679 (25.2%)	21,772 (25.3%)	0.002
Hypothyroidism	15,441 (19.4%)	15,392 (19.4%)	0.002	7,418 (18.9%)	7,277 (18.6%)	0.009	17,371 (20.2%)	17,404 (20.2%)	0.001
Nephrotic syndrome	59 (0.1%)	58 (0.1%)	0.000	33 (0.1%)	30 (0.1%)	0.003	57 (0.1%)	51 (0.1%)	0.003
Urinary incontinence	3,890 (4.9%)	3,811 (4.8%)	0.005	1,823 (4.7%)	1,821 (4.7%)	0.000	3,909 (4.5%)	3,867 (4.5%)	0.002
Biliary disease	200 (0.3%)	214 (0.3%)	0.003	76 (0.2%)	73 (0.2%)	0.002	129 (0.1%)	123 (0.1%)	0.002
Pancreatitis	115 (0.1%)	104 (0.1%)	0.004	48 (0.1%)	39 (0.1%)	0.007	84 (0.1%)	94 (0.1%)	0.004
Bowel obstruction	114 (0.1%)	115 (0.1%)	0.000	49 (0.1%)	51 (0.1%)	0.001	110 (0.1%)	122 (0.1%)	0.004
Gastroparesis	664 (0.8%)	617 (0.8%)	0.007	318 (0.8%)	326 (0.8%)	0.002	696 (0.8%)	713 (0.8%)	0.002
**Diabetes medications**									
Number of antidiabetic drugs on CED; mean (SD)	2.23 ±0.90	2.22 ±0.87	0.013	2.24 ±1.01	2.24 ±0.98	0.006	2.07 ±0.97	2.07 ±0.96	0.003
Concomitant use or initiation of Metformin	47,654 (59.9%)	47,488 (59.7%)	0.004	20,297 (51.8%)	20,367 (52.0%)	0.004	42,087 (48.8%)	42,204 (49.0%)	0.003
Concomitant use or initiation of Insulins	14,450 (18.2%)	14,038 (17.7%)	0.014	8,197 (20.9%)	8,115 (20.7%)	0.005	13,932 (16.2%)	13,906 (16.1%)	0.001

Concomitant use or initiation of Sulfonylureas	18,417 (23.2%)	18,366 (23.1%)	0.002	6,974 (17.8%)	6,911 (17.7%)	0.004	11,495 (13.3%)	11,418 (13.2%)	0.003
Concomitant use or initiation of DPP-4i	N/A	N/A	N/A	2,725 (7.0%)	2,691 (6.9%)	0.003	2,899 (3.4%)	2,846 (3.3%)	0.003
Concomitant use or initiation of SGLT-2i	12,234 (15.4%)	11,864 (14.9%)	0.013	8,143 (20.8%)	8,161 (20.8%)	0.001	15,897 (18.4%)	15,928 (18.5%)	0.001
Concomitant use or initiation of Any other glucose-lowering drugs	4,865 (6.1%)	4,835 (6.1%)	0.002	2,316 (5.9%)	2,307 (5.9%)	0.001	4,370 (5.1%)	4,384 (5.1%)	0.001
Past use of Metformin	57,422 (72.2%)	57,260 (72.0%)	0.005	26,869 (68.6%)	26,890 (68.7%)	0.001	56,572 (65.6%)	56,653 (65.7%)	0.002
Past use of Insulins	19,283 (24.3%)	18,833 (23.7%)	0.013	11,011 (28.1%)	10,896 (27.8%)	0.007	18,681 (21.7%)	18,598 (21.6%)	0.002
Past use of Sulfonylureas	24,974 (31.4%)	24,916 (31.3%)	0.002	9,990 (25.5%)	9,843 (25.1%)	0.009	16,778 (19.5%)	16,762 (19.4%)	0.000
Past use of DPP-4i	N/A	N/A	N/A	4,391 (11.2%)	4,347 (11.1%)	0.004	4,893 (5.7%)	4,785 (5.6%)	0.005
Past use of SGLT-2i	17,101 (21.5%)	16,678 (21.0%)	0.013	11,061 (28.3%)	11,049 (28.2%)	0.001	21,707 (25.2%)	21,687 (25.2%)	0.001
Past use of Any other glucose-lowering drugs	7,051 (8.9%)	7,065 (8.9%)	0.001	3,314 (8.5%)	3,268 (8.3%)	0.004	6,190 (7.2%)	6,185 (7.2%)	0.000
**Other medications**									
ACE / ARB	59,870 (75.3%)	59,907 (75.4%)	0.001	28,583 (73.0%)	28,472 (72.7%)	0.006	61,304 (71.1%)	61,148 (70.9%)	0.004
ARNI	864 (1.1%)	861 (1.1%)	0.000	627 (1.6%)	624 (1.6%)	0.001	1,347 (1.6%)	1,378 (1.6%)	0.003
Thiazides	27,881 (35.1%)	27,961 (35.2%)	0.002	13,220 (33.8%)	13,261 (33.9%)	0.002	30,433 (35.3%)	30,394 (35.3%)	0.001
Beta-blockers	33,255 (41.8%)	33,333 (41.9%)	0.002	15,200 (38.8%)	15,182 (38.8%)	0.001	32,384 (37.6%)	32,591 (37.8%)	0.005
Calcium channel blockers	26,321 (33.1%)	26,403 (33.2%)	0.002	12,321 (31.5%)	12,286 (31.4%)	0.002	26,196 (30.4%)	26,243 (30.4%)	0.001
Digoxin / Digitoxin	721 (0.9%)	706 (0.9%)	0.002	184 (0.5%)	200 (0.5%)	0.006	378 (0.4%)	351 (0.4%)	0.005
Loop diuretics	13,878 (17.5%)	13,927 (17.5%)	0.002	6,147 (15.7%)	6,040 (15.4%)	0.008	12,882 (14.9%)	12,893 (15.0%)	0.000
Other diuretics	6,442 (8.1%)	6,421 (8.1%)	0.001	3,347 (8.5%)	3,342 (8.5%)	0.000	7,464 (8.7%)	7,526 (8.7%)	0.003
Intravenous diuretics	1,007 (1.3%)	1,001 (1.3%)	0.001	437 (1.1%)	446 (1.1%)	0.002	880 (1.0%)	916 (1.1%)	0.004
Nitrates	5,322 (6.7%)	5,234 (6.6%)	0.004	2,199 (5.6%)	2,202 (5.6%)	0.000	4,173 (4.8%)	4,135 (4.8%)	0.002
Anti-arrhythmics	1,800 (2.3%)	1,823 (2.3%)	0.002	697 (1.8%)	746 (1.9%)	0.009	1,705 (2.0%)	1,707 (2.0%)	0.000
Statins	63,368 (79.7%)	63,301 (79.6%)	0.002	31,016 (79.2%)	31,109 (79.5%)	0.006	64,885 (75.3%)	64,817 (75.2%)	0.002
PCSK9 inhibitors and other lipid-lowering drugs	10,492 (13.2%)	10,370 (13.0%)	0.005	5,262 (13.4%)	5,154 (13.2%)	0.008	11,539 (13.4%)	11,571 (13.4%)	0.001
Antiplatelet medications	8,847 (11.1%)	8,786 (11.1%)	0.002	3,764 (9.6%)	3,723 (9.5%)	0.004	7,159 (8.3%)	7,239 (8.4%)	0.003
Oral anticoagulants	8,007 (10.1%)	8,055 (10.1%)	0.002	3,538 (9.0%)	3,540 (9.0%)	0.000	7,402 (8.6%)	7,426 (8.6%)	0.001
COPD/Asthma medications	26,536 (33.4%)	26,463 (33.3%)	0.002	13,489 (34.5%)	13,344 (34.1%)	0.008	30,956 (35.9%)	30,907 (35.9%)	0.001
NSAIDS	26,168 (32.9%)	26,171 (32.9%)	0.000	12,818 (32.7%)	12,802 (32.7%)	0.001	29,544 (34.3%)	29,645 (34.4%)	0.002
Oral corticosteroids	17,735 (22.3%)	17,876 (22.5%)	0.004	9,494 (24.2%)	9,504 (24.3%)	0.001	24,161 (28.0%)	24,118 (28.0%)	0.001
Osteoporosis agents (incl. bisphosphonates)	2,085 (2.6%)	2,096 (2.6%)	0.001	856 (2.2%)	871 (2.2%)	0.003	1,779 (2.1%)	1,786 (2.1%)	0.001
Opioids	24,303 (30.6%)	24,100 (30.3%)	0.006	11,132 (28.4%)	11,088 (28.3%)	0.002	25,240 (29.3%)	25,265 (29.3%)	0.001
Anti-depressants	27,710 (34.9%)	27,612 (34.7%)	0.003	15,062 (38.5%)	14,840 (37.9%)	0.012	33,833 (39.3%)	33,810 (39.2%)	0.001
Antipsychotics	3,061 (3.9%)	2,966 (3.7%)	0.006	1,707 (4.4%)	1,642 (4.2%)	0.008	3,568 (4.1%)	3,556 (4.1%)	0.001
Anxiolytics / hypnotics, benzos	15,340 (19.3%)	15,472 (19.5%)	0.004	7,976 (20.4%)	7,842 (20.0%)	0.009	19,181 (22.3%)	19,186 (22.3%)	0.000
Dementia medications	1,417 (1.8%)	1,367 (1.7%)	0.005	450 (1.1%)	435 (1.1%)	0.004	700 (0.8%)	736 (0.9%)	0.005
Urinary tract infections antibiotics	36,563 (46.0%)	36,475 (45.9%)	0.002	17,746 (45.3%)	17,734 (45.3%)	0.001	40,944 (47.5%)	40,896 (47.4%)	0.001
Laxatives	2,571 (3.2%)	2,591 (3.3%)	0.001	1,278 (3.3%)	1,292 (3.3%)	0.002	2,925 (3.4%)	2,912 (3.4%)	0.001

**Healthcare utilization marker**									
Number of distinct medications; mean (SD)	14.04 ± 6.59	13.98 ± 6.83	0.009	14.40 ± 6.95	14.31 ± 6.92	0.013	14.22 ± 7.05	14.22 ± 7.03	0.000
Number of office visits; mean (SD)	9.44 ± 6.90	9.38 ± 7.24	0.008	9.02 ± 6.57	8.99 ± 6.83	0.005	9.28 ± 6.93	9.27 ± 6.97	0.002
Number of endocrinologist visits; mean (SD)	0.43 ± 1.20	0.41 ± 1.54	0.016	0.44 ± 1.31	0.44 ± 1.43	0.005	0.42 ± 1.34	0.42 ± 1.37	0.000
Number of cardiologist visits; mean (SD)	1.46 ± 3.37	1.45 ± 3.31	0.005	1.29 ± 2.97	1.30 ± 3.16	0.002	1.28 ± 2.99	1.29 ± 3.07	0.004
Number of internal/family medicine visits; mean (SD)	6.71 ± 7.90	6.65 ± 7.76	0.007	6.35 ± 7.79	6.31 ± 7.57	0.005	6.06 ± 7.00	6.04 ± 6.88	0.003
Number of electrocardiograms (ECG/EKG) ; mean (SD)	1.00 ± 1.74	1.00 ± 1.73	0.002	0.93 ± 1.60	0.93 ± 1.64	0.000	0.89 ± 1.93	0.89 ± 1.84	0.004
Number of echocardiograms; mean (SD)	0.29 ± 0.67	0.29 ± 0.68	0.000	0.28 ± 0.63	0.28 ± 0.68	0.000	0.26 ± 0.93	0.27 ± 0.98	0.011
Out-of-pocket medication cost; mean (SD)	566.85 ± 692.83	561.76 ± 787.43	0.007	578.40 ± 784.43	574.50 ± 857.82	0.005	583.99 ± 831.09	578.25 ± 871.37	0.007
Unique brand medicines; mean (SD)	14.32 ± 6.81	14.26 ± 7.07	0.009	14.65 ± 7.17	14.56 ± 7.14	0.013	14.45 ± 7.25	14.45 ± 7.21	0.000
Unique generic medicines; mean (SD)	14.04 ± 6.59	13.98 ± 6.83	0.009	14.40 ± 6.95	14.31 ± 6.92	0.013	14.22 ± 7.05	14.22 ± 7.03	0.000
Ratio of brand to generic medications; mean (SD)	1.02 ± 0.04	1.02 ± 0.04	0.000	1.02 ± 0.04	1.02 ± 0.04	0.000	1.01 ± 0.04	1.01 ± 0.04	0.000
**Healthy behavior markers**									
Colonoscopy / Sigmoidoscopy	7,905 (9.9%)	7,919 (10.0%)	0.001	4,034 (10.3%)	4,047 (10.3%)	0.001	9,377 (10.9%)	9,251 (10.7%)	0.005
Flu Pneumococcal vaccine	23,902 (30.1%)	23,969 (30.1%)	0.002	12,399 (31.7%)	12,410 (31.7%)	0.001	24,895 (28.9%)	24,898 (28.9%)	0.000
Pap smear	5,817 (7.3%)	5,878 (7.4%)	0.003	3,016 (7.7%)	3,061 (7.8%)	0.004	8,081 (9.4%)	8,064 (9.4%)	0.001
PSA test	17,161 (21.6%)	17,161 (21.6%)	0.000	8,323 (21.3%)	8,265 (21.1%)	0.004	19,190 (22.3%)	19,128 (22.2%)	0.002
Fecal occult blood test	4,498 (5.7%)	4,494 (5.7%)	0.000	1,494 (3.8%)	1,469 (3.8%)	0.003	3,130 (3.6%)	3,102 (3.6%)	0.002
Bone mineral density tests	5,064 (6.4%)	5,122 (6.4%)	0.003	2,118 (5.4%)	2,226 (5.7%)	0.012	5,365 (6.2%)	5,346 (6.2%)	0.001
Mammograms	19,056 (24.0%)	19,162 (24.1%)	0.003	9,746 (24.9%)	9,830 (25.1%)	0.005	23,658 (27.4%)	23,538 (27.3%)	0.003
Telemedicine	17,215 (21.7%)	17,097 (21.5%)	0.004	9,743 (24.9%)	9,719 (24.8%)	0.001	21,862 (25.4%)	21,958 (25.5%)	0.003
**Laboratory and diagnostic tests**									
HbA1c tests; mean (SD)	2.39 ± 1.50	2.38 ± 1.84	0.004	2.41 ± 1.38	2.41 ± 1.32	0.000	2.27 ± 1.35	2.26 ± 1.35	0.007
Lipid panels; mean (SD)	1.66 ± 1.32	1.66 ± 1.54	0.005	1.57 ± 1.15	1.57 ± 1.18	0.001	1.63 ± 1.15	1.63 ± 1.20	0
Creatinine tests; mean (SD)	1.38 ± 2.65	1.37 ± 2.67	0.002	1.87 ± 2.82	1.87 ± 2.59	0.002	1.96 ± 2.68	1.96 ± 2.58	0.003
Natriuretic peptide tests; mean (SD)	0.18 ± 0.95	0.19 ± 1.67	0.001	0.16 ± 0.70	0.15 ± 0.77	0.014	0.15 ± 0.66	0.15 ± 0.70	0.000
Urine tests; mean (SD)	1.15 ± 2.02	1.14 ± 2.32	0.002	0.97 ± 1.60	0.97 ± 1.73	0.000	0.99 ± 1.61	0.98 ± 1.58	0.003
**Lab values**									
HbA1c (%)*; mean (SD) median [IQR]	7.88 ±1.78 7.50 [6.60, 8.80]	8.04 ±1.66 7.70 [6.90, 8.80]	0.094	7.81 ±1.77 7.40 [6.50, 8.70]	8.13 ±1.83 7.80 [6.80, 9.10]	0.178	7.59 ±1.71 7.10 [6.40, 8.40]	7.57 ±1.70 7.10 [6.40, 8.30]	0.012
Glucose (mg/dl)*; mean (SD) median [IQR]	165.78 ±72.80 148.00 [117.00, 195.00]	171.64 ±71.59 156.00 [125.00, 199.00]	0.081	161.44 ±70.21 143.50 [114.00, 189.00]	876.61 ±83,854.72 155.00 [121.00, 206.00]	0.012	154.88 ±66.82 137.00 [111.00, 179.00]	155.14 ±67.04 137.00 [111.00, 179.00]	0.004
Creatinine (mg/dl)*; mean (SD) median [IQR]	1.06 ±1.37 0.93 [0.80, 1.15]	1.07 ±1.64 0.93 [0.80, 1.16]	0.008	1.05 ±1.94 0.91 [0.80, 1.12]	1.04 ±1.14 0.91 [0.80, 1.12]	0.006	1.01 ±1.33 0.89 [0.80, 1.08]	1.00 ±0.97 0.89 [0.80, 1.08]	0.005
Systolic blood pressure (mmHg)*; mean (SD) median [IQR]	130.99 ±18.36 130.00 [121.00, 140.00]	130.94 ±19.11 130.00	0.003	129.90 ±22.63 130.00	130.13 ±27.13 130.00	0.009	129.33 ±22.75 130.00	129.21 ±21.25 130.00 [120.00, 139.00]	0.005

		[121.00, 140.00]		[120.00, 140.00]	[120.00, 140.00]		[120.00, 139.00]		
Heart rate (1/min)*; mean (SD) median [IQR]	77.62 ±17.24 76.00 [68.00, 86.00]	78.02 ±16.84 77.00 [68.00, 86.00]	0.023	78.37 ±14.56 77.00 [69.00, 87.00]	78.81 ±18.29 78.00 [69.00, 87.00]	0.027	78.65 ±14.98 78.00 [69.00, 87.00]	78.50 ±13.92 77.00 [69.00, 87.00]	0.011
BMI (kg/m2)*; mean (SD) median [IQR]	35.65 ±7.22 34.30 [30.45, 39.27]	34.45 ±6.72 33.19 [29.64, 37.87]	0.172	37.78 ±7.93 36.42 [32.12, 41.66]	36.88 ±7.60 35.60 [31.46, 40.81]	0.116	38.65 ±8.06 37.30 [32.97, 42.74]	38.19 ±7.77 36.92 [32.80, 42.13]	0.057
eGFR (ml/min/1.73m2)*; mean (SD) median [IQR]	1,293.41 ±110,565.65 72.00 [55.00, 89.00]	688.51 ±55,585.66 72.00 [54.00, 89.00]	0.007	2,618.84 ±159,419.94 79.00 [60.00, 95.00]	1,886.79 ±134,527.43 79.00 [60.00, 96.00]	0.005	2,676.77 ±161,118.82 83.00 [65.00, 98.00]	1,070.00 ±99,497.99 82.00 [64.00, 97.00]	0.012
LDL (mg/dl)*; mean (SD) median [IQR]	82.82 ±38.25 78.00 [59.00, 103.00]	83.91 ±37.73 79.00 [60.00, 104.00]	0.029	85.07 ±39.55 81.00 [60.00, 107.00]	84.53 ±39.87 80.00 [59.00, 106.00]	0.013	87.22 ±39.47 83.00 [62.00, 110.00]	87.22 ±39.48 83.00 [62.00, 110.00]	0.000
HDL (mg/dl)*; mean (SD) median [IQR]	46.44 ±14.08 45.00 [37.00, 54.00]	45.78 ±13.48 44.00 [37.00, 53.00]	0.048	45.76 ±13.91 44.00 [37.00, 53.00]	45.04 ±13.77 44.00 [36.00, 52.00]	0.052	46.02 ±13.85 45.00 [37.00, 53.00]	46.20 ±14.10 45.00 [37.00, 54.00]	0.013
Total cholesterol (mg/dl)*; mean (SD) median [IQR]	161.73 ±46.70 156.00 [132.00, 187.00]	162.19 ±47.32 157.00 [132.00, 187.00]	0.01	162.99 ±50.08 158.00 [133.00, 189.00]	163.02 ±51.02 157.00 [131.00, 190.00]	0.001	165.35 ±49.50 161.00 [135.00, 192.00]	165.31 ±49.04 161.00 [135.00, 193.00]	0.001
Triglyceride (mg/dl)*; mean (SD) median [IQR]	172.44 ±130.39 144.00 [103.00, 204.00]	172.59 ±139.71 144.00 [104.00, 204.00]	0.001	176.96 ±151.29 144.00 [103.00, 207.00]	182.12 ±166.70 148.00 [105.00, 210.00]	0.032	176.00 ±150.72 144.00 [104.00, 205.00]	175.09 ±154.99 144.00 [103.00, 204.00]	0.006
Burden of comorbidities; mean (SD)	1.85 ±2.39	1.84 ±2.37	0.007	1.70 ±2.26	1.68 ±2.22	0.011	1.53 ±2.13	1.54 ±2.13	0.005
Frailty Score; mean (SD)	0.17 ±0.06	0.17 ±0.06	0.000	0.16 ±0.06	0.16 ±0.06	0	0.15 ±0.05	0.15 ±0.05	0
**Baseline hospitalizations and hospital metrics**									
Number of hospitalizations; mean (SD)	2.54 ± 21.95	2.42 ± 17.31	0.006	1.81 ± 13.73	1.75 ± 14.13	0.00	2.54 ± 21.95	2.42 ± 17.31	0.006
Any hospitalization within prior 91 days	4,618 (5.8%)	4,509 (5.7%)	0.006	1,555 (4.0%)	1,539 (3.9%)	0.002	2,710 (3.1%)	2,715 (3.1%)	0.000
Any hospitalization within prior 92-365 days	8,809 (11.1%)	8,761 (11.0%)	0.002	3,401 (8.7%)	3,355 (8.6%)	0.004	6,566 (7.6%)	6,590 (7.6%)	0.001
Number of hospitalizations (0, 1, 2 or more)									
< 1	67,765 (85.2%)	67,846 (85.3%)	0.003	34,759 (88.8%)	34,798 (88.9%)	0.003	77,843 (90.3%)	77,792 (90.3%)	0.002
1 - <2	5,640 (7.1%)	5,641 (7.1%)	0.000	2,234 (5.7%)	2,239 (5.7%)	0.001	4,853 (5.6%)	4,898 (5.7%)	0.002
>= 2	6,096 (7.7%)	6,014 (7.6%)	0.004	2,159 (5.5%)	2,115 (5.4%)	0.005	3,495 (4.1%)	3,501 (4.1%)	0.000
Heart failure hospitalization	3,329 (4.2%)	3,275 (4.1%)	0.003	1,154 (2.9%)	1,153 (2.9%)	0.000	2,051 (2.4%)	2,100 (2.4%)	0.004
ED visit	26,726 (33.6%)	26,693 (33.6%)	0.001	11,738 (30.0%)	11,570 (29.6%)	0.009	22,894 (26.6%)	23,039 (26.7%)	0.004
**Calendar year of cohort entry**									
2018	3,519 (4.4%)	3,574 (4.5%)	0.003	N/A	N/A	N/A	N/A	N/A	N/A
2019	15,815 (19.9%)	15,567 (19.6%)	0.008	N/A	N/A	N/A	N/A	N/A	N/A
2020	17,861 (22.5%)	17,742 (22.3%)	0.004	N/A	N/A	N/A	N/A	N/A	N/A
2021	13,322 (16.8%)	13,268 (16.7%)	0.002	N/A	N/A	N/A	N/A	N/A	N/A
2022	11,982 (15.1%)	12,115 (15.2%)	0.005	8,863 (22.6%)	8,884 (22.7%)	0.001	10,029 (11.6%)	9,803 (11.4%)	0.008

2023	10,736 (13.5%)	10,879 (13.7%)	0.005	22,676 (57.9%)	22,685 (57.9%)	0.000	45,525 (52.8%)	45,620 (52.9%)	0.002
2024	6,225 (7.8%)	6,323 (8.0%)	0.005	7,473 (19.1%)	7,442 (19.0%)	0.002	29,382 (34.1%)	29,553 (34.3%)	0.004
2025	41 (0.1%)	33 (0.0%)	0.005	140 (0.4%)	141 (0.4%)	0.000	1,255 (1.5%)	1,215 (1.4%)	0.004

Abbreviations: * not used in the propensity score; ACE, angiotensin-converting enzyme inhibitors; ARB, angiotensin receptor blocker; ARNI, angiotensin receptor/neprilysin inhibitor; BMI, body mass index; BNP, brain natriuretic peptide; CABG, coronary artery bypass graft surgery; COPD, chronic obstructive pulmonary disease; DKA, diabetic ketoacidosis; DPP4i, dipeptidyl peptidase-4 inhibitors; eGFR, estimated glomerular filtration rate; HbA1c, hemoglobin A1c; HONK, hyperglycemic hyperosmolar nonketotic state; MASH, metabolic dysfunction associated steatohepatitis; MASLD, metabolic dysfunction associated steatotic liver disease; N, number of participants; NSAIDs, non-steroidal anti-inflammatory drugs; PCSK9, proprotein convertase subtilisin/kexin type 9; proBNP, pro-b-type natriuretic peptide; PSA, prostate-specific antigen; PTCA, percutaneous transluminal coronary angioplasty; SGLT2i, sodium-glucose transport protein 2 inhibitors; SMD, standardized mean difference

Missing data were handled by assuming absence of a code indicated absence of the condition for most binary covariates. Missing indicators were included for race, region, combined comorbidity index, and frailty score in the propensity score model.

Missing data were handled by assuming absence of a code indicated absence of the condition for most binary covariates. Missing indicators were included for race and region in the propensity score model.

Laboratory values were only available in a subset of the Optum database and were truncated using clinically plausible cut-off values (BMI > 100 or < 10 set to missing; missingness ≈ 63% for the semaglutide vs sitagliptin comparison / 54% for the tirzepatide vs dulaglutide comparison / 53% for the tirzepatide vs semaglutide comparison. Creatinine > 30 set to missing and < 0.8 set to 0.8; missingness ≈ 41% / 41% / 40%. eGFR > 150 set to 150 and = 0 set to missing; missingness ≈ 71% / 53% / 51%. Glucose < 30 set to missing; missingness ≈ 41% / 41% / 40%. HbA1c ≥ 20 or ≤ 2 set to missing; missingness ≈ 37% / 36% / 34%. HDL missingness ≈ 48% / 48% / 47%. Heart rate < 30 set to missing; missingness ≈ 75% / 67% / 66%. LDL missingness ≈ 46% / 46% / 45%. Systolic blood pressure < 30 set to missing; missingness ≈ 61% / 51% / 50%. Total cholesterol missingness ≈ 48% / 48% / 47%. Triglyceride missingness ≈ 49% / 49% / 48%).

The Race-Others category includes Asian and Hispanic individuals.

Supplementary Table 2. Baseline characteristics of initiators of semaglutide vs sitagliptin when applying trial eligibility criteria before and after propensity score matching, pooled across databases. Values are number (percentage) unless otherwise specified.

	Before propensity score matching			After propensity score matching		
Variable	Semaglutide (n = 77,536)	Sitagliptin (n = 80,774)	SMD	Semaglutide (n = 27,033)	Sitagliptin (n = 27,033)	SMD
**Demographics**						
Age; mean (SD)	66.27 ± 8.19	72.75 ± 8.41	0.79	69.11 ± 7.98	69.15 ± 8.21	0.005
Gender						
Male	34,957 (45.1%)	36,769 (45.5%)	0.009	12,668 (46.9%)	12,617 (46.7%)	0.004
Female	42,579 (54.9%)	44,005 (54.5%)	0.009	14,365 (53.1%)	14,416 (53.3%)	0.004
Race						
White	31,346 (54.2%)	42,993 (64.3%)	0.259	12,320 (60.2%)	12,325 (60.2%)	0
Black	7,196 (12.4%)	7,519 (11.2%)	0.001	2,454 (12.0%)	2,447 (12.0%)	0.003
Unknown / Missing	18,280 (31.6%)	13,804 (20.6%)	0.162	5,151 (25.2%)	5,166 (25.2%)	0.002
Others	1,001 (1.7%)	2,573 (3.9%)	0.128	554 (2.7%)	541 (2.6%)	0.001
Region / State						
Northeast	8,524 (11.0%)	13,108 (16.2%)	0.153	3,581 (13.2%)	3,647 (13.5%)	0.007
Midwest / North central	15,930 (20.5%)	16,518 (20.4%)	0.002	5,518 (20.4%)	5,471 (20.2%)	0.004
South	42,434 (54.7%)	39,763 (49.2%)	0.110	14,395 (53.2%)	14,405 (53.3%)	0.001
West	10,610 (13.7%)	11,279 (14.0%)	0.008	3,524 (13.0%)	3,499 (12.9%)	0.003
Missing	38 (0.0%)	106 (0.1%)	0.027	15 (0.1%)	11 (0.0%)	0.007
Dual status/Low income subsidy	1,619 (2.1%)	11,484 (14.2%)	0.455	1,473 (5.4%)	1,494 (5.5%)	0.003
**Lifestyle risk factors**						
Smoking/Tobacco use	18,696 (24.1%)	19,642 (24.3%)	0.005	6,246 (23.1%)	6,277 (23.2%)	0.003
Weight						
Underweight	111 (0.1%)	1,289 (1.6%)	0.157	101 (0.4%)	93 (0.3%)	0.005
Normal weight	1,118 (1.4%)	7,265 (9.0%)	0.345	910 (3.4%)	913 (3.4%)	0.001
Overweight (BMI 25.0-29.9)	8,887 (11.5%)	22,152 (27.4%)	0.412	5,232 (19.4%)	5,256 (19.4%)	0.002
Class 1 Obesity (BMI 30.0-34.9)	7,561 (9.8%)	8,795 (10.9%)	0.037	3,123 (11.6%)	3,177 (11.8%)	0.006
Class 2 Obesity (BMI 35.0-39.9)	5,113 (6.6%)	4,033 (5.0%)	0.069	1,723 (6.4%)	1,674 (6.2%)	0.007
Class 3 Obesity; (BMI 40.0 and above)	26,815 (34.6%)	14,273 (17.7%)	0.392	7,031 (26.0%)	6,994 (25.9%)	0.003
Unspecified Obesity	27,931 (36.0%)	22,967 (28.4%)	0.163	8,913 (33.0%)	8,926 (33.0%)	0.001
**Diabetes complications**						
Diabetic retinopathy	6,376 (8.2%)	7,894 (9.8%)	0.054	2,677 (9.9%)	2,639 (9.8%)	0.005
Diabetic neuropathy	21,085 (27.2%)	24,001 (29.7%)	0.056	8,203 (30.3%)	8,138 (30.1%)	0.005
Diabetic nephropathy	18,453 (23.8%)	24,122 (29.9%)	0.137	7,438 (27.5%)	7,466 (27.6%)	0.002
Diabetes with other ophthalmic complications	2,961 (3.8%)	3,201 (4.0%)	0.007	1,133 (4.2%)	1,145 (4.2%)	0.002
Diabetes with peripheral circulatory disorders	11,323 (14.6%)	11,885 (14.7%)	0.003	4,126 (15.3%)	4,121 (15.2%)	0.001
Diabetic foot	2,085 (2.7%)	2,606 (3.2%)	0.032	792 (2.9%)	780 (2.9%)	0.003

Erectile dysfunction	3,539 (4.6%)	2,199 (2.7%)	0.098	1,026 (3.8%)	1,034 (3.8%)	0.002
Hypoglycemia	16,673 (21.5%)	14,361 (17.8%)	0.094	5,449 (20.2%)	5,409 (20.0%)	0.004
Hyperglycemia/DKA/HONK	37,205 (48.0%)	38,980 (48.3%)	0.005	13,944 (51.6%)	13,770 (50.9%)	0.013
Skin infections	8,277 (10.7%)	8,736 (10.8%)	0.005	2,815 (10.4%)	2,832 (10.5%)	0.002
Cardiovascular-related conditions						
Coronary atherosclerosis	28,758 (37.1%)	31,980 (39.6%)	0.051	10,140 (37.5%)	10,156 (37.6%)	0.001
Stable angina	5,545 (7.2%)	5,084 (6.3%)	0.034	1,845 (6.8%)	1,852 (6.9%)	0.001
Unstable angina	2,966 (3.8%)	3,171 (3.9%)	0.005	1,048 (3.9%)	1,068 (4.0%)	0.004
Hypertension	70,539 (91.0%)	73,774 (91.3%)	0.013	24,723 (91.5%)	24,713 (91.4%)	0.001
Hypotension	2,703 (3.5%)	4,212 (5.2%)	0.085	1,094 (4.0%)	1,066 (3.9%)	0.005
Hyperlipidemia	66,789 (86.1%)	66,425 (82.2%)	0.107	23,047 (85.3%)	22,977 (85.0%)	0.007
Acute MI	1,009 (1.3%)	1,300 (1.6%)	0.026	379 (1.4%)	384 (1.4%)	0.002
Old MI	4,901 (6.3%)	6,308 (7.8%)	0.058	1,793 (6.6%)	1,877 (6.9%)	0.012
Ischemic stroke	722 (0.9%)	1,370 (1.7%)	0.067	317 (1.2%)	324 (1.2%)	0.002
TIA	2,102 (2.7%)	2,938 (3.6%)	0.053	773 (2.9%)	769 (2.8%)	0.001
Cardiac conduction disorder	5,803 (7.5%)	7,264 (9.0%)	0.055	2,078 (7.7%)	2,027 (7.5%)	0.007
Previous cardiac procedure (CABG, PTCA, Stent)	2,116 (2.7%)	2,198 (2.7%)	0.000	767 (2.8%)	770 (2.8%)	0.001
PVD diagnosis or surgery	8,697 (11.2%)	11,149 (13.8%)	0.078	3,364 (12.4%)	3,359 (12.4%)	0.001
Atrial fibrillation	11,374 (14.7%)	15,092 (18.7%)	0.108	4,138 (15.3%)	4,250 (15.7%)	0.011
Other cardiac dysrhythmia	19,564 (25.2%)	21,616 (26.8%)	0.035	6,615 (24.5%)	6,621 (24.5%)	0.001
Heart failure	15,576 (20.1%)	18,439 (22.8%)	0.067	5,403 (20.0%)	5,442 (20.1%)	0.004
Acute heart failure	3,309 (4.3%)	5,341 (6.6%)	0.104	1,258 (4.7%)	1,289 (4.8%)	0.005
Cardiomyopathy	5,795 (7.5%)	6,503 (8.1%)	0.022	1,963 (7.3%)	2,031 (7.5%)	0.010
Valve disorders	11,626 (15.0%)	13,824 (17.1%)	0.058	4,105 (15.2%)	4,084 (15.1%)	0.002
Valve replacement	1,395 (1.8%)	1,953 (2.4%)	0.043	567 (2.1%)	547 (2.0%)	0.005
Edema	11,956 (15.4%)	12,313 (15.2%)	0.005	4,000 (14.8%)	4,061 (15.0%)	0.006
Venous thromboembolism / Pulmonary embolism	3,012 (3.9%)	3,343 (4.1%)	0.013	1,034 (3.8%)	1,051 (3.9%)	0.003
Pulmonary hypertension	2,971 (3.8%)	3,626 (4.5%)	0.033	1,018 (3.8%)	1,022 (3.8%)	0.001
Implantable cardioverter defibrillator	322 (0.4%)	470 (0.6%)	0.024	116 (0.4%)	120 (0.4%)	0.002
Hyperkalemia	2,080 (2.7%)	2,666 (3.3%)	0.036	827 (3.1%)	837 (3.1%)	0.002
Cerebrovascular procedure	114 (0.1%)	184 (0.2%)	0.019	Cardiovascular-related52 (0.2%)	50 (0.2%)	0.002
Insertion of pacemakers / removal of cardiac lead	173 (0.2%)	369 (0.5%)	0.040	80 (0.3%)	81 (0.3%)	0.001
Renal-related conditions						
Hypertensive nephropathy	11,777 (15.2%)	16,139 (20.0%)	0.126	4,699 (17.4%)	4,713 (17.4%)	0.001
CKD Stage 1-2	3,933 (5.1%)	4,514 (5.6%)	0.023	1,474 (5.5%)	1,501 (5.6%)	0.004
CKD Stage 3-4	15,933 (20.5%)	20,333 (25.2%)	0.110	6,344 (23.5%)	6,346 (23.5%)	0.000
Unspecified CKD	4,951 (6.4%)	7,662 (9.5%)	0.115	2,053 (7.6%)	2,067 (7.6%)	0.002
Microalbuminuria or proteinuria	1,648 (2.1%)	1,607 (2.0%)	0.010	608 (2.2%)	626 (2.3%)	0.004
Acute kidney injury	5,097 (6.6%)	9,338 (11.6%)	0.174	2,266 (8.4%)	2,272 (8.4%)	0.001

Urinary tract infections	10,888 (14.0%)	16,407 (20.3%)	0.167	4,275 (15.8%)	4,346 (16.1%)	0.007
Genital infections	2,329 (3.0%)	2,546 (3.2%)	0.009	951 (3.5%)	935 (3.5%)	0.003
Urolithiasis (Kidney and urinary stone)	4,082 (5.3%)	4,111 (5.1%)	0.008	1,429 (5.3%)	1,374 (5.1%)	0.009
Other comorbidities						
COPD	11,713 (15.1%)	14,129 (17.5%)	0.065	4,256 (15.7%)	4,219 (15.6%)	0.004
Asthma	10,121 (13.1%)	8,011 (9.9%)	0.098	3,055 (11.3%)	3,025 (11.2%)	0.004
Obstructive sleep apnea	29,037 (37.4%)	17,241 (21.3%)	0.359	7,891 (29.2%)	7,797 (28.8%)	0.008
Serious bacterial infections	2,442 (3.1%)	5,012 (6.2%)	0.145	1,057 (3.9%)	1,074 (4.0%)	0.003
Pneumonia	4,332 (5.6%)	6,410 (7.9%)	0.094	1,656 (6.1%)	1,697 (6.3%)	0.006
Liver disease	13,027 (16.8%)	10,623 (13.2%)	0.102	4,067 (15.0%)	4,128 (15.3%)	0.006
MASH / MASLD	7,257 (9.4%)	4,551 (5.6%)	0.142	2,044 (7.6%)	2,043 (7.6%)	0.000
Fractures / Falls	4,630 (6.0%)	5,451 (6.7%)	0.032	1,633 (6.0%)	1,660 (6.1%)	0.004
Osteoporosis	3,854 (5.0%)	5,755 (7.1%)	0.090	1,527 (5.6%)	1,525 (5.6%)	0.000
Osteoarthritis	26,856 (34.6%)	26,488 (32.8%)	0.039	8,866 (32.8%)	8,888 (32.9%)	0.002
Depression	17,120 (22.1%)	15,681 (19.4%)	0.066	5,477 (20.3%)	5,485 (20.3%)	0.001
Dementia	2,810 (3.6%)	8,041 (10.0%)	0.254	1,475 (5.5%)	1,478 (5.5%)	0.000
Delirium or psychosis	937 (1.2%)	1,956 (2.4%)	0.091	391 (1.4%)	397 (1.5%)	0.002
Anxiety	16,527 (21.3%)	12,670 (15.7%)	0.145	4,711 (17.4%)	4,667 (17.3%)	0.004
Sleep disorders	28,710 (37.0%)	27,520 (34.1%)	0.062	9,344 (34.6%)	9,355 (34.6%)	0.001
Anemia	16,045 (20.7%)	22,000 (27.2%)	0.154	6,159 (22.8%)	6,208 (23.0%)	0.004
Influenza	1,358 (1.8%)	1,860 (2.3%)	0.039	439 (1.6%)	479 (1.8%)	0.011
COVID	7,783 (10.0%)	2,458 (3.0%)	0.286	1,721 (6.4%)	1,703 (6.3%)	0.003
Hyperthyroidism and other thyroid gland disorders	22,026 (28.4%)	22,077 (27.3%)	0.024	7,452 (27.6%)	7,482 (27.7%)	0.002
Hypothyroidism	17,838 (23.0%)	18,173 (22.5%)	0.012	6,067 (22.4%)	6,119 (22.6%)	0.005
Nephrotic syndrome	10 (0.0%)	7 (0.0%)	0.004	3 (0.0%)	2 (0.0%)	0.004
Urinary incontinence	4,775 (6.2%)	5,193 (6.4%)	0.011	1,623 (6.0%)	1,662 (6.1%)	0.006
Biliary disease	161 (0.2%)	300 (0.4%)	0.030	61 (0.2%)	61 (0.2%)	0.000
Pancreatitis	39 (0.1%)	62 (0.1%)	0.011	14 (0.1%)	16 (0.1%)	0.003
Bowel obstruction	128 (0.2%)	196 (0.2%)	0.017	45 (0.2%)	47 (0.2%)	0.002
Gastroparesis	539 (0.7%)	598 (0.7%)	0.005	209 (0.8%)	207 (0.8%)	0.001
Diabetes medications						
Number of antidiabetic drugs on CED, mean (SD)	2.01 ± 0.87	2.12 ± 0.81	0.124	2.15 ± 0.86	2.14 ± 0.83	0.012
Concomitant use or initiation of Metformin	37,397 (48.2%)	46,091 (57.1%)	0.178	14,828 (54.9%)	14,792 (54.7%)	0.003
Concomitant use or initiation of Insulins	12,181 (15.7%)	8,753 (10.8%)	0.144	4,350 (16.1%)	4,259 (15.8%)	0.009
Concomitant use or initiation of Sulfonylureas	13,095 (16.9%)	24,753 (30.6%)	0.327	6,677 (24.7%)	6,589 (24.4%)	0.008
Concomitant use or initiation of SGLT-2i	12,218 (15.8%)	6,125 (7.6%)	0.257	3,677 (13.6%)	3,652 (13.5%)	0.003
Concomitant use or initiation of Any other glucose-lowering drugs	3,836 (4.9%)	4,612 (5.7%)	0.034	1,515 (5.6%)	1,558 (5.8%)	0.007
Past use of Metformin	49,536 (63.9%)	53,814 (66.6%)	0.057	18,391 (68.0%)	18,323 (67.8%)	0.005
Past use of Insulins	17,130 (22.1%)	12,945 (16.0%)	0.155	6,138 (22.7%)	6,011 (22.2%)	0.011

Past use of Sulfonylureas	18,245 (23.5%)	31,996 (39.6%)	0.351	8,962 (33.2%)	8,921 (33.0%)	0.003
Past use of SGLT-2i	16,932 (21.8%)	9,350 (11.6%)	0.278	5,411 (20.0%)	5,319 (19.7%)	0.009
Past use of Any other glucose-lowering drugs	5,537 (7.1%)	6,927 (8.6%)	0.053	2,298 (8.5%)	2,338 (8.6%)	0.005
**Other medications**						
ACE / ARB	56,677 (73.1%)	60,722 (75.2%)	0.047	20,409 (75.5%)	20,431 (75.6%)	0.002
ARNI	1,933 (2.5%)	1,134 (1.4%)	0.079	492 (1.8%)	489 (1.8%)	0.001
Thiazides	27,533 (35.5%)	26,589 (32.9%)	0.055	9,423 (34.9%)	9,350 (34.6%)	0.006
Beta-blockers	42,054 (54.2%)	47,257 (58.5%)	0.086	15,063 (55.7%)	15,150 (56.0%)	0.006
Calcium channel blockers	27,385 (35.3%)	31,661 (39.2%)	0.080	10,089 (37.3%)	10,019 (37.1%)	0.005
Digoxin / Digitoxin	836 (1.1%)	2,109 (2.6%)	0.114	424 (1.6%)	441 (1.6%)	0.005
Loop diuretics	19,414 (25.0%)	22,172 (27.4%)	0.055	6,834 (25.3%)	6,805 (25.2%)	0.002
Other diuretics	9,155 (11.8%)	7,633 (9.4%)	0.077	2,768 (10.2%)	2,795 (10.3%)	0.003
Intravenous diuretics	1,363 (1.8%)	1,831 (2.3%)	0.036	479 (1.8%)	474 (1.8%)	0.001
Nitrates	8,072 (10.4%)	9,903 (12.3%)	0.058	2,992 (11.1%)	3,042 (11.3%)	0.006
Anti-arrhythmics	2,916 (3.8%)	3,389 (4.2%)	0.022	967 (3.6%)	1,003 (3.7%)	0.007
Statins	62,706 (80.9%)	65,184 (80.7%)	0.004	22,204 (82.1%)	22,123 (81.8%)	0.008
PCSK9 inhibitors and other lipid-lowering drugs	12,142 (15.7%)	11,096 (13.7%)	0.054	4,032 (14.9%)	4,042 (15.0%)	0.001
Antiplatelet medications	12,023 (15.5%)	14,153 (17.5%)	0.054	4,313 (16.0%)	4,474 (16.6%)	0.016
Oral anticoagulants	11,614 (15.0%)	13,908 (17.2%)	0.061	4,115 (15.2%)	4,198 (15.5%)	0.009
COPD / Asthma medications	30,324 (39.1%)	27,815 (34.4%)	0.097	9,803 (36.3%)	9,829 (36.4%)	0.002
NSAIDS	26,177 (33.8%)	23,041 (28.5%)	0.113	8,355 (30.9%)	8,500 (31.4%)	0.012
Oral corticosteroids	21,850 (28.2%)	18,078 (22.4%)	0.134	6,462 (23.9%)	6,514 (24.1%)	0.005
Osteoporosis agents (incl. bisphosphonates)	2,339 (3.0%)	3,539 (4.4%)	0.072	935 (3.5%)	942 (3.5%)	0.001
Opioids	27,068 (34.9%)	26,509 (32.8%)	0.044	9,011 (33.3%)	8,955 (33.1%)	0.004
Anti-depressants	32,370 (41.7%)	27,437 (34.0%)	0.161	9,998 (37.0%)	9,998 (37.0%)	0.000
Antipsychotics	3,348 (4.3%)	3,480 (4.3%)	0.000	1,044 (3.9%)	1,047 (3.9%)	0.001
Anxiolytics / hypnotics, benzos	18,132 (23.4%)	16,098 (19.9%)	0.084	5,518 (20.4%)	5,604 (20.7%)	0.008
Dementia medications	1,237 (1.6%)	4,408 (5.5%)	0.211	751 (2.8%)	777 (2.9%)	0.006
Urinary tract infections antibiotics	38,526 (49.7%)	40,217 (49.8%)	0.002	13,228 (48.9%)	13,255 (49.0%)	0.002
Laxatives	2,836 (3.7%)	3,559 (4.4%)	0.038	895 (3.3%)	941 (3.5%)	0.009
**Healthcare utilization marker**						
Number of distinct medications; mean (SD)	15.38 ± 6.73	14.42 ± 6.50	0.145	14.94 ± 6.41	14.98 ± 6.68	0.004
Number of office visits; mean (SD)	10.95 ± 7.24	10.70 ± 7.39	0.034	10.83 ± 7.04	10.80 ± 7.48	0.003
Number of endocrinologist visits; mean (SD)	0.44 ± 1.35	0.34 ± 1.21	0.079	0.44 ± 1.25	0.43 ± 1.47	0.011
Number of cardiologist visits; mean (SD)	2.16 ± 3.77	2.32 ± 3.87	0.033	2.12 ± 3.69	2.12 ± 3.75	0.001
Number of internal/family medicine visits; mean (SD)	7.26 ± 7.85	7.89 ± 8.29	0.078	7.49 ± 8.02	7.42 ± 7.99	0.009
Number of electrocardiograms (ECG/EKG); mean (SD)	1.27 ± 1.80	1.55 ± 2.23	0.14	1.31 ± 1.86	1.32 ± 1.90	0.004
Number of echocardiograms; mean (SD)	0.41 ± 0.75	0.48 ± 0.82	0.073	0.42 ± 0.75	0.42 ± 0.76	0
Out-of-pocket medication cost; mean (SD)	634.54 ± 864.56	572.90 ± 725.43	0.075	628.89 ± 718.29	630.37 ± 837.86	0.002

Unique brand medicines; mean (SD)	15.63 ± 6.92	14.68 ± 6.71	0.139	15.22 ± 6.61	15.25 ± 6.89	0.004
Unique generic medicines; mean (SD)	15.38 ± 6.73	14.42 ± 6.50	0.145	14.94 ± 6.41	14.98 ± 6.68	0.004
Ratio of brand to generic medications; mean (SD)	1.01 ± 0.03	1.02 ± 0.04	0.267	1.01 ± 0.04	1.01 ± 0.04	0.000
**Healthy behavior markers**						
Colonoscopy / Sigmoidoscopy	9,052 (11.7%)	7,822 (9.7%)	0.064	2,859 (10.6%)	2,843 (10.5%)	0.002
Flu Pneumococcal vaccine	24,541 (31.7%)	20,396 (25.3%)	0.142	7,932 (29.3%)	7,936 (29.4%)	0.000
Pap smear	4,839 (6.2%)	3,066 (3.8%)	0.112	1,345 (5.0%)	1,315 (4.9%)	0.005
PSA test	18,881 (24.4%)	17,476 (21.6%)	0.065	6,498 (24.0%)	6,458 (23.9%)	0.003
Fecal occult blood test	3,742 (4.8%)	6,274 (7.8%)	0.121	1,727 (6.4%)	1,689 (6.2%)	0.006
Bone mineral density tests	6,626 (8.5%)	6,323 (7.8%)	0.026	2,190 (8.1%)	2,197 (8.1%)	0.001
Mammograms	21,441 (27.7%)	15,842 (19.6%)	0.190	6,463 (23.9%)	6,425 (23.8%)	0.003
Telemedicine	20,233 (26.1%)	10,117 (12.5%)	0.349	5,911 (21.9%)	5,752 (21.3%)	0.014
**Laboratory and diagnostic tests**						
HbA1c tests; mean (SD)	2.38 ± 1.47	2.50 ± 2.09	0.071	2.52 ± 1.63	2.51 ± 1.87	0.008
Lipid panels; mean (SD)	1.70 ± 1.28	1.75 ± 1.82	0.036	1.76 ± 1.39	1.75 ± 1.51	0.009
Creatinine tests; mean (SD)	2.06 ± 2.63	1.26 ± 2.66	0.296	1.70 ± 2.63	1.70 ± 2.43	0.001
Natriuretic peptide tests; mean (SD)	0.25 ± 0.99	0.32 ± 2.04	0.034	0.25 ± 1.10	0.25 ± 0.94	0.004
Urine tests; mean (SD)	1.16 ± 1.86	1.49 ± 2.81	0.141	1.30 ± 2.19	1.30 ± 1.99	0.002
**Lab values**						
HbA1c (%)*; mean (SD) median [IQR]	7.44 ±1.62 7.00 [6.30, 8.20]	7.93 ±1.59 7.70 [6.80, 8.70]	0.305	7.73 ±1.70 7.40 [6.50, 8.60]	7.94 ±1.58 7.70 [6.90, 8.70]	0.128
Glucose (mg/dl)*; mean (SD) median [IQR]	151.56 ±65.11 135.00 [109.67, 174.00]	169.58 ±67.47 155.00 [125.00, 196.50]	0.272	161.42 ±69.55 145.00 [115.00, 189.00]	168.05 ±67.37 153.50 [124.00, 195.00]	0.097
Creatinine (mg/dl)*; mean (SD) median [IQR]	1.04 ±0.51 0.94 [0.80, 1.14]	1.10 ±0.39 0.99 [0.81, 1.24]	0.133	1.08 ±0.76 0.97 [0.80, 1.19]	1.08 ±0.39 0.97 [0.80, 1.20]	0.01
Systolic blood pressure (mmHg)*; mean (SD) median [IQR]	129.54 ±20.15 130.00 [120.00, 139.00]	130.92 ±18.99 130.00 [120.00, 140.00]	0.071	130.32 ±18.82 130.00 [120.00, 140.00]	130.55 ±19.44 130.00 [120.00, 140.00]	0.012
Heart rate (1/min)*; mean (SD) median [IQR]	77.43 ±74.35 76.00 [68.00, 85.00]	76.98 ±13.25 76.00 [68.00, 85.00]	0.008	79.08 ±156.71 76.00 [68.00, 85.00]	77.03 ±13.13 76.00 [68.00, 85.00]	0.018
BMI (kg/m2)*; mean (SD) median [IQR]	37.54 ±7.79 36.50 [32.23, 41.61]	32.37 ±7.33 31.44 [27.40, 36.30]	0.684	35.12 ±7.56 34.05 [29.95, 39.06]	33.56 ±7.25 32.56 [28.69, 37.40]	0.21
eGFR (ml/min/1.73m2)*; mean (SD) median [IQR]	2,877.13 ±163,592.21 74.00 [57.00, 91.00]	2,420.31 ±153,391.95 68.00 [51.00, 86.00]	0.003	5,607.26 ±235,308.75 69.00 [54.00, 87.00]	2,831.44 ±166,227.44 69.00 [52.00, 87.00]	0.014
LDL (mg/dl)*; mean (SD) median [IQR]	82.19 ±37.24 77.00 [59.00, 102.00]	81.59 ±36.40 77.00 [58.00, 101.00]	0.016	80.29 ±36.35 76.00 [57.30, 100.00]	81.57 ±36.53 77.00 [59.00, 101.00]	0.035
HDL (mg/dl)*; mean (SD) median [IQR]	47.15 ±14.66 46.00 [38.00, 55.00]	46.91 ±13.99 45.00 [38.00, 54.00]	0.016	47.13 ±14.53 45.20 [38.00, 55.00]	46.32 ±13.77 45.00 [37.00, 54.00]	0.057
Total cholesterol (mg/dl)*; mean (SD) median [IQR]	159.25 ±46.47 154.00 [131.00, 184.00]	160.18 ±44.79 155.00 [131.00, 183.00]	0.02	157.53 ±45.43 152.00 [129.00, 182.00]	158.96 ±46.09 154.00 [129.00, 183.00]	0.031
Triglyceride (mg/dl)*; mean (SD) median [IQR]	163.64 ±122.48 138.00 [100.00, 194.00]	163.65 ±102.89 140.00 [101.00, 197.75]	0	162.79 ±112.87 138.00 [99.00, 195.00]	165.52 ±103.44 140.00 [102.00, 200.00]	0.025
**Burden of comorbidities**						
Combined comorbidity score; mean (SD)	2.32 ± 2.33	2.93 ± 2.73	0.24	2.53 ± 2.45	2.53 ± 2.46	0.003
Frailty Score; mean (SD)	0.18 ± 0.05	0.21 ± 0.07	0.341	0.19 ± 0.06	0.19 ± 0.06	0
**Baseline hospitalizations and hospital metrics**						
Number of Hospitalizations; mean (SD)	2.00 ± 13.68	2.00 ± 13.23	0	4.08 ± 20.33	3.93 ± 18.34	0.003

Any hospitalization within prior 91 days	2,904 (3.7%)	8,570 (10.6%)	0.268	1,528 (5.7%)	1,558 (5.8%)	0.005
Any hospitalization within prior 92-365 days	10,105 (13.0%)	15,571 (19.3%)	0.170	3,964 (14.7%)	3,998 (14.8%)	0.004
Number of hospitalizations (0, 1, 2 or more)						
< 1	65,652 (84.7%)	60,157 (74.5%)	0.255	22,113 (81.8%)	22,078 (81.7%)	0.003
1 - <2	6,833 (8.8%)	11,258 (13.9%)	0.162	2,753 (10.2%)	2,804 (10.4%)	0.006
>= 2	5,051 (6.5%)	9,359 (11.6%)	0.177	2,167 (8.0%)	2,151 (8.0%)	0.002
Heart failure hospitalization	3,595 (4.6%)	7,367 (9.1%)	0.178	1,580 (5.8%)	1,588 (5.9%)	0.001
ED visit	27,294 (35.2%)	36,508 (45.2%)	0.205	10,464 (38.7%)	10,407 (38.5%)	0.004
Calendar year of cohort entry						
2018	1,001 (1.3%)	21,855 (27.1%)	0.795	985 (3.6%)	985 (3.6%)	0.000
2019	5,805 (7.5%)	21,786 (27.0%)	0.534	5,067 (18.7%)	5,109 (18.9%)	0.004
2020	8,725 (11.3%)	18,179 (22.5%)	0.304	6,774 (25.1%)	6,697 (24.8%)	0.007
2021	7,066 (9.1%)	6,358 (7.9%)	0.045	3,824 (14.1%)	3,705 (13.7%)	0.013
2022	12,045 (15.5%)	4,987 (6.2%)	0.304	3,772 (14.0%)	3,773 (14.0%)	0.000
2023	24,521 (31.6%)	4,439 (5.5%)	0.714	3,834 (14.2%)	3,936 (14.6%)	0.011
2024	18,081 (23.3%)	3,159 (3.9%)	0.590	2,766 (10.2%)	2,817 (10.4%)	0.006
2025	292 (0.4%)	11 (0.0%)	0.082	11 (0.0%)	11 (0.0%)	0.000

Abbreviations: *not used in the propensity score; ACE, angiotensin-converting enzyme inhibitors; ARB, angiotensin receptor blocker; ARNI, angiotensin receptor/neprilysin inhibitor; BMI, body mass index; CABG, coronary artery bypass graft surgery; CED, cohort entry date; CKD, chronic kidney disease; COPD, chronic obstructive pulmonary disease; DKA, diabetic ketoacidosis; DPP4i, dipeptidyl peptidase-4 inhibitors; ED, emergency department; eGFR, estimated glomerular filtration rate (estimated using the quadratic GFR equation: GFR = EXP(1.911 + (5.249 / Serum creatinine) - (2.114 / (Serum creatinine^2)) - (0.00686 * Age) - 0.205 (if female))); HbA1c, hemoglobin A1c; HDL, high-density lipoprotein cholesterol; HONK, hyperglycemic hyperosmolar nonketotic state; IQR, inter-quartile-range; MASH, metabolic dysfunction associated steatohepatitis; MASLD, metabolic dysfunction associated steatotic liver disease; MI, myocardial infarction; N, number of participants; NSAIDs, non-steroidal anti-inflammatory drugs; PCSK9, proprotein convertase subtilisin/kexin type 9; PSA, prostate-specific antigen; PTCA, percutaneous transluminal coronary angioplasty; SGLT2i, sodium-glucose transport protein 2 inhibitors; SD, standard deviation; SMD, standardized mean difference; TIA, transient ischemic attack.
Missing data were handled by assuming absence of a code indicated absence of the condition for most binary covariates. Missing indicators were included for race and region in the propensity score model.
Laboratory values were only available in a subset of the Optum database and were truncated using clinically plausible cut-off values (BMI values >100 or <10 were set to missing; missingness before matching ~58%. Creatinine values >30 were set to missing, and values <0.8 were set to 0.8; missingness before matching ~39%. eGFR values >150 were set to 150, and values =0 were set to missing; missingness before matching ~69%. Glucose values <30 were set to missing; missingness before matching ~39%. HbA1c values ≥20 or ≤2 were set to missing; missingness before matching ~34%. HDL value missingness before matching was ~47%. Heart rate values <30 were set to missing; missingness before matching ~71%. LDL value missingness before matching was ~44%. Systolic blood pressure values <30 were set to missing; missingness before matching ~56%. Total cholesterol value missingness before matching was ~47%. Triglyceride value missingness before matching was ~48%.).
Dual status/Low income subsidy was only available in Medicare database. Race was reported by the respective database: Medicare and Optum. The Race-Others category includes Asian and Hispanic individuals.

Supplementary Table 3. Baseline characteristics of initiators of semaglutide vs sitagliptin when applying trial eligibility criteria before and after propensity score matching, Optum database. Values are number (percentage) unless otherwise specified.

	Before propensity score matching			After propensity score matching		
Variable	Semaglutide (n = 49,843)	Sitagliptin (n = 28,279)	SMD	Semaglutide (n = 13,602)	Sitagliptin (n = 13,602)	SMD
Demographics						
Age; mean (SD) median [IQR]	67.49 ±7.76 68.00 [62.00, 73.00]	72.34 ±7.50 72.00 [68.00, 78.00]	0.636	70.57 ±7.43 71.00 [66.00, 76.00]	70.56 ±7.47 71.00 [67.00, 75.00]	0.001
Gender						
Male	21,624 (43.4%)	12,564 (44.4%)	0.021	6,005 (44.1%)	5,991 (44.0%)	0.002
Female	28,219 (56,6%)	15,715 (55,6%)	0.021	7,597 (55,9%)	7,611 (56,0%)	0.002
Race						
White	24,640 (49.4%)	12,998 (46.0%)	0.070	6,583 (48.4%)	6,563 (48.3%)	0.003
Black	6,869 (13.8%)	4,762 (16.8%)	0.085	2,152 (15.8%)	2,143 (15.8%)	0.002
Unknown / Missing	17,616 (35.3%)	9,385 (33.2%)	0.045	4,553 (33.5%)	4,585 (33.7%)	0.005
Others	718 (1.4%)	1,134 (4.0%)	0.158	314 (2.3%)	311 (2.3%)	0.001
Region / State						
Northeast	4,487 (9.0%)	2,992 (10.6%)	0.053	1,359 (10.0%)	1,409 (10.4%)	0.012
Midwest / North central	9,445 (18.9%)	3,803 (13.4%)	0.150	2,185 (16.1%)	2,162 (15.9%)	0.005
South	27,747 (55.7%)	16,289 (57.6%)	0.039	7,811 (57.4%)	7,790 (57.3%)	0.003
West	8,143 (16.3%)	5,182 (18.3%)	0.053	2,240 (16.5%)	2,238 (16.5%)	0.000
Missing	21 (0.0%)	13 (0.0%)	0.002	7 (0.1%)	3 (0.0%)	0.015
Lifestyle risk factors						
Smoking/Tobacco use	13,895 (27.9%)	7,220 (25.5%)	0.053	3,501 (25.7%)	3,512 (25.8%)	0.002
Weight						
Underweight	63 (0.1%)	388 (1.4%)	0.145	56 (0.4%)	52 (0.4%)	0.005
Normal weight	724 (1.5%)	2,662 (9.4%)	0.357	553 (4.1%)	570 (4.2%)	0.006
Overweight (BMI 25.0-29.9)	5,293 (10.6%)	6,852 (24.2%)	0.365	2,646 (19.5%)	2,612 (19.2%)	0.006
Class 1 Obesity (BMI 30.0-34.9)	5,185 (10.4%)	3,959 (14.0%)	0.11	1,844 (13.6%)	1,848 (13.6%)	0.001
Class 2 Obesity (BMI 35.0-39.9)	3,415 (6.9%)	1,703 (6.0%)	0.034	928 (6.8%)	892 (6.6%)	0.011
Class 3 Obesity; (BMI 40.0 and above)	19,481 (39.1%)	5,460 (19.3%)	0.446	3,554 (26.1%)	3,557 (26.2%)	0.001
Unspecified Obesity	15,682 (31.5%)	7,255 (25.7%)	0.129	4,021 (29.6%)	4,071 (29.9%)	0.008
Diabetes complications						
Diabetic retinopathy	4,047 (8.1%)	3,077 (10.9%)	0.094	1,393 (10.2%)	1,335 (9.8%)	0.014
Diabetic neuropathy	14,480 (29.1%)	9,795 (34.6%)	0.120	4,536 (33.3%)	4,474 (32.9%)	0.010
Diabetic nephropathy	12,927 (25.9%)	10,091 (35.7%)	0.212	4,296 (31.6%)	4,260 (31.3%)	0.006
Diabetes with other ophthalmic complications	2,382 (4.8%)	1,862 (6.6%)	0.078	797 (5.9%)	805 (5.9%)	0.002
Diabetes with peripheral circulatory disorders	8,322 (16.7%)	5,796 (20.5%)	0.098	2,607 (19.2%)	2,579 (19.0%)	0.005
Diabetic foot	1,434 (2.9%)	898 (3.2%)	0.017	439 (3.2%)	399 (2.9%)	0.017

Erectile dysfunction	2,471 (5.0%)	1,064 (3.8%)	0.059	578 (4.2%)	577 (4.2%)	0.000
Hypoglycemia	12,276 (24.6%)	7,495 (26.5%)	0.043	3,607 (26.5%)	3,521 (25.9%)	0.014
Hyperglycemia/DKA/HONK	23,311 (46.8%)	14,525 (51.4%)	0.092	7,018 (51.6%)	6,883 (50.6%)	0.02
Skin infections	5,344 (10.7%)	2,841 (10.0%)	0.022	1,381 (10.2%)	1,368 (10.1%)	0.003
**Cardiovascular-related conditions**						
Coronary atherosclerosis	18,491 (37.1%)	10,186 (36.0%)	0.022	4,844 (35.6%)	4,878 (35.9%)	0.005
Stable angina	3,737 (7.5%)	1,990 (7.0%)	0.018	966 (7.1%)	975 (7.2%)	0.003
Unstable angina	1,795 (3.6%)	1,001 (3.5%)	0.003	443 (3.3%)	465 (3.4%)	0.009
Hypertension	46,038 (92.4%)	26,616 (94.1%)	0.07	12,714 (93.5%)	12,721 (93.5%)	0.002
Hypotension	1,864 (3.7%)	1,411 (5.0%)	0.061	593 (4.4%)	568 (4.2%)	0.009
Hyperlipidemia	43,970 (88.2%)	25,165 (89.0%)	0.024	12,150 (89.3%)	12,081 (88.8%)	0.016
Acute MI	544 (1.1%)	352 (1.2%)	0.014	146 (1.1%)	161 (1.2%)	0.010
Old MI	3,370 (6.8%)	2,207 (7.8%)	0.040	930 (6.8%)	985 (7.2%)	0.016
Ischemic stroke	354 (0.7%)	264 (0.9%)	0.025	114 (0.8%)	110 (0.8%)	0.003
TIA	1,354 (2.7%)	982 (3.5%)	0.044	392 (2.9%)	394 (2.9%)	0.001
Cardiac conduction disorder	4,036 (8.1%)	2,488 (8.8%)	0.025	1,140 (8.4%)	1,121 (8.2%)	0.005
Previous cardiac procedure (CABG, PTCA, Stent)	1,178 (2.4%)	614 (2.2%)	0.013	279 (2.1%)	308 (2.3%)	0.015
PVD diagnosis or surgery	6,364 (12.8%)	4,767 (16.9%)	0.115	2,031 (14.9%)	2,002 (14.7%)	0.006
Atrial fibrillation	7,576 (15.2%)	4,414 (15.6%)	0.011	2,008 (14.8%)	2,087 (15.3%)	0.016
Other cardiac dysrhythmia	13,141 (26.4%)	7,334 (25.9%)	0.01	3,463 (25.5%)	3,459 (25.4%)	0.001
Heart failure	10,804 (21.7%)	6,093 (21.5%)	0.003	2,785 (20.5%)	2,837 (20.9%)	0.009
Acute heart failure	2,170 (4.4%)	1,397 (4.9%)	0.028	570 (4.2%)	612 (4.5%)	0.015
Cardiomyopathy	3,880 (7.8%)	2,291 (8.1%)	0.012	1,005 (7.4%)	1,046 (7.7%)	0.011
Valve disorders	7,987 (16.0%)	4,810 (17.0%)	0.027	2,180 (16.0%)	2,179 (16.0%)	0.000
Valve replacement	940 (1.9%)	585 (2.1%)	0.013	286 (2.1%)	274 (2.0%)	0.006
Edema	8,407 (16.9%)	4,621 (16.3%)	0.014	2,200 (16.2%)	2,246 (16.5%)	0.009
Venous thromboembolism / Pulmonary embolism	1,998 (4.0%)	1,111 (3.9%)	0.004	552 (4.1%)	551 (4.1%)	0.000
Pulmonary hypertension	2,152 (4.3%)	1,292 (4.6%)	0.012	566 (4.2%)	579 (4.3%)	0.005
Implantable cardioverter defibrillator	186 (0.4%)	128 (0.5%)	0.012	50 (0.4%)	55 (0.4%)	0.006
Hyperkalemia	1,585 (3.2%)	1,407 (5.0%)	0.091	579 (4.3%)	577 (4.2%)	0.001
Cerebrovascular procedure	71 (0.1%)	39 (0.1%)	0.001	21 (0.2%)	18 (0.1%)	0.006
Insertion of pacemakers / removal of cardiac lead	117 (0.2%)	101 (0.4%)	0.023	42 (0.3%)	39 (0.3%)	0.004
**Renal-related conditions**						
Hypertensive nephropathy	8,492 (17.0%)	6,296 (22.3%)	0.132	2,710 (19.9%)	2,713 (19.9%)	0.001
CKD Stage 1-2	3,068 (6.2%)	2,511 (8.9%)	0.103	1,010 (7.4%)	1,031 (7.6%)	0.006
CKD Stage 3-4	11,499 (23.1%)	8,496 (30.0%)	0.158	3,704 (27.2%)	3,685 (27.1%)	0.003
Unspecified CKD	3,524 (7.1%)	2,778 (9.8%)	0.099	1,152 (8.5%)	1,164 (8.6%)	0.003
Microalbuminuria or proteinuria	1,194 (2.4%)	771 (2.7%)	0.021	390 (2.9%)	395 (2.9%)	0.002
Acute kidney injury	3,362 (6.7%)	2,911 (10.3%)	0.127	1,178 (8.7%)	1,197 (8.8%)	0.005

Urinary tract infections	7,303 (14.7%)	5,313 (18.8%)	0.111	2,230 (16.4%)	2,280 (16.8%)	0.010
Genital infections	1,420 (2.8%)	944 (3.3%)	0.028	475 (3.5%)	458 (3.4%)	0.007
Urolithiasis (Kidney and urinary stone)	2,620 (5.3%)	1,374 (4.9%)	0.018	702 (5.2%)	685 (5.0%)	0.006
Other comorbidities						
COPD	8,574 (17.2%)	5,055 (17.9%)	0.018	2,373 (17.4%)	2,337 (17.2%)	0.007
Asthma	7,069 (14.2%)	3,032 (10.7%)	0.105	1,681 (12.4%)	1,644 (12.1%)	0.008
Obstructive sleep apnea	18,902 (37.9%)	5,849 (20.7%)	0.386	3,697 (27.2%)	3,657 (26.9%)	0.007
Serious bacterial infections	1,402 (2.8%)	1,292 (4.6%)	0.093	465 (3.4%)	474 (3.5%)	0.004
Pneumonia	2,842 (5.7%)	1,984 (7.0%)	0.054	831 (6.1%)	873 (6.4%)	0.013
Liver disease	9,167 (18.4%)	4,797 (17.0%)	0.037	2,381 (17.5%)	2,469 (18.2%)	0.017
MASH / MASLD	4,967 (10.0%)	1,928 (6.8%)	0.114	1,117 (8.2%)	1,149 (8.4%)	0.009
Fractures / Falls	3,511 (7.0%)	2,595 (9.2%)	0.078	1,075 (7.9%)	1,076 (7.9%)	0.000
Osteoporosis	2,895 (5.8%)	2,272 (8.0%)	0.088	918 (6.7%)	923 (6.8%)	0.001
Osteoarthritis	18,670 (37.5%)	9,682 (34.2%)	0.067	4,780 (35.1%)	4,798 (35.3%)	0.003
Depression	12,110 (24.3%)	6,237 (22.1%)	0.053	3,076 (22.6%)	3,064 (22.5%)	0.002
Dementia	2,026 (4.1%)	2,753 (9.7%)	0.225	920 (6.8%)	904 (6.6%)	0.005
Delirium or psychosis	724 (1.5%)	636 (2.2%)	0.059	250 (1.8%)	238 (1.7%)	0.007
Anxiety	11,468 (23.0%)	4,857 (17.2%)	0.146	2,589 (19.0%)	2,573 (18.9%)	0.003
Sleep disorders	20,010 (40.1%)	10,376 (36.7%)	0.071	5,089 (37.4%)	5,059 (37.2%)	0.005
Anemia	10,534 (21.1%)	7,339 (26.0%)	0.114	3,192 (23.5%)	3,229 (23.7%)	0.006
Influenza	861 (1.7%)	493 (1.7%)	0.001	183 (1.3%)	212 (1.6%)	0.018
COVID	5,237 (10.5%)	1,592 (5.6%)	0.180	1,154 (8.5%)	1,136 (8.4%)	0.005
Hyperthyroidism and other thyroid gland disorders	14,713 (29.5%)	8,014 (28.3%)	0.026	3,864 (28.4%)	3,912 (28.8%)	0.008
Hypothyroidism	12,013 (24.1%)	6,582 (23.3%)	0.019	3,183 (23.4%)	3,201 (23.5%)	0.003
Nephrotic syndrome	5 (0.0%)	4 (0.0%)	0.004	3 (0.0%)	2 (0.0%)	0.005
Urinary incontinence	3,556 (7.1%)	2,040 (7.2%)	0.003	982 (7.2%)	975 (7.2%)	0.002
Biliary disease	97 (0.2%)	69 (0.2%)	0.011	29 (0.2%)	29 (0.2%)	0.000
Pancreatitis	23 (0.0%)	12 (0.0%)	0.002	6 (0.0%)	6 (0.0%)	0.000
Bowel obstruction	69 (0.1%)	53 (0.2%)	0.012	19 (0.1%)	23 (0.2%)	0.007
Gastroparesis	367 (0.7%)	242 (0.9%)	0.013	121 (0.9%)	114 (0.8%)	0.006
Diabetes medications						
Number of antidiabetic drugs on CED; mean (SD) median [IQR]	1.98 ±0.86 2.00 [1.00, 3.00]	2.14 ±0.81 2.00 [2.00, 3.00]	0.193	2.14 ±0.86 2.00 [2.00, 3.00]	2.12 ±0.82 2.00 [2.00, 3.00]	0.020
Concomitant use or initiation of Metformin	23,643 (47.4%)	16,358 (57.8%)	0.210	7,515 (55.2%)	7,470 (54.9%)	0.007
Concomitant use or initiation of Insulins	6,497 (13.0%)	2,690 (9.5%)	0.112	1,640 (12.1%)	1,554 (11.4%)	0.020
Concomitant use or initiation of Sulfonylureas	8,551 (17.2%)	8,769 (31.0%)	0.328	3,600 (26.5%)	3,495 (25.7%)	0.018
Concomitant use or initiation of SGLT-2i	7,609 (15.3%)	2,722 (9.6%)	0.171	1,905 (14.0%)	1,885 (13.9%)	0.004
Concomitant use or initiation of Any other glucose-lowering drugs	2,685 (5.4%)	1,813 (6.4%)	0.043	813 (6.0%)	843 (6.2%)	0.009
Past use of Metformin	31,269 (62.7%)	19,078 (67.5%)	0.099	9,199 (67.6%)	9,130 (67.1%)	0.011
Past use of Insulins	9,352 (18.8%)	3,977 (14.1%)	0.127	2,336 (17.2%)	2,247 (16.5%)	0.017

Past use of Sulfonylureas	11,844 (23.8%)	11,407 (40.3%)	0.361	4,780 (35.1%)	4,690 (34.5%)	0.014
Past use of SGLT-2i	10,710 (21.5%)	4,150 (14.7%)	0.178	2,864 (21.1%)	2,783 (20.5%)	0.015
Past use of Any other glucose-lowering drugs	3,761 (7.5%)	2,678 (9.5%)	0.069	1,221 (9.0%)	1,230 (9.0%)	0.002
Other medications						
ACE / ARB	36,407 (73.0%)	21,648 (76.6%)	0.081	10,250 (75.4%)	10,263 (75.5%)	0.002
ARNI	1,308 (2.6%)	428 (1.5%)	0.078	259 (1.9%)	257 (1.9%)	0.001
Thiazides	17,664 (35.4%)	9,623 (34.0%)	0.030	4,719 (34.7%)	4,714 (34.7%)	0.001
Beta-blockers	26,670 (53.5%)	15,520 (54.9%)	0.028	7,310 (53.7%)	7,363 (54.1%)	0.008
Calcium channel blockers	17,766 (35.6%)	11,079 (39.2%)	0.073	5,149 (37.9%)	5,141 (37.8%)	0.001
Digoxin / Digitoxin	464 (0.9%)	516 (1.8%)	0.077	177 (1.3%)	198 (1.5%)	0.013
Loop diuretics	12,730 (25.5%)	6,593 (23.3%)	0.052	3,214 (23.6%)	3,224 (23.7%)	0.002
Other diuretics	5,875 (11.8%)	2,393 (8.5%)	0.11	1,295 (9.5%)	1,297 (9.5%)	0.001
Intravenous diuretics	910 (1.8%)	514 (1.8%)	0.001	216 (1.6%)	217 (1.6%)	0.001
Nitrates	4,979 (10.0%)	2,841 (10.0%)	0.002	1,314 (9.7%)	1,332 (9.8%)	0.004
Anti-arrhythmics	1,885 (3.8%)	933 (3.3%)	0.026	450 (3.3%)	477 (3.5%)	0.011
Statins	40,844 (81.9%)	23,398 (82.7%)	0.021	11,337 (83.3%)	11,337 (83.3%)	0.000
PCSK9 inhibitors and other lipid-lowering drugs	7,683 (15.4%)	3,633 (12.8%)	0.074	1,967 (14.5%)	1,951 (14.3%)	0.003
Antiplatelet medications	6,992 (14.0%)	4,106 (14.5%)	0.014	1,808 (13.3%)	1,906 (14.0%)	0.021
Oral anticoagulants	7,548 (15.1%)	3,966 (14.0%)	0.032	1,925 (14.2%)	1,985 (14.6%)	0.013
COPD / Asthma medications	19,893 (39.9%)	9,683 (34.2%)	0.118	5,022 (36.9%)	4,983 (36.6%)	0.006
NSAIDS	16,906 (33.9%)	8,230 (29.1%)	0.104	4,261 (31.3%)	4,321 (31.8%)	0.009
Oral corticosteroids	14,125 (28.3%)	5,976 (21.1%)	0.168	3,152 (23.2%)	3,202 (23.5%)	0.009
Osteoporosis agents (incl. bisphosphonates)	1,676 (3.4%)	1,379 (4.9%)	0.076	536 (3.9%)	542 (4.0%)	0.002
Opioids	17,708 (35.5%)	8,809 (31.2%)	0.093	4,400 (32.3%)	4,334 (31.9%)	0.010
Anti-depressants	21,362 (42.9%)	9,311 (32.9%)	0.206	4,959 (36.5%)	4,950 (36.4%)	0.001
Antipsychotics	2,421 (4.9%)	1,193 (4.2%)	0.031	587 (4.3%)	590 (4.3%)	0.001
Anxiolytics / hypnotics, benzos	11,876 (23.8%)	5,399 (19.1%)	0.116	2,694 (19.8%)	2,733 (20.1%)	0.007
Dementia medications	819 (1.6%)	1,396 (4.9%)	0.185	426 (3.1%)	433 (3.2%)	0.003
Urinary tract infections antibiotics	24,575 (49.3%)	13,197 (46.7%)	0.053	6,456 (47.5%)	6,436 (47.3%)	0.003
Laxatives	1,870 (3.8%)	1,053 (3.7%)	0.001	452 (3.3%)	454 (3.3%)	0.001
Healthcare utilization marker						
Number of distinct medications; mean (SD) median [IQR]	15.47 ±6.76 15.00 [11.00, 19.00]	14.66 ±6.48 14.00 [10.00, 18.00]	0.122	15.05 ±6.46 14.00 [10.00, 19.00]	15.01 ±6.61 14.00 [10.00, 19.00]	0.006
Number of office visits; mean (SD) median [IQR]	10.81 ±7.12 9.00 [6.00, 14.00]	9.88 ±6.83 8.00 [5.00, 13.00]	0.133	10.22 ±6.63 9.00 [6.00, 13.00]	10.17 ±7.01 9.00 [5.00, 14.00]	0.007
Number of endocrinologist visits; mean (SD) median [IQR]	0.37 ±1.24 0.00 [0.00, 0.00]	0.30 ±1.20 0.00 [0.00, 0.00]	0.057	0.34 ±1.08 0.00 [0.00, 0.00]	0.33 ±1.34 0.00 [0.00, 0.00]	0.008
Number of cardiologist visits; mean (SD) median [IQR]	2.23 ±3.87 1.00 [0.00, 3.00]	2.24 ±3.96 1.00 [0.00, 3.00]	0.002	2.12 ±3.76 1.00 [0.00, 3.00]	2.16 ±3.95 1.00 [0.00, 3.00]	0.011
Number of internal/family medicine visits; mean (SD) median [IQR]	8.05 ±8.56 6.00 [3.00, 10.00]	9.21 ±9.36 7.00 [4.00, 12.00]	0.129	8.72 ±9.25 6.00 [3.00, 11.00]	8.63 ±9.11 6.00 [3.00, 11.00]	0.010
Number of electrocardiograms (ECG/EKG) ; mean (SD) median [IQR]	1.22 ±1.75 1.00 [0.00, 2.00]	1.30 ±1.90 1.00 [0.00, 2.00]	0.043	1.19 ±1.72 1.00 [0.00, 2.00]	1.23 ±1.87 1.00 [0.00, 2.00]	0.022
Number of echocardiograms; mean (SD) median [IQR]	0.39 ±0.71 0.00 [0.00, 1.00]	0.38 ±0.70 0.00 [0.00, 1.00]	0.009	0.37 ±0.69 0.00 [0.00, 1.00]	0.37 ±0.70 0.00 [0.00, 1.00]	0.009

Out-of-pocket medication cost; mean (SD) median [IQR]	606.72 ±828.66 366.06 [146.95, 800.31]	518.72 ±663.16 306.90 [125.23, 687.83]	0.117	546.68 ±605.66 345.01 [143.55, 743.16]	547.44 ±736.98 303.45 [127.15, 718.12]	0.001
Unique brand medicines; mean (SD) median [IQR]	15.71 ±6.95 15.00 [11.00, 20.00]	14.90 ±6.67 14.00 [10.00, 19.00]	0.118	15.30 ±6.65 14.00 [11.00, 19.00]	15.25 ±6.80 14.00 [10.00, 19.00]	0.007
Unique generic medicines; mean (SD) median [IQR]	15.47 ±6.76 15.00 [11.00, 19.00]	14.66 ±6.48 14.00 [10.00, 18.00]	0.122	15.05 ±6.46 14.00 [10.00, 19.00]	15.01 ±6.61 14.00 [10.00, 19.00]	0.006
Ratio of brand to generic medications; mean (SD) median [IQR]	1.01 ±0.03 1.00 [1.00, 1.00]	1.01 ±0.04 1.00 [1.00, 1.00]	0.027	1.01 ±0.04 1.00 [1.00, 1.00]	1.01 ±0.04 1.00 [1.00, 1.00]	0.010
Healthy behavior markers						
Colonoscopy / Sigmoidoscopy	5,709 (11.5%)	2,631 (9.3%)	0.071	1,402 (10.3%)	1,391 (10.2%)	0.003
Flu Pneumococcal vaccine	17,564 (35.2%)	11,130 (39.4%)	0.085	4,992 (36.7%)	5,013 (36.9%)	0.003
Pap smear	2,659 (5.3%)	937 (3.3%)	0.100	537 (3.9%)	530 (3.9%)	0.003
PSA test	11,948 (24.0%)	6,262 (22.1%)	0.043	3,112 (22.9%)	3,133 (23.0%)	0.004
Fecal occult blood test	2,370 (4.8%)	2,515 (8.9%)	0.165	920 (6.8%)	914 (6.7%)	0.002
Bone mineral density tests	4,838 (9.7%)	2,550 (9.0%)	0.024	1,240 (9.1%)	1,246 (9.2%)	0.002
Mammograms	14,310 (28.7%)	6,299 (22.3%)	0.148	3,454 (25.4%)	3,420 (25.1%)	0.006
Telemedicine	12,448 (25.0%)	4,742 (16.8%)	0.203	3,147 (23.1%)	3,070 (22.6%)	0.013
Laboratory and diagnostic tests						
HbA1c tests; mean (SD) median [IQR]	2.36 ±1.32 2.00 [1.00, 3.00]	2.45 ±1.30 2.00 [2.00, 3.00]	0.068	2.47 ±1.36 2.00 [2.00, 3.00]	2.45 ±1.28 2.00 [2.00, 3.00]	0.011
Lipid panels; mean (SD) median [IQR]	1.69 ±1.19 2.00 [1.00, 2.00]	1.74 ±1.21 2.00 [1.00, 2.00]	0.044	1.73 ±1.22 2.00 [1.00, 2.00]	1.72 ±1.18 2.00 [1.00, 2.00]	0.009
Creatinine tests; mean (SD) median [IQR]	3.17 ±2.69 3.00 [2.00, 4.00]	3.40 ±3.50 3.00 [2.00, 4.00]	0.073	3.30 ±2.88 3.00 [2.00, 4.00]	3.30 ±2.52 3.00 [2.00, 4.00]	0.000
Natriuretic peptide tests; mean (SD) median [IQR]	0.24 ±0.81 0.00 [0.00, 0.00]	0.23 ±2.29 0.00 [0.00, 0.00]	0.005	0.21 ±0.69 0.00 [0.00, 0.00]	0.21 ±0.70 0.00 [0.00, 0.00]	0.002
Urine tests; mean (SD) median [IQR]	1.12 ±1.66 1.00 [0.00, 2.00]	1.32 ±2.82 1.00 [0.00, 2.00]	0.088	1.23 ±1.80 1.00 [0.00, 2.00]	1.22 ±1.73 1.00 [0.00, 2.00]	0.007
Lab values						
HbA1c (%)*; mean (SD) median [IQR]	7.44 ±1.62 7.00 [6.30, 8.20]	7.93 ±1.59 7.70 [6.80, 8.70]	0.305	7.73 ±1.70 7.40 [6.50, 8.60]	7.94 ±1.58 7.70 [6.90, 8.70]	0.128
Glucose (mg/dl)*; mean (SD) median [IQR]	151.56 ±65.11 135.00 [109.67, 174.00]	169.58 ±67.47 155.00 [125.00, 196.50]	0.272	161.42 ±69.55 145.00 [115.00, 189.00]	168.05 ±67.37 153.50 [124.00, 195.00]	0.097
Creatinine (mg/dl)*; mean (SD) median [IQR]	1.04 ±0.51 0.94 [0.80, 1.14]	1.10 ±0.39 0.99 [0.81, 1.24]	0.133	1.08 ±0.76 0.97 [0.80, 1.19]	1.08 ±0.39 0.97 [0.80, 1.20]	0.010
Systolic blood pressure (mmHg)*; mean (SD) median [IQR]	129.54 ±20.15 130.00 [120.00, 139.00]	130.92 ±18.99 130.00 [120.00, 140.00]	0.071	130.32 ±18.82 130.00 [120.00, 140.00]	130.55 ±19.44 130.00 [120.00, 140.00]	0.012
Heart rate (1/min)*; mean (SD) median [IQR]	77.43 ±74.35 76.00 [68.00, 85.00]	76.98 ±13.25 76.00 [68.00, 85.00]	0.008	79.08 ±156.71 76.00 [68.00, 85.00]	77.03 ±13.13 76.00 [68.00, 85.00]	0.018
BMI (kg/m2)*; mean (SD) median [IQR]	37.54 ±7.79 36.50 [32.23, 41.61]	32.37 ±7.33 31.44 [27.40, 36.30]	0.684	35.12 ±7.56 34.05 [29.95, 39.06]	33.56 ±7.25 32.56 [28.69, 37.40]	0.210
eGFR (ml/min/1.73m2)*; mean (SD) median [IQR]	2,877.13 ±163,592.21 74.00 [57.00, 91.00]	2,420.31 ±153,391.95 68.00 [51.00, 86.00]	0.003	5,607.26 ±235,308.75 69.00 [54.00, 87.00]	2,831.44 ±166,227.44 69.00 [52.00, 87.00]	0.014
LDL (mg/dl)*; mean (SD) median [IQR]	82.19 ±37.24 77.00 [59.00, 102.00]	81.59 ±36.40 77.00 [58.00, 101.00]	0.016	80.29 ±36.35 76.00 [57.30, 100.00]	81.57 ±36.53 77.00 [59.00, 101.00]	0.035
HDL (mg/dl)*; mean (SD) median [IQR]	47.15 ±14.66 46.00 [38.00, 55.00]	46.91 ±13.99 45.00 [38.00, 54.00]	0.016	47.13 ±14.53 45.20 [38.00, 55.00]	46.32 ±13.77 45.00 [37.00, 54.00]	0.057
Total cholesterol (mg/dl)*; mean (SD) median [IQR]	159.25 ±46.47 154.00 [131.00, 184.00]	160.18 ±44.79 155.00 [131.00, 183.00]	0.020	157.53 ±45.43 152.00 [129.00, 182.00]	158.96 ±46.09 154.00 [129.00, 183.00]	0.031
Triglyceride (mg/dl)*; mean (SD) median [IQR]	163.64 ±122.48 138.00 [100.00, 194.00]	163.65 ±102.89 140.00 [101.00, 197.75]	0.000	162.79 ±112.87 138.00 [99.00, 195.00]	165.52 ±103.44 140.00 [102.00, 200.00]	0.025

**Burden of comorbidities**						
Combined comorbidity score; mean (SD) median [IQR]	2.51 ±2.39 2.00 [1.00, 4.00]	3.13 ±2.73 3.00 [1.00, 5.00]	0.242	2.77 ±2.55 2.00 [1.00, 4.00]	2.77 ±2.54 2.00 [1.00, 4.00]	0.001
Frailty Score; mean (SD) median [IQR]	0.18 ±0.05 0.17 [0.14, 0.21]	0.19 ±0.06 0.18 [0.14, 0.22]	0.117	0.18 ±0.06 0.17 [0.14, 0.21]	0.18 ±0.06 0.17 [0.14, 0.21]	0.001
Number of Hospitalizations; mean (SD) median [IQR]	0.21 ±0.63 0.00 [0.00, 0.00]	0.33 ±0.86 0.00 [0.00, 0.00]	0.164	0.25 ±0.73 0.00 [0.00, 0.00]	0.26 ±0.74 0.00 [0.00, 0.00]	0.010
Any hospitalization within prior 91 days	1,839 (3.7%)	2,473 (8.7%)	0.211	805 (5.9%)	813 (6.0%)	0.002
Any hospitalization within prior 92-365 days	6,138 (12.3%)	4,030 (14.3%)	0.057	1,686 (12.4%)	1,737 (12.8%)	0.011
**Baseline hospitalizations and hospital metrics**						
Number of hospitalizations (0, 1, 2 or more)						
< 1	42,618 (85.5%)	22,657 (80.1%)	0.143	11,403 (83.8%)	11,357 (83.5%)	0.009
1 - <2	5,223 (10.5%)	3,518 (12.4%)	0.062	1,485 (10.9%)	1,504 (11.1%)	0.004
>= 2	2,002 (4.0%)	2,104 (7.4%)	0.148	714 (5.2%)	741 (5.4%)	0.009
Heart failure hospitalization	2,061 (4.1%)	1,591 (5.6%)	0.069	636 (4.7%)	643 (4.7%)	0.002
ED visit	16,681 (33.5%)	11,311 (40.0%)	0.136	5,023 (36.9%)	5,017 (36.9%)	0.001
**Calendar year of cohort entry**						
2018	44 (0.1%)	4,704 (16.6%)	0.626	44 (0.3%)	65 (0.5%)	0.024
2019	1,369 (2.7%)	4,855 (17.2%)	0.496	1,203 (8.8%)	1,227 (9.0%)	0.006
2020	2,560 (5.1%)	4,659 (16.5%)	0.371	1,910 (14.0%)	1,893 (13.9%)	0.004
2021	4,304 (8.6%)	4,231 (15.0%)	0.197	2,388 (17.6%)	2,297 (16.9%)	0.018
2022	7,572 (15.2%)	3,451 (12.2%)	0.087	2,535 (18.6%)	2,504 (18.4%)	0.006
2023	15,621 (31.3%)	3,209 (11.3%)	0.503	2,745 (20.2%)	2,788 (20.5%)	0.008
2024	18,081 (36.3%)	3,159 (11.2%)	0.618	2,766 (20.3%)	2,817 (20.7%)	0.009
2025	292 (0.6%)	11 (0.0%)	0.098	11 (0.1%)	11 (0.1%)	0.000

Abbreviations: *not used in the propensity score; ACE, angiotensin-converting enzyme inhibitors; ARB, angiotensin receptor blocker; ARNI, angiotensin receptor/neprilysin inhibitor; BMI, body mass index; CABG, coronary artery bypass graft surgery; CED, cohort entry date; CKD, chronic kidney disease; COPD, chronic obstructive pulmonary disease; DKA, diabetic ketoacidosis; DPP4i, dipeptidyl peptidase-4 inhibitors; ED, emergency department; eGFR, estimated glomerular filtration rate (estimated using the quadratic GFR equation: GFR = EXP(1.911 + (5.249 / Serum creatinine) - (2.114 / (Serum creatinine^2)) - (0.00686 * Age) - 0.205 (if female))); HbA1c, hemoglobin A1c; HDL, high-density lipoprotein cholesterol; HONK, hyperglycemic hyperosmolar nonketotic state; IQR, inter-quartile-range; MASH, metabolic dysfunction associated steatohepatitis; MASLD, metabolic dysfunction associated steatotic liver disease; MI, myocardial infarction; N, number of participants; NSAIDs, non-steroidal anti-inflammatory drugs; PCSK9, proprotein convertase subtilisin/kexin type 9; PSA, prostate-specific antigen; PTCA, percutaneous transluminal coronary angioplasty; SGLT2i, sodium-glucose transport protein 2 inhibitors; SD, standard deviation; SMD, standardized mean difference; TIA, transient ischemic attack.
Missing data were handled by assuming absence of a code indicated absence of the condition for most binary covariates. Missing indicators were included for race and region in the propensity score model.
Laboratory values were only available in a subset of the Optum database and were truncated using clinically plausible cut-off values (BMI values >100 or <10 were set to missing; missingness before matching ~58%. Creatinine values >30 were set to missing, and values <0.8 were set to 0.8; missingness before matching ~39%. eGFR values >150 were set to 150, and values =0 were set to missing; missingness before matching ~69%. Glucose values <30 were set to missing; missingness before matching ~39%. HbA1c values ≥20 or ≤2 were set to missing; missingness before matching ~34%. HDL value missingness before matching was ~47%. Heart rate values <30 were set to missing; missingness before matching ~71%. LDL value missingness before matching was ~44%. Systolic blood pressure values <30 were set to missing; missingness before matching

~56%. Total cholesterol value missingness before matching was ~47%. Triglyceride value missingness before matching was ~48%.).
The Race-Others category includes Asian and Hispanic individuals.

Supplementary Table 4. Baseline characteristics of initiators of semaglutide vs sitagliptin when applying trial eligibility criteria before and after propensity score matching, Marketscan databases. Values are number (percentage) unless otherwise specified.

	Before propensity score matching			After propensity score matching		
Variable	Semaglutide (n = 19,713)	Sitagliptin (n = 13,885)	SMD	Semaglutide (n = 6,554)	Sitagliptin (n = 6,554)	SMD
Demographics						
Age; mean (SD) median [IQR]	67.49 ±7.76 68.00 [62.00, 73.00]	72.34 ±7.50 72.00 [68.00, 78.00]	0.636	70.57 ±7.43 71.00 [66.00, 76.00]	70.56 ±7.47 71.00 [67.00, 75.00]	0.001
Gender						
Male	21,624 (43.4%)	12,564 (44.4%)	0.021	6,005 (44.1%)	5,991 (44.0%)	0.002
Female	28,219 (56,6%)	15,715 (55,6%)	0.021	7,597 (55,9%)	7,611 (56,0%)	0.002
Race						
White	24,640 (49.4%)	12,998 (46.0%)	0.070	6,583 (48.4%)	6,563 (48.3%)	0.003
Black	6,869 (13.8%)	4,762 (16.8%)	0.085	2,152 (15.8%)	2,143 (15.8%)	0.002
Unknown / Missing	17,616 (35.3%)	9,385 (33.2%)	0.045	4,553 (33.5%)	4,585 (33.7%)	0.005
Others	718 (1.4%)	1,134 (4.0%)	0.158	314 (2.3%)	311 (2.3%)	0.001
Region / State						
Northeast	4,487 (9.0%)	2,992 (10.6%)	0.053	1,359 (10.0%)	1,409 (10.4%)	0.012
Midwest / North central	9,445 (18.9%)	3,803 (13.4%)	0.150	2,185 (16.1%)	2,162 (15.9%)	0.005
South	27,747 (55.7%)	16,289 (57.6%)	0.039	7,811 (57.4%)	7,790 (57.3%)	0.003
West	8,143 (16.3%)	5,182 (18.3%)	0.053	2,240 (16.5%)	2,238 (16.5%)	0.000
Missing	21 (0.0%)	13 (0.0%)	0.002	7 (0.1%)	3 (0.0%)	0.015
Lifestyle risk factors						
Smoking/Tobacco use	13,895 (27.9%)	7,220 (25.5%)	0.053	3,501 (25.7%)	3,512 (25.8%)	0.002
Weight						
Underweight	63 (0.1%)	388 (1.4%)	0.145	56 (0.4%)	52 (0.4%)	0.005
Normal weight	724 (1.5%)	2,662 (9.4%)	0.357	553 (4.1%)	570 (4.2%)	0.006
Overweight (BMI 25.0-29.9)	5,293 (10.6%)	6,852 (24.2%)	0.365	2,646 (19.5%)	2,612 (19.2%)	0.006
Class 1 Obesity (BMI 30.0-34.9)	5,185 (10.4%)	3,959 (14.0%)	0.110	1,844 (13.6%)	1,848 (13.6%)	0.001
Class 2 Obesity (BMI 35.0-39.9)	3,415 (6.9%)	1,703 (6.0%)	0.034	928 (6.8%)	892 (6.6%)	0.011
Class 3 Obesity; (BMI 40.0 and above)	19,481 (39.1%)	5,460 (19.3%)	0.446	3,554 (26.1%)	3,557 (26.2%)	0.001
Unspecified Obesity	15,682 (31.5%)	7,255 (25.7%)	0.129	4,021 (29.6%)	4,071 (29.9%)	0.008
Diabetes complications						
Diabetic retinopathy	4,047 (8.1%)	3,077 (10.9%)	0.094	1,393 (10.2%)	1,335 (9.8%)	0.014
Diabetic neuropathy	14,480 (29.1%)	9,795 (34.6%)	0.120	4,536 (33.3%)	4,474 (32.9%)	0.010
Diabetic nephropathy	12,927 (25.9%)	10,091 (35.7%)	0.212	4,296 (31.6%)	4,260 (31.3%)	0.006
Diabetes with other ophthalmic complications	2,382 (4.8%)	1,862 (6.6%)	0.078	797 (5.9%)	805 (5.9%)	0.002
Diabetes with peripheral circulatory disorders	8,322 (16.7%)	5,796 (20.5%)	0.098	2,607 (19.2%)	2,579 (19.0%)	0.005
Diabetic foot	1,434 (2.9%)	898 (3.2%)	0.017	439 (3.2%)	399 (2.9%)	0.017

Erectile dysfunction	2,471 (5.0%)	1,064 (3.8%)	0.059	578 (4.2%)	577 (4.2%)	0.000
Hypoglycemia	12,276 (24.6%)	7,495 (26.5%)	0.043	3,607 (26.5%)	3,521 (25.9%)	0.014
Hyperglycemia/DKA/HONK	23,311 (46.8%)	14,525 (51.4%)	0.092	7,018 (51.6%)	6,883 (50.6%)	0.020
Skin infections	5,344 (10.7%)	2,841 (10.0%)	0.022	1,381 (10.2%)	1,368 (10.1%)	0.003
**Cardiovascular-related conditions**						
Coronary atherosclerosis	18,491 (37.1%)	10,186 (36.0%)	0.022	4,844 (35.6%)	4,878 (35.9%)	0.005
Stable angina	3,737 (7.5%)	1,990 (7.0%)	0.018	966 (7.1%)	975 (7.2%)	0.003
Unstable angina	1,795 (3.6%)	1,001 (3.5%)	0.003	443 (3.3%)	465 (3.4%)	0.009
Hypertension	46,038 (92.4%)	26,616 (94.1%)	0.07	12,714 (93.5%)	12,721 (93.5%)	0.002
Hypotension	1,864 (3.7%)	1,411 (5.0%)	0.061	593 (4.4%)	568 (4.2%)	0.009
Hyperlipidemia	43,970 (88.2%)	25,165 (89.0%)	0.024	12,150 (89.3%)	12,081 (88.8%)	0.016
Acute MI	544 (1.1%)	352 (1.2%)	0.014	146 (1.1%)	161 (1.2%)	0.010
Old MI	3,370 (6.8%)	2,207 (7.8%)	0.040	930 (6.8%)	985 (7.2%)	0.016
Ischemic stroke	354 (0.7%)	264 (0.9%)	0.025	114 (0.8%)	110 (0.8%)	0.003
TIA	1,354 (2.7%)	982 (3.5%)	0.044	392 (2.9%)	394 (2.9%)	0.001
Cardiac conduction disorder	4,036 (8.1%)	2,488 (8.8%)	0.025	1,140 (8.4%)	1,121 (8.2%)	0.005
Previous cardiac procedure (CABG, PTCA, Stent)	1,178 (2.4%)	614 (2.2%)	0.013	279 (2.1%)	308 (2.3%)	0.015
PVD diagnosis or surgery	6,364 (12.8%)	4,767 (16.9%)	0.115	2,031 (14.9%)	2,002 (14.7%)	0.006
Atrial fibrillation	7,576 (15.2%)	4,414 (15.6%)	0.011	2,008 (14.8%)	2,087 (15.3%)	0.016
Other cardiac dysrhythmia	13,141 (26.4%)	7,334 (25.9%)	0.010	3,463 (25.5%)	3,459 (25.4%)	0.001
Heart failure	10,804 (21.7%)	6,093 (21.5%)	0.003	2,785 (20.5%)	2,837 (20.9%)	0.009
Acute heart failure	2,170 (4.4%)	1,397 (4.9%)	0.028	570 (4.2%)	612 (4.5%)	0.015
Cardiomyopathy	3,880 (7.8%)	2,291 (8.1%)	0.012	1,005 (7.4%)	1,046 (7.7%)	0.011
Valve disorders	7,987 (16.0%)	4,810 (17.0%)	0.027	2,180 (16.0%)	2,179 (16.0%)	0.000
Valve replacement	940 (1.9%)	585 (2.1%)	0.013	286 (2.1%)	274 (2.0%)	0.006
Edema	8,407 (16.9%)	4,621 (16.3%)	0.014	2,200 (16.2%)	2,246 (16.5%)	0.009
Venous thromboembolism / Pulmonary embolism	1,998 (4.0%)	1,111 (3.9%)	0.004	552 (4.1%)	551 (4.1%)	0.000
Pulmonary hypertension	2,152 (4.3%)	1,292 (4.6%)	0.012	566 (4.2%)	579 (4.3%)	0.005
Implantable cardioverter defibrillator	186 (0.4%)	128 (0.5%)	0.012	50 (0.4%)	55 (0.4%)	0.006
Hyperkalemia	1,585 (3.2%)	1,407 (5.0%)	0.091	579 (4.3%)	577 (4.2%)	0.001
Cerebrovascular procedure	71 (0.1%)	39 (0.1%)	0.001	21 (0.2%)	18 (0.1%)	0.006
Insertion of pacemakers / removal of cardiac lead	117 (0.2%)	101 (0.4%)	0.023	42 (0.3%)	39 (0.3%)	0.004
**Renal-related conditions**						
Hypertensive nephropathy	8,492 (17.0%)	6,296 (22.3%)	0.132	2,710 (19.9%)	2,713 (19.9%)	0.001
CKD Stage 1-2	3,068 (6.2%)	2,511 (8.9%)	0.103	1,010 (7.4%)	1,031 (7.6%)	0.006
CKD Stage 3-4	11,499 (23.1%)	8,496 (30.0%)	0.158	3,704 (27.2%)	3,685 (27.1%)	0.003
Unspecified CKD	3,524 (7.1%)	2,778 (9.8%)	0.099	1,152 (8.5%)	1,164 (8.6%)	0.003
Microalbuminuria or proteinuria	1,194 (2.4%)	771 (2.7%)	0.021	390 (2.9%)	395 (2.9%)	0.002
Acute kidney injury	3,362 (6.7%)	2,911 (10.3%)	0.127	1,178 (8.7%)	1,197 (8.8%)	0.005

Urinary tract infections	7,303 (14.7%)	5,313 (18.8%)	0.111	2,230 (16.4%)	2,280 (16.8%)	0.010
Genital infections	1,420 (2.8%)	944 (3.3%)	0.028	475 (3.5%)	458 (3.4%)	0.007
Urolithiasis (Kidney and urinary stone)	2,620 (5.3%)	1,374 (4.9%)	0.018	702 (5.2%)	685 (5.0%)	0.006
**Other comorbidities**						
COPD	8,574 (17.2%)	5,055 (17.9%)	0.018	2,373 (17.4%)	2,337 (17.2%)	0.007
Asthma	7,069 (14.2%)	3,032 (10.7%)	0.105	1,681 (12.4%)	1,644 (12.1%)	0.008
Obstructive sleep apnea	18,902 (37.9%)	5,849 (20.7%)	0.386	3,697 (27.2%)	3,657 (26.9%)	0.007
Serious bacterial infections	1,402 (2.8%)	1,292 (4.6%)	0.093	465 (3.4%)	474 (3.5%)	0.004
Pneumonia	2,842 (5.7%)	1,984 (7.0%)	0.054	831 (6.1%)	873 (6.4%)	0.013
Liver disease	9,167 (18.4%)	4,797 (17.0%)	0.037	2,381 (17.5%)	2,469 (18.2%)	0.017
MASH / MASLD	4,967 (10.0%)	1,928 (6.8%)	0.114	1,117 (8.2%)	1,149 (8.4%)	0.009
Fractures / Falls	3,511 (7.0%)	2,595 (9.2%)	0.078	1,075 (7.9%)	1,076 (7.9%)	0.000
Osteoporosis	2,895 (5.8%)	2,272 (8.0%)	0.088	918 (6.7%)	923 (6.8%)	0.001
Osteoarthritis	18,670 (37.5%)	9,682 (34.2%)	0.067	4,780 (35.1%)	4,798 (35.3%)	0.003
Depression	12,110 (24.3%)	6,237 (22.1%)	0.053	3,076 (22.6%)	3,064 (22.5%)	0.002
Dementia	2,026 (4.1%)	2,753 (9.7%)	0.225	920 (6.8%)	904 (6.6%)	0.005
Delirium or psychosis	724 (1.5%)	636 (2.2%)	0.059	250 (1.8%)	238 (1.7%)	0.007
Anxiety	11,468 (23.0%)	4,857 (17.2%)	0.146	2,589 (19.0%)	2,573 (18.9%)	0.003
Sleep disorders	20,010 (40.1%)	10,376 (36.7%)	0.071	5,089 (37.4%)	5,059 (37.2%)	0.005
Anemia	10,534 (21.1%)	7,339 (26.0%)	0.114	3,192 (23.5%)	3,229 (23.7%)	0.006
Influenza	861 (1.7%)	493 (1.7%)	0.001	183 (1.3%)	212 (1.6%)	0.018
COVID	5,237 (10.5%)	1,592 (5.6%)	0.180	1,154 (8.5%)	1,136 (8.4%)	0.005
Hyperthyroidism and other thyroid gland disorders	14,713 (29.5%)	8,014 (28.3%)	0.026	3,864 (28.4%)	3,912 (28.8%)	0.008
Hypothyroidism	12,013 (24.1%)	6,582 (23.3%)	0.019	3,183 (23.4%)	3,201 (23.5%)	0.003
Nephrotic syndrome	5 (0.0%)	4 (0.0%)	0.004	3 (0.0%)	2 (0.0%)	0.005
Urinary incontinence	3,556 (7.1%)	2,040 (7.2%)	0.003	982 (7.2%)	975 (7.2%)	0.002
Biliary disease	97 (0.2%)	69 (0.2%)	0.011	29 (0.2%)	29 (0.2%)	0.0
Pancreatitis	23 (0.0%)	12 (0.0%)	0.002	6 (0.0%)	6 (0.0%)	0.0
Bowel obstruction	69 (0.1%)	53 (0.2%)	0.012	19 (0.1%)	23 (0.2%)	0.007
Gastroparesis	367 (0.7%)	242 (0.9%)	0.013	121 (0.9%)	114 (0.8%)	0.006
**Diabetes medications**						
Number of antidiabetic drugs on CED; mean (SD) median [IQR]	1.98 ±0.86 2.00 [1.00, 3.00]	2.14 ±0.81 2.00 [2.00, 3.00]	0.193	2.14 ±0.86 2.00 [2.00, 3.00]	2.12 ±0.82 2.00 [2.00, 3.00]	0.020
Concomitant use or initiation of Metformin	23,643 (47.4%)	16,358 (57.8%)	0.210	7,515 (55.2%)	7,470 (54.9%)	0.007
Concomitant use or initiation of Insulins	6,497 (13.0%)	2,690 (9.5%)	0.112	1,640 (12.1%)	1,554 (11.4%)	0.020
Concomitant use or initiation of Sulfonylureas	8,551 (17.2%)	8,769 (31.0%)	0.328	3,600 (26.5%)	3,495 (25.7%)	0.018
Concomitant use or initiation of SGLT-2i	7,609 (15.3%)	2,722 (9.6%)	0.171	1,905 (14.0%)	1,885 (13.9%)	0.004
Concomitant use or initiation of Any other glucose-lowering drugs	2,685 (5.4%)	1,813 (6.4%)	0.043	813 (6.0%)	843 (6.2%)	0.009
Past use of Metformin	31,269 (62.7%)	19,078 (67.5%)	0.099	9,199 (67.6%)	9,130 (67.1%)	0.011
Past use of Insulins	9,352 (18.8%)	3,977 (14.1%)	0.127	2,336 (17.2%)	2,247 (16.5%)	0.017

Past use of Sulfonylureas	11,844 (23.8%)	11,407 (40.3%)	0.361	4,780 (35.1%)	4,690 (34.5%)	0.014
Past use of SGLT-2i	10,710 (21.5%)	4,150 (14.7%)	0.178	2,864 (21.1%)	2,783 (20.5%)	0.015
Past use of Any other glucose-lowering drugs	3,761 (7.5%)	2,678 (9.5%)	0.069	1,221 (9.0%)	1,230 (9.0%)	0.002
Other medications						
ACE / ARB	36,407 (73.0%)	21,648 (76.6%)	0.081	10,250 (75.4%)	10,263 (75.5%)	0.002
ARNI	1,308 (2.6%)	428 (1.5%)	0.078	259 (1.9%)	257 (1.9%)	0.001
Thiazides	17,664 (35.4%)	9,623 (34.0%)	0.030	4,719 (34.7%)	4,714 (34.7%)	0.001
Beta-blockers	26,670 (53.5%)	15,520 (54.9%)	0.028	7,310 (53.7%)	7,363 (54.1%)	0.008
Calcium channel blockers	17,766 (35.6%)	11,079 (39.2%)	0.073	5,149 (37.9%)	5,141 (37.8%)	0.001
Digoxin / Digitoxin	464 (0.9%)	516 (1.8%)	0.077	177 (1.3%)	198 (1.5%)	0.013
Loop diuretics	12,730 (25.5%)	6,593 (23.3%)	0.052	3,214 (23.6%)	3,224 (23.7%)	0.002
Other diuretics	5,875 (11.8%)	2,393 (8.5%)	0.110	1,295 (9.5%)	1,297 (9.5%)	0.001
Intravenous diuretics	910 (1.8%)	514 (1.8%)	0.001	216 (1.6%)	217 (1.6%)	0.001
Nitrates	4,979 (10.0%)	2,841 (10.0%)	0.002	1,314 (9.7%)	1,332 (9.8%)	0.004
Anti-arrhythmics	1,885 (3.8%)	933 (3.3%)	0.026	450 (3.3%)	477 (3.5%)	0.011
Statins	40,844 (81.9%)	23,398 (82.7%)	0.021	11,337 (83.3%)	11,337 (83.3%)	0.000
PCSK9 inhibitors and other lipid-lowering drugs	7,683 (15.4%)	3,633 (12.8%)	0.074	1,967 (14.5%)	1,951 (14.3%)	0.003
Antiplatelet medications	6,992 (14.0%)	4,106 (14.5%)	0.014	1,808 (13.3%)	1,906 (14.0%)	0.021
Oral anticoagulants	7,548 (15.1%)	3,966 (14.0%)	0.032	1,925 (14.2%)	1,985 (14.6%)	0.013
COPD / Asthma medications	19,893 (39.9%)	9,683 (34.2%)	0.118	5,022 (36.9%)	4,983 (36.6%)	0.006
NSAIDS	16,906 (33.9%)	8,230 (29.1%)	0.104	4,261 (31.3%)	4,321 (31.8%)	0.009
Oral corticosteroids	14,125 (28.3%)	5,976 (21.1%)	0.168	3,152 (23.2%)	3,202 (23.5%)	0.009
Osteoporosis agents (incl. bisphosphonates)	1,676 (3.4%)	1,379 (4.9%)	0.076	536 (3.9%)	542 (4.0%)	0.002
Opioids	17,708 (35.5%)	8,809 (31.2%)	0.093	4,400 (32.3%)	4,334 (31.9%)	0.010
Anti-depressants	21,362 (42.9%)	9,311 (32.9%)	0.206	4,959 (36.5%)	4,950 (36.4%)	0.001
Antipsychotics	2,421 (4.9%)	1,193 (4.2%)	0.031	587 (4.3%)	590 (4.3%)	0.001
Anxiolytics / hypnotics, benzos	11,876 (23.8%)	5,399 (19.1%)	0.116	2,694 (19.8%)	2,733 (20.1%)	0.007
Dementia medications	819 (1.6%)	1,396 (4.9%)	0.185	426 (3.1%)	433 (3.2%)	0.003
Urinary tract infections antibiotics	24,575 (49.3%)	13,197 (46.7%)	0.053	6,456 (47.5%)	6,436 (47.3%)	0.003
Laxatives	1,870 (3.8%)	1,053 (3.7%)	0.001	452 (3.3%)	454 (3.3%)	0.001
Healthcare utilization marker						
Number of distinct medications; mean (SD) median [IQR]	15.47 ±6.76 15.00 [11.00, 19.00]	14.66 ±6.48 14.00 [10.00, 18.00]	0.122	15.05 ±6.46 14.00 [10.00, 19.00]	15.01 ±6.61 14.00 [10.00, 19.00]	0.006
Number of office visits; mean (SD) median [IQR]	10.81 ±7.12 9.00 [6.00, 14.00]	9.88 ±6.83 8.00 [5.00, 13.00]	0.133	10.22 ±6.63 9.00 [6.00, 13.00]	10.17 ±7.01 9.00 [5.00, 14.00]	0.007
Number of endocrinologist visits; mean (SD) median [IQR]	0.37 ±1.24 0.00 [0.00, 0.00]	0.30 ±1.20 0.00 [0.00, 0.00]	0.057	0.34 ±1.08 0.00 [0.00, 0.00]	0.33 ±1.34 0.00 [0.00, 0.00]	0.008
Number of cardiologist visits; mean (SD) median [IQR]	2.23 ±3.87 1.00 [0.00, 3.00]	2.24 ±3.96 1.00 [0.00, 3.00]	0.002	2.12 ±3.76 1.00 [0.00, 3.00]	2.16 ±3.95 1.00 [0.00, 3.00]	0.011
Number of internal/family medicine visits; mean (SD) median [IQR]	8.05 ±8.56 6.00 [3.00, 10.00]	9.21 ±9.36 7.00 [4.00, 12.00]	0.129	8.72 ±9.25 6.00 [3.00, 11.00]	8.63 ±9.11 6.00 [3.00, 11.00]	0.010
Number of electrocardiograms (ECG/EKG); mean (SD) median [IQR]	1.22 ±1.75 1.00 [0.00, 2.00]	1.30 ±1.90 1.00 [0.00, 2.00]	0.043	1.19 ±1.72 1.00 [0.00, 2.00]	1.23 ±1.87 1.00 [0.00, 2.00]	0.022
Number of echocardiograms; mean (SD) median [IQR]	0.39 ±0.71 0.00 [0.00, 1.00]	0.38 ±0.70 0.00 [0.00, 1.00]	0.009	0.37 ±0.69 0.00 [0.00, 1.00]	0.37 ±0.70 0.00 [0.00, 1.00]	0.009

Out-of-pocket medication cost; mean (SD) median [IQR]	606.72 ±828.66 366.06 [146.95, 800.31]	518.72 ±663.16 306.90 [125.23, 687.83]	0.117	546.68 ±605.66 345.01 [143.55, 743.16]	547.44 ±736.98 303.45 [127.15, 718.12]	0.001
Unique brand medicines; mean (SD) median [IQR]	15.71 ±6.95 15.00 [11.00, 20.00]	14.90 ±6.67 14.00 [10.00, 19.00]	0.118	15.30 ±6.65 14.00 [11.00, 19.00]	15.25 ±6.80 14.00 [10.00, 19.00]	0.007
Unique generic medicines; mean (SD) median [IQR]	15.47 ±6.76 15.00 [11.00, 19.00]	14.66 ±6.48 14.00 [10.00, 18.00]	0.122	15.05 ±6.46 14.00 [10.00, 19.00]	15.01 ±6.61 14.00 [10.00, 19.00]	0.006
Ratio of brand to generic medications; mean (SD) median [IQR]	1.01 ±0.03 1.00 [1.00, 1.00]	1.01 ±0.04 1.00 [1.00, 1.00]	0.027	1.01 ±0.04 1.00 [1.00, 1.00]	1.01 ±0.04 1.00 [1.00, 1.00]	0.010
Healthy behavior markers						
Colonoscopy / Sigmoidoscopy	5,709 (11.5%)	2,631 (9.3%)	0.071	1,402 (10.3%)	1,391 (10.2%)	0.003
Flu Pneumococcal vaccine	17,564 (35.2%)	11,130 (39.4%)	0.085	4,992 (36.7%)	5,013 (36.9%)	0.003
Pap smear	2,659 (5.3%)	937 (3.3%)	0.100	537 (3.9%)	530 (3.9%)	0.003
PSA test	11,948 (24.0%)	6,262 (22.1%)	0.043	3,112 (22.9%)	3,133 (23.0%)	0.004
Fecal occult blood test	2,370 (4.8%)	2,515 (8.9%)	0.165	920 (6.8%)	914 (6.7%)	0.002
Bone mineral density tests	4,838 (9.7%)	2,550 (9.0%)	0.024	1,240 (9.1%)	1,246 (9.2%)	0.002
Mammograms	14,310 (28.7%)	6,299 (22.3%)	0.148	3,454 (25.4%)	3,420 (25.1%)	0.006
Telemedicine	12,448 (25.0%)	4,742 (16.8%)	0.203	3,147 (23.1%)	3,070 (22.6%)	0.013
Laboratory and diagnostic tests						
HbA1c tests; mean (SD) median [IQR]	2.36 ±1.32 2.00 [1.00, 3.00]	2.45 ±1.30 2.00 [2.00, 3.00]	0.068	2.47 ±1.36 2.00 [2.00, 3.00]	2.45 ±1.28 2.00 [2.00, 3.00]	0.011
Lipid panels; mean (SD) median [IQR]	1.69 ±1.19 2.00 [1.00, 2.00]	1.74 ±1.21 2.00 [1.00, 2.00]	0.044	1.73 ±1.22 2.00 [1.00, 2.00]	1.72 ±1.18 2.00 [1.00, 2.00]	0.009
Creatinine tests; mean (SD) median [IQR]	3.17 ±2.69 3.00 [2.00, 4.00]	3.40 ±3.50 3.00 [2.00, 4.00]	0.073	3.30 ±2.88 3.00 [2.00, 4.00]	3.30 ±2.52 3.00 [2.00, 4.00]	0.000
Natriuretic peptide tests; mean (SD) median [IQR]	0.24 ±0.81 0.00 [0.00, 0.00]	0.23 ±2.29 0.00 [0.00, 0.00]	0.005	0.21 ±0.69 0.00 [0.00, 0.00]	0.21 ±0.70 0.00 [0.00, 0.00]	0.002
Urine tests; mean (SD) median [IQR]	1.12 ±1.66 1.00 [0.00, 2.00]	1.32 ±2.82 1.00 [0.00, 2.00]	0.088	1.23 ±1.80 1.00 [0.00, 2.00]	1.22 ±1.73 1.00 [0.00, 2.00]	0.007
Lab values						
HbA1c (%)*; mean (SD) median [IQR]	7.44 ±1.62 7.00 [6.30, 8.20]	7.93 ±1.59 7.70 [6.80, 8.70]	0.305	7.73 ±1.70 7.40 [6.50, 8.60]	7.94 ±1.58 7.70 [6.90, 8.70]	0.128
Glucose (mg/dl)*; mean (SD) median [IQR]	151.56 ±65.11 135.00 [109.67, 174.00]	169.58 ±67.47 155.00 [125.00, 196.50]	0.272	161.42 ±69.55 145.00 [115.00, 189.00]	168.05 ±67.37 153.50 [124.00, 195.00]	0.097
Creatinine (mg/dl)*; mean (SD) median [IQR]	1.04 ±0.51 0.94 [0.80, 1.14]	1.10 ±0.39 0.99 [0.81, 1.24]	0.133	1.08 ±0.76 0.97 [0.80, 1.19]	1.08 ±0.39 0.97 [0.80, 1.20]	0.010
Systolic blood pressure (mmHg)*; mean (SD) median [IQR]	129.54 ±20.15 130.00 [120.00, 139.00]	130.92 ±18.99 130.00 [120.00, 140.00]	0.071	130.32 ±18.82 130.00 [120.00, 140.00]	130.55 ±19.44 130.00 [120.00, 140.00]	0.012
Heart rate (1/min)*; mean (SD) median [IQR]	77.43 ±74.35 76.00 [68.00, 85.00]	76.98 ±13.25 76.00 [68.00, 85.00]	0.008	79.08 ±156.71 76.00 [68.00, 85.00]	77.03 ±13.13 76.00 [68.00, 85.00]	0.018
BMI (kg/m2)*; mean (SD) median [IQR]	37.54 ±7.79 36.50 [32.23, 41.61]	32.37 ±7.33 31.44 [27.40, 36.30]	0.684	35.12 ±7.56 34.05 [29.95, 39.06]	33.56 ±7.25 32.56 [28.69, 37.40]	0.210
eGFR (ml/min/1.73m2)*; mean (SD) median [IQR]	2,877.13 ±163,592.21 74.00 [57.00, 91.00]	2,420.31 ±153,391.95 68.00 [51.00, 86.00]	0.003	5,607.26 ±235,308.75 69.00 [54.00, 87.00]	2,831.44 ±166,227.44 69.00 [52.00, 87.00]	0.014
LDL (mg/dl)*; mean (SD) median [IQR]	82.19 ±37.24 77.00 [59.00, 102.00]	81.59 ±36.40 77.00 [58.00, 101.00]	0.016	80.29 ±36.35 76.00 [57.30, 100.00]	81.57 ±36.53 77.00 [59.00, 101.00]	0.035
HDL (mg/dl)*; mean (SD) median [IQR]	47.15 ±14.66 46.00 [38.00, 55.00]	46.91 ±13.99 45.00 [38.00, 54.00]	0.016	47.13 ±14.53 45.20 [38.00, 55.00]	46.32 ±13.77 45.00 [37.00, 54.00]	0.057
Total cholesterol (mg/dl)*; mean (SD) median [IQR]	159.25 ±46.47 154.00 [131.00, 184.00]	160.18 ±44.79 155.00 [131.00, 183.00]	0.020	157.53 ±45.43 152.00 [129.00, 182.00]	158.96 ±46.09 154.00 [129.00, 183.00]	0.031
Triglyceride (mg/dl)*; mean (SD) median [IQR]	163.64 ±122.48 138.00 [100.00, 194.00]	163.65 ±102.89 140.00 [101.00, 197.75]	0.000	162.79 ±112.87 138.00 [99.00, 195.00]	165.52 ±103.44 140.00 [102.00, 200.00]	0.025

**Burden of comorbidities**						
Combined comorbidity score; mean (SD) median [IQR]	2.51 ±2.39 2.00 [1.00, 4.00]	3.13 ±2.73 3.00 [1.00, 5.00]	0.242	2.77 ±2.55 2.00 [1.00, 4.00]	2.77 ±2.54 2.00 [1.00, 4.00]	0.001
Frailty Score; mean (SD) median [IQR]	0.18 ±0.05 0.17 [0.14, 0.21]	0.19 ±0.06 0.18 [0.14, 0.22]	0.117	0.18 ±0.06 0.17 [0.14, 0.21]	0.18 ±0.06 0.17 [0.14, 0.21]	0.001
Number of Hospitalizations; mean (SD) median [IQR]	0.21 ±0.63 0.00 [0.00, 0.00]	0.33 ±0.86 0.00 [0.00, 0.00]	0.164	0.25 ±0.73 0.00 [0.00, 0.00]	0.26 ±0.74 0.00 [0.00, 0.00]	0.010
Any hospitalization within prior 91 days	1,839 (3.7%)	2,473 (8.7%)	0.211	805 (5.9%)	813 (6.0%)	0.002
Any hospitalization within prior 92-365 days	6,138 (12.3%)	4,030 (14.3%)	0.057	1,686 (12.4%)	1,737 (12.8%)	0.011
**Baseline hospitalizations and hospital metrics**						
Number of hospitalizations (0, 1, 2 or more)						
< 1	42,618 (85.5%)	22,657 (80.1%)	0.143	11,403 (83.8%)	11,357 (83.5%)	0.009
1 - <2	5,223 (10.5%)	3,518 (12.4%)	0.062	1,485 (10.9%)	1,504 (11.1%)	0.004
>= 2	2,002 (4.0%)	2,104 (7.4%)	0.148	714 (5.2%)	741 (5.4%)	0.009
Heart failure hospitalization	2,061 (4.1%)	1,591 (5.6%)	0.069	636 (4.7%)	643 (4.7%)	0.002
ED visit	16,681 (33.5%)	11,311 (40.0%)	0.136	5,023 (36.9%)	5,017 (36.9%)	0.001
**Calendar year of cohort entry**						
2018	44 (0.1%)	4,704 (16.6%)	0.626	44 (0.3%)	65 (0.5%)	0.024
2019	1,369 (2.7%)	4,855 (17.2%)	0.496	1,203 (8.8%)	1,227 (9.0%)	0.006
2020	2,560 (5.1%)	4,659 (16.5%)	0.371	1,910 (14.0%)	1,893 (13.9%)	0.004
2021	4,304 (8.6%)	4,231 (15.0%)	0.197	2,388 (17.6%)	2,297 (16.9%)	0.018
2022	7,572 (15.2%)	3,451 (12.2%)	0.087	2,535 (18.6%)	2,504 (18.4%)	0.006
2023	15,621 (31.3%)	3,209 (11.3%)	0.503	2,745 (20.2%)	2,788 (20.5%)	0.008
2024	18,081 (36.3%)	3,159 (11.2%)	0.618	2,766 (20.3%)	2,817 (20.7%)	0.009
2025	292 (0.6%)	11 (0.0%)	0.098	11 (0.1%)	11 (0.1%)	0.000

Abbreviations: *not used in the propensity score; ACE, angiotensin-converting enzyme inhibitors; ARB, angiotensin receptor blocker; ARNI, angiotensin receptor/neprilysin inhibitor; BMI, body mass index; CABG, coronary artery bypass graft surgery; CED, cohort entry date; CKD, chronic kidney disease; COPD, chronic obstructive pulmonary disease; DKA, diabetic ketoacidosis; DPP4i, dipeptidyl peptidase-4 inhibitors; ED, emergency department; eGFR, estimated glomerular filtration rate (estimated using the quadratic GFR equation: GFR = EXP(1.911 + (5.249 / Serum creatinine) - (2.114 / (Serum creatinine^2)) - (0.00686 * Age) - 0.205 (if female))); HbA1c, hemoglobin A1c; HDL, high-density lipoprotein cholesterol; HONK, hyperglycemic hyperosmolar nonketotic state; IQR, inter-quartile-range; MASH, metabolic dysfunction associated steatohepatitis; MASLD, metabolic dysfunction associated steatotic liver disease; MI, myocardial infarction; N, number of participants; NSAIDs, non-steroidal anti-inflammatory drugs; PCSK9, proprotein convertase subtilisin/kexin type 9; PSA, prostate-specific antigen; PTCA, percutaneous transluminal coronary angioplasty; SGLT2i, sodium-glucose transport protein 2 inhibitors; SD, standard deviation; SMD, standardized mean difference; TIA, transient ischemic attack.
Missing data were handled by assuming absence of a code indicated absence of the condition for most binary covariates. Missing indicators were included for race and region in the propensity score model.
The Race-Others category includes Asian and Hispanic individuals.

Supplementary Table 5. Baseline characteristics of initiators of semaglutide vs sitagliptin when applying trial eligibility criteria before and after propensity score matching, Medicare database. Values are number (percentage) unless otherwise specified.

	Before propensity score matching			After propensity score matching		
Variable	Semaglutide (n = 7,980)	Sitagliptin (n = 38,610)	SMD	Semaglutide (n = 6,877)	Sitagliptin (n = 6,877)	SMD
Demographics						
Age; mean (SD) median [IQR]	72.12 ±4.96 71.00 [68.00, 75.00]	75.45 ±6.86 74.00 [70.00, 80.00]	0.557	72.41 ±5.05 72.00 [68.00, 75.00]	72.48 ±5.37 71.00 [68.00, 76.00]	0.013
Gender						
Male	3,846 (48.2%)	16,946 (43.9%)	0.086	3,281 (47.7%)	3,252 (47.3%)	0.008
Female	4,134 (51,8%)	21,664 (56,1%)	0.086	3,596 (52,3%)	3,625 (52,7%)	0.008
Race						
White	6,706 (84.0%)	29,995 (77.7%)	0.162	5,737 (83.4%)	5,762 (83.8%)	0.010
Other	327 (4.1%)	2,757 (7.1%)	0.132	302 (4.4%)	304 (4.4%)	0.001
Black	664 (8.3%)	4,419 (11.4%)	0.105	598 (8.7%)	581 (8.4%)	0.009
Unknown / Missing	283 (3.5%)	1,439 (3.7%)	0.010	240 (3.5%)	230 (3.3%)	0.008
Region / State						
Northeast	1,264 (15.8%)	7,473 (19.4%)	0.092	1,092 (15.9%)	1,092 (15.9%)	0.000
Midwest / North central	1,845 (23.1%)	8,005 (20.7%)	0.058	1,560 (22.7%)	1,570 (22.8%)	0.003
South	3,851 (48.3%)	17,616 (45.6%)	0.053	3,327 (48.4%)	3,342 (48.6%)	0.004
West	1,016 (12.7%)	5,443 (14.1%)	0.04	894 (13.0%)	869 (12.6%)	0.011
Missing	4 (0.1%)	73 (0.2%)	0.042	4 (0.1%)	4 (0.1%)	0.000
Dual status/Low income subsidy	1,619 (20.3%)	11,484 (29.7%)	0.220	1,473 (21.4%)	1,494 (21.7%)	0.007
Lifestyle risk factors						
Smoking/Tobacco use	2,235 (28.0%)	10,680 (27.7%)	0.008	1,934 (28.1%)	1,929 (28.1%)	0.002
Weight						
Underweight	32 (0.4%)	810 (2.1%)	0.153	32 (0.5%)	25 (0.4%)	0.016
Normal weight	235 (2.9%)	3,876 (10.0%)	0.291	232 (3.4%)	219 (3.2%)	0.011
Overweight (BMI 25.0-29.9)	1,769 (22.2%)	12,521 (32.4%)	0.232	1,657 (24.1%)	1,675 (24.4%)	0.006
Class 1 Obesity (BMI 30.0-34.9)	599 (7.5%)	3,034 (7.9%)	0.013	542 (7.9%)	595 (8.7%)	0.028
Class 2 Obesity (BMI 35.0-39.9)	403 (5.1%)	1,429 (3.7%)	0.066	339 (4.9%)	333 (4.8%)	0.004
Class 3 Obesity; (BMI 40.0 and above)	2,126 (26.6%)	5,921 (15.3%)	0.280	1,698 (24.7%)	1,711 (24.9%)	0.004
Unspecified Obesity	2,816 (35.3%)	11,019 (28.5%)	0.145	2,377 (34.6%)	2,319 (33.7%)	0.018
Diabetes complications						
Diabetic retinopathy	996 (12.5%)	3,745 (9.7%)	0.089	793 (11.5%)	816 (11.9%)	0.010
Diabetic neuropathy	2,696 (33.8%)	10,973 (28.4%)	0.116	2,230 (32.4%)	2,259 (32.8%)	0.009
Diabetic nephropathy	2,403 (30.1%)	11,209 (29.0%)	0.024	2,000 (29.1%)	2,043 (29.7%)	0.014
Diabetes with other ophthalmic complications	206 (2.6%)	1,013 (2.6%)	0.003	173 (2.5%)	185 (2.7%)	0.011
Diabetes with peripheral circulatory disorders	1,074 (13.5%)	4,680 (12.1%)	0.040	879 (12.8%)	914 (13.3%)	0.015
Diabetic foot	259 (3.2%)	1,372 (3.6%)	0.017	208 (3.0%)	235 (3.4%)	0.022

Erectile dysfunction	185 (2.3%)	566 (1.5%)	0.063	147 (2.1%)	158 (2.3%)	0.011
Hypoglycemia	1,091 (13.7%)	4,937 (12.8%)	0.026	894 (13.0%)	897 (13.0%)	0.001
Hyperglycemia/DKA/HONK	4,461 (55.9%)	17,806 (46.1%)	0.197	3,656 (53.2%)	3,662 (53.2%)	0.002
Skin infections	950 (11.9%)	4,516 (11.7%)	0.006	791 (11.5%)	803 (11.7%)	0.005
Cardiovascular-related conditions						
Coronary atherosclerosis	3,616 (45.3%)	16,767 (43.4%)	0.038	3,067 (44.6%)	3,057 (44.5%)	0.003
Stable angina	632 (7.9%)	2,328 (6.0%)	0.074	511 (7.4%)	504 (7.3%)	0.004
Unstable angina	414 (5.2%)	1,629 (4.2%)	0.046	337 (4.9%)	345 (5.0%)	0.005
Hypertension	7,109 (89.1%)	34,600 (89.6%)	0.017	6,117 (88.9%)	6,134 (89.2%)	0.008
Hypotension	372 (4.7%)	2,368 (6.1%)	0.065	331 (4.8%)	329 (4.8%)	0.001
Hyperlipidemia	6,207 (77.8%)	29,388 (76.1%)	0.04	5,303 (77.1%)	5,308 (77.2%)	0.002
Acute MI	156 (2.0%)	731 (1.9%)	0.004	127 (1.8%)	129 (1.9%)	0.002
Old MI	730 (9.1%)	3,545 (9.2%)	0.001	618 (9.0%)	647 (9.4%)	0.015
Ischemic stroke	127 (1.6%)	860 (2.2%)	0.046	110 (1.6%)	116 (1.7%)	0.007
TIA	204 (2.6%)	1,496 (3.9%)	0.075	185 (2.7%)	181 (2.6%)	0.004
Cardiac conduction disorder	711 (8.9%)	3,952 (10.2%)	0.045	624 (9.1%)	582 (8.5%)	0.022
Previous cardiac procedure (CABG, PTCA, Stent)	368 (4.6%)	1,209 (3.1%)	0.077	287 (4.2%)	278 (4.0%)	0.007
PVD diagnosis or surgery	906 (11.4%)	4,988 (12.9%)	0.048	775 (11.3%)	810 (11.8%)	0.016
Atrial fibrillation	1,662 (20.8%)	8,887 (23.0%)	0.053	1,437 (20.9%)	1,454 (21.1%)	0.006
Other cardiac dysrhythmia	2,053 (25.7%)	11,191 (29.0%)	0.073	1,784 (25.9%)	1,767 (25.7%)	0.006
Heart failure	2,013 (25.2%)	10,263 (26.6%)	0.031	1,707 (24.8%)	1,705 (24.8%)	0.001
Acute heart failure	558 (7.0%)	3,384 (8.8%)	0.066	475 (6.9%)	463 (6.7%)	0.007
Cardiomyopathy	676 (8.5%)	3,287 (8.5%)	0.002	562 (8.2%)	560 (8.1%)	0.001
Valve disorders	1,290 (16.2%)	7,167 (18.6%)	0.063	1,120 (16.3%)	1,099 (16.0%)	0.008
Valve replacement	235 (2.9%)	1,157 (3.0%)	0.003	205 (3.0%)	194 (2.8%)	0.010
Edema	1,269 (15.9%)	6,118 (15.8%)	0.002	1,066 (15.5%)	1,099 (16.0%)	0.013
Venous thromboembolism / Pulmonary embolism	326 (4.1%)	1,758 (4.6%)	0.023	273 (4.0%)	285 (4.1%)	0.009
Pulmonary hypertension	349 (4.4%)	1,953 (5.1%)	0.032	292 (4.2%)	285 (4.1%)	0.005
Implantable cardioverter defibrillator	55 (0.7%)	283 (0.7%)	0.005	44 (0.6%)	42 (0.6%)	0.004
Hyperkalemia	131 (1.6%)	887 (2.3%)	0.047	110 (1.6%)	115 (1.7%)	0.006
Cerebrovascular procedure	27 (0.3%)	126 (0.3%)	0.002	24 (0.3%)	25 (0.4%)	0.002
Insertion of pacemakers / removal of cardiac lead	39 (0.5%)	238 (0.6%)	0.017	31 (0.5%)	35 (0.5%)	0.008
Renal-related conditions						
Hypertensive nephropathy	1,649 (20.7%)	8,274 (21.4%)	0.019	1,392 (20.2%)	1,392 (20.2%)	0.000
CKD stage 1-2	317 (4.0%)	1,530 (4.0%)	0.000	268 (3.9%)	277 (4.0%)	0.007
CKD stage 3-4	2,096 (26.3%)	9,602 (24.9%)	0.032	1,758 (25.6%)	1,754 (25.5%)	0.001
Unspecified CKD	809 (10.1%)	4,258 (11.0%)	0.029	681 (9.9%)	694 (10.1%)	0.006
Microalbuminuria or proteinuria	110 (1.4%)	544 (1.4%)	0.003	91 (1.3%)	98 (1.4%)	0.009
Acute kidney injury	845 (10.6%)	5,487 (14.2%)	0.110	733 (10.7%)	747 (10.9%)	0.007

Urinary tract infections	1,493 (18.7%)	9,285 (24.0%)	0.131	1,316 (19.1%)	1,338 (19.5%)	0.008
Genital infections	221 (2.8%)	1,085 (2.8%)	0.002	192 (2.8%)	210 (3.1%)	0.016
Urolithiasis (Kidney and urinary stone)	429 (5.4%)	1,997 (5.2%)	0.009	370 (5.4%)	347 (5.0%)	0.015
Other comorbidities						
COPD	1,489 (18.7%)	7,568 (19.6%)	0.024	1,286 (18.7%)	1,288 (18.7%)	0.001
Asthma	880 (11.0%)	3,741 (9.7%)	0.044	737 (10.7%)	763 (11.1%)	0.012
Obstructive sleep apnea	2,602 (32.6%)	7,779 (20.1%)	0.286	2,089 (30.4%)	2,050 (29.8%)	0.012
Serious bacterial infections	401 (5.0%)	3,058 (7.9%)	0.118	358 (5.2%)	379 (5.5%)	0.014
Pneumonia	570 (7.1%)	3,643 (9.4%)	0.083	500 (7.3%)	510 (7.4%)	0.006
Liver disease	905 (11.3%)	3,915 (10.1%)	0.039	753 (10.9%)	739 (10.7%)	0.007
MASH / MASLD	473 (5.9%)	1,656 (4.3%)	0.074	382 (5.6%)	363 (5.3%)	0.012
Fractures / Falls	317 (4.0%)	2,008 (5.2%)	0.059	273 (4.0%)	280 (4.1%)	0.005
Osteoporosis	499 (6.3%)	3,078 (8.0%)	0.067	445 (6.5%)	442 (6.4%)	0.002
Osteoarthritis	2,848 (35.7%)	13,310 (34.5%)	0.025	2,470 (35.9%)	2,445 (35.6%)	0.008
Depression	1,608 (20.2%)	7,295 (18.9%)	0.032	1,362 (19.8%)	1,385 (20.1%)	0.008
Dementia	440 (5.5%)	4,587 (11.9%)	0.227	400 (5.8%)	418 (6.1%)	0.011
Delirium or psychosis	108 (1.4%)	1,145 (3.0%)	0.111	95 (1.4%)	118 (1.7%)	0.027
Anxiety	1,192 (14.9%)	5,786 (15.0%)	0.001	1,041 (15.1%)	1,034 (15.0%)	0.003
Sleep disorders	2,785 (34.9%)	13,350 (34.6%)	0.007	2,387 (34.7%)	2,400 (34.9%)	0.004
Anemia	2,022 (25.3%)	11,829 (30.6%)	0.118	1,769 (25.7%)	1,786 (26.0%)	0.006
Influenza	173 (2.2%)	1,098 (2.8%)	0.043	156 (2.3%)	164 (2.4%)	0.008
COVID	57 (0.7%)	220 (0.6%)	0.018	51 (0.7%)	57 (0.8%)	0.010
Hyperthyroidism and other thyroid gland disorders	2,333 (29.2%)	10,684 (27.7%)	0.035	1,964 (28.6%)	1,993 (29.0%)	0.009
Hypothyroidism	1,923 (24.1%)	8,886 (23.0%)	0.026	1,621 (23.6%)	1,674 (24.3%)	0.018
Nephrotic syndrome	0 (0.0%)	3 (0.0%)	0.012	0 (0.0%)	0 (0.0%)	-
Urinary incontinence	477 (6.0%)	2,592 (6.7%)	0.030	410 (6.0%)	441 (6.4%)	0.019
Biliary disease	15 (0.2%)	164 (0.4%)	0.043	12 (0.2%)	14 (0.2%)	0.007
Pancreatitis	5 (0.1%)	31 (0.1%)	0.007	5 (0.1%)	7 (0.1%)	0.010
Bowel obstruction	18 (0.2%)	106 (0.3%)	0.010	11 (0.2%)	12 (0.2%)	0.004
Gastroparesis	55 (0.7%)	281 (0.7%)	0.005	49 (0.7%)	47 (0.7%)	0.003
Diabetes medications						
Number of antidiabetic drugs on CED; mean (SD) median [IQR]	2.23 ±0.86 2.00 [2.00, 3.00]	2.11 ±0.80 2.00 [2.00, 3.00]	0.145	2.20 ±0.86 2.00 [2.00, 3.00]	2.21 ±0.84 2.00 [2.00, 3.00]	0.012
Concomitant use or initiation of Metformin	3,806 (47.7%)	21,268 (55.1%)	0.148	3,432 (49.9%)	3,428 (49.8%)	0.001
Concomitant use or initiation of Insulins	2,728 (34.2%)	4,941 (12.8%)	0.521	1,926 (28.0%)	1,962 (28.5%)	0.012
Concomitant use or initiation of Sulfonylureas	1,882 (23.6%)	12,535 (32.5%)	0.199	1,768 (25.7%)	1,771 (25.8%)	0.001
Concomitant use or initiation of SGLT-2i	915 (11.5%)	1,892 (4.9%)	0.241	696 (10.1%)	716 (10.4%)	0.010
Concomitant use or initiation of Any other glucose-lowering drugs	478 (6.0%)	2,197 (5.7%)	0.013	415 (6.0%)	428 (6.2%)	0.008
Past use of Metformin	5,054 (63.3%)	24,716 (64.0%)	0.014	4,431 (64.4%)	4,449 (64.7%)	0.005
Past use of Insulins	3,636 (45.6%)	7,259 (18.8%)	0.598	2,639 (38.4%)	2,683 (39.0%)	0.013

Past use of Sulfonylureas	2,654 (33.3%)	15,926 (41.2%)	0.166	2,433 (35.4%)	2,447 (35.6%)	0.004
Past use of SGLT-2i	1,401 (17.6%)	2,973 (7.7%)	0.300	1,059 (15.4%)	1,064 (15.5%)	0.002
Past use of Any other glucose-lowering drugs	770 (9.6%)	3,320 (8.6%)	0.036	660 (9.6%)	694 (10.1%)	0.017
Other medications						
ACE / ARB	6,133 (76.9%)	28,677 (74.3%)	0.06	5,273 (76.7%)	5,308 (77.2%)	0.012
ARNI	151 (1.9%)	508 (1.3%)	0.046	123 (1.8%)	112 (1.6%)	0.012
Thiazides	2,732 (34.2%)	12,173 (31.5%)	0.058	2,334 (33.9%)	2,298 (33.4%)	0.011
Beta-blockers	5,126 (64.2%)	24,199 (62.7%)	0.032	4,357 (63.4%)	4,359 (63.4%)	0.001
Calcium channel blockers	3,011 (37.7%)	15,650 (40.5%)	0.057	2,625 (38.2%)	2,622 (38.1%)	0.001
Digoxin / Digitoxin	214 (2.7%)	1,378 (3.6%)	0.051	181 (2.6%)	176 (2.6%)	0.005
Loop diuretics	2,913 (36.5%)	12,958 (33.6%)	0.062	2,438 (35.5%)	2,406 (35.0%)	0.010
Other diuretics	1,061 (13.3%)	4,021 (10.4%)	0.089	842 (12.2%)	836 (12.2%)	0.003
Intravenous diuretics	224 (2.8%)	1,157 (3.0%)	0.011	187 (2.7%)	181 (2.6%)	0.005
Nitrates	1,280 (16.0%)	5,598 (14.5%)	0.043	1,059 (15.4%)	1,075 (15.6%)	0.006
Anti-arrhythmics	384 (4.8%)	1,995 (5.2%)	0.016	327 (4.8%)	324 (4.7%)	0.002
Statins	6,649 (83.3%)	30,909 (80.1%)	0.085	5,693 (82.8%)	5,668 (82.4%)	0.010
PCSK9 inhibitors and other lipid-lowering drugs	1,326 (16.6%)	5,321 (13.8%)	0.079	1,087 (15.8%)	1,089 (15.8%)	0.001
Antiplatelet medications	1,601 (20.1%)	7,466 (19.3%)	0.018	1,349 (19.6%)	1,380 (20.1%)	0.011
Oral anticoagulants	1,608 (20.2%)	8,073 (20.9%)	0.019	1,388 (20.2%)	1,418 (20.6%)	0.011
COPD / Asthma medications	3,068 (38.4%)	13,671 (35.4%)	0.063	2,595 (37.7%)	2,631 (38.3%)	0.011
NSAIDS	2,282 (28.6%)	10,567 (27.4%)	0.027	1,960 (28.5%)	1,998 (29.1%)	0.012
Oral corticosteroids	1,939 (24.3%)	8,875 (23.0%)	0.031	1,664 (24.2%)	1,719 (25.0%)	0.019
Osteoporosis agents (incl. bisphosphonates)	302 (3.8%)	1,831 (4.7%)	0.047	268 (3.9%)	267 (3.9%)	0.001
Opioids	3,124 (39.1%)	13,628 (35.3%)	0.080	2,651 (38.5%)	2,656 (38.6%)	0.001
Anti-depressants	3,395 (42.5%)	14,016 (36.3%)	0.128	2,836 (41.2%)	2,889 (42.0%)	0.016
Antipsychotics	306 (3.8%)	1,887 (4.9%)	0.052	266 (3.9%)	272 (4.0%)	0.005
Anxiolytics / hypnotics, benzos	1,747 (21.9%)	8,242 (21.3%)	0.013	1,499 (21.8%)	1,558 (22.7%)	0.021
Dementia medications	288 (3.6%)	2,660 (6.9%)	0.147	257 (3.7%)	272 (4.0%)	0.011
Urinary tract infections antibiotics	4,429 (55.5%)	20,605 (53.4%)	0.043	3,783 (55.0%)	3,802 (55.3%)	0.006
Laxatives	297 (3.7%)	2,065 (5.3%)	0.078	250 (3.6%)	279 (4.1%)	0.022
Healthcare utilization marker						
Number of distinct medications; mean (SD) median [IQR]	15.97 ±6.61 15.00 [11.00, 20.00]	14.57 ±6.59 14.00 [10.00, 18.00]	0.212	15.59 ±6.50 15.00 [11.00, 19.00]	15.81 ±6.77 15.00 [11.00, 20.00]	0.033
Number of office visits; mean (SD) median [IQR]	13.40 ±8.24 12.00 [8.00, 18.00]	11.74 ±7.88 10.00 [6.00, 16.00]	0.206	13.02 ±7.86 12.00 [7.00, 17.00]	13.06 ±8.54 11.00 [7.00, 17.00]	0.005
Number of endocrinologist visits; mean (SD) median [IQR]	0.89 ±1.97 0.00 [0.00, 1.00]	0.38 ±1.25 0.00 [0.00, 0.00]	0.308	0.70 ±1.63 0.00 [0.00, 1.00]	0.68 ±1.80 0.00 [0.00, 0.00]	0.011
Number of cardiologist visits; mean (SD) median [IQR]	2.36 ±3.75 1.00 [0.00, 3.00]	2.53 ±3.92 1.00 [0.00, 3.00]	0.042	2.35 ±3.79 1.00 [0.00, 3.00]	2.31 ±3.62 1.00 [0.00, 3.00]	0.012
Number of internal/family medicine visits; mean (SD) median [IQR]	6.41 ±6.39 5.00 [2.00, 9.00]	7.35 ±7.53 5.00 [3.00, 10.00]	0.135	6.48 ±6.43 5.00 [2.00, 9.00]	6.50 ±6.72 5.00 [2.00, 9.00]	0.004
Number of Electrocardiograms (ECG/EKG) ; mean (SD) median [IQR]	1.62 ±2.16 1.00 [0.00, 2.00]	1.81 ±2.37 1.00 [0.00, 2.00]	0.082	1.62 ±2.16 1.00 [0.00, 2.00]	1.60 ±2.10 1.00 [0.00, 2.00]	0.008
Number of Echocardiograms; mean (SD) median [IQR]	0.52 ±0.84 0.00 [0.00, 1.00]	0.57 ±0.89 0.00 [0.00, 1.00]	0.058	0.51 ±0.84 0.00 [0.00, 1.00]	0.51 ±0.83 0.00 [0.00, 1.00]	0.003

Out-of-pocket medication cost; mean (SD) median [IQR]	1,034.73 ±1,112.54 747.50 [228.42, 1,505.11]	659.81 ±798.91 424.62 [103.10, 920.09]	0.387	930.77 ±941.27 684.78 [204.44, 1,328.66]	938.92 ±1,041.14 689.81 [197.80, 1,337.36]	0.008
Unique brand medicines; mean (SD) median [IQR]	16.33 ±6.85 15.00 [12.00, 20.00]	14.86 ±6.81 14.00 [10.00, 19.00]	0.214	15.93 ±6.74 15.00 [11.00, 20.00]	16.16 ±7.03 15.00 [11.00, 20.00]	0.034
Unique generic medicines; mean (SD) median [IQR]	15.97 ±6.61 15.00 [11.00, 20.00]	14.57 ±6.59 14.00 [10.00, 18.00]	0.212	15.59 ±6.50 15.00 [11.00, 19.00]	15.81 ±6.77 15.00 [11.00, 20.00]	0.033
Ratio of brand to generic medications; mean (SD) median [IQR]	1.02 ±0.04 1.00 [1.00, 1.04]	1.02 ±0.04 1.00 [1.00, 1.00]	0.057	1.02 ±0.04 1.00 [1.00, 1.04]	1.02 ±0.04 1.00 [1.00, 1.03]	0.012
**Healthy behavior markers**						
Colonoscopy / Sigmoidoscopy	869 (10.9%)	3,665 (9.5%)	0.046	729 (10.6%)	721 (10.5%)	0.004
Flu Pneumococcal vaccine	794 (9.9%)	4,138 (10.7%)	0.025	678 (9.9%)	686 (10.0%)	0.004
Pap smear	349 (4.4%)	1,289 (3.3%)	0.054	304 (4.4%)	290 (4.2%)	0.010
PSA test	1,899 (23.8%)	7,758 (20.1%)	0.090	1,615 (23.5%)	1,604 (23.3%)	0.004
Fecal occult blood test	475 (6.0%)	2,836 (7.3%)	0.056	436 (6.3%)	409 (5.9%)	0.016
Bone mineral density tests	731 (9.2%)	3,152 (8.2%)	0.035	633 (9.2%)	621 (9.0%)	0.006
Mammograms	1,808 (22.7%)	6,892 (17.9%)	0.120	1,515 (22.0%)	1,489 (21.7%)	0.009
Telemedicine	1,390 (17.4%)	3,075 (8.0%)	0.287	1,099 (16.0%)	1,048 (15.2%)	0.020
**Laboratory and diagnostic tests**						
HbA1c tests; mean (SD) median [IQR]	2.94 ±2.04 3.00 [2.00, 4.00]	2.69 ±2.62 3.00 [2.00, 3.00]	0.109	2.89 ±2.01 3.00 [2.00, 4.00]	2.89 ±2.86 3.00 [2.00, 4.00]	0.001
Lipid panels; mean (SD) median [IQR]	1.95 ±1.74 2.00 [1.00, 3.00]	1.85 ±2.27 2.00 [1.00, 3.00]	0.053	1.93 ±1.70 2.00 [1.00, 3.00]	1.93 ±2.14 2.00 [1.00, 3.00]	0.004
Creatinine tests; mean (SD) median [IQR]	0.10 ±0.51 0.00 [0.00, 0.00]	0.11 ±0.78 0.00 [0.00, 0.00]	0.029	0.10 ±0.52 0.00 [0.00, 0.00]	0.09 ±0.49 0.00 [0.00, 0.00]	0.012
Natriuretic peptide tests; mean (SD) median [IQR]	0.41 ±1.75 0.00 [0.00, 0.00]	0.43 ±2.12 0.00 [0.00, 0.00]	0.008	0.40 ±1.75 0.00 [0.00, 0.00]	0.39 ±1.31 0.00 [0.00, 0.00]	0.008
Urine tests; mean (SD) median [IQR]	1.61 ±2.81 1.00 [0.00, 2.00]	1.76 ±3.02 1.00 [0.00, 2.00]	0.052	1.63 ±2.90 1.00 [0.00, 2.00]	1.64 ±2.55 1.00 [0.00, 2.00]	0.005
**Burden of comorbidities**						
Combined comorbidity score; mean (SD) median [IQR]	2.83 ±2.45 2.00 [1.00, 4.00]	3.12 ±2.81 3.00 [1.00, 5.00]	0.108	2.80 ±2.47 2.00 [1.00, 4.00]	2.83 ±2.52 2.00 [1.00, 4.00]	0.011
Frailty Score; mean (SD) median [IQR]	0.22 ±0.06 0.21 [0.17, 0.25]	0.23 ±0.08 0.21 [0.17, 0.27]	0.142	0.22 ±0.07 0.21 [0.17, 0.25]	0.22 ±0.07 0.21 [0.17, 0.25]	0.008
**Baseline hospitalizations and hospital metrics**						
Number of Hospitalizations; mean (SD) median [IQR]	0.38 ±0.82 0.00 [0.00, 1.00]	0.57 ±1.08 0.00 [0.00, 1.00]	0.196	0.39 ±0.83 0.00 [0.00, 1.00]	0.39 ±0.83 0.00 [0.00, 1.00]	0.002
Any hospitalization within prior 91 days	485 (6.1%)	5,234 (13.6%)	0.253	454 (6.6%)	467 (6.8%)	0.008
Any hospitalization within prior 92-365 days	1,762 (22.1%)	9,784 (25.3%)	0.077	1,518 (22.1%)	1,523 (22.1%)	0.002
Number of hospitalizations (0, 1, 2 or more)						
< 1	5,962 (74.7%)	25,976 (67.3%)	0.164	5,115 (74.4%)	5,106 (74.2%)	0.003
1 - <2	1,392 (17.4%)	7,564 (19.6%)	0.055	1,198 (17.4%)	1,219 (17.7%)	0.008
>= 2	626 (7.8%)	5,070 (13.1%)	0.173	564 (8.2%)	552 (8.0%)	0.006
Heart failure hospitalization	796 (10.0%)	5,064 (13.1%)	0.098	686 (10.0%)	686 (10.0%)	0.000
ED visit	3,466 (43.4%)	19,853 (51.4%)	0.160	3,016 (43.9%)	3,007 (43.7%)	0.003
**Calendar year of cohort entry**						
2018	571 (7.2%)	13,930 (36.1%)	0.750	566 (8.2%)	562 (8.2%)	0.002
2019	3,028 (37.9%)	13,697 (35.5%)	0.051	2,727 (39.7%)	2,764 (40.2%)	0.011
2020	4,381 (54.9%)	10,983 (28.4%)	0.557	3,584 (52.1%)	3,551 (51.6%)	0.010

Abbreviations: *not used in the propensity score; ACE, angiotensin-converting enzyme inhibitors; ARB, angiotensin receptor blocker; ARNI, angiotensin receptor/neprilysin inhibitor; BMI, body mass index; CABG, coronary artery bypass graft surgery; CED, cohort entry date; CKD, chronic kidney disease; COPD, chronic obstructive pulmonary disease; DKA, diabetic ketoacidosis; DPP4i, dipeptidyl peptidase-4 inhibitors; ED, emergency department; eGFR, estimated glomerular filtration rate (estimated using the quadratic GFR equation: GFR = EXP(1.911 + (5.249 / Serum creatinine) - (2.114 / (Serum creatinine^2)) - (0.00686 * Age) - 0.205 (if female))); HbA1c, hemoglobin A1c; HDL, high-density lipoprotein cholesterol; HONK, hyperglycemic hyperosmolar nonketotic state; IQR, inter-quartile-range; MASH, metabolic dysfunction associated steatohepatitis; MASLD, metabolic dysfunction associated steatotic liver disease; MI, myocardial infarction; N, number of participants; NSAIDs, non-steroidal anti-inflammatory drugs; PCSK9, proprotein convertase subtilisin/kexin type 9; PSA, prostate-specific antigen; PTCA, percutaneous transluminal coronary angioplasty; SGLT2i, sodium-glucose transport protein 2 inhibitors; SD, standard deviation; SMD, standardized mean difference; TIA, transient ischemic attack.
Missing data were handled by assuming absence of a code indicated absence of the condition for most binary covariates. Missing indicators were included for race and region in the propensity score model.
The Race-Others category includes Asian and Hispanic individuals.

Supplementary Table 6. Baseline characteristics of initiators of semaglutide vs sitagliptin when applying expanded eligibility criteria before and after propensity score matching, pooled across databases. Values are number (percentage) unless otherwise specified.

	Before propensity score matching			After propensity score matching		
Variable	Semaglutide (n = 262,044)	Sitagliptin (n = 191,157)	SMD	Semaglutide (n = 79,501)	Sitagliptin (n = 79,501)	SMD
Demographics						
Age; mean (SD)	59.52 ± 11.69	67.20 ± 11.56	0.668	63.28 ± 11.18	63.32 ± 11.89	0.003
Gender						
Male	116,668 (44.5%)	88,232 (46.2%)	0.033	36,976 (46.5%)	36,844 (46.3%)	0.003
Female	145,376 (55.5%)	102,925 (53.8%)	0.033	42,525 (53.5%)	42,657 (53.7%)	0.003
Race						
White	78,256 (48.6%)	77,019 (59.9%)	0.220	25,746 (55.6%)	25,740 (55.6%)	0.000
Black	20,514 (12.7%)	16,639 (12.9%)	0.032	6,321 (13.6%)	6,254 (13.5%)	0.003
Unknown / Missing	59,536 (36.9%)	30,695 (23.9%)	0.169	13,038 (28.2%)	13,114 (28.3%)	0.003
Others	2,869 (1.8%)	4,347 (3.4%)	0.092	1,207 (2.6%)	1,204 (2.6%)	0.000
Region / State						
Northeast	27,095 (10.3%)	30,255 (15.8%)	0.163	10,455 (13.2%)	10,520 (13.2%)	0.002
Midwest / North central	54,587 (20.8%)	38,886 (20.3%)	0.012	15,841 (19.9%)	15,989 (20.1%)	0.005
South	145,962 (55.7%)	95,922 (50.2%)	0.111	43,242 (54.4%)	43,187 (54.3%)	0.001
West	34,239 (13.1%)	25,852 (13.5%)	0.013	9,901 (12.5%)	9,742 (12.3%)	0.006
Missing	161 (0.1%)	242 (0.1%)	0.021	62 (0.1%)	63 (0.1%)	0.000
Dual status/Low income subsidy	3,108 (1.2%)	20,191 (10.6%)	0.407	2,843 (3.6%)	2,876 (3.6%)	0.002
Lifestyle risk factors						
Smoking/Tobacco use	49,846 (19.0%)	40,272 (21.1%)	0.051	15,188 (19.1%)	15,201 (19.1%)	0.000
Weight						
Underweight	88 (0.0%)	187 (0.1%)	0.025	44 (0.1%)	36 (0.0%)	0.004
Normal weight	700 (0.3%)	2,698 (1.4%)	0.126	491 (0.6%)	507 (0.6%)	0.003
Overweight (BMI 25.0-29.9)	25,399 (9.7%)	42,557 (22.3%)	0.348	13,016 (16.4%)	12,821 (16.1%)	0.007
Class 1 Obesity (BMI 30.0-34.9)	24,665 (9.4%)	25,832 (13.5%)	0.129	9,831 (12.4%)	9,751 (12.3%)	0.003
Class 2 Obesity (BMI 35.0-39.9)	18,264 (7.0%)	12,700 (6.6%)	0.013	5,791 (7.3%)	5,829 (7.3%)	0.002
Class 3 Obesity; (BMI 40.0 and above)	93,151 (35.5%)	41,839 (21.9%)	0.305	22,425 (28.2%)	22,492 (28.3%)	0.002
Unspecified Obesity	99,777 (38.1%)	65,344 (34.2%)	0.081	27,903 (35.1%)	28,065 (35.3%)	0.004
Diabetes complications						
Diabetic retinopathy	19,987 (7.6%)	17,246 (9.0%)	0.050	7,018 (8.8%)	6,895 (8.7%)	0.005
Diabetic neuropathy	57,699 (22.0%)	50,724 (26.5%)	0.105	20,103 (25.3%)	19,893 (25.0%)	0.006
Diabetic nephropathy	45,338 (17.3%)	43,924 (23.0%)	0.142	16,382 (20.6%)	16,090 (20.2%)	0.009
Diabetes with other ophthalmic complications	7,584 (2.9%)	6,747 (3.5%)	0.036	2,776 (3.5%)	2,701 (3.4%)	0.005
Diabetes with peripheral circulatory disorders	29,145 (11.1%)	23,930 (12.5%)	0.043	9,605 (12.1%)	9,583 (12.1%)	0.001
Diabetic foot	6,445 (2.5%)	5,826 (3.0%)	0.036	2,096 (2.6%)	2,073 (2.6%)	0.002

Erectile dysfunction	11,450 (4.4%)	6,113 (3.2%)	0.061	3,153 (4.0%)	3,084 (3.9%)	0.004
Hypoglycemia	53,382 (20.4%)	33,007 (17.3%)	0.079	15,124 (19.0%)	15,177 (19.1%)	0.002
Hyperglycemia/DKA/HONK	134,191 (51.2%)	96,004 (50.2%)	0.020	42,188 (53.1%)	41,857 (52.6%)	0.008
Skin infections	26,226 (10.0%)	20,475 (10.7%)	0.023	8,090 (10.2%)	8,056 (10.1%)	0.001
Cardiovascular-related conditions						
Coronary atherosclerosis	47,402 (18.1%)	46,394 (24.3%)	0.152	16,317 (20.5%)	16,130 (20.3%)	0.006
Stable angina	10,184 (3.9%)	8,317 (4.4%)	0.023	3,301 (4.2%)	3,251 (4.1%)	0.003
Unstable angina	5,855 (2.2%)	5,951 (3.1%)	0.054	2,046 (2.6%)	2,033 (2.6%)	0.001
Hypertension	223,037 (85.1%)	168,441 (88.1%)	0.088	68,720 (86.4%)	68,763 (86.5%)	0.002
Hypotension	6,266 (2.4%)	7,506 (3.9%)	0.088	2,336 (2.9%)	2,296 (2.9%)	0.003
Hyperlipidemia	214,688 (81.9%)	156,233 (81.7%)	0.005	65,752 (82.7%)	65,555 (82.5%)	0.007
Acute MI	2,866 (1.1%)	3,091 (1.6%)	0.045	1,008 (1.3%)	990 (1.2%)	0.002
Old MI	9,101 (3.5%)	9,896 (5.2%)	0.084	3,238 (4.1%)	3,192 (4.0%)	0.003
Ischemic stroke	2,261 (0.9%)	3,477 (1.8%)	0.083	909 (1.1%)	926 (1.2%)	0.002
TIA	3,528 (1.3%)	4,395 (2.3%)	0.071	1,243 (1.6%)	1,245 (1.6%)	0.000
Cardiac conduction disorder	12,054 (4.6%)	12,368 (6.5%)	0.082	4,154 (5.2%)	4,115 (5.2%)	0.002
Previous cardiac procedure (CABG, PTCA, Stent)	4,341 (1.7%)	3,993 (2.1%)	0.032	1,537 (1.9%)	1,467 (1.8%)	0.006
PVD diagnosis or surgery	18,880 (7.2%)	20,848 (10.9%)	0.129	7,128 (9.0%)	7,027 (8.8%)	0.004
Atrial fibrillation	21,406 (8.2%)	24,171 (12.6%)	0.147	7,657 (9.6%)	7,722 (9.7%)	0.003
Other cardiac dysrhythmia	46,916 (17.9%)	39,436 (20.6%)	0.069	14,397 (18.1%)	14,437 (18.2%)	0.001
Heart failure	28,435 (10.9%)	29,343 (15.4%)	0.134	9,656 (12.1%)	9,516 (12.0%)	0.005
Acute heart failure	7,119 (2.7%)	9,471 (5.0%)	0.117	2,614 (3.3%)	2,582 (3.2%)	0.002
Cardiomyopathy	11,196 (4.3%)	10,472 (5.5%)	0.056	3,661 (4.6%)	3,619 (4.6%)	0.003
Valve disorders	22,947 (8.8%)	22,686 (11.9%)	0.102	7,803 (9.8%)	7,812 (9.8%)	0.000
Valve replacement	2,463 (0.9%)	2,835 (1.5%)	0.050	915 (1.2%)	925 (1.2%)	0.001
Edema	30,466 (11.6%)	25,059 (13.1%)	0.045	9,313 (11.7%)	9,315 (11.7%)	0.000
Venous thromboembolism / Pulmonary embolism	7,629 (2.9%)	6,805 (3.6%)	0.037	2,405 (3.0%)	2,434 (3.1%)	0.002
Pulmonary hypertension	6,047 (2.3%)	6,398 (3.3%)	0.063	1,977 (2.5%)	2,009 (2.5%)	0.003
Implantable cardioverter defibrillator	594 (0.2%)	740 (0.4%)	0.029	234 (0.3%)	225 (0.3%)	0.002
Hyperkalemia	5,805 (2.2%)	5,351 (2.8%)	0.037	2,048 (2.6%)	2,028 (2.6%)	0.002
Cerebrovascular procedure	304 (0.1%)	442 (0.2%)	0.028	132 (0.2%)	130 (0.2%)	0.001
Insertion of pacemakers / removal of cardiac lead	321 (0.1%)	647 (0.3%)	0.045	149 (0.2%)	143 (0.2%)	0.002
Renal-related conditions						
Hypertensive nephropathy	25,386 (9.7%)	28,062 (14.7%)	0.153	9,474 (11.9%)	9,344 (11.8%)	0.005
CKD Stage 1-2	10,242 (3.9%)	9,405 (4.9%)	0.049	3,603 (4.5%)	3,549 (4.5%)	0.003
CKD Stage 3-4	29,878 (11.4%)	31,805 (16.6%)	0.151	11,173 (14.1%)	11,117 (14.0%)	0.002
Unspecified CKD	11,867 (4.5%)	14,054 (7.4%)	0.120	4,472 (5.6%)	4,462 (5.6%)	0.001
Microalbuminuria or proteinuria	13,795 (5.3%)	8,731 (4.6%)	0.032	3,911 (4.9%)	3,842 (4.8%)	0.004
Acute kidney injury	13,285 (5.1%)	18,088 (9.5%)	0.170	5,238 (6.6%)	5,236 (6.6%)	0.000

Urinary tract infections	30,997 (11.8%)	32,384 (16.9%)	0.146	10,771 (13.5%)	10,818 (13.6%)	0.002
Genital infections	10,189 (3.9%)	7,440 (3.9%)	0.000	3,395 (4.3%)	3,340 (4.2%)	0.003
Urolithiasis (Kidney and urinary stone)	11,373 (4.3%)	8,751 (4.6%)	0.012	3,510 (4.4%)	3,495 (4.4%)	0.001
**Other comorbidities**						
COPD	25,512 (9.7%)	26,130 (13.7%)	0.123	8,914 (11.2%)	8,911 (11.2%)	0.000
Asthma	29,978 (11.4%)	18,744 (9.8%)	0.053	8,083 (10.2%)	8,050 (10.1%)	0.001
Obstructive sleep apnea	82,955 (31.7%)	40,456 (21.2%)	0.240	20,290 (25.5%)	20,293 (25.5%)	0.000
Serious bacterial infections	7,247 (2.8%)	10,066 (5.3%)	0.128	2,798 (3.5%)	2,804 (3.5%)	0.000
Pneumonia	11,640 (4.4%)	12,486 (6.5%)	0.092	4,006 (5.0%)	4,048 (5.1%)	0.002
Liver disease	41,712 (15.9%)	25,498 (13.3%)	0.073	11,550 (14.5%)	11,473 (14.4%)	0.003
MASH / MASLD	25,516 (9.7%)	12,368 (6.5%)	0.120	6,375 (8.0%)	6,278 (7.9%)	0.005
Fractures / Falls	11,828 (4.5%)	10,413 (5.4%)	0.043	3,941 (5.0%)	3,945 (5.0%)	0.000
Osteoporosis	8,379 (3.2%)	9,884 (5.2%)	0.099	3,177 (4.0%)	3,221 (4.1%)	0.003
Osteoarthritis	69,446 (26.5%)	55,777 (29.2%)	0.060	21,376 (26.9%)	21,391 (26.9%)	0.000
Depression	54,548 (20.8%)	35,798 (18.7%)	0.052	15,261 (19.2%)	15,175 (19.1%)	0.003
Dementia	5,989 (2.3%)	12,624 (6.6%)	0.211	2,955 (3.7%)	2,909 (3.7%)	0.003
Delirium or psychosis	3,200 (1.2%)	4,221 (2.2%)	0.076	1,218 (1.5%)	1,205 (1.5%)	0.001
Anxiety	56,993 (21.7%)	30,099 (15.7%)	0.154	13,744 (17.3%)	13,792 (17.3%)	0.002
Sleep disorders	83,945 (32.0%)	59,984 (31.4%)	0.014	24,589 (30.9%)	24,449 (30.8%)	0.004
Anemia	43,553 (16.6%)	42,364 (22.2%)	0.141	14,705 (18.5%)	14,618 (18.4%)	0.003
Influenza	4,770 (1.8%)	4,245 (2.2%)	0.028	1,389 (1.7%)	1,393 (1.8%)	0.000
COVID	26,411 (10.1%)	6,446 (3.4%)	0.270	4,830 (6.1%)	5,015 (6.3%)	0.010
Hyperthyroidism and other thyroid gland disorders	64,190 (24.5%)	46,854 (24.5%)	0.000	19,154 (24.1%)	19,121 (24.1%)	0.001
Hypothyroidism	51,228 (19.5%)	38,044 (19.9%)	0.009	15,441 (19.4%)	15,392 (19.4%)	0.002
Nephrotic syndrome	221 (0.1%)	152 (0.1%)	0.002	59 (0.1%)	58 (0.1%)	0.000
Urinary incontinence	11,790 (4.5%)	10,356 (5.4%)	0.042	3,890 (4.9%)	3,811 (4.8%)	0.005
Biliary disease	488 (0.2%)	681 (0.4%)	0.033	200 (0.3%)	214 (0.3%)	0.003
Pancreatitis	286 (0.1%)	388 (0.2%)	0.024	115 (0.1%)	104 (0.1%)	0.004
Bowel obstruction	299 (0.1%)	365 (0.2%)	0.020	114 (0.1%)	115 (0.1%)	0.000
Gastroparesis	1,797 (0.7%)	1,502 (0.8%)	0.012	664 (0.8%)	617 (0.8%)	0.007
**Diabetes medications**						
Number of antidiabetic drugs on CED; mean (SD)	2.09 ± 0.91	2.21 ± 0.83	0.127	2.23 ± 0.90	2.22 ± 0.87	0.013
Concomitant use or initiation of Metformin	136,472 (52.1%)	119,637 (62.6%)	0.214	47,654 (59.9%)	47,488 (59.7%)	0.004
Concomitant use or initiation of Insulins	51,607 (19.7%)	25,390 (13.3%)	0.173	14,450 (18.2%)	14,038 (17.7%)	0.014
Concomitant use or initiation of Sulfonylureas	40,444 (15.4%)	55,817 (29.2%)	0.335	18,417 (23.2%)	18,366 (23.1%)	0.002
Concomitant use or initiation of SGLT-2i	43,173 (16.5%)	18,640 (9.8%)	0.200	12,234 (15.4%)	11,864 (14.9%)	0.013
Concomitant use or initiation of Any other glucose-lowering drugs	12,790 (4.9%)	12,012 (6.3%)	0.061	4,865 (6.1%)	4,835 (6.1%)	0.002
Past use of Metformin	180,070 (68.7%)	136,250 (71.3%)	0.056	57,422 (72.2%)	57,260 (72.0%)	0.005
Past use of Insulins	67,877 (25.9%)	35,331 (18.5%)	0.179	19,283 (24.3%)	18,833 (23.7%)	0.013

Past use of Sulfonylureas	57,929 (22.1%)	72,414 (37.9%)	0.349	24,974 (31.4%)	24,916 (31.3%)	0.002
Past use of SGLT-2i	58,736 (22.4%)	27,526 (14.4%)	0.208	17,101 (21.5%)	16,678 (21.0%)	0.013
Past use of Any other glucose-lowering drugs	18,411 (7.0%)	17,501 (9.2%)	0.078	7,051 (8.9%)	7,065 (8.9%)	0.001
Other medications						
ACE / ARB	188,997 (72.1%)	146,706 (76.7%)	0.106	59,870 (75.3%)	59,907 (75.4%)	0.001
ARNI	3,753 (1.4%)	1,800 (0.9%)	0.045	864 (1.1%)	861 (1.1%)	0.000
Thiazides	92,746 (35.4%)	66,557 (34.8%)	0.012	27,881 (35.1%)	27,961 (35.2%)	0.002
Beta-blockers	101,916 (38.9%)	88,720 (46.4%)	0.152	33,255 (41.8%)	33,333 (41.9%)	0.002
Calcium channel blockers	81,068 (30.9%)	68,805 (36.0%)	0.107	26,321 (33.1%)	26,403 (33.2%)	0.002
Digoxin / Digitoxin	1,521 (0.6%)	2,851 (1.5%)	0.090	721 (0.9%)	706 (0.9%)	0.002
Loop diuretics	42,465 (16.2%)	39,648 (20.7%)	0.117	13,878 (17.5%)	13,927 (17.5%)	0.002
Other diuretics	23,205 (8.9%)	15,422 (8.1%)	0.028	6,442 (8.1%)	6,421 (8.1%)	0.001
Intravenous diuretics	3,018 (1.2%)	3,205 (1.7%)	0.044	1,007 (1.3%)	1,001 (1.3%)	0.001
Nitrates	14,924 (5.7%)	15,532 (8.1%)	0.096	5,322 (6.7%)	5,234 (6.6%)	0.004
Anti-arrhythmics	5,346 (2.0%)	5,433 (2.8%)	0.052	1,800 (2.3%)	1,823 (2.3%)	0.002
Statins	201,165 (76.8%)	152,264 (79.7%)	0.070	63,368 (79.7%)	63,301 (79.6%)	0.002
PCSK9 inhibitors and other lipid-lowering drugs	33,320 (12.7%)	24,370 (12.7%)	0.001	10,492 (13.2%)	10,370 (13.0%)	0.005
Antiplatelet medications	25,440 (9.7%)	24,478 (12.8%)	0.098	8,847 (11.1%)	8,786 (11.1%)	0.002
Oral anticoagulants	23,597 (9.0%)	23,250 (12.2%)	0.103	8,007 (10.1%)	8,055 (10.1%)	0.002
COPD / Asthma medications	91,501 (34.9%)	62,744 (32.8%)	0.044	26,536 (33.4%)	26,463 (33.3%)	0.002
NSAIDS	89,120 (34.0%)	59,433 (31.1%)	0.062	26,168 (32.9%)	26,171 (32.9%)	0.000
Oral corticosteroids	67,992 (25.9%)	40,801 (21.3%)	0.108	17,735 (22.3%)	17,876 (22.5%)	0.004
Osteoporosis agents (incl. bisphosphonates)	5,414 (2.1%)	6,457 (3.4%)	0.081	2,085 (2.6%)	2,096 (2.6%)	0.001
Opioids	79,309 (30.3%)	60,807 (31.8%)	0.033	24,303 (30.6%)	24,100 (30.3%)	0.006
Anti-depressants	102,501 (39.1%)	61,553 (32.2%)	0.145	27,710 (34.9%)	27,612 (34.7%)	0.003
Antipsychotics	10,735 (4.1%)	7,563 (4.0%)	0.007	3,061 (3.9%)	2,966 (3.7%)	0.006
Anxiolytics / hypnotics, benzos	56,507 (21.6%)	35,934 (18.8%)	0.069	15,340 (19.3%)	15,472 (19.5%)	0.004
Dementia medications	2,589 (1.0%)	6,394 (3.3%)	0.162	1,417 (1.8%)	1,367 (1.7%)	0.005
Urinary tract infections antibiotics	121,257 (46.3%)	90,370 (47.3%)	0.020	36,563 (46.0%)	36,475 (45.9%)	0.002
Laxatives	8,804 (3.4%)	7,765 (4.1%)	0.037	2,571 (3.2%)	2,591 (3.3%)	0.001
Healthcare utilization marker						
Number of distinct medications; mean (SD)	14.18 ± 6.85	13.83 ± 6.69	0.055	14.04 ± 6.59	13.98 ± 6.83	0.009
Number of office visits; mean (SD)	9.46 ± 6.99	9.59 ± 7.26	0.018	9.44 ± 6.90	9.38 ± 7.24	0.008
Number of endocrinologist visits; mean (SD)	0.49 ± 1.42	0.33 ± 1.26	0.117	0.43 ± 1.20	0.41 ± 1.54	0.016
Number of cardiologist visits; mean (SD)	1.36 ± 3.25	1.74 ± 3.64	0.109	1.46 ± 3.37	1.45 ± 3.31	0.005
Number of internal/family medicine visits; mean (SD)	6.31 ± 7.33	7.20 ± 8.31	0.115	6.71 ± 7.90	6.65 ± 7.76	0.007
Number of electrocardiograms (ECG/EKG); mean (SD)	0.89 ± 1.60	1.22 ± 2.06	0.18	1.00 ± 1.74	1.00 ± 1.73	0.002
Number of echocardiograms; mean (SD)	0.26 ± 0.65	0.36 ± 0.77	0.136	0.29 ± 0.67	0.29 ± 0.68	0.000
Out-of-pocket medication cost; mean (SD)	574.49 ± 813.98	532.37 ± 711.75	0.054	566.85 ± 692.83	561.76 ± 787.43	0.007

Unique brand medicines; mean (SD)	14.48 ± 7.08	14.10 ± 6.93	0.052	14.32 ± 6.81	14.26 ± 7.07	0.009
Unique generic medicines; mean (SD)	14.18 ± 6.85	13.83 ± 6.69	0.055	14.04 ± 6.59	13.98 ± 6.83	0.009
Ratio of brand to generic medications; mean (SD)	1.02 ± 0.04	1.02 ± 0.04	0.000	1.02 ± 0.04	1.02 ± 0.04	0.000
Healthy behavior markers						
Colonoscopy / Sigmoidoscopy	27,619 (10.5%)	18,414 (9.6%)	0.030	7,905 (9.9%)	7,919 (10.0%)	0.001
Flu Pneumococcal vaccine	79,837 (30.5%)	53,460 (28.0%)	0.055	23,902 (30.1%)	23,969 (30.1%)	0.002
Pap smear	24,469 (9.3%)	11,003 (5.8%)	0.136	5,817 (7.3%)	5,878 (7.4%)	0.003
PSA test	54,862 (20.9%)	40,267 (21.1%)	0.003	17,161 (21.6%)	17,161 (21.6%)	0.000
Fecal occult blood test	11,095 (4.2%)	13,719 (7.2%)	0.127	4,498 (5.7%)	4,494 (5.7%)	0.000
Bone mineral density tests	15,450 (5.9%)	12,864 (6.7%)	0.034	5,064 (6.4%)	5,122 (6.4%)	0.003
Mammograms	69,472 (26.5%)	40,829 (21.4%)	0.121	19,056 (24.0%)	19,162 (24.1%)	0.003
Telemedicine	72,325 (27.6%)	24,741 (12.9%)	0.371	17,215 (21.7%)	17,097 (21.5%)	0.004
Laboratory and diagnostic tests						
HbA1c tests; mean (SD)	2.30 ± 1.44	2.40 ± 2.03	0.148	2.39 ± 1.50	2.38 ± 1.84	0.004
Lipid panels; mean (SD)	1.62 ± 1.26	1.67 ± 1.63	0.038	1.66 ± 1.32	1.66 ± 1.54	0.005
Creatinine tests; mean (SD)	1.63 ± 2.60	1.15 ± 2.44	0.189	1.38 ± 2.65	1.37 ± 2.67	0.002
Natriuretic peptide tests; mean (SD)	0.17 ± 0.89	0.23 ± 1.53	0.04	0.18 ± 0.95	0.19 ± 1.67	0.001
Urine tests; mean (SD)	1.02 ± 1.73	1.31 ± 2.55	0.135	1.15 ± 2.02	1.14 ± 2.32	0.002
Lab values						
HbA1c (%)*; mean (SD) median [IQR]	7.69 ±1.77 7.20 [6.40, 8.50]	8.01 ±1.66 7.70 [6.90, 8.80]	0.184	7.88 ±1.78 7.50 [6.60, 8.80]	8.04 ±1.66 7.70 [6.90, 8.80]	0.094
Glucose (mg/dl)*; mean (SD) median [IQR]	159.09 ±70.38 140.00 [112.00, 185.00]	171.69 ±71.28 156.00 [125.00, 199.00]	0.178	165.78 ±72.80 148.00 [117.00, 195.00]	171.64 ±71.59 156.00 [125.00, 199.00]	0.081
Creatinine (mg/dl)*; mean (SD) median [IQR]	1.01 ±0.93 0.90 [0.80, 1.09]	1.09 ±1.60 0.95 [0.80, 1.19]	0.054	1.06 ±1.37 0.93 [0.80, 1.15]	1.07 ±1.64 0.93 [0.80, 1.16]	0.008
Systolic blood pressure (mmHg)*; mean (SD) median [IQR]	129.84 ±19.63 130.00 [120.00, 140.00]	131.45 ±18.92 130.00 [121.00, 140.00]	0.083	130.99 ±18.36 130.00 [121.00, 140.00]	130.94 ±19.11 130.00 [121.00, 140.00]	0.003
Heart rate (1/min)*; mean (SD) median [IQR]	78.56 ±49.82 77.00 [69.00, 87.00]	77.87 ±16.26 77.00 [68.00, 86.00]	0.018	77.62 ±17.24 76.00 [68.00, 86.00]	78.02 ±16.84 77.00 [68.00, 86.00]	0.023
BMI (kg/m2)*; mean (SD) median [IQR]	37.93 ±7.77 36.61 [32.50, 41.83]	33.85 ±6.56 32.50 [29.12, 37.16]	0.566	35.65 ±7.22 34.30 [30.45, 39.27]	34.45 ±6.72 33.19 [29.64, 37.87]	0.172
eGFR (ml/min/1.73m2)*; mean (SD) median [IQR]	1,591.66 ±119,111.34 80.00 [62.00, 96.00]	629.87 ±52,913.02 71.00 [54.00, 89.00]	0.010	1,293.41 ±110,565.65 72.00 [55.00, 89.00]	688.51 ±55,585.66 72.00 [54.00, 89.00]	0.007
LDL (mg/dl)*; mean (SD) median [IQR]	86.48 ±39.37 82.80 [61.00, 109.00]	83.58 ±37.58 79.00 [60.00, 104.00]	0.075	82.82 ±38.25 78.00 [59.00, 103.00]	83.91 ±37.73 79.00 [60.00, 104.00]	0.029
HDL (mg/dl)*; mean (SD) median [IQR]	46.08 ±13.99 45.00 [37.00, 53.00]	46.35 ±13.64 45.00 [37.00, 54.00]	0.019	46.44 ±14.08 45.00 [37.00, 54.00]	45.78 ±13.48 44.00 [37.00, 53.00]	0.048
Total cholesterol (mg/dl)*; mean (SD) median [IQR]	165.41 ±48.78 161.00 [135.00, 192.00]	163.03 ±46.09 158.00 [133.00, 187.00]	0.050	161.73 ±46.70 156.00 [132.00, 187.00]	162.19 ±47.32 157.00 [132.00, 187.00]	0.010
Triglyceride (mg/dl)*; mean (SD) median [IQR]	177.55 ±160.58 145.00 [104.00, 207.00]	170.21 ±128.81 143.00 [103.00, 202.00]	0.050	172.44 ±130.39 144.00 [103.00, 204.00]	172.59 ±139.71 144.00 [104.00, 204.00]	0.001
Burden of comorbidities						
Combined comorbidity score; mean (SD)	1.63 ± 2.21	2.22 ± 2.70	0.239	1.85 ± 2.39	1.84 ± 2.37	0.007
Frailty Score; mean (SD)	0.16 ± 0.06	0.18 ± 0.07	0.308	0.17 ± 0.06	0.17 ± 0.06	0.000

**Baseline hospitalizations and hospital metrics**						
Number of Hospitalizations; mean (SD)	2.03 ± 16.69	2.42 ± 16.40	0.023	2.54 ± 21.95	2.42 ± 17.31	0.006
Any hospitalization within prior 91 days	10,410 (4.0%)	18,373 (9.6%)	0.226	4,618 (5.8%)	4,509 (5.7%)	0.006
Any hospitalization within prior 92-365 days	24,361 (9.3%)	28,747 (15.0%)	0.176	8,809 (11.1%)	8,761 (11.0%)	0.002
Number of hospitalizations (0, 1, 2 or more)						
< 1	230,747 (88.1%)	151,450 (79.2%)	0.240	67,765 (85.2%)	67,846 (85.3%)	0.003
1 - <2	16,197 (6.2%)	19,370 (10.1%)	0.145	5,640 (7.1%)	5,641 (7.1%)	0.000
>= 2	15,100 (5.8%)	20,337 (10.6%)	0.178	6,096 (7.7%)	6,014 (7.6%)	0.004
Heart failure hospitalization	8,237 (3.1%)	13,106 (6.9%)	0.171	3,329 (4.2%)	3,275 (4.1%)	0.003
ED visit	77,106 (29.4%)	74,660 (39.1%)	0.204	26,726 (33.6%)	26,693 (33.6%)	0.001
**Calendar year of cohort entry**						
2018	3,582 (1.4%)	52,558 (27.5%)	0.801	3,519 (4.4%)	3,574 (4.5%)	0.003
2019	20,023 (7.6%)	48,754 (25.5%)	0.495	15,815 (19.9%)	15,567 (19.6%)	0.008
2020	26,488 (10.1%)	38,483 (20.1%)	0.283	17,861 (22.5%)	17,742 (22.3%)	0.004
2021	30,171 (11.5%)	18,825 (9.8%)	0.054	13,322 (16.8%)	13,268 (16.7%)	0.002
2022	47,035 (17.9%)	14,196 (7.4%)	0.320	11,982 (15.1%)	12,115 (15.2%)	0.005
2023	86,077 (32.8%)	11,564 (6.0%)	0.720	10,736 (13.5%)	10,879 (13.7%)	0.005
2024	47,352 (18.1%)	6,742 (3.5%)	0.482	6,225 (7.8%)	6,323 (8.0%)	0.005
2025	1,316 (0.5%)	35 (0.0%)	0.095	41 (0.1%)	33 (0.0%)	0.005

Abbreviations: *not used in the propensity score; ACE, angiotensin-converting enzyme inhibitors; ARB, angiotensin receptor blocker; ARNI, angiotensin receptor/neprilysin inhibitor; BMI, body mass index; CABG, coronary artery bypass graft surgery; CED, cohort entry date; CKD, chronic kidney disease; COPD, chronic obstructive pulmonary disease; DKA, diabetic ketoacidosis; DPP4i, dipeptidyl peptidase-4 inhibitors; ED, emergency department; eGFR, estimated glomerular filtration rate (estimated using the quadratic GFR equation: GFR = EXP(1.911 + (5.249 / Serum creatinine) - (2.114 / (Serum creatinine^2)) - (0.00686 * Age) - 0.205 (if female))); HbA1c, hemoglobin A1c; HDL, high-density lipoprotein cholesterol; HONK, hyperglycemic hyperosmolar nonketotic state; IQR, inter-quartile-range; MASH, metabolic dysfunction associated steatohepatitis; MASLD, metabolic dysfunction associated steatotic liver disease; MI, myocardial infarction; N, number of participants; NSAIDs, non-steroidal anti-inflammatory drugs; PCSK9, proprotein convertase subtilisin/kexin type 9; PSA, prostate-specific antigen; PTCA, percutaneous transluminal coronary angioplasty; SGLT2i, sodium-glucose transport protein 2 inhibitors; SD, standard deviation; SMD, standardized mean difference; TIA, transient ischemic attack.

Missing data were handled by assuming absence of a code indicated absence of the condition for most binary covariates. Missing indicators were included for race and region in the propensity score model.

Laboratory values were only available in a subset of the Optum database and were truncated using clinically plausible cut-off values (BMI values >100 or <10 were set to missing; missingness before matching ~63%. Creatinine values >30 were set to missing, and values <0.8 were set to 0.8; missingness before matching ~41%. eGFR values >150 were set to 150, and values =0 were set to missing; missingness before matching ~71%. Glucose values <30 were set to missing; missingness before matching ~41%. HbA1c values ≥20 or ≤2 were set to missing; missingness before matching ~37%. HDL value missingness before matching was ~48%. Heart rate values <30 were set to missing; missingness before matching ~75%. LDL value missingness before matching was~46%. Systolic blood pressure values <30 were set to missing; missingness before matching ~61%. Total cholesterol value missingness before matching was ~48%. Triglyceride value missingness before matching was ~49%).

Dual status/Low income subsidy was only available in Medicare database. Race was reported by the respective database: Medicare and Optum. The Race-Others category includes Asian and Hispanic individuals.

Supplementary Table 7. Baseline characteristics of initiators of semaglutide vs sitagliptin when applying trial eligibility criteria before and after propensity score matching, Optum database. Values are number (percentage) unless otherwise specified.

	Before propensity score matching			After propensity score matching		
Variable	Semaglutide (n = 146,540)	Sitagliptin (n = 65,509)	SMD	Semaglutide (n = 33,973)	Sitagliptin (n = 33,973)	SMD
Demographics						
Age; mean (SD) median [IQR]	62.35 ±11.30 65.00 [55.00, 70.00]	69.57 ±9.36 70.00 [66.00, 76.00]	0.697	67.78 ±9.32 69.00 [63.00, 74.00]	67.89 ±9.61 69.00 [64.00, 74.00]	0.012
Gender						
Male	64,467 (44.0%)	29,099 (44.4%)	0.009	14,960 (44.0%)	14,946 (44.0%)	0.001
Female	82,073 (56,0%)	36,410 (55,6%)	0.009	19,013 (56,0%)	19,027 (56,0%)	0.001
Race						
White	66,030 (45.1%)	28,948 (44.2%)	0.018	15,545 (45.8%)	15,554 (45.8%)	0.001
Black	19,891 (13.6%)	11,899 (18.2%)	0.126	5,757 (16.9%)	5,684 (16.7%)	0.006
Unknown / Missing	58,262 (39.8%)	22,514 (34.4%)	0.112	11,895 (35.0%)	11,962 (35.2%)	0.004
Others	2,357 (1.6%)	2,148 (3.3%)	0.108	776 (2.3%)	773 (2.3%)	0.001
Region / State						
Northeast	12,799 (8.7%)	7,242 (11.1%)	0.078	3,569 (10.5%)	3,605 (10.6%)	0.003
Midwest / North central	30,398 (20.7%)	9,310 (14.2%)	0.173	5,604 (16.5%)	5,673 (16.7%)	0.005
South	80,054 (54.6%)	37,213 (56.8%)	0.044	19,152 (56.4%)	19,073 (56.1%)	0.005
West	23,230 (15.9%)	11,710 (17.9%)	0.054	5,632 (16.6%)	5,608 (16.5%)	0.002
Missing; n (%)	59 (0.0%)	34 (0.1%)	0.005	16 (0.0%)	14 (0.0%)	0.003
Lifestyle risk factors						
Smoking/Tobacco use	35,849 (24.5%)	16,044 (24.5%)	0.001	8,395 (24.7%)	8,376 (24.7%)	0.001
Weight						
Underweight	41 (0.0%)	64 (0.1%)	0.028	15 (0.0%)	13 (0.0%)	0.003
Normal weight	424 (0.3%)	1,170 (1.8%)	0.148	270 (0.8%)	280 (0.8%)	0.003
Overweight (BMI 25.0-29.9)	15,116 (10.3%)	17,125 (26.1%)	0.419	6,789 (20.0%)	6,744 (19.9%)	0.003
Class 1 Obesity (BMI 30.0-34.9)	14,784 (10.1%)	10,018 (15.3%)	0.157	4,777 (14.1%)	4,756 (14.0%)	0.002
Class 2 Obesity (BMI 35.0-39.9)	10,306 (7.0%)	4,424 (6.8%)	0.011	2,409 (7.1%)	2,439 (7.2%)	0.003
Class 3 Obesity; (BMI 40.0 and above)	58,436 (39.9%)	14,046 (21.4%)	0.408	9,132 (26.9%)	9,206 (27.1%)	0.005
Unspecified Obesity	47,433 (32.4%)	18,662 (28.5%)	0.084	10,581 (31.1%)	10,535 (31.0%)	0.003
Diabetes complications						
Diabetic retinopathy	12,474 (8.5%)	7,021 (10.7%)	0.075	3,514 (10.3%)	3,457 (10.2%)	0.006
Diabetic neuropathy	38,182 (26.1%)	21,697 (33.1%)	0.155	10,890 (32.1%)	10,697 (31.5%)	0.012
Diabetic nephropathy	30,714 (21.0%)	19,291 (29.4%)	0.196	9,148 (26.9%)	9,049 (26.6%)	0.007
Diabetes with other ophthalmic complications	5,844 (4.0%)	4,010 (6.1%)	0.097	1,948 (5.7%)	1,886 (5.6%)	0.008
Diabetes with peripheral circulatory disorders	20,462 (14.0%)	11,814 (18.0%)	0.111	5,878 (17.3%)	5,882 (17.3%)	0.000
Diabetic foot	4,359 (3.0%)	2,161 (3.3%)	0.019	1,123 (3.3%)	1,088 (3.2%)	0.006

Erectile dysfunction	7,345 (5.0%)	2,631 (4.0%)	0.048	1,531 (4.5%)	1,492 (4.4%)	0.006
Hypoglycemia	35,046 (23.9%)	16,711 (25.5%)	0.037	8,717 (25.7%)	8,722 (25.7%)	0.0
Hyperglycemia/DKA/HONK	75,069 (51.2%)	34,625 (52.9%)	0.033	18,118 (53.3%)	18,004 (53.0%)	0.007
Skin infections	15,469 (10.6%)	6,799 (10.4%)	0.006	3,601 (10.6%)	3,544 (10.4%)	0.005
Cardiovascular-related conditions						
Coronary atherosclerosis	31,160 (21.3%)	16,289 (24.9%)	0.086	8,157 (24.0%)	8,131 (23.9%)	0.002
Stable angina	7,012 (4.8%)	3,404 (5.2%)	0.019	1,758 (5.2%)	1,718 (5.1%)	0.005
Unstable angina	3,596 (2.5%)	1,961 (3.0%)	0.033	943 (2.8%)	926 (2.7%)	0.003
Hypertension	129,348 (88.3%)	60,446 (92.3%)	0.135	30,983 (91.2%)	31,001 (91.3%)	0.002
Hypotension	4,294 (2.9%)	2,748 (4.2%)	0.068	1,293 (3.8%)	1,255 (3.7%)	0.006
Hyperlipidemia	124,937 (85.3%)	57,587 (87.9%)	0.078	29,853 (87.9%)	29,727 (87.5%)	0.011
Acute MI	1,628 (1.1%)	877 (1.3%)	0.021	419 (1.2%)	406 (1.2%)	0.003
Old MI	6,389 (4.4%)	3,762 (5.7%)	0.063	1,729 (5.1%)	1,756 (5.2%)	0.004
Ischemic stroke	978 (0.7%)	719 (1.1%)	0.046	313 (0.9%)	298 (0.9%)	0.005
TIA	2,479 (1.7%)	1,592 (2.4%)	0.052	677 (2.0%)	678 (2.0%)	0.000
Cardiac conduction disorder	8,245 (5.6%)	4,498 (6.9%)	0.051	2,231 (6.6%)	2,229 (6.6%)	0.000
Previous cardiac procedure (CABG, PTCA, Stent)	2,505 (1.7%)	1,178 (1.8%)	0.007	621 (1.8%)	578 (1.7%)	0.010
PVD diagnosis or surgery	13,697 (9.3%)	9,099 (13.9%)	0.142	4,271 (12.6%)	4,208 (12.4%)	0.006
Atrial fibrillation	14,252 (9.7%)	7,859 (12.0%)	0.073	3,849 (11.3%)	3,897 (11.5%)	0.004
Other cardiac dysrhythmia	29,604 (20.2%)	14,125 (21.6%)	0.033	7,175 (21.1%)	7,164 (21.1%)	0.001
Heart failure	19,955 (13.6%)	10,742 (16.4%)	0.078	5,254 (15.5%)	5,219 (15.4%)	0.003
Acute heart failure	4,816 (3.3%)	2,954 (4.5%)	0.063	1,319 (3.9%)	1,334 (3.9%)	0.002
Cardiomyopathy	7,488 (5.1%)	4,004 (6.1%)	0.044	1,877 (5.5%)	1,919 (5.6%)	0.005
Valve disorders	15,354 (10.5%)	8,567 (13.1%)	0.081	4,186 (12.3%)	4,202 (12.4%)	0.001
Valve replacement	1,667 (1.1%)	951 (1.5%)	0.028	465 (1.4%)	474 (1.4%)	0.002
Edema	20,415 (13.9%)	9,890 (15.1%)	0.033	5,020 (14.8%)	5,007 (14.7%)	0.001
Venous thromboembolism / Pulmonary embolism	4,777 (3.3%)	2,376 (3.6%)	0.020	1,172 (3.4%)	1,191 (3.5%)	0.003
Pulmonary hypertension	4,393 (3.0%)	2,449 (3.7%)	0.041	1,135 (3.3%)	1,163 (3.4%)	0.005
Implantable cardioverter defibrillator	349 (0.2%)	239 (0.4%)	0.023	103 (0.3%)	107 (0.3%)	0.002
Hyperkalemia	4,343 (3.0%)	2,982 (4.6%)	0.084	1,352 (4.0%)	1,356 (4.0%)	0.001
Cerebrovascular procedure	202 (0.1%)	125 (0.2%)	0.013	66 (0.2%)	59 (0.2%)	0.005
Insertion of pacemakers / removal of cardiac lead	225 (0.2%)	207 (0.3%)	0.034	87 (0.3%)	78 (0.2%)	0.005
Renal-related conditions						
Hypertensive nephropathy	18,427 (12.6%)	11,888 (18.1%)	0.155	5,615 (16.5%)	5,562 (16.4%)	0.004
CKD Stage 1-2	7,718 (5.3%)	5,345 (8.2%)	0.116	2,427 (7.1%)	2,389 (7.0%)	0.004
CKD Stage 3-4	22,030 (15.0%)	14,299 (21.8%)	0.176	6,744 (19.9%)	6,729 (19.8%)	0.001
Unspecified CKD	8,740 (6.0%)	5,698 (8.7%)	0.105	2,656 (7.8%)	2,663 (7.8%)	0.001
Microalbuminuria or proteinuria	9,587 (6.5%)	4,267 (6.5%)	0.001	2,198 (6.5%)	2,173 (6.4%)	0.003
Acute kidney injury	8,885 (6.1%)	6,159 (9.4%)	0.125	2,767 (8.1%)	2,733 (8.0%)	0.004

Urinary tract infections	19,129 (13.1%)	11,376 (17.4%)	0.120	5,302 (15.6%)	5,305 (15.6%)	0.000
Genital infections	5,201 (3.5%)	2,508 (3.8%)	0.015	1,338 (3.9%)	1,302 (3.8%)	0.005
Urolithiasis (Kidney and urinary stone)	6,716 (4.6%)	2,947 (4.5%)	0.004	1,569 (4.6%)	1,557 (4.6%)	0.002
Other comorbidities						
COPD	18,765 (12.8%)	10,525 (16.1%)	0.093	5,232 (15.4%)	5,173 (15.2%)	0.005
Asthma	18,901 (12.9%)	7,158 (10.9%)	0.061	4,037 (11.9%)	3,994 (11.8%)	0.004
Obstructive sleep apnea	48,510 (33.1%)	13,320 (20.3%)	0.292	8,479 (25.0%)	8,413 (24.8%)	0.004
Serious bacterial infections	3,924 (2.7%)	2,706 (4.1%)	0.080	1,159 (3.4%)	1,171 (3.4%)	0.002
Pneumonia	7,210 (4.9%)	4,213 (6.4%)	0.065	1,953 (5.7%)	1,985 (5.8%)	0.004
Liver disease	26,199 (17.9%)	11,081 (16.9%)	0.025	5,929 (17.5%)	5,925 (17.4%)	0.000
MASH / MASLD	15,221 (10.4%)	4,960 (7.6%)	0.099	2,970 (8.7%)	2,911 (8.6%)	0.006
Fractures / Falls	8,413 (5.7%)	5,129 (7.8%)	0.083	2,455 (7.2%)	2,459 (7.2%)	0.000
Osteoporosis	6,308 (4.3%)	4,315 (6.6%)	0.101	1,969 (5.8%)	1,992 (5.9%)	0.003
Osteoarthritis	45,336 (30.9%)	21,532 (32.9%)	0.041	11,056 (32.5%)	11,097 (32.7%)	0.003
Depression	34,185 (23.3%)	14,524 (22.2%)	0.028	7,715 (22.7%)	7,631 (22.5%)	0.006
Dementia	4,391 (3.0%)	4,845 (7.4%)	0.199	1,886 (5.6%)	1,820 (5.4%)	0.009
Delirium or psychosis	2,406 (1.6%)	1,755 (2.7%)	0.071	803 (2.4%)	761 (2.2%)	0.008
Anxiety	34,402 (23.5%)	11,583 (17.7%)	0.144	6,608 (19.5%)	6,566 (19.3%)	0.003
Sleep disorders	53,052 (36.2%)	23,188 (35.4%)	0.017	12,209 (35.9%)	12,067 (35.5%)	0.009
Anemia	26,540 (18.1%)	15,037 (23.0%)	0.120	7,256 (21.4%)	7,203 (21.2%)	0.004
Influenza	2,568 (1.8%)	1,131 (1.7%)	0.002	498 (1.5%)	502 (1.5%)	0.001
COVID	14,783 (10.1%)	3,375 (5.2%)	0.187	2,480 (7.3%)	2,593 (7.6%)	0.013
Hyperthyroidism and other thyroid gland disorders	38,345 (26.2%)	17,304 (26.4%)	0.006	8,996 (26.5%)	8,954 (26.4%)	0.003
Hypothyroidism	31,043 (21.2%)	14,224 (21.7%)	0.013	7,357 (21.7%)	7,358 (21.7%)	0.000
Nephrotic syndrome	140 (0.1%)	57 (0.1%)	0.003	24 (0.1%)	25 (0.1%)	0.001
Urinary incontinence	8,269 (5.6%)	4,393 (6.7%)	0.044	2,308 (6.8%)	2,219 (6.5%)	0.011
Biliary disease	201 (0.1%)	164 (0.3%)	0.026	68 (0.2%)	81 (0.2%)	0.008
Pancreatitis	147 (0.1%)	107 (0.2%)	0.017	39 (0.1%)	42 (0.1%)	0.003
Bowel obstruction	149 (0.1%)	99 (0.2%)	0.014	47 (0.1%)	46 (0.1%)	0.001
Gastroparesis	1,193 (0.8%)	639 (1.0%)	0.017	375 (1.1%)	344 (1.0%)	0.009
Diabetes medications						
Number of antidiabetic drugs on CED; mean (SD) median [IQR]	2.08 ±0.90 2.00 [1.00, 3.00]	2.24 ±0.85 2.00 [2.00, 3.00]	0.184	2.24 ±0.91 2.00 [2.00, 3.00]	2.22 ±0.87 2.00 [2.00, 3.00]	0.022
Concomitant use or initiation of Metformin	74,154 (50.6%)	40,064 (61.2%)	0.214	19,850 (58.4%)	19,703 (58.0%)	0.009
Concomitant use or initiation of Insulins	28,757 (19.6%)	8,830 (13.5%)	0.166	5,752 (16.9%)	5,552 (16.3%)	0.016
Concomitant use or initiation of Sulfonylureas	23,766 (16.2%)	20,225 (30.9%)	0.351	8,839 (26.0%)	8,739 (25.7%)	0.007
Concomitant use or initiation of SGLT-2i	23,236 (15.9%)	6,999 (10.7%)	0.153	5,063 (14.9%)	4,884 (14.4%)	0.015
Concomitant use or initiation of Any other glucose-lowering drugs	8,009 (5.5%)	4,817 (7.4%)	0.077	2,458 (7.2%)	2,424 (7.1%)	0.004
Past use of Metformin	97,987 (66.9%)	46,144 (70.4%)	0.077	23,832 (70.1%)	23,689 (69.7%)	0.009
Past use of Insulins	37,726 (25.7%)	12,255 (18.7%)	0.170	7,722 (22.7%)	7,505 (22.1%)	0.015

Past use of Sulfonylureas	33,839 (23.1%)	26,215 (40.0%)	0.370	11,803 (34.7%)	11,663 (34.3%)	0.009
Past use of SGLT-2i	32,183 (22.0%)	10,505 (16.0%)	0.151	7,322 (21.6%)	7,109 (20.9%)	0.015
Past use of Any other glucose-lowering drugs	11,263 (7.7%)	6,939 (10.6%)	0.101	3,468 (10.2%)	3,442 (10.1%)	0.003
Other medications						
ACE / ARB	106,815 (72.9%)	51,282 (78.3%)	0.126	26,037 (76.6%)	26,030 (76.6%)	0.000
ARNI	2,511 (1.7%)	770 (1.2%)	0.045	462 (1.4%)	486 (1.4%)	0.006
Thiazides	51,553 (35.2%)	23,234 (35.5%)	0.006	11,993 (35.3%)	12,037 (35.4%)	0.003
Beta-blockers	61,361 (41.9%)	31,100 (47.5%)	0.113	15,569 (45.8%)	15,686 (46.2%)	0.007
Calcium channel blockers	47,975 (32.7%)	24,798 (37.9%)	0.107	12,433 (36.6%)	12,414 (36.5%)	0.001
Digoxin / Digitoxin	903 (0.6%)	818 (1.2%)	0.066	323 (1.0%)	325 (1.0%)	0.001
Loop diuretics	27,529 (18.8%)	13,192 (20.1%)	0.034	6,761 (19.9%)	6,782 (20.0%)	0.002
Other diuretics	13,715 (9.4%)	5,134 (7.8%)	0.054	2,857 (8.4%)	2,871 (8.5%)	0.001
Intravenous diuretics	2,069 (1.4%)	1,105 (1.7%)	0.022	542 (1.6%)	536 (1.6%)	0.001
Nitrates	9,447 (6.4%)	5,044 (7.7%)	0.049	2,501 (7.4%)	2,426 (7.1%)	0.009
Anti-arrhythmics	3,413 (2.3%)	1,631 (2.5%)	0.010	839 (2.5%)	851 (2.5%)	0.002
Statins	116,370 (79.4%)	54,445 (83.1%)	0.095	28,402 (83.6%)	28,349 (83.4%)	0.004
PCSK9 inhibitors and other lipid-lowering drugs	19,421 (13.3%)	8,155 (12.4%)	0.024	4,569 (13.4%)	4,514 (13.3%)	0.005
Antiplatelet medications	14,864 (10.1%)	7,633 (11.7%)	0.048	3,786 (11.1%)	3,770 (11.1%)	0.001
Oral anticoagulants	15,122 (10.3%)	7,403 (11.3%)	0.032	3,780 (11.1%)	3,834 (11.3%)	0.005
COPD / Asthma medications	53,080 (36.2%)	22,260 (34.0%)	0.047	12,093 (35.6%)	11,961 (35.2%)	0.008
NSAIDS	49,724 (33.9%)	20,582 (31.4%)	0.054	11,367 (33.5%)	11,262 (33.1%)	0.007
Oral corticosteroids	37,969 (25.9%)	13,316 (20.3%)	0.133	7,426 (21.9%)	7,437 (21.9%)	0.001
Osteoporosis agents (incl. bisphosphonates)	3,717 (2.5%)	2,736 (4.2%)	0.091	1,197 (3.5%)	1,209 (3.6%)	0.002
Opioids	47,067 (32.1%)	21,041 (32.1%)	0.000	10,896 (32.1%)	10,780 (31.7%)	0.007
Anti-depressants	59,566 (40.6%)	21,685 (33.1%)	0.157	12,425 (36.6%)	12,325 (36.3%)	0.006
Antipsychotics	7,152 (4.9%)	3,011 (4.6%)	0.013	1,683 (5.0%)	1,618 (4.8%)	0.009
Anxiolytics / hypnotics, benzos	32,904 (22.5%)	12,831 (19.6%)	0.070	6,945 (20.4%)	6,947 (20.4%)	0.000
Dementia medications	1,819 (1.2%)	2,314 (3.5%)	0.151	854 (2.5%)	828 (2.4%)	0.005
Urinary tract infections antibiotics	68,561 (46.8%)	30,037 (45.9%)	0.019	15,597 (45.9%)	15,553 (45.8%)	0.003
Laxatives	5,244 (3.6%)	2,582 (3.9%)	0.019	1,203 (3.5%)	1,224 (3.6%)	0.003
Healthcare utilization marker						
Number of distinct medications; mean (SD) median [IQR]	14.67 ±7.03 14.00 [10.00, 19.00]	14.62 ±6.77 14.00 [10.00, 18.00]	0.008	14.93 ±6.83 14.00 [10.00, 19.00]	14.81 ±6.91 14.00 [10.00, 19.00]	0.018
Number of office visits; mean (SD) median [IQR]	9.79 ±7.09 8.00 [5.00, 13.00]	9.46 ±6.88 8.00 [5.00, 13.00]	0.047	9.70 ±6.82 8.00 [5.00, 13.00]	9.61 ±7.04 8.00 [5.00, 13.00]	0.014
Number of endocrinologist visits; mean (SD) median [IQR]	0.48 ±1.44 0.00 [0.00, 0.00]	0.32 ±1.31 0.00 [0.00, 0.00]	0.117	0.39 ±1.15 0.00 [0.00, 0.00]	0.37 ±1.50 0.00 [0.00, 0.00]	0.016
Number of cardiologist visits; mean (SD) median [IQR]	1.59 ±3.58 0.00 [0.00, 2.00]	1.89 ±3.93 0.00 [0.00, 2.00]	0.078	1.76 ±3.84 0.00 [0.00, 2.00]	1.75 ±3.82 0.00 [0.00, 2.00]	0.001
Number of internal/family medicine visits; mean (SD) median [IQR]	7.31 ±8.36 5.00 [3.00, 9.00]	8.86 ±9.69 6.00 [3.00, 11.00]	0.172	8.36 ±9.68 6.00 [3.00, 10.00]	8.31 ±9.39 6.00 [3.00, 10.00]	0.006
Number of electrocardiograms (ECG/EKG) ; mean (SD) median [IQR]	0.95 ±1.66 0.00 [0.00, 1.00]	1.12 ±1.88 1.00 [0.00, 1.00]	0.098	1.05 ±1.79 0.00 [0.00, 1.00]	1.05 ±1.83 0.00 [0.00, 1.00]	0.002
Number of echocardiograms; mean (SD) median [IQR]	0.28 ±0.64 0.00 [0.00, 0.00]	0.32 ±0.67 0.00 [0.00, 0.00]	0.061	0.30 ±0.65 0.00 [0.00, 0.00]	0.30 ±0.66 0.00 [0.00, 0.00]	0.004

Out-of-pocket medication cost; mean (SD) median [IQR]	589.31 ±766.63 349.61 [140.87, 783.39]	528.48 ±675.73 300.20 [118.88, 700.12]	0.084	556.67 ±619.95 346.20 [143.12, 756.61]	549.80 ±736.21 295.08 [116.23, 730.45]	0.010
Unique brand medicines; mean (SD) median [IQR]	14.97 ±7.27 14.00 [10.00, 19.00]	14.88 ±7.01 14.00 [10.00, 19.00]	0.012	15.22 ±7.07 14.00 [10.00, 19.00]	15.09 ±7.16 14.00 [10.00, 19.00]	0.018
Unique generic medicines; mean (SD) median [IQR]	14.67 ±7.03 14.00 [10.00, 19.00]	14.62 ±6.77 14.00 [10.00, 18.00]	0.008	14.93 ±6.83 14.00 [10.00, 19.00]	14.81 ±6.91 14.00 [10.00, 19.00]	0.018
Ratio of brand to generic medications; mean (SD) median [IQR]	1.02 ±0.04 1.00 [1.00, 1.00]	1.02 ±0.04 1.00 [1.00, 1.00]	0.053	1.02 ±0.04 1.00 [1.00, 1.00]	1.02 ±0.04 1.00 [1.00, 1.00]	0.003
Healthy behavior markers						
Colonoscopy / Sigmoidoscopy	15,505 (10.6%)	6,142 (9.4%)	0.040	3,330 (9.8%)	3,348 (9.9%)	0.002
Flu Pneumococcal vaccine	49,218 (33.6%)	25,667 (39.2%)	0.116	12,513 (36.8%)	12,484 (36.7%)	0.002
Pap smear	11,052 (7.5%)	2,812 (4.3%)	0.138	1,643 (4.8%)	1,649 (4.9%)	0.001
PSA test	31,754 (21.7%)	14,180 (21.6%)	0.001	7,375 (21.7%)	7,400 (21.8%)	0.002
Fecal occult blood test	6,401 (4.4%)	5,592 (8.5%)	0.170	2,191 (6.4%)	2,186 (6.4%)	0.001
Bone mineral density tests	10,824 (7.4%)	5,684 (8.7%)	0.047	2,864 (8.4%)	2,898 (8.5%)	0.004
Mammograms	39,540 (27.0%)	15,397 (23.5%)	0.080	8,558 (25.2%)	8,582 (25.3%)	0.002
Telemedicine	37,986 (25.9%)	10,567 (16.1%)	0.242	7,599 (22.4%)	7,496 (22.1%)	0.007
Laboratory and diagnostic tests						
HbA1c tests; mean (SD) median [IQR]	2.34 ±1.33 2.00 [1.00, 3.00]	2.42 ±1.34 2.00 [2.00, 3.00]	0.058	2.43 ±1.34 2.00 [2.00, 3.00]	2.41 ±1.34 2.00 [2.00, 3.00]	0.015
Lipid panels; mean (SD) median [IQR]	1.64 ±1.18 1.00 [1.00, 2.00]	1.70 ±1.20 2.00 [1.00, 2.00]	0.051	1.67 ±1.16 1.00 [1.00, 2.00]	1.68 ±1.18 1.00 [1.00, 2.00]	0.007
Creatinine tests; mean (SD) median [IQR]	2.87 ±2.90 2.00 [1.00, 4.00]	3.21 ±3.19 3.00 [2.00, 4.00]	0.113	3.15 ±3.27 3.00 [1.00, 4.00]	3.14 ±3.33 3.00 [1.00, 4.00]	0.004
Natriuretic peptide tests; mean (SD) median [IQR]	0.18 ±0.79 0.00 [0.00, 0.00]	0.19 ±1.59 0.00 [0.00, 0.00]	0.005	0.19 ±0.67 0.00 [0.00, 0.00]	0.19 ±2.10 0.00 [0.00, 0.00]	0.003
Urine tests; mean (SD) median [IQR]	1.04 ±1.64 0.00 [0.00, 1.00]	1.25 ±2.28 1.00 [0.00, 2.00]	0.104	1.17 ±1.80 1.00 [0.00, 2.00]	1.17 ±2.62 1.00 [0.00, 2.00]	0.001
Lab values						
HbA1c (%)*; mean (SD) median [IQR]	7.69 ±1.77 7.20 [6.40, 8.50]	8.01 ±1.66 7.70 [6.90, 8.80]	0.184	7.88 ±1.78 7.50 [6.60, 8.80]	8.04 ±1.66 7.70 [6.90, 8.80]	0.094
Glucose (mg/dl)*; mean (SD) median [IQR]	159.09 ±70.38 140.00 [112.00, 185.00]	171.69 ±71.28 156.00 [125.00, 199.00]	0.178	165.78 ±72.80 148.00 [117.00, 195.00]	171.64 ±71.59 156.00 [125.00, 199.00]	0.081
Creatinine (mg/dl)*; mean (SD) median [IQR]	1.01 ±0.93 0.90 [0.80, 1.09]	1.09 ±1.60 0.95 [0.80, 1.19]	0.054	1.06 ±1.37 0.93 [0.80, 1.15]	1.07 ±1.64 0.93 [0.80, 1.16]	0.008
Systolic blood pressure (mmHg)*; mean (SD) median [IQR]	129.84 ±19.63 130.00 [120.00, 140.00]	131.45 ±18.92 130.00 [121.00, 140.00]	0.083	130.99 ±18.36 130.00 [121.00, 140.00]	130.94 ±19.11 130.00 [121.00, 140.00]	0.003
Heart rate (1/min)*; mean (SD) median [IQR]	78.56 ±49.82 77.00 [69.00, 87.00]	77.87 ±16.26 77.00 [68.00, 86.00]	0.018	77.62 ±17.24 76.00 [68.00, 86.00]	78.02 ±16.84 77.00 [68.00, 86.00]	0.023
BMI (kg/m2)*; mean (SD) median [IQR]	37.93 ±7.77 36.61 [32.50, 41.83]	33.85 ±6.56 32.50 [29.12, 37.16]	0.566	35.65 ±7.22 34.30 [30.45, 39.27]	34.45 ±6.72 33.19 [29.64, 37.87]	0.172
eGFR (ml/min/1.73m2)*; mean (SD) median [IQR]	1,591.66 ±119,111.34 80.00 [62.00, 96.00]	629.87 ±52,913.02 71.00 [54.00, 89.00]	0.010	1,293.41 ±110,565.65 72.00 [55.00, 89.00]	688.51 ±55,585.66 72.00 [54.00, 89.00]	0.007
LDL (mg/dl)*; mean (SD) median [IQR]	86.48 ±39.37 82.80 [61.00, 109.00]	83.58 ±37.58 79.00 [60.00, 104.00]	0.075	82.82 ±38.25 78.00 [59.00, 103.00]	83.91 ±37.73 79.00 [60.00, 104.00]	0.029
HDL (mg/dl)*; mean (SD) median [IQR]	46.08 ±13.99 45.00 [37.00, 53.00]	46.35 ±13.64 45.00 [37.00, 54.00]	0.019	46.44 ±14.08 45.00 [37.00, 54.00]	45.78 ±13.48 44.00 [37.00, 53.00]	0.048
Total cholesterol (mg/dl)*; mean (SD) median [IQR]	165.41 ±48.78 161.00 [135.00, 192.00]	163.03 ±46.09 158.00 [133.00, 187.00]	0.050	161.73 ±46.70 156.00 [132.00, 187.00]	162.19 ±47.32 157.00 [132.00, 187.00]	0.010
Triglyceride (mg/dl)*; mean (SD) median [IQR]	177.55 ±160.58 145.00 [104.00, 207.00]	170.21 ±128.81 143.00 [103.00, 202.00]	0.050	172.44 ±130.39 144.00 [103.00, 204.00]	172.59 ±139.71 144.00 [104.00, 204.00]	0.001

**Burden of comorbidities**						
Combined comorbidity score; mean (SD) median [IQR]	1.96 ±2.38 1.00 [0.00, 3.00]	2.62 ±2.77 2.00 [1.00, 4.00]	0.255	2.37 ±2.62 2.00 [0.00, 4.00]	2.35 ±2.58 2.00 [0.00, 4.00]	0.009
Frailty Score; mean (SD) median [IQR]	0.17 ±0.06 0.16 [0.13, 0.19]	0.18 ±0.06 0.17 [0.13, 0.21]	0.187	0.17 ±0.06 0.16 [0.13, 0.20]	0.17 ±0.06 0.16 [0.13, 0.20]	0.009
**Baseline hospitalizations and hospital metrics**						
Number of Hospitalizations; mean (SD) median [IQR]	0.20 ±0.65 0.00 [0.00, 0.00]	0.33 ±0.96 0.00 [0.00, 0.00]	0.168	0.27 ±0.85 0.00 [0.00, 0.00]	0.27 ±0.84 0.00 [0.00, 0.00]	0.001
Any hospitalization within prior 91 days	6,298 (4.3%)	5,714 (8.7%)	0.180	2,238 (6.6%)	2,192 (6.5%)	0.005
Any hospitalization within prior 92-365 days	14,569 (9.9%)	8,358 (12.8%)	0.089	3,899 (11.5%)	3,889 (11.4%)	0.001
Number of hospitalizations (0, 1, 2 or more)						
< 1	127,957 (87.3%)	53,635 (81.9%)	0.151	28,746 (84.6%)	28,752 (84.6%)	0.0
1 - <2	12,994 (8.9%)	7,022 (10.7%)	0.062	3,279 (9.7%)	3,294 (9.7%)	0.001
>= 2	5,589 (3.8%)	4,852 (7.4%)	0.157	1,948 (5.7%)	1,927 (5.7%)	0.003
Heart failure hospitalization	5,056 (3.5%)	3,416 (5.2%)	0.087	1,448 (4.3%)	1,491 (4.4%)	0.006
ED visit	41,255 (28.2%)	24,198 (36.9%)	0.188	11,610 (34.2%)	11,600 (34.1%)	0.001
**Calendar year of cohort entry**						
2018	193 (0.1%)	12,168 (18.6%)	0.668	193 (0.6%)	217 (0.6%)	0.009
2019	5,785 (3.9%)	11,876 (18.1%)	0.465	3,962 (11.7%)	3,836 (11.3%)	0.012
2020	8,825 (6.0%)	10,642 (16.2%)	0.329	5,033 (14.8%)	4,986 (14.7%)	0.004
2021	14,639 (10.0%)	9,335 (14.2%)	0.131	5,951 (17.5%)	5,898 (17.4%)	0.004
2022	24,189 (16.5%)	7,776 (11.9%)	0.133	6,202 (18.3%)	6,275 (18.5%)	0.006
2023	44,241 (30.2%)	6,935 (10.6%)	0.502	6,366 (18.7%)	6,405 (18.9%)	0.003
2024	47,352 (32.3%)	6,742 (10.3%)	0.558	6,225 (18.3%)	6,323 (18.6%)	0.007
2025	1,316 (0.9%)	35 (0.1%)	0.123	41 (0.1%)	33 (0.1%)	0.007

Abbreviations: *not used in the propensity score; ACE, angiotensin-converting enzyme inhibitors; ARB, angiotensin receptor blocker; ARNI, angiotensin receptor/neprilysin inhibitor; BMI, body mass index; CABG, coronary artery bypass graft surgery; CED, cohort entry date; CKD, chronic kidney disease; COPD, chronic obstructive pulmonary disease; DKA, diabetic ketoacidosis; DPP4i, dipeptidyl peptidase-4 inhibitors; ED, emergency department; eGFR, estimated glomerular filtration rate (estimated using the quadratic GFR equation: GFR = EXP(1.911 + (5.249 / Serum creatinine) - (2.114 / (Serum creatinine^2)) - (0.00686 * Age) - 0.205 (if female))); HbA1c, hemoglobin A1c; HDL, high-density lipoprotein cholesterol; HONK, hyperglycemic hyperosmolar nonketotic state; IQR, inter-quartile-range; MASH, metabolic dysfunction associated steatohepatitis; MASLD, metabolic dysfunction associated steatotic liver disease; MI, myocardial infarction; N, number of participants; NSAIDs, non-steroidal anti-inflammatory drugs; PCSK9, proprotein convertase subtilisin/kexin type 9; PSA, prostate-specific antigen; PTCA, percutaneous transluminal coronary angioplasty; SGLT2i, sodium-glucose transport protein 2 inhibitors; SD, standard deviation; SMD, standardized mean difference; TIA, transient ischemic attack.

Missing data were handled by assuming absence of a code indicated absence of the condition for most binary covariates. Missing indicators were included for race and region in the propensity score model.

Laboratory values were only available in a subset of the Optum database and were truncated using clinically plausible cut-off values (BMI values >100 or <10 were set to missing; missingness before matching ~63%. Creatinine values >30 were set to missing, and values <0.8 were set to 0.8; missingness before matching ~41%. eGFR values >150 were set to 150, and values =0 were set to missing; missingness before matching ~71%. Glucose values <30 were set to missing; missingness before matching ~41%. HbA1c values ≥20 or ≤2 were set to missing; missingness before matching ~37%. HDL value missingness before matching was ~48%. Heart rate values <30 were set to missing; missingness before matching ~75%. LDL value missingness before matching was~46%. Systolic blood pressure values <30 were set to missing; missingness before matching

~61%. Total cholesterol value missingness before matching was ~48%. Triglyceride value missingness before matching was ~49%).
The Race-Others category includes Asian and Hispanic individuals.

Supplementary Table 8. Baseline characteristics of initiators of semaglutide vs sitagliptin when applying trial eligibility criteria before and after propensity score matching, Marketscan database. Values are number (percentage) unless otherwise specified.

	Before propensity score matching			After propensity score matching		
Variable	Semaglutide (n = 100,869)	Sitagliptin (n = 62,457)	SMD	Semaglutide (n = 33,189)	Sitagliptin (n = 33,189)	SMD
Demographics						
Age; mean (SD) median [IQR]	53.66 ±9.98 54.00 [47.00, 60.00]	57.85 ±11.72 58.00 [50.00, 64.00]	0.385	55.51 ±9.68 56.00 [49.00, 61.00]	55.50 ±11.21 56.00 [48.00, 62.00]	0.000
Gender						
Male	45,429 (45.0%)	32,670 (52.3%)	0.146	16,431 (49.5%)	16,431 (49.5%)	0.000
Female	55,440 (55,0%)	29,787 (47,7%)	0.146	16,758 (50,5%)	16,758 (50,5%)	0.000
Region / State						
Northeast	11,928 (11.8%)	10,524 (16.8%)	0.144	4,827 (14.5%)	4,897 (14.8%)	0.006
Midwest / North central	20,650 (20.5%)	16,026 (25.7%)	0.123	7,344 (22.1%)	7,415 (22.3%)	0.005
South	59,129 (58.6%)	31,366 (50.2%)	0.169	18,418 (55.5%)	18,355 (55.3%)	0.004
West	9,066 (9.0%)	4,434 (7.1%)	0.069	2,559 (7.7%)	2,479 (7.5%)	0.009
Missing	96 (0.1%)	107 (0.2%)	0.021	41 (0.1%)	43 (0.1%)	0.002
Lifestyle risk factors						
Smoking/Tobacco use	9,881 (9.8%)	6,486 (10.4%)	0.020	3,338 (10.1%)	3,336 (10.1%)	0.000
Weight						
Underweight	37 (0.0%)	37 (0.1%)	0.010	20 (0.1%)	14 (0.0%)	0.008
Normal weight	199 (0.2%)	516 (0.8%)	0.088	148 (0.4%)	143 (0.4%)	0.002
Overweight (BMI 25.0-29.9)	8,454 (8.4%)	11,877 (19.0%)	0.313	4,509 (13.6%)	4,432 (13.4%)	0.007
Class 1 Obesity (BMI 30.0-34.9)	8,446 (8.4%)	8,213 (13.1%)	0.155	3,747 (11.3%)	3,714 (11.2%)	0.003
Class 2 Obesity (BMI 35.0-39.9)	7,022 (7.0%)	4,739 (7.6%)	0.024	2,577 (7.8%)	2,577 (7.8%)	0.000
Class 3 Obesity; (BMI 40.0 and above)	30,309 (30.0%)	15,207 (24.3%)	0.128	9,875 (29.8%)	9,802 (29.5%)	0.005
Unspecified Obesity	46,402 (46.0%)	21,868 (35.0%)	0.225	12,313 (37.1%)	12,507 (37.7%)	0.012
Diabetes complications						
Diabetic retinopathy	5,532 (5.5%)	3,779 (6.1%)	0.024	1,970 (5.9%)	1,906 (5.7%)	0.008
Diabetic neuropathy	14,342 (14.2%)	10,029 (16.1%)	0.051	5,082 (15.3%)	5,062 (15.3%)	0.002
Diabetic nephropathy	10,355 (10.3%)	7,446 (11.9%)	0.053	3,791 (11.4%)	3,623 (10.9%)	0.016
Diabetes with other ophthalmic complications	1,336 (1.3%)	929 (1.5%)	0.014	488 (1.5%)	484 (1.5%)	0.001
Diabetes with peripheral circulatory disorders	6,575 (6.5%)	3,938 (6.3%)	0.009	2,062 (6.2%)	2,023 (6.1%)	0.005
Diabetic foot	1,519 (1.5%)	1,033 (1.7%)	0.012	497 (1.5%)	511 (1.5%)	0.003
Erectile dysfunction	3,749 (3.7%)	2,436 (3.9%)	0.010	1,337 (4.0%)	1,300 (3.9%)	0.006
Hypoglycemia	16,135 (16.0%)	7,914 (12.7%)	0.095	4,648 (14.0%)	4,631 (14.0%)	0.001
Hyperglycemia/DKA/HONK	50,576 (50.1%)	30,989 (49.6%)	0.010	17,227 (51.9%)	17,026 (51.3%)	0.012
Skin infections	8,922 (8.8%)	5,552 (8.9%)	0.002	2,990 (9.0%)	2,954 (8.9%)	0.004
Cardiovascular-related conditions						

Coronary atherosclerosis	10,879 (10.8%)	8,059 (12.9%)	0.066	3,803 (11.5%)	3,692 (11.1%)	0.011
Stable angina	2,179 (2.2%)	1,440 (2.3%)	0.010	757 (2.3%)	737 (2.2%)	0.004
Unstable angina	1,554 (1.5%)	1,143 (1.8%)	0.022	545 (1.6%)	538 (1.6%)	0.002
Hypertension	80,690 (80.0%)	51,108 (81.8%)	0.047	26,784 (80.7%)	26,797 (80.7%)	0.001
Hypotension	1,289 (1.3%)	1,071 (1.7%)	0.036	472 (1.4%)	482 (1.5%)	0.003
Hyperlipidemia	78,385 (77.7%)	49,887 (79.9%)	0.053	26,364 (79.4%)	26,306 (79.3%)	0.004
Acute MI	892 (0.9%)	647 (1.0%)	0.016	310 (0.9%)	287 (0.9%)	0.007
Old MI	1,506 (1.5%)	1,019 (1.6%)	0.011	512 (1.5%)	479 (1.4%)	0.008
TIA	1,023 (1.0%)	858 (1.4%)	0.033	364 (1.1%)	372 (1.1%)	0.002
Ischemic stroke	709 (0.7%)	716 (1.1%)	0.046	276 (0.8%)	268 (0.8%)	0.003
Cardiac conduction disorder	2,541 (2.5%)	1,831 (2.9%)	0.025	846 (2.5%)	830 (2.5%)	0.003
Previous cardiac procedure (CABG, PTCA, Stent)	1,238 (1.2%)	821 (1.3%)	0.008	446 (1.3%)	421 (1.3%)	0.007
PVD diagnosis or surgery	3,300 (3.3%)	3,073 (4.9%)	0.083	1,299 (3.9%)	1,268 (3.8%)	0.005
Atrial fibrillation	4,460 (4.4%)	3,601 (5.8%)	0.061	1,558 (4.7%)	1,568 (4.7%)	0.001
Other cardiac dysrhythmia	13,698 (13.6%)	8,337 (13.3%)	0.007	4,214 (12.7%)	4,225 (12.7%)	0.001
Heart failure	5,305 (5.3%)	3,892 (6.2%)	0.042	1,789 (5.4%)	1,724 (5.2%)	0.009
Acute heart failure	1,321 (1.3%)	1,167 (1.9%)	0.045	480 (1.4%)	433 (1.3%)	0.012
Cardiomyopathy	2,617 (2.6%)	1,851 (3.0%)	0.022	874 (2.6%)	841 (2.5%)	0.006
Valve disorders	5,470 (5.4%)	3,978 (6.4%)	0.040	1,843 (5.6%)	1,840 (5.5%)	0.000
Valve replacement	433 (0.4%)	377 (0.6%)	0.024	150 (0.5%)	142 (0.4%)	0.004
Edema	7,624 (7.6%)	4,489 (7.2%)	0.014	2,314 (7.0%)	2,310 (7.0%)	0.000
Venous thromboembolism / Pulmonary embolism	2,202 (2.2%)	1,419 (2.3%)	0.006	685 (2.1%)	709 (2.1%)	0.005
Pulmonary hypertension	1,064 (1.1%)	784 (1.3%)	0.019	344 (1.0%)	357 (1.1%)	0.004
Implantable cardioverter defibrillator	164 (0.2%)	111 (0.2%)	0.004	56 (0.2%)	52 (0.2%)	0.003
Hyperkalemia	1,221 (1.2%)	1,020 (1.6%)	0.036	486 (1.5%)	455 (1.4%)	0.008
Cerebrovascular procedure	54 (0.1%)	52 (0.1%)	0.011	23 (0.1%)	15 (0.0%)	0.010
Insertion of pacemakers / removal of cardiac lead	43 (0.0%)	59 (0.1%)	0.020	19 (0.1%)	16 (0.0%)	0.004
Renal-related conditions						
Hypertensive nephropathy	4,035 (4.0%)	3,300 (5.3%)	0.061	1,473 (4.4%)	1,428 (4.3%)	0.007
CKD stage 1-2	1,896 (1.9%)	1,356 (2.2%)	0.021	660 (2.0%)	648 (2.0%)	0.003
CKD stage 3-4	4,484 (4.4%)	3,936 (6.3%)	0.082	1,721 (5.2%)	1,697 (5.1%)	0.003
Unspecified CKD	1,661 (1.6%)	1,464 (2.3%)	0.050	612 (1.8%)	603 (1.8%)	0.002
Microalbuminuria or proteinuria	3,523 (3.5%)	2,086 (3.3%)	0.008	1,170 (3.5%)	1,125 (3.4%)	0.007
Acute kidney injury	2,729 (2.7%)	2,551 (4.1%)	0.076	1,044 (3.1%)	1,044 (3.1%)	0.000
Urinary tract infections	9,045 (9.0%)	6,110 (9.8%)	0.028	3,017 (9.1%)	3,028 (9.1%)	0.001
Genital infections	4,554 (4.5%)	3,002 (4.8%)	0.014	1,701 (5.1%)	1,669 (5.0%)	0.004
Urolithiasis (Kidney and urinary stone)	3,895 (3.9%)	2,546 (4.1%)	0.011	1,292 (3.9%)	1,306 (3.9%)	0.002
Other comorbidities						
COPD	4,152 (4.1%)	3,481 (5.6%)	0.068	1,461 (4.4%)	1,543 (4.6%)	0.012

Asthma	9,455 (9.4%)	4,762 (7.6%)	0.063	2,696 (8.1%)	2,716 (8.2%)	0.002
Obstructive sleep apnea	29,550 (29.3%)	13,054 (20.9%)	0.195	8,000 (24.1%)	8,034 (24.2%)	0.002
Serious bacterial infections	2,548 (2.5%)	2,094 (3.4%)	0.049	951 (2.9%)	923 (2.8%)	0.005
Pneumonia	3,407 (3.4%)	2,437 (3.9%)	0.028	1,173 (3.5%)	1,202 (3.6%)	0.005
Liver disease	13,750 (13.6%)	7,433 (11.9%)	0.052	4,195 (12.6%)	4,129 (12.4%)	0.006
MASH / MASLD	9,357 (9.3%)	4,311 (6.9%)	0.087	2,659 (8.0%)	2,632 (7.9%)	0.003
Fractures / Falls	2,843 (2.8%)	2,244 (3.6%)	0.044	994 (3.0%)	1,011 (3.0%)	0.003
Osteoporosis	1,165 (1.2%)	1,016 (1.6%)	0.040	423 (1.3%)	444 (1.3%)	0.006
Osteoarthritis	18,776 (18.6%)	11,268 (18.0%)	0.015	5,829 (17.6%)	5,868 (17.7%)	0.003
Depression	17,368 (17.2%)	8,553 (13.7%)	0.098	5,038 (15.2%)	4,995 (15.1%)	0.004
Dementia	857 (0.8%)	1,357 (2.2%)	0.109	390 (1.2%)	392 (1.2%)	0.001
Delirium or psychosis	548 (0.5%)	503 (0.8%)	0.032	192 (0.6%)	203 (0.6%)	0.004
Anxiety	20,396 (20.2%)	8,628 (13.8%)	0.171	5,250 (15.8%)	5,341 (16.1%)	0.007
Sleep disorders	25,829 (25.6%)	14,645 (23.4%)	0.05	8,122 (24.5%)	8,096 (24.4%)	0.002
Anemia	13,368 (13.3%)	8,745 (14.0%)	0.022	4,333 (13.1%)	4,319 (13.0%)	0.001
Influenza	1,921 (1.9%)	1,415 (2.3%)	0.025	637 (1.9%)	632 (1.9%)	0.001
COVID	11,540 (11.4%)	2,765 (4.4%)	0.262	2,277 (6.9%)	2,353 (7.1%)	0.009
Hyperthyroidism and other thyroid gland disorders	21,543 (21.4%)	11,945 (19.1%)	0.056	6,564 (19.8%)	6,590 (19.9%)	0.002
Hypothyroidism	16,643 (16.5%)	9,343 (15.0%)	0.042	5,129 (15.5%)	5,089 (15.3%)	0.003
Nephrotic syndrome	66 (0.1%)	40 (0.1%)	0.001	22 (0.1%)	20 (0.1%)	0.002
Urinary incontinence	2,599 (2.6%)	1,557 (2.5%)	0.005	789 (2.4%)	791 (2.4%)	0.000
Biliary disease	248 (0.2%)	235 (0.4%)	0.023	101 (0.3%)	103 (0.3%)	0.001
Pancreatitis	124 (0.1%)	132 (0.2%)	0.022	62 (0.2%)	50 (0.2%)	0.009
Bowel obstruction	114 (0.1%)	99 (0.2%)	0.012	38 (0.1%)	35 (0.1%)	0.003
Gastroparesis	497 (0.5%)	330 (0.5%)	0.005	200 (0.6%)	184 (0.6%)	0.006
Diabetes medications						
Number of antidiabetic drugs on CED; mean (SD) median [IQR]	2.06 ±0.91 2.00 [1.00, 3.00]	2.21 ±0.82 2.00 [2.00, 3.00]	0.165	2.20 ±0.90 2.00 [2.00, 3.00]	2.19 ±0.85 2.00 [2.00, 3.00]	0.019
Concomitant use or initiation of Metformin	55,123 (54.6%)	42,974 (68.8%)	0.294	21,440 (64.6%)	21,444 (64.6%)	0.000
Concomitant use or initiation of Insulins	16,996 (16.8%)	6,002 (9.6%)	0.215	4,572 (13.8%)	4,330 (13.0%)	0.021
Concomitant use or initiation of Sulfonylureas	13,292 (13.2%)	15,070 (24.1%)	0.284	6,481 (19.5%)	6,445 (19.4%)	0.003
Concomitant use or initiation of SGLT-2i	18,057 (17.9%)	8,056 (12.9%)	0.139	5,810 (17.5%)	5,572 (16.8%)	0.019
Concomitant use or initiation of Any other glucose-lowering drugs	3,806 (3.8%)	3,023 (4.8%)	0.053	1,582 (4.8%)	1,549 (4.7%)	0.005
Past use of Metformin	72,578 (72.0%)	47,787 (76.5%)	0.104	25,416 (76.6%)	25,388 (76.5%)	0.002
Past use of Insulins	22,691 (22.5%)	8,397 (13.4%)	0.237	6,184 (18.6%)	5,913 (17.8%)	0.021
Past use of Sulfonylureas	19,234 (19.1%)	19,775 (31.7%)	0.293	8,834 (26.6%)	8,806 (26.5%)	0.002
Past use of SGLT-2i	23,796 (23.6%)	11,402 (18.3%)	0.131	7,761 (23.4%)	7,508 (22.6%)	0.018
Past use of Any other glucose-lowering drugs	5,580 (5.5%)	4,351 (7.0%)	0.059	2,280 (6.9%)	2,242 (6.8%)	0.005
Other medications						
ACE / ARB	70,619 (70.0%)	46,356 (74.2%)	0.094	24,155 (72.8%)	24,113 (72.7%)	0.003

ARNI	1,029 (1.0%)	352 (0.6%)	0.052	225 (0.7%)	221 (0.7%)	0.001
Thiazides	35,859 (35.6%)	20,945 (33.5%)	0.042	11,415 (34.4%)	11,443 (34.5%)	0.002
Beta-blockers	31,879 (31.6%)	20,935 (33.5%)	0.041	10,528 (31.7%)	10,504 (31.6%)	0.002
Calcium channel blockers	27,539 (27.3%)	18,193 (29.1%)	0.041	9,183 (27.7%)	9,267 (27.9%)	0.006
Digoxin / Digitoxin	309 (0.3%)	366 (0.6%)	0.042	136 (0.4%)	137 (0.4%)	0.000
Loop diuretics	9,811 (9.7%)	6,024 (9.6%)	0.003	3,016 (9.1%)	3,005 (9.1%)	0.001
Other diuretics	7,652 (7.6%)	3,731 (6.0%)	0.064	2,148 (6.5%)	2,092 (6.3%)	0.007
Intravenous diuretics	565 (0.6%)	331 (0.5%)	0.004	154 (0.5%)	153 (0.5%)	0.000
Nitrates	3,482 (3.5%)	2,636 (4.2%)	0.040	1,216 (3.7%)	1,196 (3.6%)	0.003
Anti-arrhythmics	1,313 (1.3%)	881 (1.4%)	0.009	436 (1.3%)	432 (1.3%)	0.001
Statins	72,447 (71.8%)	46,719 (74.8%)	0.067	24,647 (74.3%)	24,612 (74.2%)	0.002
PCSK9 inhibitors and other lipid-lowering drugs	11,487 (11.4%)	7,580 (12.1%)	0.023	4,013 (12.1%)	3,925 (11.8%)	0.008
Antiplatelet medications	8,005 (7.9%)	6,224 (10.0%)	0.071	2,967 (8.9%)	2,902 (8.7%)	0.007
Oral anticoagulants	5,754 (5.7%)	4,068 (6.5%)	0.034	1,957 (5.9%)	1,940 (5.8%)	0.002
COPD / Asthma medications	32,810 (32.5%)	17,438 (27.9%)	0.100	9,758 (29.4%)	9,796 (29.5%)	0.003
NSAIDS	34,820 (34.5%)	19,570 (31.3%)	0.068	10,910 (32.9%)	10,961 (33.0%)	0.003
Oral corticosteroids	26,471 (26.2%)	12,920 (20.7%)	0.131	7,345 (22.1%)	7,444 (22.4%)	0.007
Osteoporosis agents (incl. bisphosphonates)	1,196 (1.2%)	955 (1.5%)	0.030	442 (1.3%)	431 (1.3%)	0.003
Opioids	26,257 (26.0%)	16,005 (25.6%)	0.009	8,468 (25.5%)	8,340 (25.1%)	0.009
Anti-depressants	36,709 (36.4%)	16,775 (26.9%)	0.206	10,178 (30.7%)	10,111 (30.5%)	0.004
Antipsychotics	2,981 (3.0%)	1,492 (2.4%)	0.035	860 (2.6%)	838 (2.5%)	0.004
Anxiolytics / hypnotics, benzos	20,355 (20.2%)	9,813 (15.7%)	0.117	5,682 (17.1%)	5,743 (17.3%)	0.005
Dementia medications	320 (0.3%)	673 (1.1%)	0.091	158 (0.5%)	158 (0.5%)	0.000
Urinary tract infections antibiotics	44,562 (44.2%)	26,465 (42.4%)	0.036	14,162 (42.7%)	14,049 (42.3%)	0.007
Laxatives	2,946 (2.9%)	1,550 (2.5%)	0.027	850 (2.6%)	832 (2.5%)	0.003
Healthcare utilization marker						
Number of distinct medications; mean (SD) median {IQR}	13.17 ±6.44 12.00 [9.00, 17.00]	11.96 ±6.02 11.00 [8.00, 15.00]	0.194	12.46 ±5.91 11.00 [8.00, 16.00]	12.39 ±6.31 11.00 [8.00, 16.00]	0.011
Number of office visits; mean (SD) median {IQR}	8.40 ±6.30 7.00 [4.00, 11.00]	7.61 ±5.98 6.00 [4.00, 10.00]	0.128	7.87 ±5.89 6.00 [4.00, 10.00]	7.81 ±6.10 6.00 [4.00, 10.00]	0.010
Number of endocrinologist visits; mean (SD) median {IQR}	0.43 ±1.26 0.00 [0.00, 0.00]	0.26 ±1.08 0.00 [0.00, 0.00]	0.149	0.36 ±1.02 0.00 [0.00, 0.00]	0.34 ±1.31 0.00 [0.00, 0.00]	0.021
Number of cardiologist visits; mean (SD) median {IQR}	0.90 ±2.53 0.00 [0.00, 1.00]	0.96 ±2.68 0.00 [0.00, 1.00]	0.023	0.91 ±2.52 0.00 [0.00, 1.00]	0.88 ±2.41 0.00 [0.00, 1.00]	0.015
Number of internal/family medicine visits; mean (SD) median {IQR}	4.82 ±5.29 4.00 [2.00, 6.00]	5.37 ±6.38 4.00 [2.00, 7.00]	0.095	5.08 ±5.60 4.00 [2.00, 7.00]	5.00 ±5.56 4.00 [2.00, 6.00]	0.014
Number of Electrocardiograms (ECG/EKG) ; mean (SD) median {IQR}	0.72 ±1.35 0.00 [0.00, 1.00]	0.81 ±1.65 0.00 [0.00, 1.00]	0.057	0.75 ±1.40 0.00 [0.00, 1.00]	0.74 ±1.37 0.00 [0.00, 1.00]	0.009
Number of Echocardiograms; mean (SD) median {IQR}	0.21 ±0.62 0.00 [0.00, 0.00]	0.23 ±0.65 0.00 [0.00, 0.00]	0.029	0.21 ±0.60 0.00 [0.00, 0.00]	0.21 ±0.59 0.00 [0.00, 0.00]	0.010
Out-of-pocket medication cost; mean (SD) median {IQR}	480.98 ±813.50 285.52 [110.00, 611.65]	389.99 ±559.90 253.61 [105.46, 500.00]	0.130	434.85 ±595.56 271.43 [110.00, 573.54]	429.65 ±662.77 266.10 [106.06, 551.39]	0.008
Unique brand medicines; mean (SD) median {IQR}	13.44 ±6.62 12.00 [9.00, 17.00]	12.20 ±6.21 11.00 [8.00, 15.00]	0.194	12.70 ±6.07 12.00 [8.00, 16.00]	12.64 ±6.51 11.00 [8.00, 16.00]	0.009
Unique generic medicines; mean (SD) median {IQR}	13.17 ±6.44 12.00 [9.00, 17.00]	11.96 ±6.02 11.00 [8.00, 15.00]	0.194	12.46 ±5.91 11.00 [8.00, 16.00]	12.39 ±6.31 11.00 [8.00, 16.00]	0.011

Ratio of brand to generic medications; mean (SD) median {IQR}	1.02 ±0.05 1.00 [1.00, 1.00]	1.02 ±0.05 1.00 [1.00, 1.00]	0.035	1.02 ±0.04 1.00 [1.00, 1.00]	1.02 ±0.05 1.00 [1.00, 1.00]	0.003
**Healthy behavior markers**						
Colonoscopy / Sigmoidoscopy	10,417 (10.3%)	5,892 (9.4%)	0.030	3,207 (9.7%)	3,195 (9.6%)	0.001
Flu Pneumococcal vaccine	29,026 (28.8%)	19,898 (31.9%)	0.067	10,040 (30.3%)	10,109 (30.5%)	0.005
Pap smear	12,717 (12.6%)	5,815 (9.3%)	0.106	3,596 (10.8%)	3,589 (10.8%)	0.001
PSA test	19,787 (19.6%)	13,757 (22.0%)	0.059	7,046 (21.2%)	7,069 (21.3%)	0.002
Fecal occult blood test	3,797 (3.8%)	3,407 (5.5%)	0.081	1,518 (4.6%)	1,520 (4.6%)	0.000
Bone mineral density tests	3,229 (3.2%)	1,940 (3.1%)	0.005	1,038 (3.1%)	1,055 (3.2%)	0.003
Mammograms	26,469 (26.2%)	12,630 (20.2%)	0.143	7,624 (23.0%)	7,642 (23.0%)	0.001
Telemedicine	32,017 (31.7%)	9,901 (15.9%)	0.380	7,900 (23.8%)	7,916 (23.9%)	0.001
**Laboratory and diagnostic tests**						
HbA1c tests; mean (SD) median [IQR]	2.15 ±1.47 2.00 [1.00, 3.00]	2.08 ±1.54 2.00 [1.00, 3.00]	0.046	2.16 ±1.42 2.00 [1.00, 3.00]	2.16 ±1.42 2.00 [1.00, 3.00]	0.004
Lipid panels; mean (SD) median [IQR]	1.55 ±1.29 1.00 [1.00, 2.00]	1.50 ±1.36 1.00 [1.00, 2.00]	0.037	1.55 ±1.26 1.00 [1.00, 2.00]	1.55 ±1.23 1.00 [1.00, 2.00]	0.001
Creatinine tests; mean (SD) median [IQR]	0.04 ±0.37 0.00 [0.00, 0.00]	0.05 ±0.36 0.00 [0.00, 0.00]	0.025	0.04 ±0.38 0.00 [0.00, 0.00]	0.04 ±0.32 0.00 [0.00, 0.00]	0.001
Natriuretic peptide tests; mean (SD) median [IQR]	0.12 ±0.88 0.00 [0.00, 0.00]	0.11 ±0.95 0.00 [0.00, 0.00]	0.014	0.11 ±0.84 0.00 [0.00, 0.00]	0.11 ±1.01 0.00 [0.00, 0.00]	0.001
Urine tests; mean (SD) median [IQR]	0.90 ±1.62 0.00 [0.00, 1.00]	0.96 ±1.77 0.00 [0.00, 1.00]	0.033	0.95 ±1.79 0.00 [0.00, 1.00]	0.94 ±1.67 0.00 [0.00, 1.00]	0.010
**Burden of comorbidities**						
Combined comorbidity score; mean (SD) median {IQR}	1.00 ±1.66 1.00 [0.00, 2.00]	1.14 ±1.93 1.00 [0.00, 2.00]	0.076	1.04 ±1.71 1.00 [0.00, 2.00]	1.02 ±1.73 1.00 [0.00, 2.00]	0.011
Frailty Score; mean (SD) median {IQR}	0.14 ±0.04 0.14 [0.12, 0.16]	0.15 ±0.05 0.14 [0.12, 0.17]	0.104	0.14 ±0.04 0.14 [0.12, 0.16]	0.14 ±0.04 0.13 [0.12, 0.16]	0.002
**Baseline hospitalizations and hospital metrics**						
Number of Hospitalizations; mean (SD) median {IQR}	4.93 ±26.63 0.00 [0.00, 0.00]	6.43 ±28.23 0.00 [0.00, 0.00]	0.055	5.64 ±33.72 0.00 [0.00, 0.00]	5.36 ±26.49 0.00 [0.00, 0.00]	0.009
Any hospitalization within prior 91 days	2,965 (2.9%)	3,211 (5.1%)	0.112	1,319 (4.0%)	1,245 (3.8%)	0.012
Any hospitalization within prior 92-365 days	6,625 (6.6%)	4,658 (7.5%)	0.035	2,243 (6.8%)	2,181 (6.6%)	0.007
Number of hospitalizations (0, 1, 2 or more)						
< 1	91,922 (91.1%)	55,378 (88.7%)	0.082	29,918 (90.1%)	30,029 (90.5%)	0.011
1 - <2	735 (0.7%)	538 (0.9%)	0.015	267 (0.8%)	248 (0.7%)	0.007
>= 2	8,212 (8.1%)	6,541 (10.5%)	0.080	3,004 (9.1%)	2,912 (8.8%)	0.010
Heart failure hospitalization	1,752 (1.7%)	1,638 (2.6%)	0.061	665 (2.0%)	593 (1.8%)	0.016
ED visit	29,567 (29.3%)	18,928 (30.3%)	0.022	9,775 (29.5%)	9,700 (29.2%)	0.005
**Calendar year of cohort entry**						
2018	2,333 (2.3%)	16,154 (25.9%)	0.719	2,274 (6.9%)	2,254 (6.8%)	0.002
2019	8,407 (8.3%)	14,495 (23.2%)	0.417	6,664 (20.1%)	6,554 (19.7%)	0.008
2020	9,915 (9.8%)	11,269 (18.0%)	0.239	6,730 (20.3%)	6,697 (20.2%)	0.002
2021	15,532 (15.4%)	9,490 (15.2%)	0.006	7,371 (22.2%)	7,370 (22.2%)	0.000
2022	22,846 (22.6%)	6,420 (10.3%)	0.338	5,780 (17.4%)	5,840 (17.6%)	0.005
2023	41,836 (41.5%)	4,629 (7.4%)	0.863	4,370 (13.2%)	4,474 (13.5%)	0.009

Abbreviations: *not used in the propensity score; ACE, angiotensin-converting enzyme inhibitors; ARB, angiotensin receptor blocker; ARNI, angiotensin receptor/neprilysin inhibitor; BMI, body mass index; CABG, coronary artery bypass graft surgery; CED, cohort entry date; CKD, chronic kidney disease; COPD, chronic

obstructive pulmonary disease; DKA, diabetic ketoacidosis; DPP4i, dipeptidyl peptidase-4 inhibitors; ED, emergency department; eGFR, estimated glomerular filtration rate (estimated using the quadratic GFR equation: GFR = EXP(1.911 + (5.249 / Serum creatinine) - (2.114 / (Serum creatinine^2)) - (0.00686 * Age) - 0.205 (if female))); HbA1c, hemoglobin A1c; HDL, high-density lipoprotein cholesterol; HONK, hyperglycemic hyperosmolar nonketotic state; IQR, inter-quartile-range; MASH, metabolic dysfunction associated steatohepatitis; MASLD, metabolic dysfunction associated steatotic liver disease; MI, myocardial infarction; N, number of participants; NSAIDs, non-steroidal anti-inflammatory drugs; PCSK9, proprotein convertase subtilisin/kexin type 9; PSA, prostate-specific antigen; PTCA, percutaneous transluminal coronary angioplasty; SGLT2i, sodium-glucose transport protein 2 inhibitors; SD, standard deviation; SMD, standardized mean difference; TIA, transient ischemic attack.
Missing data were handled by assuming absence of a code indicated absence of the condition for most binary covariates. Missing indicators were included for race and region in the propensity score model.

Supplementary Table 9. Baseline characteristics of initiators of semaglutide vs sitagliptin when applying trial eligibility criteria before and after propensity score matching, Medicare database. Values are number (percentage) unless otherwise specified.

	Before propensity score matching			After propensity score matching		
Variable	Semaglutide (n = 14,635)	Sitagliptin (n = 63,191)	SMD	Semaglutide (n = 12,339)	Sitagliptin (n = 12,339)	SMD
**Demographics**						
Age; mean (SD) median [IQR]	71.51 ±4.76 71.00 [68.00, 74.00]	73.98 ±6.31 73.00 [69.00, 78.00]	0.441	71.79 ±4.88 71.00 [68.00, 75.00]	71.74 ±5.08 71.00 [68.00, 75.00]	0.010
Gender						
Male	6,772 (46.3%)	26,463 (41.9%)	0.089	5,585 (45.3%)	5,467 (44.3%)	0.019
Female	7,863 (53,7%)	36,728 (58,1%)	0.089	6,754 (54,7%)	6,872 (55,7%)	0.019
Race						
White	12,226 (83.5%)	48,071 (76.1%)	0.187	10,201 (82.7%)	10,186 (82.6%)	0.003
Other	623 (4.3%)	4,740 (7.5%)	0.138	564 (4.6%)	570 (4.6%)	0.002
Black	1,274 (8.7%)	8,181 (12.9%)	0.137	1,143 (9.3%)	1,152 (9.3%)	0.003
Unknown / Missing	512 (3.5%)	2,199 (3.5%)	0.001	431 (3.5%)	431 (3.5%)	0.000
Region / State						
Northeast	2,368 (16.2%)	12,489 (19.8%)	0.093	2,059 (16.7%)	2,018 (16.4%)	0.009
Midwest / North central	3,539 (24.2%)	13,550 (21.4%)	0.065	2,893 (23.4%)	2,901 (23.5%)	0.002
South	6,779 (46.3%)	27,343 (43.3%)	0.061	5,672 (46.0%)	5,759 (46.7%)	0.014
West	1,943 (13.3%)	9,708 (15.4%)	0.060	1,710 (13.9%)	1,655 (13.4%)	0.013
Missing	6 (0.0%)	101 (0.2%)	0.037	5 (0.0%)	6 (0.0%)	0.004
Dual status/Low income subsidy	3,108 (21.2%)	20,191 (32.0%)	0.244	2,843 (23.0%)	2,876 (23.3%)	0.006
**Lifestyle risk factors**						
Smoking/Tobacco use	4,116 (28.1%)	17,742 (28.1%)	0.001	3,455 (28.0%)	3,489 (28.3%)	0.006
Weight						
Underweight	10 (0.1%)	86 (0.1%)	0.021	9 (0.1%)	9 (0.1%)	0.000
Normal weight	77 (0.5%)	1,012 (1.6%)	0.105	73 (0.6%)	84 (0.7%)	0.011
Overweight (BMI 25.0-29.9)	1,829 (12.5%)	13,555 (21.5%)	0.240	1,718 (13.9%)	1,645 (13.3%)	0.017
Class 1 Obesity (BMI 30.0-34.9)	1,435 (9.8%)	7,601 (12.0%)	0.071	1,307 (10.6%)	1,281 (10.4%)	0.007
Class 2 Obesity (BMI 35.0-39.9)	936 (6.4%)	3,537 (5.6%)	0.034	805 (6.5%)	813 (6.6%)	0.003
Class 3 Obesity; (BMI 40.0 and above)	4,406 (30.1%)	12,586 (19.9%)	0.237	3,418 (27.7%)	3,484 (28.2%)	0.012
Unspecified Obesity	5,942 (40.6%)	24,814 (39.3%)	0.027	5,009 (40.6%)	5,023 (40.7%)	0.002
**Diabetes complications**						
Diabetic retinopathy	1,981 (13.5%)	6,446 (10.2%)	0.103	1,534 (12.4%)	1,532 (12.4%)	0.000
Diabetic neuropathy	5,175 (35.4%)	18,998 (30.1%)	0.113	4,131 (33.5%)	4,134 (33.5%)	0.001
Diabetic nephropathy	4,269 (29.2%)	17,187 (27.2%)	0.044	3,443 (27.9%)	3,418 (27.7%)	0.005
Diabetes with other ophthalmic complications	404 (2.8%)	1,808 (2.9%)	0.006	340 (2.8%)	331 (2.7%)	0.004
Diabetes with peripheral circulatory disorders	2,108 (14.4%)	8,178 (12.9%)	0.043	1,665 (13.5%)	1,678 (13.6%)	0.003
Diabetic foot	567 (3.9%)	2,632 (4.2%)	0.015	476 (3.9%)	474 (3.8%)	0.001

Erectile dysfunction	356 (2.4%)	1,046 (1.7%)	0.055	285 (2.3%)	292 (2.4%)	0.004
Hypoglycemia	2,201 (15.0%)	8,382 (13.3%)	0.051	1,759 (14.3%)	1,824 (14.8%)	0.015
Hyperglycemia/DKA/HONK	8,546 (58.4%)	30,390 (48.1%)	0.208	6,843 (55.5%)	6,827 (55.3%)	0.003
Skin infections	1,835 (12.5%)	8,124 (12.9%)	0.010	1,499 (12.1%)	1,558 (12.6%)	0.015
Cardiovascular-related conditions						
Coronary atherosclerosis	5,363 (36.6%)	22,046 (34.9%)	0.037	4,357 (35.3%)	4,307 (34.9%)	0.008
Stable angina	993 (6.8%)	3,473 (5.5%)	0.054	786 (6.4%)	796 (6.5%)	0.003
Unstable angina	705 (4.8%)	2,847 (4.5%)	0.015	558 (4.5%)	569 (4.6%)	0.004
Hypertension	12,999 (88.8%)	56,887 (90.0%)	0.039	10,953 (88.8%)	10,965 (88.9%)	0.003
Hypotension	683 (4.7%)	3,687 (5.8%)	0.052	571 (4.6%)	559 (4.5%)	0.005
Hyperlipidemia	11,366 (77.7%)	48,759 (77.2%)	0.012	9,535 (77.3%)	9,522 (77.2%)	0.003
Acute MI	346 (2.4%)	1,567 (2.5%)	0.008	279 (2.3%)	297 (2.4%)	0.010
Old MI	1,206 (8.2%)	5,115 (8.1%)	0.005	997 (8.1%)	957 (7.8%)	0.012
Ischemic stroke	260 (1.8%)	1,900 (3.0%)	0.081	232 (1.9%)	256 (2.1%)	0.014
TIA	340 (2.3%)	2,087 (3.3%)	0.059	290 (2.4%)	299 (2.4%)	0.005
Cardiac conduction disorder	1,268 (8.7%)	6,039 (9.6%)	0.031	1,077 (8.7%)	1,056 (8.6%)	0.006
Previous cardiac procedure (CABG, PTCA, Stent)	598 (4.1%)	1,994 (3.2%)	0.050	470 (3.8%)	468 (3.8%)	0.001
PVD diagnosis or surgery	1,883 (12.9%)	8,676 (13.7%)	0.025	1,558 (12.6%)	1,551 (12.6%)	0.002
Atrial fibrillation	2,694 (18.4%)	12,711 (20.1%)	0.043	2,250 (18.2%)	2,257 (18.3%)	0.001
Other cardiac dysrhythmia	3,614 (24.7%)	16,974 (26.9%)	0.050	3,008 (24.4%)	3,048 (24.7%)	0.008
Heart failure	3,175 (21.7%)	14,709 (23.3%)	0.038	2,613 (21.2%)	2,573 (20.9%)	0.008
Acute heart failure	982 (6.7%)	5,350 (8.5%)	0.066	815 (6.6%)	815 (6.6%)	0.000
Cardiomyopathy	1,091 (7.5%)	4,617 (7.3%)	0.006	910 (7.4%)	859 (7.0%)	0.016
Valve disorders	2,123 (14.5%)	10,141 (16.0%)	0.043	1,774 (14.4%)	1,770 (14.3%)	0.001
Valve replacement	363 (2.5%)	1,507 (2.4%)	0.006	300 (2.4%)	309 (2.5%)	0.005
Edema	2,427 (16.6%)	10,680 (16.9%)	0.009	1,979 (16.0%)	1,998 (16.2%)	0.004
Venous thromboembolism / Pulmonary embolism	650 (4.4%)	3,010 (4.8%)	0.015	548 (4.4%)	534 (4.3%)	0.006
Pulmonary hypertension	590 (4.0%)	3,165 (5.0%)	0.047	498 (4.0%)	489 (4.0%)	0.004
Implantable cardioverter defibrillator	81 (0.6%)	390 (0.6%)	0.008	75 (0.6%)	66 (0.5%)	0.010
Hyperkalemia	241 (1.6%)	1,349 (2.1%)	0.036	210 (1.7%)	217 (1.8%)	0.004
Cerebrovascular procedure	48 (0.3%)	265 (0.4%)	0.015	43 (0.3%)	56 (0.5%)	0.017
Insertion of pacemakers / removal of cardiac lead	53 (0.4%)	381 (0.6%)	0.035	43 (0.3%)	49 (0.4%)	0.008
Renal-related conditions						
Hypertensive nephropathy	2,924 (20.0%)	12,874 (20.4%)	0.010	2,386 (19.3%)	2,354 (19.1%)	0.007
CKD stage 1-2	628 (4.3%)	2,704 (4.3%)	0.001	516 (4.2%)	512 (4.1%)	0.002
CKD stage 3-4	3,364 (23.0%)	13,570 (21.5%)	0.036	2,708 (21.9%)	2,691 (21.8%)	0.003
Unspecified CKD	1,466 (10.0%)	6,892 (10.9%)	0.029	1,204 (9.8%)	1,196 (9.7%)	0.002
Microalbuminuria or proteinuria	685 (4.7%)	2,378 (3.8%)	0.046	543 (4.4%)	544 (4.4%)	0.000
Acute kidney injury	1,671 (11.4%)	9,378 (14.8%)	0.101	1,427 (11.6%)	1,459 (11.8%)	0.008

Urinary tract infections	2,823 (19.3%)	14,898 (23.6%)	0.105	2,452 (19.9%)	2,485 (20.1%)	0.007
Genital infections	434 (3.0%)	1,930 (3.1%)	0.005	356 (2.9%)	369 (3.0%)	0.006
Urolithiasis (Kidney and urinary stone)	762 (5.2%)	3,258 (5.2%)	0.002	649 (5.3%)	632 (5.1%)	0.006
**Other comorbidities**						
COPD	2,595 (17.7%)	12,124 (19.2%)	0.038	2,221 (18.0%)	2,195 (17.8%)	0.005
Asthma	1,622 (11.1%)	6,824 (10.8%)	0.009	1,350 (10.9%)	1,340 (10.9%)	0.003
Obstructive sleep apnea	4,895 (33.4%)	14,082 (22.3%)	0.251	3,811 (30.9%)	3,846 (31.2%)	0.006
Serious bacterial infections	775 (5.3%)	5,266 (8.3%)	0.121	688 (5.6%)	710 (5.8%)	0.008
Pneumonia	1,023 (7.0%)	5,836 (9.2%)	0.082	880 (7.1%)	861 (7.0%)	0.006
Liver disease	1,763 (12.0%)	6,984 (11.1%)	0.031	1,426 (11.6%)	1,419 (11.5%)	0.002
MASH / MASLD	938 (6.4%)	3,097 (4.9%)	0.065	746 (6.0%)	735 (6.0%)	0.004
Fractures / Falls	572 (3.9%)	3,040 (4.8%)	0.044	492 (4.0%)	475 (3.8%)	0.007
Osteoporosis	906 (6.2%)	4,553 (7.2%)	0.041	785 (6.4%)	785 (6.4%)	0.000
Osteoarthritis	5,334 (36.4%)	22,977 (36.4%)	0.002	4,491 (36.4%)	4,426 (35.9%)	0.011
Depression	2,995 (20.5%)	12,721 (20.1%)	0.008	2,508 (20.3%)	2,549 (20.7%)	0.008
Dementia	741 (5.1%)	6,422 (10.2%)	0.193	679 (5.5%)	697 (5.6%)	0.006
Delirium or psychosis	246 (1.7%)	1,963 (3.1%)	0.093	223 (1.8%)	241 (2.0%)	0.011
Anxiety	2,195 (15.0%)	9,888 (15.6%)	0.018	1,886 (15.3%)	1,885 (15.3%)	0.000
Sleep disorders	5,064 (34.6%)	22,151 (35.1%)	0.009	4,258 (34.5%)	4,286 (34.7%)	0.005
Anemia	3,645 (24.9%)	18,582 (29.4%)	0.101	3,116 (25.3%)	3,096 (25.1%)	0.004
Influenza	281 (1.9%)	1,699 (2.7%)	0.051	254 (2.1%)	259 (2.1%)	0.003
COVID	88 (0.6%)	306 (0.5%)	0.016	73 (0.6%)	69 (0.6%)	0.004
Hyperthyroidism and other thyroid gland disorders	4,302 (29.4%)	17,605 (27.9%)	0.034	3,594 (29.1%)	3,577 (29.0%)	0.003
Hypothyroidism	3,542 (24.2%)	14,477 (22.9%)	0.030	2,955 (23.9%)	2,945 (23.9%)	0.002
Nephrotic syndrome	15 (0.1%)	55 (0.1%)	0.005	13 (0.1%)	13 (0.1%)	0.000
Urinary incontinence	922 (6.3%)	4,406 (7.0%)	0.027	793 (6.4%)	801 (6.5%)	0.003
Biliary disease	39 (0.3%)	282 (0.4%)	0.030	31 (0.3%)	30 (0.2%)	0.002
Pancreatitis	15 (0.1%)	149 (0.2%)	0.032	14 (0.1%)	12 (0.1%)	0.005
Bowel obstruction	36 (0.2%)	167 (0.3%)	0.004	29 (0.2%)	34 (0.3%)	0.008
Gastroparesis	107 (0.7%)	533 (0.8%)	0.013	89 (0.7%)	89 (0.7%)	0.000
**Diabetes medications**						
Number of antidiabetic drugs on CED; mean (SD) median [IQR]	2.32 ±0.90 2.00 [2.00, 3.00]	2.19 ±0.83 2.00 [2.00, 3.00]	0.143	2.28 ±0.89 2.00 [2.00, 3.00]	2.29 ±0.89 2.00 [2.00, 3.00]	0.016
Concomitant use or initiation of Metformin	7,195 (49.2%)	36,599 (57.9%)	0.176	6,364 (51.6%)	6,341 (51.4%)	0.004
Concomitant use or initiation of Insulins	5,854 (40.0%)	10,558 (16.7%)	0.535	4,126 (33.4%)	4,156 (33.7%)	0.005
Concomitant use or initiation of Sulfonylureas	3,386 (23.1%)	20,522 (32.5%)	0.210	3,097 (25.1%)	3,182 (25.8%)	0.016
Concomitant use or initiation of SGLT-2i	1,880 (12.8%)	3,585 (5.7%)	0.249	1,361 (11.0%)	1,408 (11.4%)	0.012
Concomitant use or initiation of Any other glucose-lowering drugs	975 (6.7%)	4,172 (6.6%)	0.002	825 (6.7%)	862 (7.0%)	0.012
Past use of Metformin	9,505 (64.9%)	42,319 (67.0%)	0.043	8,174 (66.2%)	8,183 (66.3%)	0.002
Past use of Insulins	7,460 (51.0%)	14,679 (23.2%)	0.600	5,377 (43.6%)	5,415 (43.9%)	0.006

Past use of Sulfonylureas	4,856 (33.2%)	26,424 (41.8%)	0.179	4,337 (35.1%)	4,447 (36.0%)	0.019
Past use of SGLT-2i	2,757 (18.8%)	5,619 (8.9%)	0.291	2,018 (16.4%)	2,061 (16.7%)	0.009
Past use of Any other glucose-lowering drugs	1,568 (10.7%)	6,211 (9.8%)	0.029	1,303 (10.6%)	1,381 (11.2%)	0.020
Other medications						
ACE / ARB	11,563 (79.0%)	49,068 (77.7%)	0.033	9,678 (78.4%)	9,764 (79.1%)	0.017
ARNI	213 (1.5%)	678 (1.1%)	0.034	177 (1.4%)	154 (1.2%)	0.016
Thiazides	5,334 (36.4%)	22,378 (35.4%)	0.022	4,473 (36.3%)	4,481 (36.3%)	0.001
Beta-blockers	8,676 (59.3%)	36,685 (58.1%)	0.025	7,158 (58.0%)	7,143 (57.9%)	0.002
Calcium channel blockers	5,554 (38.0%)	25,814 (40.9%)	0.059	4,705 (38.1%)	4,722 (38.3%)	0.003
Digoxin / Digitoxin	309 (2.1%)	1,667 (2.6%)	0.035	262 (2.1%)	244 (2.0%)	0.010
Loop diuretics	5,125 (35.0%)	20,432 (32.3%)	0.057	4,101 (33.2%)	4,140 (33.6%)	0.007
Other diuretics	1,838 (12.6%)	6,557 (10.4%)	0.069	1,437 (11.6%)	1,458 (11.8%)	0.005
Intravenous diuretics	384 (2.6%)	1,769 (2.8%)	0.011	311 (2.5%)	312 (2.5%)	0.001
Nitrates	1,995 (13.6%)	7,852 (12.4%)	0.036	1,605 (13.0%)	1,612 (13.1%)	0.002
Anti-arrhythmics	620 (4.2%)	2,921 (4.6%)	0.019	525 (4.3%)	540 (4.4%)	0.006
Statins	12,348 (84.4%)	51,100 (80.9%)	0.093	10,319 (83.6%)	10,340 (83.8%)	0.005
PCSK9 inhibitors and other lipid-lowering drugs	2,412 (16.5%)	8,635 (13.7%)	0.079	1,910 (15.5%)	1,931 (15.6%)	0.005
Antiplatelet medications	2,571 (17.6%)	10,621 (16.8%)	0.020	2,094 (17.0%)	2,114 (17.1%)	0.004
Oral anticoagulants	2,721 (18.6%)	11,779 (18.6%)	0.001	2,270 (18.4%)	2,281 (18.5%)	0.002
COPD / Asthma medications	5,611 (38.3%)	23,046 (36.5%)	0.039	4,685 (38.0%)	4,706 (38.1%)	0.004
NSAIDS	4,576 (31.3%)	19,281 (30.5%)	0.016	3,891 (31.5%)	3,948 (32.0%)	0.010
Oral corticosteroids	3,552 (24.3%)	14,565 (23.0%)	0.029	2,964 (24.0%)	2,995 (24.3%)	0.006
Osteoporosis agents (incl. bisphosphonates)	501 (3.4%)	2,766 (4.4%)	0.049	446 (3.6%)	456 (3.7%)	0.004
Opioids	5,985 (40.9%)	23,761 (37.6%)	0.067	4,939 (40.0%)	4,980 (40.4%)	0.007
Anti-depressants	6,226 (42.5%)	23,093 (36.5%)	0.123	5,107 (41.4%)	5,176 (41.9%)	0.011
Antipsychotics	602 (4.1%)	3,060 (4.8%)	0.035	518 (4.2%)	510 (4.1%)	0.003
Anxiolytics / hypnotics, benzos	3,248 (22.2%)	13,290 (21.0%)	0.028	2,713 (22.0%)	2,782 (22.5%)	0.013
Dementia medications	450 (3.1%)	3,407 (5.4%)	0.115	405 (3.3%)	381 (3.1%)	0.011
Urinary tract infections antibiotics	8,134 (55.6%)	33,868 (53.6%)	0.040	6,804 (55.1%)	6,873 (55.7%)	0.011
Laxatives	614 (4.2%)	3,633 (5.7%)	0.072	518 (4.2%)	535 (4.3%)	0.007
Healthcare utilization marker						
Number of distinct medications; mean (SD) median [IQR]	16.28 ±6.83 15.00 [11.00, 20.00]	14.85 ±6.86 14.00 [10.00, 19.00]	0.209	15.84 ±6.71 15.00 [11.00, 20.00]	15.96 ±7.01 15.00 [11.00, 20.00]	0.016
Number of office visits; mean (SD) median [IQR]	13.48 ±8.56 12.00 [7.00, 18.00]	11.68 ±8.17 10.00 [6.00, 16.00]	0.216	12.96 ±8.16 11.00 [7.00, 17.00]	13.00 ±9.01 11.00 [7.00, 17.00]	0.004
Number of endocrinologist visits; mean (SD) median [IQR]	0.96 ±1.99 0.00 [0.00, 1.00]	0.40 ±1.36 0.00 [0.00, 0.00]	0.326	0.75 ±1.64 0.00 [0.00, 1.00]	0.72 ±2.08 0.00 [0.00, 0.00]	0.014
Number of cardiologist visits; mean (SD) median [IQR]	2.17 ±3.74 1.00 [0.00, 3.00]	2.35 ±4.00 1.00 [0.00, 3.00]	0.046	2.14 ±3.75 1.00 [0.00, 3.00]	2.15 ±3.63 1.00 [0.00, 3.00]	0.003
Number of internal/family medicine visits; mean (SD) median [IQR]	6.48 ±6.65 5.00 [2.00, 9.00]	7.29 ±8.08 5.00 [2.00, 10.00]	0.110	6.52 ±6.74 5.00 [2.00, 9.00]	6.52 ±6.87 5.00 [2.00, 9.00]	0.000
Number of Electrocardiograms (ECG/EKG) ; mean (SD) median [IQR]	1.53 ±2.19 1.00 [0.00, 2.00]	1.74 ±2.46 1.00 [0.00, 2.00]	0.092	1.53 ±2.22 1.00 [0.00, 2.00]	1.54 ±2.13 1.00 [0.00, 2.00]	0.004
Number of Echocardiograms; mean (SD) median [IQR]	0.49 ±0.86 0.00 [0.00, 1.00]	0.53 ±0.92 0.00 [0.00, 1.00]	0.051	0.48 ±0.86 0.00 [0.00, 1.00]	0.48 ±0.88 0.00 [0.00, 1.00]	0.004

Out-of-pocket medication cost; mean (SD) median [IQR]	1,070.64 ±1,052.97 767.08 [221.78, 1,614.22]	677.14 ±841.58 424.38 [97.40, 952.26]	0.413	949.95 ±941.67 682.14 [188.49, 1,376.07]	950.03 ±1,059.80 698.52 [176.09, 1,372.64]	0.000
Unique brand medicines; mean (SD) median [IQR]	16.68 ±7.13 16.00 [12.00, 21.00]	15.18 ±7.13 14.00 [10.00, 19.00]	0.212	16.23 ±7.02 15.00 [11.00, 20.00]	16.34 ±7.32 15.00 [11.00, 20.00]	0.016
Unique generic medicines; mean (SD) median [IQR]	16.28 ±6.83 15.00 [11.00, 20.00]	14.85 ±6.86 14.00 [10.00, 19.00]	0.209	15.84 ±6.71 15.00 [11.00, 20.00]	15.96 ±7.01 15.00 [11.00, 20.00]	0.016
Ratio of brand to generic medications; mean (SD) median [IQR]	1.02 ±0.04 1.00 [1.00, 1.04]	1.02 ±0.04 1.00 [1.00, 1.00]	0.072	1.02 ±0.04 1.00 [1.00, 1.04]	1.02 ±0.04 1.00 [1.00, 1.04]	0.006
**Healthy behavior markers**						
Colonoscopy / Sigmoidoscopy	1,697 (11.6%)	6,380 (10.1%)	0.048	1,368 (11.1%)	1,376 (11.2%)	0.002
Flu Pneumococcal vaccine	1,593 (10.9%)	7,895 (12.5%)	0.050	1,349 (10.9%)	1,376 (11.2%)	0.007
Pap smear	700 (4.8%)	2,376 (3.8%)	0.051	578 (4.7%)	640 (5.2%)	0.023
PSA test	3,321 (22.7%)	12,330 (19.5%)	0.078	2,740 (22.2%)	2,692 (21.8%)	0.009
Fecal occult blood test	897 (6.1%)	4,720 (7.5%)	0.053	789 (6.4%)	788 (6.4%)	0.000
Bone mineral density tests	1,397 (9.5%)	5,240 (8.3%)	0.044	1,162 (9.4%)	1,169 (9.5%)	0.002
Mammograms	3,463 (23.7%)	12,802 (20.3%)	0.082	2,874 (23.3%)	2,938 (23.8%)	0.012
Telemedicine	2,322 (15.9%)	4,273 (6.8%)	0.290	1,716 (13.9%)	1,685 (13.7%)	0.007
**Laboratory and diagnostic tests**						
HbA1c tests; mean (SD) median [IQR]	2.96 ±2.04 3.00 [2.00, 4.00]	2.71 ±2.84 3.00 [2.00, 3.00]	0.102	2.89 ±1.93 3.00 [2.00, 4.00]	2.90 ±3.32 3.00 [2.00, 4.00]	0.003
Lipid panels; mean (SD) median [IQR]	1.92 ±1.74 2.00 [1.00, 3.00]	1.82 ±2.15 2.00 [1.00, 2.00]	0.052	1.91 ±1.78 2.00 [1.00, 3.00]	1.93 ±2.68 2.00 [1.00, 3.00]	0.007
Creatinine tests; mean (SD) median [IQR]	0.10 ±0.53 0.00 [0.00, 0.00]	0.11 ±0.78 0.00 [0.00, 0.00]	0.022	0.09 ±0.51 0.00 [0.00, 0.00]	0.09 ±0.49 0.00 [0.00, 0.00]	0.005
Natriuretic peptide tests; mean (SD) median [IQR]	0.38 ±1.64 0.00 [0.00, 0.00]	0.40 ±1.88 0.00 [0.00, 0.00]	0.011	0.37 ±1.63 0.00 [0.00, 0.00]	0.38 ±1.72 0.00 [0.00, 0.00]	0.004
Urine tests; mean (SD) median [IQR]	1.60 ±2.82 1.00 [0.00, 2.00]	1.73 ±3.30 1.00 [0.00, 2.00]	0.043	1.61 ±2.91 1.00 [0.00, 2.00]	1.61 ±2.82 1.00 [0.00, 2.00]	0.001
**Burden of comorbidities**						
Combined comorbidity score; mean (SD) median [IQR]	2.66 ±2.58 2.00 [1.00, 4.00]	2.88 ±2.95 2.00 [1.00, 5.00]	0.079	2.62 ±2.61 2.00 [1.00, 4.00]	2.62 ±2.60 2.00 [1.00, 4.00]	0.003
Frailty Score; mean (SD) median [IQR]	0.22 ±0.07 0.20 [0.17, 0.25]	0.22 ±0.08 0.21 [0.17, 0.26]	0.099	0.22 ±0.07 0.20 [0.17, 0.25]	0.22 ±0.07 0.20 [0.16, 0.25]	0.001
**Baseline hospitalizations and hospital metrics**						
Number of Hospitalizations; mean (SD) median [IQR]	0.41 ±0.89 0.00 [0.00, 1.00]	0.61 ±1.19 0.00 [0.00, 1.00]	0.189	0.43 ±0.92 0.00 [0.00, 1.00]	0.43 ±0.91 0.00 [0.00, 1.00]	0.004
Any hospitalization within prior 91 days	1,147 (7.8%)	9,448 (15.0%)	0.225	1,061 (8.6%)	1,072 (8.7%)	0.003
Any hospitalization within prior 92-365 days	3,167 (21.6%)	15,731 (24.9%)	0.077	2,667 (21.6%)	2,691 (21.8%)	0.005
Number of hospitalizations (0, 1, 2 or more)						
< 1	10,868 (74.3%)	42,437 (67.2%)	0.157	9,101 (73.8%)	9,065 (73.5%)	0.007
1 - <2	2,468 (16.9%)	11,810 (18.7%)	0.048	2,094 (17.0%)	2,099 (17.0%)	0.001
>= 2	1,299 (8.9%)	8,944 (14.2%)	0.166	1,144 (9.3%)	1,175 (9.5%)	0.009
Heart failure hospitalization	1,429 (9.8%)	8,052 (12.7%)	0.094	1,216 (9.9%)	1,191 (9.7%)	0.007
ED visit	6,284 (42.9%)	31,534 (49.9%)	0.140	5,341 (43.3%)	5,393 (43.7%)	0.009
**Calendar year of cohort entry**						
2018	1,056 (7.2%)	24,236 (38.4%)	0.799	1,052 (8.5%)	1,103 (8.9%)	0.015
2019	5,831 (39.8%)	22,383 (35.4%)	0.091	5,189 (42.1%)	5,177 (42.0%)	0.002
2020	7,748 (52.9%)	16,572 (26.2%)	0.568	6,098 (49.4%)	6,059 (49.1%)	0.006

Abbreviations: *not used in the propensity score; ACE, angiotensin-converting enzyme inhibitors; ARB, angiotensin receptor blocker; ARNI, angiotensin receptor/neprilysin inhibitor; BMI, body mass index; CABG, coronary artery bypass graft surgery; CED, cohort entry date; CKD, chronic kidney disease; COPD, chronic obstructive pulmonary disease; DKA, diabetic ketoacidosis; DPP4i, dipeptidyl peptidase-4 inhibitors; ED, emergency department; eGFR, estimated glomerular filtration rate (estimated using the quadratic GFR equation: GFR = EXP(1.911 + (5.249 / Serum creatinine) - (2.114 / (Serum creatinine^2)) - (0.00686 * Age) - 0.205 (if female))); HbA1c, hemoglobin A1c; HDL, high-density lipoprotein cholesterol; HONK, hyperglycemic hyperosmolar nonketotic state; IQR, inter-quartile-range; MASH, metabolic dysfunction associated steatohepatitis; MASLD, metabolic dysfunction associated steatotic liver disease; MI, myocardial infarction; N, number of participants; NSAIDs, non-steroidal anti-inflammatory drugs; PCSK9, proprotein convertase subtilisin/kexin type 9; PSA, prostate-specific antigen; PTCA, percutaneous transluminal coronary angioplasty; SGLT2i, sodium-glucose transport protein 2 inhibitors; SD, standard deviation; SMD, standardized mean difference; TIA, transient ischemic attack.
Missing data were handled by assuming absence of a code indicated absence of the condition for most binary covariates. Missing indicators were included for race and region in the propensity score model.
The Race-Others category includes Asian and Hispanic individuals.

Supplementary Table 10. Baseline characteristics of initiators of tirzepatide vs dulaglutide when applying trial eligibility criteria before and after propensity score matching, pooled across database. Values are number (percentage) unless otherwise specified.

	Before propensity score matching			After propensity score matching		
Variable	Tirzepatide (n = 25,377)	Dulaglutide (n = 19,294)	SMD	Tirzepatide (n = 12,827)	Dulaglutide (n = 12,827)	SMD
Demographics						
Age; mean (SD) median [IQR]	65.50 ± 9.63	67.65 ± 9.80	0.221	66.53 ± 9.58	66.53 ± 9.79	0.001
Gender						
Male	12,121 (47.8%)	9,755 (50.6%)	0.056	6,353 (49.5%)	6,360 (49.6%)	0.001
Female	13,256 (52.2%)	9,539 (49.4%)	0.056	6,474 (50.5%)	6,467 (50.4%)	0.001
Race						
White	10,227 (50.5%)	6,463 (46.3%)	0.141	4,596 (48.2%)	4,570 (48.0%)	0.004
Black	2,567 (12.7%)	2,182 (15.6%)	0.039	1,402 (14.7%)	1,373 (14.4%)	0.007
Unknown / Missing	7,229 (35.7%)	5,115 (36.6%)	0.044	3,411 (35.8%)	3,468 (36.4%)	0.010
Others	240 (1.2%)	210 (1.5%)	0.014	121 (1.3%)	119 (1.3%)	0.002
Region / State						
Northeast	2,203 (8.7%)	2,137 (11.1%)	0.080	1,283 (10.0%)	1,255 (9.8%)	0.007
Midwest / North central	4,997 (19.7%)	5,045 (26.1%)	0.154	2,989 (23.3%)	3,003 (23.4%)	0.003
South	15,371 (60.6%)	9,256 (48.0%)	0.255	6,841 (53.3%)	6,841 (53.3%)	0.000
West	2,791 (11.0%)	2,844 (14.7%)	0.112	1,706 (13.3%)	1,719 (13.4%)	0.003
Missing	15 (0.1%)	12 (0.1%)	0.001	8 (0.1%)	9 (0.1%)	0.003
Lifestyle risk factors						
Smoking / Tobacco use	6,625 (26.1%)	5,193 (26.9%)	0.018	3,389 (26.4%)	3,382 (26.4%)	0.001
Weight						
Overweight (25.0-29.9)	1,786 (7.0%)	2,532 (13.1%)	0.203	1,297 (10.1%)	1,286 (10.0%)	0.003
Class 1 Obesity (30.0-34.9)	4,486 (17.7%)	4,047 (21.0%)	0.084	2,551 (19.9%)	2,553 (19.9%)	0.000
Class 2 Obesity (35.0-39.9)	7,527 (29.7%)	4,823 (25.0%)	0.105	3,457 (27.0%)	3,462 (27.0%)	0.001
Class 3 Obesity (40.0 and above)	6,232 (24.6%)	3,808 (19.7%)	0.116	2,638 (20.6%)	2,669 (20.8%)	0.006
Unspecified Obesity	5,346 (21.1%)	4,084 (21.2%)	0.002	2,884 (22.5%)	2,857 (22.3%)	0.005
Diabetes complications						
Diabetic retinopathy	2,887 (11.4%)	3,061 (15.9%)	0.131	1,818 (14.2%)	1,800 (14.0%)	0.004
Diabetic neuropathy	8,605 (33.9%)	8,171 (42.3%)	0.174	5,014 (39.1%)	4,974 (38.8%)	0.006
Diabetic nephropathy	6,549 (25.8%)	6,528 (33.8%)	0.176	3,885 (30.3%)	3,869 (30.2%)	0.003
Diabetes with other ophthalmic complications	1,027 (4.0%)	987 (5.1%)	0.051	596 (4.6%)	599 (4.7%)	0.001
Diabetes with peripheral circulatory disorders	5,219 (20.6%)	4,999 (25.9%)	0.127	3,017 (23.5%)	2,983 (23.3%)	0.006
Diabetic foot	1,111 (4.4%)	1,128 (5.8%)	0.067	677 (5.3%)	666 (5.2%)	0.004
Erectile dysfunction	1,396 (5.5%)	988 (5.1%)	0.017	715 (5.6%)	693 (5.4%)	0.008
Hypoglycemia	6,668 (26.3%)	5,633 (29.2%)	0.065	3,573 (27.9%)	3,580 (27.9%)	0.001
Hyperglycemia / DKA / HONK	13,542 (53.4%)	11,853 (61.4%)	0.164	7,463 (58.2%)	7,468 (58.2%)	0.001

Skin infections	3,233 (12.7%)	2,611 (13.5%)	0.023	1,670 (13.0%)	1,660 (12.9%)	0.002
Cardiovascular-related conditions						
Coronary atherosclerosis	10,502 (41.4%)	8,468 (43.9%)	0.051	5,498 (42.9%)	5,463 (42.6%)	0.006
Stable angina	2,852 (11.2%)	2,156 (11.2%)	0.002	1,423 (11.1%)	1,417 (11.0%)	0.001
Unstable angina	1,274 (5.0%)	1,004 (5.2%)	0.008	654 (5.1%)	641 (5.0%)	0.005
Hypertension	23,467 (92.5%)	18,049 (93.5%)	0.042	11,921 (92.9%)	11,931 (93.0%)	0.003
Hypotension	1,062 (4.2%)	1,101 (5.7%)	0.07	606 (4.7%)	618 (4.8%)	0.004
Hyperlipidemia	22,739 (89.6%)	17,290 (89.6%)	0.000	11,477 (89.5%)	11,475 (89.5%)	0.001
Acute MI	497 (2.0%)	504 (2.6%)	0.044	285 (2.2%)	289 (2.3%)	0.002
Old MI	2,174 (8.6%)	1,945 (10.1%)	0.052	1,192 (9.3%)	1,191 (9.3%)	0.000
Ischemic stroke	291 (1.1%)	406 (2.1%)	0.076	192 (1.5%)	204 (1.6%)	0.008
TIA	824 (3.2%)	761 (3.9%)	0.037	449 (3.5%)	447 (3.5%)	0.001
Cardiac conduction disorder	2,232 (8.8%)	1,956 (10.1%)	0.046	1,162 (9.1%)	1,160 (9.0%)	0.001
Previous cardiac procedure (CABG, PTCA, Stent)	822 (3.2%)	718 (3.7%)	0.026	453 (3.5%)	453 (3.5%)	0.000
PVD diagnosis or surgery	5,452 (21.5%)	4,697 (24.3%)	0.068	2,934 (22.9%)	2,888 (22.5%)	0.009
Atrial fibrillation	3,995 (15.7%)	3,171 (16.4%)	0.019	2,001 (15.6%)	2,037 (15.9%)	0.008
Other cardiac dysrhythmia	7,299 (28.8%)	5,580 (28.9%)	0.004	3,568 (27.8%)	3,576 (27.9%)	0.001
Heart failure	5,470 (21.6%)	4,712 (24.4%)	0.068	2,907 (22.7%)	2,901 (22.6%)	0.001
Acute heart failure	1,168 (4.6%)	1,154 (6.0%)	0.062	667 (5.2%)	671 (5.2%)	0.001
Cardiomyopathy	2,188 (8.6%)	1,961 (10.2%)	0.053	1,195 (9.3%)	1,217 (9.5%)	0.006
Valve disorders	4,402 (17.3%)	3,512 (18.2%)	0.022	2,233 (17.4%)	2,238 (17.4%)	0.001
Valve replacement	515 (2.0%)	432 (2.2%)	0.015	278 (2.2%)	272 (2.1%)	0.003
Edema	4,568 (18.0%)	3,581 (18.6%)	0.014	2,287 (17.8%)	2,262 (17.6%)	0.005
Venous thromboembolism / Pulmonary embolism	1,150 (4.5%)	950 (4.9%)	0.018	566 (4.4%)	569 (4.4%)	0.001
Pulmonary hypertension	1,075 (4.2%)	822 (4.3%)	0.001	497 (3.9%)	521 (4.1%)	0.010
Implantable cardioverter defibrillator	135 (0.5%)	126 (0.7%)	0.016	82 (0.6%)	79 (0.6%)	0.003
Hyperkalemia	927 (3.7%)	1,057 (5.5%)	0.088	528 (4.1%)	562 (4.4%)	0.013
Cerebrovascular procedure	47 (0.2%)	61 (0.3%)	0.026	31 (0.2%)	34 (0.3%)	0.005
Insertion of pacemakers / removal of cardiac lead	65 (0.3%)	60 (0.3%)	0.010	27 (0.2%)	32 (0.2%)	0.008
Renal-related conditions						
Hypertensive nephropathy	4,223 (16.6%)	4,237 (22.0%)	0.135	2,470 (19.3%)	2,494 (19.4%)	0.005
CKD Stage 1-2	1,466 (5.8%)	1,155 (6.0%)	0.009	772 (6.0%)	775 (6.0%)	0.001
CKD Stage 3-4	4,550 (17.9%)	4,124 (21.4%)	0.087	2,567 (20.0%)	2,564 (20.0%)	0.001
Unspecified CKD	1,991 (7.8%)	2,131 (11.0%)	0.110	1,220 (9.5%)	1,206 (9.4%)	0.004
Microalbuminuria or proteinuria	1,793 (7.1%)	1,576 (8.2%)	0.042	971 (7.6%)	993 (7.7%)	0.006
Acute kidney injury	1,954 (7.7%)	2,248 (11.7%)	0.134	1,206 (9.4%)	1,221 (9.5%)	0.004
Urinary tract infections	4,039 (15.9%)	3,253 (16.9%)	0.026	2,009 (15.7%)	2,015 (15.7%)	0.001
Genital infections	683 (2.7%)	654 (3.4%)	0.041	395 (3.1%)	403 (3.1%)	0.004
Urolithiasis (Kidney and urinary stone)	1,308 (5.2%)	940 (4.9%)	0.013	615 (4.8%)	614 (4.8%)	0.000

Other comorbidities						
COPD	4,096 (16.1%)	3,410 (17.7%)	0.041	2,171 (16.9%)	2,129 (16.6%)	0.009
Asthma	3,301 (13.0%)	2,140 (11.1%)	0.059	1,511 (11.8%)	1,485 (11.6%)	0.006
Obstructive sleep apnea	10,224 (40.3%)	6,411 (33.2%)	0.147	4,593 (35.8%)	4,573 (35.7%)	0.003
Serious bacterial infections	878 (3.5%)	1,027 (5.3%)	0.091	525 (4.1%)	545 (4.2%)	0.008
Pneumonia	1,354 (5.3%)	1,383 (7.2%)	0.076	759 (5.9%)	759 (5.9%)	0.000
Liver disease	4,731 (18.6%)	3,443 (17.8%)	0.021	2,292 (17.9%)	2,271 (17.7%)	0.004
MASH / MASLD	2,478 (9.8%)	1,480 (7.7%)	0.074	1,087 (8.5%)	1,070 (8.3%)	0.005
Fractures / Falls	1,559 (6.1%)	1,537 (8.0%)	0.071	917 (7.1%)	891 (6.9%)	0.008
Osteoporosis	1,212 (4.8%)	1,034 (5.4%)	0.027	623 (4.9%)	665 (5.2%)	0.015
Osteoarthritis	8,400 (33.1%)	6,197 (32.1%)	0.021	4,099 (32.0%)	4,120 (32.1%)	0.004
Depression	5,299 (20.9%)	4,350 (22.5%)	0.040	2,723 (21.2%)	2,700 (21.0%)	0.004
Dementia	1,073 (4.2%)	1,546 (8.0%)	0.158	725 (5.7%)	714 (5.6%)	0.004
Delirium or psychosis	328 (1.3%)	451 (2.3%)	0.078	224 (1.7%)	216 (1.7%)	0.005
Anxiety	5,835 (23.0%)	3,974 (20.6%)	0.058	2,693 (21.0%)	2,665 (20.8%)	0.005
Sleep disorders	9,615 (37.9%)	6,832 (35.4%)	0.051	4,607 (35.9%)	4,576 (35.7%)	0.005
Anemia	5,961 (23.5%)	4,994 (25.9%)	0.056	3,148 (24.5%)	3,128 (24.4%)	0.004
Influenza	517 (2.0%)	335 (1.7%)	0.022	242 (1.9%)	245 (1.9%)	0.002
COVID	3,093 (12.2%)	2,650 (13.7%)	0.046	1,646 (12.8%)	1,648 (12.8%)	0.000
Hyperthyroidism and other thyroid gland disorders	7,666 (30.2%)	5,362 (27.8%)	0.053	3,663 (28.6%)	3,661 (28.5%)	0.000
Hypothyroidism	6,046 (23.8%)	4,278 (22.2%)	0.039	2,925 (22.8%)	2,900 (22.6%)	0.005
Nephrotic syndrome	28 (0.1%)	34 (0.2%)	0.017	20 (0.2%)	20 (0.2%)	0.000
Urinary incontinence	1,677 (6.6%)	1,481 (7.7%)	0.041	894 (7.0%)	884 (6.9%)	0.003
Biliary disease	37 (0.1%)	57 (0.3%)	0.032	24 (0.2%)	22 (0.2%)	0.004
Pancreatitis	14 (0.1%)	12 (0.1%)	0.003	7 (0.1%)	7 (0.1%)	0.000
Bowel obstruction	49 (0.2%)	46 (0.2%)	0.010	27 (0.2%)	29 (0.2%)	0.003
Gastroparesis	265 (1.0%)	219 (1.1%)	0.009	150 (1.2%)	127 (1.0%)	0.017
Diabetes medications						
Number of antidiabetic drugs on CED, mean (SD)	2.15 ± 0.99	2.37 ± 1.01	0.222	2.31 ± 1.03	2.30 ± 1.00	0.007
Concomitant use or initiation of Metformin	10,999 (43.3%)	8,740 (45.3%)	0.039	5,864 (45.7%)	5,843 (45.6%)	0.003
Concomitant use or initiation of Insulins	5,944 (23.4%)	6,213 (32.2%)	0.197	3,721 (29.0%)	3,659 (28.5%)	0.011
Concomitant use or initiation of Sulfonylureas	3,668 (14.5%)	4,209 (21.8%)	0.192	2,427 (18.9%)	2,442 (19.0%)	0.003
Concomitant use or initiation of DPP-4i	1,673 (6.6%)	1,628 (8.4%)	0.070	970 (7.6%)	996 (7.8%)	0.008
Concomitant use or initiation of SGLT-2i	5,437 (21.4%)	4,487 (23.3%)	0.044	3,060 (23.9%)	3,022 (23.6%)	0.007
Concomitant use or initiation of Any other glucose-lowering drugs	1,422 (5.6%)	1,164 (6.0%)	0.018	759 (5.9%)	744 (5.8%)	0.005
Past use of Metformin	15,045 (59.3%)	11,986 (62.1%)	0.058	7,932 (61.8%)	7,911 (61.7%)	0.003
Past use of Insulins	7,754 (30.6%)	8,221 (42.6%)	0.252	4,879 (38.0%)	4,823 (37.6%)	0.009
Past use of Sulfonylureas	5,372 (21.2%)	5,889 (30.5%)	0.215	3,465 (27.0%)	3,468 (27.0%)	0.001
Past use of DPP-4i	2,622 (10.3%)	2,565 (13.3%)	0.092	1,523 (11.9%)	1,546 (12.1%)	0.006

Past use of SGLT-2i	7,431 (29.3%)	6,274 (32.5%)	0.070	4,217 (32.9%)	4,166 (32.5%)	0.008
Past use of Any other glucose-lowering drugs	2,060 (8.1%)	1,758 (9.1%)	0.035	1,126 (8.8%)	1,094 (8.5%)	0.009
Other medications						
ACE / ARB	18,459 (72.7%)	14,415 (74.7%)	0.045	9,448 (73.7%)	9,512 (74.2%)	0.011
ARNI	941 (3.7%)	662 (3.4%)	0.015	485 (3.8%)	480 (3.7%)	0.002
Thiazides	8,634 (34.0%)	6,086 (31.5%)	0.053	4,178 (32.6%)	4,142 (32.3%)	0.006
Beta-blockers	14,550 (57.3%)	11,812 (61.2%)	0.079	7,621 (59.4%)	7,653 (59.7%)	0.005
Calcium channel blockers	9,146 (36.0%)	7,490 (38.8%)	0.057	4,834 (37.7%)	4,802 (37.4%)	0.005
Digoxin / Digitoxin	218 (0.9%)	219 (1.1%)	0.028	118 (0.9%)	116 (0.9%)	0.002
Loop diuretics	6,682 (26.3%)	5,451 (28.3%)	0.043	3,466 (27.0%)	3,436 (26.8%)	0.005
Other diuretics	3,034 (12.0%)	2,195 (11.4%)	0.018	1,496 (11.7%)	1,511 (11.8%)	0.004
Intravenous diuretics	502 (2.0%)	421 (2.2%)	0.014	268 (2.1%)	262 (2.0%)	0.003
Nitrates	3,224 (12.7%)	2,830 (14.7%)	0.057	1,768 (13.8%)	1,753 (13.7%)	0.003
Anti-arrhythmics	1,021 (4.0%)	692 (3.6%)	0.023	452 (3.5%)	472 (3.7%)	0.008
Statins	20,721 (81.7%)	16,676 (86.4%)	0.131	10,870 (84.7%)	10,868 (84.7%)	0.000
PCSK9 inhibitors and other lipid-lowering drugs	4,828 (19.0%)	3,423 (17.7%)	0.033	2,380 (18.6%)	2,371 (18.5%)	0.002
Antiplatelet medications	5,256 (20.7%)	4,529 (23.5%)	0.067	2,878 (22.4%)	2,882 (22.5%)	0.001
Oral anticoagulants	4,038 (15.9%)	3,298 (17.1%)	0.032	2,077 (16.2%)	2,071 (16.1%)	0.001
COPD / Asthma medications	10,436 (41.1%)	7,245 (37.6%)	0.073	4,958 (38.7%)	4,973 (38.8%)	0.002
NSAIDS	8,079 (31.8%)	5,495 (28.5%)	0.073	3,832 (29.9%)	3,822 (29.8%)	0.002
Oral corticosteroids	7,421 (29.2%)	4,561 (23.6%)	0.127	3,282 (25.6%)	3,273 (25.5%)	0.002
Osteoporosis agents (incl. bisphosphonates)	749 (3.0%)	630 (3.3%)	0.018	412 (3.2%)	401 (3.1%)	0.005
Opioids	6,551 (25.8%)	4,716 (24.4%)	0.032	3,167 (24.7%)	3,186 (24.8%)	0.003
Anti-depressants	10,526 (41.5%)	7,713 (40.0%)	0.031	5,169 (40.3%)	5,147 (40.1%)	0.003
Antipsychotics	1,102 (4.3%)	970 (5.0%)	0.032	587 (4.6%)	581 (4.5%)	0.002
Anxiolytics / hypnotics, benzos	6,117 (24.1%)	3,758 (19.5%)	0.112	2,709 (21.1%)	2,641 (20.6%)	0.013
Dementia medications	446 (1.8%)	642 (3.3%)	0.100	296 (2.3%)	315 (2.5%)	0.010
Urinary tract infections antibiotics	13,149 (51.8%)	9,395 (48.7%)	0.062	6,310 (49.2%)	6,316 (49.2%)	0.001
Laxatives	994 (3.9%)	756 (3.9%)	0.000	500 (3.9%)	508 (4.0%)	0.003
Healthcare utilization marker						
Number of distinct medications; mean (SD)	16.17 ±6.98	16.54 ±6.92	0.053	16.42 ±6.86	16.36 ±6.95	0.010
Number of office visits; mean (SD)	11.69 ±7.53	11.21 ±7.49	0.064	11.37 ±7.19	11.32 ±7.59	0.006
Number of endocrinologist visits; mean (SD)	0.54 ±1.50	0.63 ±1.73	0.055	0.58 ±1.56	0.58 ±1.61	0.000
Number of cardiologist visits; mean (SD)	2.61 ±4.11	2.63 ±4.43	0.002	2.53 ±4.06	2.53 ±4.25	0.001
Number of internal/family medicine visits; mean (SD)	7.72 ±8.48	9.06 ±11.03	0.137	8.25 ±9.33	8.23 ±9.73	0.003
Number of electrocardiograms (ECG/EKG); mean (SD)	1.41 ±1.96	1.48 ±2.25	0.033	1.42 ±1.90	1.42 ±1.99	0.000
Number of echocardiograms; mean (SD)	0.47 ±0.78	0.48 ±0.86	0.012	0.47 ±0.76	0.47 ±0.87	0.000
Out-of-pocket medication cost; mean (SD)	691.43 ±1021.02	672.78 ±934.38	0.019	689.65 ±833.32	686.45 ±964.74	0.004
Unique brand medicines; mean (SD)	16.45 ±7.20	16.89 ±7.20	0.061	16.74 ±7.10	16.66 ±7.20	0.011

Unique generic medicines; mean (SD)	16.17 ±6.98	16.54 ±6.92	0.053	16.42 ±6.86	16.36 ±6.95	0.010
Ratio of brand to generic medications; mean (SD)	1.02 ±0.03	1.02 ±0.04	0.000	1.02 ±0.04	1.02 ±0.04	0.000
**Healthy behavior markers**						
Colonoscopy / Sigmoidoscopy	3,013 (11.9%)	2,047 (10.6%)	0.040	1,452 (11.3%)	1,446 (11.3%)	0.001
Flu Pneumococcal vaccine	7,857 (31.0%)	6,899 (35.8%)	0.102	4,260 (33.2%)	4,381 (34.2%)	0.020
Pap smear	1,599 (6.3%)	834 (4.3%)	0.088	641 (5.0%)	650 (5.1%)	0.003
PSA test	6,788 (26.7%)	4,649 (24.1%)	0.061	3,210 (25.0%)	3,245 (25.3%)	0.006
Fecal occult blood test	1,079 (4.3%)	920 (4.8%)	0.025	592 (4.6%)	581 (4.5%)	0.004
Bone mineral density tests	2,064 (8.1%)	1,382 (7.2%)	0.037	958 (7.5%)	963 (7.5%)	0.001
Mammograms	6,631 (26.1%)	4,165 (21.6%)	0.107	3,019 (23.5%)	2,996 (23.4%)	0.004
Telemedicine	6,450 (25.4%)	4,900 (25.4%)	0.000	3,180 (24.8%)	3,176 (24.8%)	0.001
**Laboratory and diagnostic tests**						
HbA1c tests; mean (SD)	2.47 ± 1.40	2.63 ± 1.40	0.114	2.59 ± 1.46	2.58 ± 1.37	0.007
Lipid panels; mean (SD)	1.80 ± 1.29	1.68 ± 1.26	0.094	1.74 ± 1.24	1.74 ± 1.27	0.002
Creatinine tests; mean (SD)	2.84 ± 3.41	2.86 ± 3.95	0.005	2.79 ± 3.75	2.79 ± 3.30	0.000
Natriuretic peptide tests; mean (SD)	0.26 ± 0.86	0.27 ± 0.86	0.017	0.25 ± 0.82	0.26 ± 0.85	0.012
Urine tests; mean (SD)	1.27 ± 1.84	1.25 ± 1.88	0.007	1.23 ± 1.82	1.24 ± 1.89	0.004
**Lab values**						
HbA1c (%)*; mean (SD) median [IQR]	7.61 ±1.66 7.20 [6.40, 8.40]	8.21 ±1.78 7.90 [7.00, 9.10]	0.351	7.87 ±1.72 7.50 [6.60, 8.80]	8.08 ±1.75 7.80 [6.80, 9.00]	0.124
Glucose (mg/dl)*; mean (SD) median [IQR]	154.58 ±66.18 137.00 [110.00, 179.50]	1,380.56 ±109,788.81 157.00 [123.00, 208.00]	0.016	162.30 ±69.35 145.75 [114.62, 191.00]	1,896.01 ±131,292.86 154.50 [120.00, 205.00]	0.019
Creatinine (mg/dl)*; mean (SD) median [IQR]	1.07 ±0.40 0.96 [0.80, 1.19]	1.15 ±1.42 1.00 [0.81, 1.27]	0.072	1.11 ±0.39 0.99 [0.81, 1.23]	1.12 ±1.07 0.98 [0.80, 1.24]	0.016
Systolic blood pressure (mmHg)*; mean (SD) median [IQR]	129.55 ±22.93 130.00 [120.00, 139.00]	130.42 ±28.37 130.00 [120.00, 140.00]	0.034	129.98 ±26.70 130.00 [120.00, 139.92]	130.00 ±28.46 130.00 [120.00, 139.50]	0.001
Heart rate (1/min)*; mean (SD) median [IQR]	76.70 ±16.10 76.00 [67.00, 85.00]	77.34 ±20.43 76.00 [68.00, 86.00]	0.035	76.69 ±19.05 76.00 [67.00, 84.00]	77.27 ±22.50 76.00 [68.00, 85.00]	0.028
BMI (kg/m2)*; mean (SD) median [IQR]	37.91 ±7.65 36.80 [32.42, 41.84]	35.52 ±7.12 34.30 [30.40, 39.10]	0.324	36.89 ±7.51 35.74 [31.50, 40.70]	35.87 ±7.07 34.70 [30.90, 39.56]	0.140
eGFR (ml/min/1.73m2)*; mean (SD) median [IQR]	980.17 ±95,276.29 74.00 [56.00, 91.00]	1,677.75 ±126,856.10 69.00 [50.00, 88.00]	0.006	69.69 ±22.85 71.00 [52.50, 89.00]	2,210.54 ±146,316.06 71.00 [52.10, 89.00]	0.021
LDL (mg/dl)*; mean (SD) median [IQR]	80.96 ±37.86 76.00 [57.00, 101.00]	78.65 ±38.25 74.00 [54.00, 98.00]	0.061	79.55 ±37.96 75.00 [56.00, 99.00]	78.66 ±38.16 74.00 [54.85, 98.00]	0.023
HDL (mg/dl)*; mean (SD) median [IQR]	46.06 ±13.92 45.00 [37.00, 54.00]	44.62 ±13.66 43.00 [36.00, 52.00]	0.105	45.37 ±13.90 44.00 [36.10, 53.00]	44.70 ±13.71 43.00 [36.00, 52.00]	0.049
Total cholesterol (mg/dl)*; mean (SD) median [IQR]	157.26 ±47.98 152.00 [127.00, 182.00]	155.07 ±49.41 149.00 [124.00, 180.00]	0.045	155.52 ±48.29 150.00 [125.00, 181.00]	154.95 ±49.75 150.00 [124.00, 180.00]	0.012
Triglyceride (mg/dl)*; mean (SD) median [IQR]	168.74 ±127.17 141.00 [102.00, 199.00]	173.24 ±135.70 143.00 [103.00, 203.00]	0.034	173.05 ±139.11 142.00 [102.00, 205.00]	172.74 ±137.79 143.00 [102.00, 204.00]	0.002
**Burden of comorbidities**						
Combined comorbidity score; mean (SD)	2.75 ± 2.61	3.22 ± 2.86	0.175	2.95 ± 2.72	2.95 ± 2.67	0.001
Frailty Score; mean (SD)	0.18 ± 0.06	0.19 ± 0.07	0.170	0.18 ± 0.06	0.18 ± 0.06	0.000
**Baseline hospitalizations and hospital metrics**						

Number of Hospitalizations; mean (SD)	1.45 ± 11.61	3.17 ± 17.77	0.115	2.08 ± 13.95	2.15 ± 14.19	0.005
Any hospitalization within prior 91 days	1,002 (3.9%)	1,243 (6.4%)	0.113	631 (4.9%)	638 (5.0%)	0.003
Any hospitalization within prior 92-365 days	3,241 (12.8%)	3,260 (16.9%)	0.116	1,836 (14.3%)	1,844 (14.4%)	0.002
Number of hospitalizations (0, 1, 2 or more)						
< 1	21,556 (84.9%)	15,325 (79.4%)	0.144	10,625 (82.8%)	10,599 (82.6%)	0.005
1 - <2	2,265 (8.9%)	1,945 (10.1%)	0.039	1,192 (9.3%)	1,204 (9.4%)	0.003
>= 2	1,556 (6.1%)	2,024 (10.5%)	0.158	1,010 (7.9%)	1,024 (8.0%)	0.004
Heart failure hospitalization	1,203 (4.7%)	1,317 (6.8%)	0.089	713 (5.6%)	728 (5.7%)	0.005
ED visit	9,112 (35.9%)	8,151 (42.2%)	0.130	4,997 (39.0%)	5,019 (39.1%)	0.004
Calendar year of cohort entry						
2022	2,207 (8.7%)	6,672 (34.6%)	0.662	2,110 (16.4%)	2,081 (16.2%)	0.006
2023	12,235 (48.2%)	9,091 (47.1%)	0.022	7,288 (56.8%)	7,364 (57.4%)	0.012
2024	10,644 (41.9%)	3,495 (18.1%)	0.538	3,389 (26.4%)	3,346 (26.1%)	0.008
2025	291 (1.1%)	36 (0.2%)	0.118	40 (0.3%)	36 (0.3%)	0.006

Abbreviations: *not used in the propensity score; ACE, angiotensin-converting enzyme inhibitors; ARB, angiotensin receptor blocker; ARNI, angiotensin receptor/neprilysin inhibitor; BMI, body mass index; CABG, coronary artery bypass graft surgery; CED, cohort entry date; CKD, chronic kidney disease; COPD, chronic obstructive pulmonary disease; DKA, diabetic ketoacidosis; DPP4i, dipeptidyl peptidase-4 inhibitors; ED, emergency department; eGFR, estimated glomerular filtration rate (estimated using the quadratic GFR equation: GFR = EXP(1.911 + (5.249 / Serum creatinine) - (2.114 / (Serum creatinine^2)) - (0.00686 * Age) - 0.205 (if female))); HbA1c, hemoglobin A1c; HDL, high-density lipoprotein cholesterol; HONK, hyperglycemic hyperosmolar nonketotic state; IQR, inter-quartile-range; MASH, metabolic dysfunction associated steatohepatitis; MASLD, metabolic dysfunction associated steatotic liver disease; MI, myocardial infarction; N, number of participants; NSAIDs, non-steroidal anti-inflammatory drugs; PCSK9, proprotein convertase subtilisin/kexin type 9; PSA, prostate-specific antigen; PTCA, percutaneous transluminal coronary angioplasty; SGLT2i, sodium-glucose transport protein 2 inhibitors; SD, standard deviation; SMD, standardized mean difference; TIA, transient ischemic attack.

Missing data were handled by assuming absence of a code indicated absence of the condition for most binary covariates. Missing indicators were included for race and region in the propensity score model.

Laboratory values were only available in a subset of the Optum database and were truncated using clinically plausible cut-off values (BMI values >100 or <10 were set to missing; missingness before matching ~46%. Creatinine values >30 were set to missing, and values <0.8 were set to 0.8; missingness before matching ~38%. eGFR values >150 were set to 150, and values =0 were set to missing; missingness before matching ~50%. Glucose values <30 were set to missing; missingness before matching ~38%. HbA1c values ≥20 or ≤2 were set to missing; missingness before matching ~33%. HDL value missingness before matching was ~47%. Heart rate values <30 were set to missing; missingness before matching ~59%. LDL value missingness before matching was~44%. Systolic blood pressure values <30 were set to missing; missingness before matching ~43%. Total cholesterol value missingness before matching was ~46%. Triglyceride value missingness before matching was ~47%).

Race was reported by the respective database: Optum. The Race-Others category includes Asian and Hispanic individuals.

Supplementary Table 11. Baseline characteristics of initiators of tirzepatide vs dulaglutide when applying trial eligibility criteria before and after propensity score matching, Optum database. Values are number (percentage) unless otherwise specified.

	Before propensity score matching			After propensity score matching		
Variable	Tirzepatide (n = 20,263)	Dulaglutide (n = 13,970)	SMD	Tirzepatide (n = 9,530)	Dulaglutide (n = 9,530)	SMD
Demographics						
Age; mean (SD) median [IQR]	66.92 ±9.17 68.00 [61.00, 73.00]	69.14 ±9.05 70.00 [64.00, 75.00]	0.244	68.34 ±8.84 69.00 [63.00, 74.00]	68.35 ±9.07 69.00 [63.00, 75.00]	0.000
Gender						
Male	9,584 (47.3%)	6,873 (49.2%)	0.038	4,641 (48.7%)	4,640 (48.7%)	0.000
Female	10,679 (52,7%)	7,097 (50,8%)	0.038	4,889 (51,3%)	4,890 (51,3%)	0.000
Race						
White	10,227 (50.5%)	6,463 (46.3%)	0.084	4,596 (48.2%)	4,570 (48.0%)	0.005
Black	2,567 (12.7%)	2,182 (15.6%)	0.085	1,402 (14.7%)	1,373 (14.4%)	0.009
Unknown / Missing	7,229 (35.7%)	5,115 (36.6%)	0.020	3,411 (35.8%)	3,468 (36.4%)	0.012
Others	240 (1.2%)	210 (1.5%)	0.028	121 (1.3%)	119 (1.2%)	0.002
Region / State						
Northeast	1,665 (8.2%)	1,502 (10.8%)	0.087	909 (9.5%)	888 (9.3%)	0.008
Midwest / North central	3,645 (18.0%)	2,830 (20.3%)	0.058	1,890 (19.8%)	1,903 (20.0%)	0.003
South	12,397 (61.2%)	7,077 (50.7%)	0.213	5,192 (54.5%)	5,191 (54.5%)	0.000
West	2,547 (12.6%)	2,555 (18.3%)	0.159	1,535 (16.1%)	1,543 (16.2%)	0.002
Missing	9 (0.0%)	6 (0.0%)	0.001	4 (0.0%)	5 (0.1%)	0.004
Lifestyle risk factors						
Smoking / Tobacco use	5,928 (29.3%)	4,385 (31.4%)	0.046	2,908 (30.5%)	2,916 (30.6%)	0.002
Weight						
Overweight (25.0-29.9)	1,346 (6.6%)	1,719 (12.3%)	0.194	950 (10.0%)	934 (9.8%)	0.006
Class 1 Obesity (30.0-34.9)	4,061 (20.0%)	3,478 (24.9%)	0.117	2,249 (23.6%)	2,241 (23.5%)	0.002
Class 2 Obesity (35.0-39.9)	7,185 (35.5%)	4,497 (32.2%)	0.069	3,237 (34.0%)	3,239 (34.0%)	0.000
Class 3 Obesity (40.0 and above)	5,242 (25.9%)	2,603 (18.6%)	0.175	1,949 (20.5%)	1,978 (20.8%)	0.008
Unspecified Obesity	2,429 (12.0%)	1,673 (12.0%)	0.000	1,145 (12.0%)	1,138 (11.9%)	0.002
Diabetes complications						
Diabetic retinopathy	2,393 (11.8%)	2,355 (16.9%)	0.144	1,457 (15.3%)	1,440 (15.1%)	0.005
Diabetic neuropathy	7,322 (36.1%)	6,341 (45.4%)	0.189	4,061 (42.6%)	4,008 (42.1%)	0.011
Diabetic nephropathy	5,712 (28.2%)	5,201 (37.2%)	0.194	3,264 (34.2%)	3,249 (34.1%)	0.003
Diabetes with other opthalmic complications	1,027 (5.1%)	987 (7.1%)	0.084	596 (6.3%)	599 (6.3%)	0.001
Diabetes with peripheral circulatory disorders	4,517 (22.3%)	4,024 (28.8%)	0.150	2,519 (26.4%)	2,468 (25.9%)	0.012
Diabetic foot	960 (4.7%)	905 (6.5%)	0.076	561 (5.9%)	546 (5.7%)	0.007
Erectile dysfunction	1,149 (5.7%)	742 (5.3%)	0.016	546 (5.7%)	535 (5.6%)	0.005
Hypoglycemia	5,630 (27.8%)	4,402 (31.5%)	0.082	2,875 (30.2%)	2,858 (30.0%)	0.004
Hyperglycemia / DKA / HONK	10,893 (53.8%)	8,731 (62.5%)	0.178	5,652 (59.3%)	5,652 (59.3%)	0.000

Skin infections	2,648 (13.1%)	1,931 (13.8%)	0.022	1,290 (13.5%)	1,272 (13.3%)	0.006
Cardiovascular-related conditions						
Coronary atherosclerosis	8,807 (43.5%)	6,364 (45.6%)	0.042	4,324 (45.4%)	4,281 (44.9%)	0.009
Stable angina	2,452 (12.1%)	1,649 (11.8%)	0.009	1,135 (11.9%)	1,143 (12.0%)	0.003
Unstable angina	1,074 (5.3%)	734 (5.3%)	0.002	509 (5.3%)	497 (5.2%)	0.006
Hypertension	18,911 (93.3%)	13,207 (94.5%)	0.051	8,966 (94.1%)	8,971 (94.1%)	0.002
Hypotension	958 (4.7%)	890 (6.4%)	0.072	522 (5.5%)	539 (5.7%)	0.008
Hyperlipidemia	18,288 (90.3%)	12,648 (90.5%)	0.010	8,595 (90.2%)	8,616 (90.4%)	0.007
Acute MI	388 (1.9%)	340 (2.4%)	0.036	203 (2.1%)	209 (2.2%)	0.004
Old MI	1,938 (9.6%)	1,672 (12.0%)	0.078	1,033 (10.8%)	1,026 (10.8%)	0.002
Ischemic stroke	213 (1.1%)	262 (1.9%)	0.069	132 (1.4%)	140 (1.5%)	0.007
TIA	676 (3.3%)	585 (4.2%)	0.045	358 (3.8%)	351 (3.7%)	0.004
Cardiac conduction disorder	1,966 (9.7%)	1,524 (10.9%)	0.04	965 (10.1%)	969 (10.2%)	0.001
Previous cardiac procedure (CABG, PTCA, Stent)	667 (3.3%)	498 (3.6%)	0.015	336 (3.5%)	338 (3.5%)	0.001
PVD diagnosis or surgery	4,719 (23.3%)	3,769 (27.0%)	0.085	2,415 (25.3%)	2,381 (25.0%)	0.008
Atrial fibrillation	3,439 (17.0%)	2,416 (17.3%)	0.009	1,611 (16.9%)	1,646 (17.3%)	0.010
Other cardiac dysrhythmia	6,097 (30.1%)	4,213 (30.2%)	0.001	2,793 (29.3%)	2,803 (29.4%)	0.002
Heart failure	4,852 (23.9%)	3,768 (27.0%)	0.070	2,455 (25.8%)	2,440 (25.6%)	0.004
Acute heart failure	1,032 (5.1%)	926 (6.6%)	0.065	575 (6.0%)	572 (6.0%)	0.001
Cardiomyopathy	1,849 (9.1%)	1,509 (10.8%)	0.056	950 (10.0%)	974 (10.2%)	0.008
Valve disorders	3,780 (18.7%)	2,716 (19.4%)	0.020	1,803 (18.9%)	1,806 (19.0%)	0.001
Valve replacement	465 (2.3%)	344 (2.5%)	0.011	237 (2.5%)	232 (2.4%)	0.003
Edema	3,971 (19.6%)	2,873 (20.6%)	0.024	1,891 (19.8%)	1,872 (19.6%)	0.005
Venous thromboembolism / Pulmonary embolism	978 (4.8%)	721 (5.2%)	0.015	437 (4.6%)	448 (4.7%)	0.005
Pulmonary hypertension	971 (4.8%)	692 (5.0%)	0.008	437 (4.6%)	455 (4.8%)	0.009
Implantable cardioverter defibrillator	115 (0.6%)	89 (0.6%)	0.009	67 (0.7%)	62 (0.7%)	0.006
Hyperkalemia	845 (4.2%)	877 (6.3%)	0.095	465 (4.9%)	496 (5.2%)	0.015
Cerebrovascular procedure	40 (0.2%)	50 (0.4%)	0.031	26 (0.3%)	29 (0.3%)	0.006
Insertion of pacemakers / removal of cardiac lead	63 (0.3%)	45 (0.3%)	0.002	25 (0.3%)	28 (0.3%)	0.006
Renal-related conditions						
Hypertensive nephropathy	3,802 (18.8%)	3,494 (25.0%)	0.152	2,149 (22.5%)	2,156 (22.6%)	0.002
CKD Stage 1-2	1,466 (7.2%)	1,155 (8.3%)	0.039	772 (8.1%)	775 (8.1%)	0.001
CKD Stage 3-4	4,550 (22.5%)	4,124 (29.5%)	0.162	2,567 (26.9%)	2,564 (26.9%)	0.001
Unspecified CKD	1,831 (9.0%)	1,828 (13.1%)	0.129	1,093 (11.5%)	1,076 (11.3%)	0.006
Microalbuminuria or proteinuria	1,586 (7.8%)	1,309 (9.4%)	0.055	828 (8.7%)	863 (9.1%)	0.013
Acute kidney injury	1,747 (8.6%)	1,815 (13.0%)	0.141	1,042 (10.9%)	1,053 (11.0%)	0.004
Urinary tract infections	3,413 (16.8%)	2,597 (18.6%)	0.046	1,635 (17.2%)	1,626 (17.1%)	0.003
Genital infections	522 (2.6%)	479 (3.4%)	0.050	278 (2.9%)	289 (3.0%)	0.007
Urolithiasis (Kidney and urinary stone)	1,085 (5.4%)	703 (5.0%)	0.015	477 (5.0%)	476 (5.0%)	0.000

**Other comorbidities**						
COPD	3,648 (18.0%)	2,827 (20.2%)	0.057	1,861 (19.5%)	1,818 (19.1%)	0.011
Asthma	2,760 (13.6%)	1,646 (11.8%)	0.055	1,199 (12.6%)	1,155 (12.1%)	0.014
Obstructive sleep apnea	8,250 (40.7%)	4,591 (32.9%)	0.163	3,417 (35.9%)	3,369 (35.4%)	0.011
Serious bacterial infections	711 (3.5%)	737 (5.3%)	0.086	410 (4.3%)	411 (4.3%)	0.001
Pneumonia	1,152 (5.7%)	1,089 (7.8%)	0.084	626 (6.6%)	609 (6.4%)	0.007
Liver disease	3,932 (19.4%)	2,680 (19.2%)	0.006	1,823 (19.1%)	1,826 (19.2%)	0.001
MASH / MASLD	2,040 (10.1%)	1,113 (8.0%)	0.073	841 (8.8%)	832 (8.7%)	0.003
Fractures / Falls	1,358 (6.7%)	1,237 (8.9%)	0.080	767 (8.0%)	743 (7.8%)	0.009
Osteoporosis	1,117 (5.5%)	896 (6.4%)	0.038	553 (5.8%)	587 (6.2%)	0.015
Osteoarthritis	7,132 (35.2%)	4,825 (34.5%)	0.014	3,275 (34.4%)	3,279 (34.4%)	0.001
Depression	4,496 (22.2%)	3,470 (24.8%)	0.063	2,203 (23.1%)	2,185 (22.9%)	0.004
Dementia	976 (4.8%)	1,295 (9.3%)	0.175	643 (6.7%)	639 (6.7%)	0.002
Delirium or psychosis	303 (1.5%)	402 (2.9%)	0.095	204 (2.1%)	193 (2.0%)	0.008
Anxiety	4,782 (23.6%)	3,037 (21.7%)	0.044	2,069 (21.7%)	2,052 (21.5%)	0.004
Sleep disorders	8,113 (40.0%)	5,422 (38.8%)	0.025	3,690 (38.7%)	3,675 (38.6%)	0.003
Anemia	4,932 (24.3%)	3,828 (27.4%)	0.070	2,492 (26.1%)	2,476 (26.0%)	0.004
Influenza	381 (1.9%)	252 (1.8%)	0.006	185 (1.9%)	182 (1.9%)	0.002
COVID	2,345 (11.6%)	1,860 (13.3%)	0.053	1,176 (12.3%)	1,161 (12.2%)	0.005
Hyperthyroidism and other thyroid gland disorders	6,266 (30.9%)	4,034 (28.9%)	0.045	2,803 (29.4%)	2,822 (29.6%)	0.004
Hypothyroidism	4,979 (24.6%)	3,264 (23.4%)	0.028	2,250 (23.6%)	2,261 (23.7%)	0.003
Nephrotic syndrome	27 (0.1%)	27 (0.2%)	0.015	19 (0.2%)	18 (0.2%)	0.002
Urinary incontinence	1,486 (7.3%)	1,224 (8.8%)	0.053	773 (8.1%)	750 (7.9%)	0.009
Biliary disease	26 (0.1%)	27 (0.2%)	0.016	17 (0.2%)	15 (0.2%)	0.005
Pancreatitis	10 (0.0%)	10 (0.1%)	0.009	6 (0.1%)	5 (0.1%)	0.004
Bowel obstruction	39 (0.2%)	24 (0.2%)	0.005	17 (0.2%)	19 (0.2%)	0.005
Gastroparesis	217 (1.1%)	181 (1.3%)	0.021	123 (1.3%)	103 (1.1%)	0.019
**Diabetes medications**						
Number of antidiabetic drugs on CED, mean (SD)	2.14 ±0.99 2.00 [1.00, 3.00]	2.39 ±1.00 2.00 [2.00, 3.00]	0.248	2.32 ±1.03 2.00 [2.00, 3.00]	2.31 ±0.99 2.00 [2.00, 3.00]	0.009
Concomitant use or initiation of Metformin	8,597 (42.4%)	6,322 (45.3%)	0.057	4,294 (45.1%)	4,279 (44.9%)	0.003
Concomitant use or initiation of Insulins	4,855 (24.0%)	4,659 (33.4%)	0.209	2,894 (30.4%)	2,826 (29.7%)	0.016
Concomitant use or initiation of Sulfonylureas	3,076 (15.2%)	3,225 (23.1%)	0.202	1,954 (20.5%)	1,961 (20.6%)	0.002
Concomitant use or initiation of DPP-4i	1,256 (6.2%)	1,147 (8.2%)	0.078	677 (7.1%)	706 (7.4%)	0.012
Concomitant use or initiation of SGLT-2i	4,141 (20.4%)	3,167 (22.7%)	0.054	2,178 (22.9%)	2,151 (22.6%)	0.007
Concomitant use or initiation of Any other glucose-lowering drugs	1,205 (5.9%)	879 (6.3%)	0.014	605 (6.3%)	588 (6.2%)	0.007
Past use of Metformin	11,782 (58.1%)	8,549 (61.2%)	0.062	5,788 (60.7%)	5,779 (60.6%)	0.002
Past use of Insulins	6,333 (31.3%)	6,082 (43.5%)	0.256	3,785 (39.7%)	3,723 (39.1%)	0.013
Past use of Sulfonylureas	4,483 (22.1%)	4,475 (32.0%)	0.224	2,766 (29.0%)	2,762 (29.0%)	0.001
Past use of DPP-4i	1,961 (9.7%)	1,790 (12.8%)	0.099	1,064 (11.2%)	1,083 (11.4%)	0.006

Past use of SGLT-2i	5,757 (28.4%)	4,480 (32.1%)	0.080	3,067 (32.2%)	3,027 (31.8%)	0.009
Past use of Any other glucose-lowering drugs	1,757 (8.7%)	1,344 (9.6%)	0.033	908 (9.5%)	882 (9.3%)	0.009
Other medications						
ACE / ARB	14,817 (73.1%)	10,492 (75.1%)	0.045	7,085 (74.3%)	7,120 (74.7%)	0.008
ARNI	773 (3.8%)	474 (3.4%)	0.023	366 (3.8%)	370 (3.9%)	0.002
Thiazides	6,881 (34.0%)	4,386 (31.4%)	0.055	3,089 (32.4%)	3,066 (32.2%)	0.005
Beta-blockers	11,807 (58.3%)	8,625 (61.7%)	0.071	5,755 (60.4%)	5,806 (60.9%)	0.011
Calcium channel blockers	7,505 (37.0%)	5,569 (39.9%)	0.058	3,714 (39.0%)	3,690 (38.7%)	0.005
Digoxin / Digitoxin	182 (0.9%)	165 (1.2%)	0.028	94 (1.0%)	96 (1.0%)	0.002
Loop diuretics	5,742 (28.3%)	4,225 (30.2%)	0.042	2,810 (29.5%)	2,799 (29.4%)	0.003
Other diuretics	2,493 (12.3%)	1,631 (11.7%)	0.019	1,156 (12.1%)	1,159 (12.2%)	0.001
Intravenous diuretics	450 (2.2%)	343 (2.5%)	0.016	234 (2.5%)	229 (2.4%)	0.003
Nitrates	2,667 (13.2%)	2,066 (14.8%)	0.047	1,357 (14.2%)	1,339 (14.1%)	0.005
Anti-arrhythmics	850 (4.2%)	508 (3.6%)	0.029	350 (3.7%)	364 (3.8%)	0.008
Statins	16,727 (82.5%)	12,221 (87.5%)	0.138	8,205 (86.1%)	8,202 (86.1%)	0.001
PCSK9 inhibitors and other lipid-lowering drugs	3,905 (19.3%)	2,428 (17.4%)	0.049	1,772 (18.6%)	1,765 (18.5%)	0.002
Antiplatelet medications	4,204 (20.7%)	3,199 (22.9%)	0.052	2,138 (22.4%)	2,117 (22.2%)	0.005
Oral anticoagulants	3,374 (16.7%)	2,428 (17.4%)	0.019	1,607 (16.9%)	1,615 (16.9%)	0.002
COPD / Asthma medications	8,430 (41.6%)	5,367 (38.4%)	0.065	3,740 (39.2%)	3,753 (39.4%)	0.003
NSAIDS	6,317 (31.2%)	3,989 (28.6%)	0.057	2,792 (29.3%)	2,770 (29.1%)	0.005
Oral corticosteroids	5,850 (28.9%)	3,202 (22.9%)	0.136	2,388 (25.1%)	2,341 (24.6%)	0.011
Osteoporosis agents (incl. bisphosphonates)	663 (3.3%)	520 (3.7%)	0.025	345 (3.6%)	335 (3.5%)	0.006
Opioids	5,416 (26.7%)	3,569 (25.5%)	0.027	2,479 (26.0%)	2,464 (25.9%)	0.004
Anti-depressants	8,545 (42.2%)	5,785 (41.4%)	0.015	3,932 (41.3%)	3,929 (41.2%)	0.001
Antipsychotics	935 (4.6%)	800 (5.7%)	0.050	486 (5.1%)	474 (5.0%)	0.006
Anxiolytics / hypnotics, benzos	4,920 (24.3%)	2,785 (19.9%)	0.105	2,025 (21.2%)	1,973 (20.7%)	0.013
Dementia medications	410 (2.0%)	524 (3.8%)	0.103	264 (2.8%)	284 (3.0%)	0.013
Urinary tract infections antibiotics	10,578 (52.2%)	6,916 (49.5%)	0.054	4,767 (50.0%)	4,751 (49.9%)	0.003
Laxatives	808 (4.0%)	595 (4.3%)	0.014	399 (4.2%)	404 (4.2%)	0.003
Healthcare utilization marker						
Number of distinct medications; mean (SD) median [IQR]	16.34 ±7.03 15.00 [11.00, 20.00]	16.99 ±7.03 16.00 [12.00, 21.00]	0.092	16.83 ±6.96 16.00 [12.00, 21.00]	16.72 ±7.01 16.00 [12.00, 21.00]	0.015
Number of office visits; mean (SD) median [IQR]	11.86 ±7.62 10.00 [6.00, 16.00]	11.31 ±7.61 10.00 [6.00, 15.00]	0.072	11.60 ±7.29 10.00 [6.00, 15.00]	11.51 ±7.69 10.00 [6.00, 15.00]	0.012
Number of endocrinologist visits; mean (SD) median [IQR]	0.55 ±1.52 0.00 [0.00, 0.00]	0.63 ±1.74 0.00 [0.00, 0.00]	0.048	0.59 ±1.58 0.00 [0.00, 0.00]	0.59 ±1.64 0.00 [0.00, 0.00]	0.001
Number of cardiologist visits; mean (SD) median [IQR]	2.75 ±4.24 1.00 [0.00, 4.00]	2.70 ±4.57 1.00 [0.00, 4.00]	0.012	2.67 ±4.23 1.00 [0.00, 4.00]	2.67 ±4.42 1.00 [0.00, 4.00]	0.001
Number of internal / family medicine visits; mean (SD) median [IQR]	8.26 ±8.93 6.00 [3.00, 10.00]	10.10 ±11.87 6.00 [3.00, 12.00]	0.175	9.17 ±10.09 6.00 [3.00, 11.00]	9.14 ±10.34 6.00 [3.00, 11.00]	0.003
Number of electrocardiograms (ECG/EKG) ; mean (SD) median [IQR]	1.41 ±1.96 1.00 [0.00, 2.00]	1.48 ±2.25 1.00 [0.00, 2.00]	0.034	1.42 ±1.90 1.00 [0.00, 2.00]	1.42 ±1.99 1.00 [0.00, 2.00]	0.003
Number of echocardiograms; mean (SD) median [IQR]	0.47 ±0.78 0.00 [0.00, 1.00]	0.48 ±0.86 0.00 [0.00, 1.00]	0.009	0.47 ±0.76 0.00 [0.00, 1.00]	0.47 ±0.87 0.00 [0.00, 1.00]	0.001
Out-of-pocket medication cost; mean (SD) median [IQR]	703.26 ±992.89 422.78 [150.35, 971.64]	697.86 ±897.38 381.83 [126.64, 982.81]	0.006	710.96 ±799.71 429.76 [153.00, 1,008.05]	704.48 ±932.87 395.12 [131.00, 979.44]	0.007

Unique brand medicines; mean (SD) median [IQR]	16.62 ±7.26 16.00 [11.00, 21.00]	17.36 ±7.33 16.00 [12.00, 21.00]	0.101	17.16 ±7.22 16.00 [12.00, 21.00]	17.04 ±7.27 16.00 [12.00, 21.00]	0.017
Unique generic medicines; mean (SD) median [IQR]	16.34 ±7.03 15.00 [11.00, 20.00]	16.99 ±7.03 16.00 [12.00, 21.00]	0.092	16.83 ±6.96 16.00 [12.00, 21.00]	16.72 ±7.01 16.00 [12.00, 21.00]	0.015
Ratio of brand to generic medications; mean (SD) median [IQR]	1.02 ±0.03 1.00 [1.00, 1.00]	1.02 ±0.04 1.00 [1.00, 1.03]	0.119	1.02 ±0.04 1.00 [1.00, 1.00]	1.02 ±0.04 1.00 [1.00, 1.00]	0.019
**Healthy behavior markers**						
Colonoscopy / Sigmoidoscopy	2,364 (11.7%)	1,453 (10.4%)	0.040	1,065 (11.2%)	1,057 (11.1%)	0.003
Flu Pneumococcal vaccine	6,428 (31.7%)	5,059 (36.2%)	0.095	3,277 (34.4%)	3,360 (35.3%)	0.018
Pap smear	1,096 (5.4%)	505 (3.6%)	0.087	385 (4.0%)	396 (4.2%)	0.006
PSA test	5,402 (26.7%)	3,312 (23.7%)	0.068	2,361 (24.8%)	2,381 (25.0%)	0.005
Fecal occult blood test	877 (4.3%)	724 (5.2%)	0.040	464 (4.9%)	457 (4.8%)	0.003
Bone mineral density tests	1,800 (8.9%)	1,132 (8.1%)	0.028	800 (8.4%)	812 (8.5%)	0.005
Mammograms	5,293 (26.1%)	3,012 (21.6%)	0.107	2,211 (23.2%)	2,202 (23.1%)	0.002
Telemedicine	4,875 (24.1%)	3,299 (23.6%)	0.010	2,195 (23.0%)	2,174 (22.8%)	0.005
**Laboratory and diagnostic tests**						
HbA1c tests; mean (SD) median [IQR]	2.47 ±1.40 2.00 [2.00, 3.00]	2.63 ±1.40 3.00 [2.00, 3.00]	0.111	2.59 ±1.46 3.00 [2.00, 3.00]	2.58 ±1.37 3.00 [2.00, 3.00]	0.008
Lipid panels; mean (SD) median [IQR]	1.81 ±1.27 2.00 [1.00, 3.00]	1.71 ±1.25 2.00 [1.00, 2.00]	0.076	1.76 ±1.24 2.00 [1.00, 2.00]	1.76 ±1.25 2.00 [1.00, 2.00]	0.003
Creatinine tests; mean (SD) median [IQR]	3.55 ±3.47 3.00 [2.00, 5.00]	3.93 ±4.17 3.00 [2.00, 5.00]	0.100	3.74 ±3.92 3.00 [2.00, 5.00]	3.74 ±3.33 3.00 [2.00, 5.00]	0.002
Natriuretic peptide tests; mean (SD) median [IQR]	0.27 ±0.83 0.00 [0.00, 0.00]	0.29 ±0.86 0.00 [0.00, 0.00]	0.020	0.27 ±0.77 0.00 [0.00, 0.00]	0.28 ±0.84 0.00 [0.00, 0.00]	0.017
Urine tests; mean (SD) median [IQR]	1.30 ±1.84 1.00 [0.00, 2.00]	1.31 ±1.89 1.00 [0.00, 2.00]	0.008	1.29 ±1.83 1.00 [0.00, 2.00]	1.29 ±1.90 1.00 [0.00, 2.00]	0.002
**Lab values**						
HbA1c (%)*; mean (SD) median [IQR]	7.61 ±1.66 7.20 [6.40, 8.40]	8.21 ±1.78 7.90 [7.00, 9.10]	0.351	7.87 ±1.72 7.50 [6.60, 8.80]	8.08 ±1.75 7.80 [6.80, 9.00]	0.124
Glucose (mg/dl)*; mean (SD) median [IQR]	154.58 ±66.18 137.00 [110.00, 179.50]	1,380.56 ±109,788.81 157.00 [123.00, 208.00]	0.016	162.30 ±69.35 145.75 [114.62, 191.00]	1,896.01 ±131,292.86 154.50 [120.00, 205.00]	0.019
Creatinine (mg/dl)*; mean (SD) median [IQR]	1.07 ±0.40 0.96 [0.80, 1.19]	1.15 ±1.42 1.00 [0.81, 1.27]	0.072	1.11 ±0.39 0.99 [0.81, 1.23]	1.12 ±1.07 0.98 [0.80, 1.24]	0.016
Systolic blood pressure (mmHg)*; mean (SD) median [IQR]	129.55 ±22.93 130.00 [120.00, 139.00]	130.42 ±28.37 130.00 [120.00, 140.00]	0.034	129.98 ±26.70 130.00 [120.00, 139.92]	130.00 ±28.46 130.00 [120.00, 139.50]	0.001
Heart rate (1/min)*; mean (SD) median [IQR]	76.70 ±16.10 76.00 [67.00, 85.00]	77.34 ±20.43 76.00 [68.00, 86.00]	0.035	76.69 ±19.05 76.00 [67.00, 84.00]	77.27 ±22.50 76.00 [68.00, 85.00]	0.028
BMI (kg/m2)*; mean (SD) median [IQR]	37.91 ±7.65 36.80 [32.42, 41.84]	35.52 ±7.12 34.30 [30.40, 39.10]	0.324	36.89 ±7.51 35.74 [31.50, 40.70]	35.87 ±7.07 34.70 [30.90, 39.56]	0.140
eGFR (ml/min/1.73m2)*; mean (SD) median [IQR]	980.17 ±95,276.29 74.00 [56.00, 91.00]	1,677.75 ±126,856.10 69.00 [50.00, 88.00]	0.006	69.69 ±22.85 71.00 [52.50, 89.00]	2,210.54 ±146,316.06 71.00 [52.10, 89.00]	0.021
LDL (mg/dl)*; mean (SD) median [IQR]	80.96 ±37.86 76.00 [57.00, 101.00]	78.65 ±38.25 74.00 [54.00, 98.00]	0.061	79.55 ±37.96 75.00 [56.00, 99.00]	78.66 ±38.16 74.00 [54.85, 98.00]	0.023
HDL (mg/dl)*; mean (SD) median [IQR]	46.06 ±13.92 45.00 [37.00, 54.00]	44.62 ±13.66 43.00 [36.00, 52.00]	0.105	45.37 ±13.90 44.00 [36.10, 53.00]	44.70 ±13.71 43.00 [36.00, 52.00]	0.049
Total cholesterol (mg/dl)*; mean (SD) median [IQR]	157.26 ±47.98 152.00 [127.00, 182.00]	155.07 ±49.41 149.00 [124.00, 180.00]	0.045	155.52 ±48.29 150.00 [125.00, 181.00]	154.95 ±49.75 150.00 [124.00, 180.00]	0.012
Triglyceride (mg/dl)*; mean (SD) median [IQR]	168.74 ±127.17 141.00 [102.00, 199.00]	173.24 ±135.70 143.00 [103.00, 203.00]	0.034	173.05 ±139.11 142.00 [102.00, 205.00]	172.74 ±137.79 143.00 [102.00, 204.00]	0.002
**Burden of comorbidities**						
Combined comorbidity score; mean (SD) median [IQR]	2.99 ±2.68 2.00 [1.00, 5.00]	3.56 ±2.91 3.00 [1.00, 5.00]	0.206	3.31 ±2.82 3.00 [1.00, 5.00]	3.30 ±2.74 3.00 [1.00, 5.00]	0.007

Frailty Score; mean (SD) median [IQR]	0.19 ±0.06 0.18 [0.15, 0.22]	0.20 ±0.07 0.19 [0.15, 0.23]	0.179	0.19 ±0.06 0.18 [0.15, 0.22]	0.19 ±0.06 0.18 [0.15, 0.22]	0.006
**Baseline hospitalizations and hospital metrics**						
Number of Hospitalizations; mean (SD) median [IQR]	0.25 ±0.70 0.00 [0.00, 0.00]	0.38 ±0.97 0.00 [0.00, 0.00]	0.159	0.31 ±0.81 0.00 [0.00, 0.00]	0.30 ±0.82 0.00 [0.00, 0.00]	0.002
Any hospitalization within prior 91 days	855 (4.2%)	986 (7.1%)	0.123	524 (5.5%)	516 (5.4%)	0.004
Any hospitalization within prior 92-365 days	2,773 (13.7%)	2,508 (18.0%)	0.117	1,493 (15.7%)	1,500 (15.7%)	0.002
Number of hospitalizations (0, 1, 2 or more)						
< 1	17,004 (83.9%)	10,923 (78.2%)	0.147	7,743 (81.2%)	7,732 (81.1%)	0.003
1 - <2	2,235 (11.0%)	1,900 (13.6%)	0.078	1,170 (12.3%)	1,187 (12.5%)	0.005
>= 2	1,024 (5.1%)	1,147 (8.2%)	0.127	617 (6.5%)	611 (6.4%)	0.003
Heart failure hospitalization	1,059 (5.2%)	1,013 (7.3%)	0.084	607 (6.4%)	610 (6.4%)	0.001
ED visit	7,218 (35.6%)	5,921 (42.4%)	0.139	3,748 (39.3%)	3,752 (39.4%)	0.001
**Calendar year of cohort entry**						
2022	1,352 (6.7%)	4,345 (31.1%)	0.657	1,298 (13.6%)	1,293 (13.6%)	0.002
2023	7,976 (39.4%)	6,094 (43.6%)	0.087	4,803 (50.4%)	4,855 (50.9%)	0.011
2024	10,644 (52.5%)	3,495 (25.0%)	0.589	3,389 (35.6%)	3,346 (35.1%)	0.009
2025	291 (1.4%)	36 (0.3%)	0.129	40 (0.4%)	36 (0.4%)	0.007

Abbreviations: *not used in the propensity score; ACE, angiotensin-converting enzyme inhibitors; ARB, angiotensin receptor blocker; ARNI, angiotensin receptor/neprilysin inhibitor; BMI, body mass index; CABG, coronary artery bypass graft surgery; CED, cohort entry date; CKD, chronic kidney disease; COPD, chronic obstructive pulmonary disease; DKA, diabetic ketoacidosis; DPP4i, dipeptidyl peptidase-4 inhibitors; ED, emergency department; eGFR, estimated glomerular filtration rate (estimated using the quadratic GFR equation: GFR = EXP(1.911 + (5.249 / Serum creatinine) - (2.114 / (Serum creatinine^2)) - (0.00686 * Age) - 0.205 (if female))); HbA1c, hemoglobin A1c; HDL, high-density lipoprotein cholesterol; HONK, hyperglycemic hyperosmolar nonketotic state; IQR, inter-quartile-range; MASH, metabolic dysfunction associated steatohepatitis; MASLD, metabolic dysfunction associated steatotic liver disease; MI, myocardial infarction; N, number of participants; NSAIDs, non-steroidal anti-inflammatory drugs; PCSK9, proprotein convertase subtilisin/kexin type 9; PSA, prostate-specific antigen; PTCA, percutaneous transluminal coronary angioplasty; SGLT2i, sodium-glucose transport protein 2 inhibitors; SD, standard deviation; SMD, standardized mean difference; TIA, transient ischemic attack.
Missing data were handled by assuming absence of a code indicated absence of the condition for most binary covariates. Missing indicators were included for race and region in the propensity score model.
Laboratory values were only available in a subset of the Optum database and were truncated using clinically plausible cut-off values (BMI values >100 or <10 were set to missing; missingness before matching ~46%. Creatinine values >30 were set to missing, and values <0.8 were set to 0.8; missingness before matching ~38%. eGFR values >150 were set to 150, and values =0 were set to missing; missingness before matching ~50%. Glucose values <30 were set to missing; missingness before matching ~38%. HbA1c values ≥20 or ≤2 were set to missing; missingness before matching ~33%. HDL value missingness before matching was ~47%. Heart rate values <30 were set to missing; missingness before matching ~59%. LDL value missingness before matching was~44%. Systolic blood pressure values <30 were set to missing; missingness before matching ~43%. Total cholesterol value missingness before matching was ~46%. Triglyceride value missingness before matching was ~47%).
The Race-Others category includes Asian and Hispanic individuals.

Supplementary Table 12. Baseline characteristics of initiators of tirzepatide vs dulaglutide when applying trial eligibility criteria before and after propensity score matching. Marketscan database. Values are number (percentage) unless otherwise specified.

	Before propensity score matching			After propensity score matching		
Variable	Tirzepatide (n = 5,114)	Dulaglutide (n = 5,324)	SMD	Tirzepatide (n = 3,297)	Dulaglutide (n = 3,297)	SMD
**Demographics**						
Age; mean (SD) median [IQR]	59.86 ±9.36 59.00 [53.00, 65.00]	63.74 ±10.60 63.00 [56.00, 72.00]	0.388	61.28 ±9.70 61.00 [55.00, 68.00]	61.27 ±9.90 61.00 [54.00, 68.00]	0.002
Gender						
Male	2,537 (49.6%)	2,882 (54.1%)	0.091	1,712 (51.9%)	1,720 (52.2%)	0.005
Female	2,577 (50,4%)	2,442 (45,9%)	0.091	1,585 (48,1%)	1,577 (47,8%)	0.005
Region / State						
Northeast	538 (10.5%)	635 (11.9%)	0.045	374 (11.3%)	367 (11.1%)	0.007
Midwest / North central	1,352 (26.4%)	2,215 (41.6%)	0.324	1,099 (33.3%)	1,100 (33.4%)	0.001
South	2,974 (58.2%)	2,179 (40.9%)	0.350	1,649 (50.0%)	1,650 (50.0%)	0.001
West	244 (4.8%)	289 (5.4%)	0.030	171 (5.2%)	176 (5.3%)	0.007
Missing	6 (0.1%)	6 (0.1%)	0.001	4 (0.1%)	4 (0.1%)	0.000
**Lifestyle risk factors**						
Smoking / Tobacco use	697 (13.6%)	808 (15.2%)	0.044	481 (14.6%)	466 (14.1%)	0.013
Weight						
Overweight (25.0-29.9)	440 (8.6%)	813 (15.3%)	0.207	347 (10.5%)	352 (10.7%)	0.005
Class 1 Obesity (30.0-34.9)	425 (8.3%)	569 (10.7%)	0.081	302 (9.2%)	312 (9.5%)	0.010
Class 2 Obesity (35.0-39.9)	342 (6.7%)	326 (6.1%)	0.023	220 (6.7%)	223 (6.8%)	0.004
Class 3 Obesity (40.0 and above)	990 (19.4%)	1,205 (22.6%)	0.080	689 (20.9%)	691 (21.0%)	0.001
Unspecified Obesity	2,917 (57.0%)	2,411 (45.3%)	0.237	1,739 (52.7%)	1,719 (52.1%)	0.012
**Diabetes complications**						
Diabetic retinopathy	494 (9.7%)	706 (13.3%)	0.113	361 (10.9%)	360 (10.9%)	0.001
Diabetic neuropathy	1,283 (25.1%)	1,830 (34.4%)	0.204	953 (28.9%)	966 (29.3%)	0.009
Diabetic nephropathy	837 (16.4%)	1,327 (24.9%)	0.213	621 (18.8%)	620 (18.8%)	0.001
Diabetes with other opthalmic complications	114 (2.2%)	152 (2.9%)	0.040	88 (2.7%)	77 (2.3%)	0.021
Diabetes with peripheral circulatory disorders	702 (13.7%)	975 (18.3%)	0.125	498 (15.1%)	515 (15.6%)	0.014
Diabetic foot	151 (3.0%)	223 (4.2%)	0.067	116 (3.5%)	120 (3.6%)	0.007
Erectile dysfunction	247 (4.8%)	246 (4.6%)	0.010	169 (5.1%)	158 (4.8%)	0.015
Hypoglycemia	1,038 (20.3%)	1,231 (23.1%)	0.069	698 (21.2%)	722 (21.9%)	0.018
Hyperglycemia / DKA / HONK	2,649 (51.8%)	3,122 (58.6%)	0.138	1,811 (54.9%)	1,816 (55.1%)	0.003
Skin infections	585 (11.4%)	680 (12.8%)	0.041	380 (11.5%)	388 (11.8%)	0.008
**Cardiovascular-related conditions**						
Coronary atherosclerosis	1,695 (33.1%)	2,104 (39.5%)	0.133	1,174 (35.6%)	1,182 (35.9%)	0.005
Stable angina	400 (7.8%)	507 (9.5%)	0.060	288 (8.7%)	274 (8.3%)	0.015

Unstable angina	200 (3.9%)	270 (5.1%)	0.056	145 (4.4%)	144 (4.4%)	0.001
Hypertension	4,556 (89.1%)	4,842 (90.9%)	0.062	2,955 (89.6%)	2,960 (89.8%)	0.005
Hypotension	104 (2.0%)	211 (4.0%)	0.113	84 (2.5%)	79 (2.4%)	0.010
Hyperlipidemia	4,451 (87.0%)	4,642 (87.2%)	0.005	2,882 (87.4%)	2,859 (86.7%)	0.021
Acute MI	109 (2.1%)	164 (3.1%)	0.06	82 (2.5%)	80 (2.4%)	0.004
Old MI	236 (4.6%)	273 (5.1%)	0.024	159 (4.8%)	165 (5.0%)	0.008
Ischemic stroke	78 (1.5%)	144 (2.7%)	0.082	60 (1.8%)	64 (1.9%)	0.009
TIA	148 (2.9%)	176 (3.3%)	0.024	91 (2.8%)	96 (2.9%)	0.009
Cardiac conduction disorder	266 (5.2%)	432 (8.1%)	0.117	197 (6.0%)	191 (5.8%)	0.008
Previous cardiac procedure (CABG, PTCA, Stent)	155 (3.0%)	220 (4.1%)	0.059	117 (3.5%)	115 (3.5%)	0.003
PVD diagnosis or surgery	733 (14.3%)	928 (17.4%)	0.085	519 (15.7%)	507 (15.4%)	0.010
Atrial fibrillation	556 (10.9%)	755 (14.2%)	0.100	390 (11.8%)	391 (11.9%)	0.001
Other cardiac dysrhythmia	1,202 (23.5%)	1,367 (25.7%)	0.050	775 (23.5%)	773 (23.4%)	0.001
Heart failure	618 (12.1%)	944 (17.7%)	0.159	452 (13.7%)	461 (14.0%)	0.008
Acute heart failure	136 (2.7%)	228 (4.3%)	0.089	92 (2.8%)	99 (3.0%)	0.013
Cardiomyopathy	339 (6.6%)	452 (8.5%)	0.070	245 (7.4%)	243 (7.4%)	0.002
Valve disorders	622 (12.2%)	796 (15.0%)	0.082	430 (13.0%)	432 (13.1%)	0.002
Valve replacement	50 (1.0%)	88 (1.7%)	0.059	41 (1.2%)	40 (1.2%)	0.003
Edema	597 (11.7%)	708 (13.3%)	0.049	396 (12.0%)	390 (11.8%)	0.006
Venous thromboembolism / Pulmonary embolism	172 (3.4%)	229 (4.3%)	0.049	129 (3.9%)	121 (3.7%)	0.013
Pulmonary hypertension	104 (2.0%)	130 (2.4%)	0.028	60 (1.8%)	66 (2.0%)	0.013
Implantable cardioverter defibrillator	20 (0.4%)	37 (0.7%)	0.041	15 (0.5%)	17 (0.5%)	0.009
Hyperkalemia	82 (1.6%)	180 (3.4%)	0.114	63 (1.9%)	66 (2.0%)	0.007
Cerebrovascular procedure	7 (0.1%)	11 (0.2%)	0.017	5 (0.2%)	5 (0.2%)	0.000
Insertion of pacemakers / removal of cardiac lead	2 (0.0%)	15 (0.3%)	0.061	2 (0.1%)	4 (0.1%)	0.02
Renal-related conditions						
Hypertensive nephropathy	421 (8.2%)	743 (14.0%)	0.183	321 (9.7%)	338 (10.3%)	0.017
CKD Stage 1-2	151 (3.0%)	188 (3.5%)	0.033	112 (3.4%)	109 (3.3%)	0.005
CKD Stage 3-4	480 (9.4%)	879 (16.5%)	0.213	381 (11.6%)	378 (11.5%)	0.003
Unspecified CKD	160 (3.1%)	303 (5.7%)	0.125	127 (3.9%)	130 (3.9%)	0.005
Microalbuminuria or proteinuria	207 (4.0%)	267 (5.0%)	0.047	143 (4.3%)	130 (3.9%)	0.02
Acute kidney injury	207 (4.0%)	433 (8.1%)	0.171	164 (5.0%)	168 (5.1%)	0.006
Urinary tract infections	626 (12.2%)	656 (12.3%)	0.002	374 (11.3%)	389 (11.8%)	0.014
Genital infections	161 (3.1%)	175 (3.3%)	0.008	117 (3.5%)	114 (3.5%)	0.005
Urolithiasis (Kidney and urinary stone)	223 (4.4%)	237 (4.5%)	0.004	138 (4.2%)	138 (4.2%)	0.000
Other comorbidities						
COPD	448 (8.8%)	583 (11.0%)	0.074	310 (9.4%)	311 (9.4%)	0.001
Asthma	541 (10.6%)	494 (9.3%)	0.043	312 (9.5%)	330 (10.0%)	0.018
Obstructive sleep apnea	1,974 (38.6%)	1,820 (34.2%)	0.092	1,176 (35.7%)	1,204 (36.5%)	0.018

Serious bacterial infections	167 (3.3%)	290 (5.4%)	0.107	115 (3.5%)	134 (4.1%)	0.030
Pneumonia	202 (3.9%)	294 (5.5%)	0.074	133 (4.0%)	150 (4.5%)	0.025
Liver disease	799 (15.6%)	763 (14.3%)	0.036	469 (14.2%)	445 (13.5%)	0.021
MASH / MASLD	438 (8.6%)	367 (6.9%)	0.063	246 (7.5%)	238 (7.2%)	0.009
Fractures / Falls	201 (3.9%)	300 (5.6%)	0.080	150 (4.5%)	148 (4.5%)	0.003
Osteoporosis	95 (1.9%)	138 (2.6%)	0.050	70 (2.1%)	78 (2.4%)	0.016
Osteoarthritis	1,268 (24.8%)	1,372 (25.8%)	0.022	824 (25.0%)	841 (25.5%)	0.012
Depression	803 (15.7%)	880 (16.5%)	0.022	520 (15.8%)	515 (15.6%)	0.004
Dementia	97 (1.9%)	251 (4.7%)	0.158	82 (2.5%)	75 (2.3%)	0.014
Delirium or psychosis	25 (0.5%)	49 (0.9%)	0.052	20 (0.6%)	23 (0.7%)	0.011
Anxiety	1,053 (20.6%)	937 (17.6%)	0.076	624 (18.9%)	613 (18.6%)	0.009
Sleep disorders	1,502 (29.4%)	1,410 (26.5%)	0.064	917 (27.8%)	901 (27.3%)	0.011
Anemia	1,029 (20.1%)	1,166 (21.9%)	0.044	656 (19.9%)	652 (19.8%)	0.003
Influenza	136 (2.7%)	83 (1.6%)	0.077	57 (1.7%)	63 (1.9%)	0.014
COVID	748 (14.6%)	790 (14.8%)	0.006	470 (14.3%)	487 (14.8%)	0.015
Hyperthyroidism and other thyroid gland disorders	1,400 (27.4%)	1,328 (24.9%)	0.055	860 (26.1%)	839 (25.4%)	0.015
Hypothyroidism	1,067 (20.9%)	1,014 (19.0%)	0.046	675 (20.5%)	639 (19.4%)	0.027
Nephrotic syndrome	1 (0.0%)	7 (0.1%)	0.041	1 (0.0%)	2 (0.1%)	0.014
Urinary incontinence	191 (3.7%)	257 (4.8%)	0.054	121 (3.7%)	134 (4.1%)	0.020
Biliary disease	11 (0.2%)	30 (0.6%)	0.056	7 (0.2%)	7 (0.2%)	0.000
Pancreatitis	4 (0.1%)	2 (0.0%)	0.017	1 (0.0%)	2 (0.1%)	0.014
Bowel obstruction	10 (0.2%)	22 (0.4%)	0.040	10 (0.3%)	10 (0.3%)	0.000
Gastroparesis	48 (0.9%)	38 (0.7%)	0.025	27 (0.8%)	24 (0.7%)	0.010
**Diabetes medications**						
Number of antidiabetic drugs on CED, mean (SD)	2.18 ±1.01 2.00 [1.00, 3.00]	2.32 ±1.05 2.00 [2.00, 3.00]	0.142	2.27 ±1.03 2.00 [2.00, 3.00]	2.27 ±1.03 2.00 [2.00, 3.00]	0.001
Concomitant use or initiation of Metformin	2,402 (47.0%)	2,418 (45.4%)	0.031	1,570 (47.6%)	1,564 (47.4%)	0.004
Concomitant use or initiation of Insulins	1,089 (21.3%)	1,554 (29.2%)	0.182	827 (25.1%)	833 (25.3%)	0.004
Concomitant use or initiation of Sulfonylureas	592 (11.6%)	984 (18.5%)	0.194	473 (14.3%)	481 (14.6%)	0.007
Concomitant use or initiation of DPP-4i	417 (8.2%)	481 (9.0%)	0.031	293 (8.9%)	290 (8.8%)	0.003
Concomitant use or initiation of SGLT-2i	1,296 (25.3%)	1,320 (24.8%)	0.013	882 (26.8%)	871 (26.4%)	0.008
Concomitant use or initiation of Any other glucose-lowering drugs	217 (4.2%)	285 (5.4%)	0.052	154 (4.7%)	156 (4.7%)	0.003
Past use of Metformin	3,263 (63.8%)	3,437 (64.6%)	0.016	2,144 (65.0%)	2,132 (64.7%)	0.008
Past use of Insulins	1,421 (27.8%)	2,139 (40.2%)	0.264	1,094 (33.2%)	1,100 (33.4%)	0.004
Past use of Sulfonylureas	889 (17.4%)	1,414 (26.6%)	0.223	699 (21.2%)	706 (21.4%)	0.005
Past use of DPP-4i	661 (12.9%)	775 (14.6%)	0.047	459 (13.9%)	463 (14.0%)	0.003
Past use of SGLT-2i	1,674 (32.7%)	1,794 (33.7%)	0.020	1,150 (34.9%)	1,139 (34.5%)	0.007
Past use of Any other glucose-lowering drugs	303 (5.9%)	414 (7.8%)	0.073	218 (6.6%)	212 (6.4%)	0.007
**Other medications**						
ACE / ARB	3,642 (71.2%)	3,923 (73.7%)	0.055	2,363 (71.7%)	2,392 (72.6%)	0.020

ARNI	168 (3.3%)	188 (3.5%)	0.014	119 (3.6%)	110 (3.3%)	0.015
Thiazides	1,753 (34.3%)	1,700 (31.9%)	0.05	1,089 (33.0%)	1,076 (32.6%)	0.008
Beta-blockers	2,743 (53.6%)	3,187 (59.9%)	0.126	1,866 (56.6%)	1,847 (56.0%)	0.012
Calcium channel blockers	1,641 (32.1%)	1,921 (36.1%)	0.084	1,120 (34.0%)	1,112 (33.7%)	0.005
Digoxin / Digitoxin	36 (0.7%)	54 (1.0%)	0.034	24 (0.7%)	20 (0.6%)	0.015
Loop diuretics	940 (18.4%)	1,226 (23.0%)	0.115	656 (19.9%)	637 (19.3%)	0.015
Other diuretics	541 (10.6%)	564 (10.6%)	0.000	340 (10.3%)	352 (10.7%)	0.012
Intravenous diuretics	52 (1.0%)	78 (1.5%)	0.04	34 (1.0%)	33 (1.0%)	0.003
Nitrates	557 (10.9%)	764 (14.4%)	0.104	411 (12.5%)	414 (12.6%)	0.003
Anti-arrhythmics	171 (3.3%)	184 (3.5%)	0.006	102 (3.1%)	108 (3.3%)	0.010
Statins	3,994 (78.1%)	4,455 (83.7%)	0.142	2,665 (80.8%)	2,666 (80.9%)	0.001
PCSK9 inhibitors and other lipid-lowering drugs	923 (18.0%)	995 (18.7%)	0.017	608 (18.4%)	606 (18.4%)	0.002
Antiplatelet medications	1,052 (20.6%)	1,330 (25.0%)	0.105	740 (22.4%)	765 (23.2%)	0.018
Oral anticoagulants	664 (13.0%)	870 (16.3%)	0.095	470 (14.3%)	456 (13.8%)	0.012
COPD / Asthma medications	2,006 (39.2%)	1,878 (35.3%)	0.082	1,218 (36.9%)	1,220 (37.0%)	0.001
NSAIDS	1,762 (34.5%)	1,506 (28.3%)	0.133	1,040 (31.5%)	1,052 (31.9%)	0.008
Oral corticosteroids	1,571 (30.7%)	1,359 (25.5%)	0.116	894 (27.1%)	932 (28.3%)	0.026
Osteoporosis agents (incl. bisphosphonates)	86 (1.7%)	110 (2.1%)	0.028	67 (2.0%)	66 (2.0%)	0.002
Opioids	1,135 (22.2%)	1,147 (21.5%)	0.016	688 (20.9%)	722 (21.9%)	0.025
Anti-depressants	1,981 (38.7%)	1,928 (36.2%)	0.052	1,237 (37.5%)	1,218 (36.9%)	0.012
Antipsychotics	167 (3.3%)	170 (3.2%)	0.004	101 (3.1%)	107 (3.2%)	0.010
Anxiolytics / hypnotics, benzos	1,197 (23.4%)	973 (18.3%)	0.127	684 (20.7%)	668 (20.3%)	0.012
Dementia medications	36 (0.7%)	118 (2.2%)	0.126	32 (1.0%)	31 (0.9%)	0.003
Urinary tract infections antibiotics	2,571 (50.3%)	2,479 (46.6%)	0.074	1,543 (46.8%)	1,565 (47.5%)	0.013
Laxatives	186 (3.6%)	161 (3.0%)	0.034	101 (3.1%)	104 (3.2%)	0.005
Healthcare utilization marker						
Number of distinct medications; mean (SD) median [IQR]	15.49 ±6.74 15.00 [11.00, 19.00]	15.36 ±6.48 15.00 [11.00, 19.00]	0.021	15.25 ±6.43 14.00 [11.00, 19.00]	15.30 ±6.66 15.00 [11.00, 19.00]	0.007
Number of office visits; mean (SD) median [IQR]	11.00 ±7.13 9.00 [6.00, 14.00]	10.94 ±7.17 9.00 [6.00, 14.00]	0.008	10.69 ±6.86 9.00 [6.00, 14.00]	10.79 ±7.26 9.00 [6.00, 14.00]	0.014
Number of endocrinologist visits; mean (SD) median [IQR]	0.52 ±1.41 0.00 [0.00, 0.00]	0.64 ±1.70 0.00 [0.00, 0.00]	0.076	0.56 ±1.50 0.00 [0.00, 0.00]	0.56 ±1.53 0.00 [0.00, 0.00]	0.003
Number of cardiologist visits; mean (SD) median [IQR]	2.08 ±3.48 1.00 [0.00, 3.00]	2.43 ±4.02 1.00 [0.00, 3.00]	0.092	2.12 ±3.51 1.00 [0.00, 3.00]	2.14 ±3.70 1.00 [0.00, 3.00]	0.004
Number of internal / family medicine visits; mean (SD) median [IQR]	5.56 ±5.91 4.00 [2.00, 7.00]	6.33 ±7.78 4.00 [2.00, 8.00]	0.113	5.61 ±5.90 4.00 [2.00, 7.00]	5.59 ±7.05 4.00 [2.00, 7.00]	0.004
Number of electrocardiograms (ECG/EKG) ; mean (SD) median [IQR]	1.24 ±1.73 1.00 [0.00, 2.00]	1.43 ±2.01 1.00 [0.00, 2.00]	0.105	1.28 ±1.79 1.00 [0.00, 2.00]	1.30 ±1.85 1.00 [0.00, 2.00]	0.011
Number of echocardiograms; mean (SD) median [IQR]	0.43 ±0.81 0.00 [0.00, 1.00]	0.51 ±0.92 0.00 [0.00, 1.00]	0.087	0.44 ±0.82 0.00 [0.00, 1.00]	0.45 ±0.84 0.00 [0.00, 1.00]	0.015
Out-of-pocket medication cost; mean (SD) median [IQR]	644.54 ±1,124.49 379.12 [150.00, 805.88]	606.97 ±1,022.30 379.82 [175.86, 765.84]	0.035	628.06 ±920.97 371.38 [154.90, 811.08]	634.32 ±1,049.85 385.12 [167.24, 780.43]	0.006
Unique brand medicines; mean (SD) median [IQR]	15.77 ±6.94 15.00 [11.00, 20.00]	15.65 ±6.70 15.00 [11.00, 19.00]	0.018	15.53 ±6.60 15.00 [11.00, 19.00]	15.58 ±6.88 15.00 [11.00, 19.00]	0.008
Unique generic medicines; mean (SD) median [IQR]	15.49 ±6.74 15.00 [11.00, 19.00]	15.36 ±6.48 15.00 [11.00, 19.00]	0.021	15.25 ±6.43 14.00 [11.00, 19.00]	15.30 ±6.66 15.00 [11.00, 19.00]	0.007
Ratio of brand to generic medications; mean (SD) median [IQR]	1.02 ±0.04 1.00 [1.00, 1.00]	1.02 ±0.04 1.00 [1.00, 1.00]	0.013	1.02 ±0.04 1.00 [1.00, 1.00]	1.02 ±0.04 1.00 [1.00, 1.00]	0.004
Healthy behavior markers						

Colonoscopy / Sigmoidoscopy	649 (12.7%)	594 (11.2%)	0.047	387 (11.7%)	389 (11.8%)	0.002
Flu Pneumococcal vaccine	1,429 (27.9%)	1,840 (34.6%)	0.143	983 (29.8%)	1,021 (31.0%)	0.025
Pap smear	503 (9.8%)	329 (6.2%)	0.135	256 (7.8%)	254 (7.7%)	0.002
PSA test	1,386 (27.1%)	1,337 (25.1%)	0.045	849 (25.8%)	864 (26.2%)	0.010
Fecal occult blood test	202 (3.9%)	196 (3.7%)	0.014	128 (3.9%)	124 (3.8%)	0.006
Bone mineral density tests	264 (5.2%)	250 (4.7%)	0.022	158 (4.8%)	151 (4.6%)	0.010
Mammograms	1,338 (26.2%)	1,153 (21.7%)	0.106	808 (24.5%)	794 (24.1%)	0.010
Telemedicine	1,575 (30.8%)	1,601 (30.1%)	0.016	985 (29.9%)	1,002 (30.4%)	0.011
**Laboratory and diagnostic tests**						
HbA1c tests; mean (SD) median [IQR]	2.34 ±1.54 2.00 [1.00, 3.00]	2.36 ±1.49 2.00 [1.00, 3.00]	0.010	2.35 ±1.52 2.00 [1.00, 3.00]	2.33 ±1.46 2.00 [1.00, 3.00]	0.010
Lipid panels; mean (SD) median [IQR]	1.76 ±1.38 2.00 [1.00, 2.00]	1.60 ±1.28 1.00 [1.00, 2.00]	0.119	1.68 ±1.24 2.00 [1.00, 2.00]	1.69 ±1.33 1.00 [1.00, 2.00]	0.005
Creatinine tests; mean (SD) median [IQR]	0.04 ±0.28 0.00 [0.00, 0.00]	0.06 ±0.35 0.00 [0.00, 0.00]	0.056	0.05 ±0.30 0.00 [0.00, 0.00]	0.05 ±0.31 0.00 [0.00, 0.00]	0.005
Natriuretic peptide tests; mean (SD) median [IQR]	0.20 ±0.98 0.00 [0.00, 0.00]	0.22 ±0.87 0.00 [0.00, 0.00]	0.020	0.20 ±0.94 0.00 [0.00, 0.00]	0.21 ±0.88 0.00 [0.00, 0.00]	0.015
Urine tests; mean (SD) median [IQR]	1.13 ±1.85 1.00 [0.00, 2.00]	1.10 ±1.85 0.00 [0.00, 2.00]	0.014	1.06 ±1.77 0.00 [0.00, 1.00]	1.09 ±1.86 0.00 [0.00, 1.00]	0.020
**Burden of comorbidities**						
Combined comorbidity score; mean (SD) median [IQR]	1.78 ±2.02 1.00 [0.00, 3.00]	2.34 ±2.50 2.00 [1.00, 3.00]	0.249	1.91 ±2.10 1.00 [0.00, 3.00]	1.93 ±2.15 1.00 [0.00, 3.00]	0.010
Frailty Score; mean (SD) median [IQR]	0.16 ±0.04 0.16 [0.13, 0.19]	0.18 ±0.05 0.17 [0.14, 0.20]	0.244	0.17 ±0.05 0.16 [0.14, 0.19]	0.17 ±0.05 0.16 [0.14, 0.19]	0.018
**Baseline hospitalizations and hospital metrics**						
Number of Hospitalizations; mean (SD) median [IQR]	6.19 ±25.28 0.00 [0.00, 0.00]	10.50 ±32.68 0.00 [0.00, 0.00]	0.147	7.19 ±26.84 0.00 [0.00, 0.00]	7.49 ±27.27 0.00 [0.00, 0.00]	0.011
Any hospitalization within prior 91 days; n (%)	147 (2.9%)	257 (4.8%)	0.102	107 (3.2%)	122 (3.7%)	0.025
Any hospitalization within prior 92-365 days; n (%)	468 (9.2%)	752 (14.1%)	0.156	343 (10.4%)	344 (10.4%)	0.001
Number of hospitalizations (0, 1, 2 or more)						
< 1	4,552 (89.0%)	4,402 (82.7%)	0.182	2,882 (87.4%)	2,867 (87.0%)	0.014
1 - <2	30 (0.6%)	45 (0.8%)	0.031	22 (0.7%)	17 (0.5%)	0.020
>= 2	532 (10.4%)	877 (16.5%)	0.179	393 (11.9%)	413 (12.5%)	0.019
Heart failure hospitalization	144 (2.8%)	304 (5.7%)	0.144	106 (3.2%)	118 (3.6%)	0.020
ED visit	1,894 (37.0%)	2,230 (41.9%)	0.099	1,249 (37.9%)	1,267 (38.4%)	0.011
**Calendar year of cohort entry**						
2022	855 (16.7%)	2,327 (43.7%)	0.615	812 (24.6%)	788 (23.9%)	0.017
2023	4,259 (83.3%)	2,997 (56.3%)	0.615	2,485 (75.4%)	2,509 (76.1%)	0.017

Abbreviations: *not used in the propensity score; ACE, angiotensin-converting enzyme inhibitors; ARB, angiotensin receptor blocker; ARNI, angiotensin receptor/neprilysin inhibitor; BMI, body mass index; CABG, coronary artery bypass graft surgery; CED, cohort entry date; CKD, chronic kidney disease; COPD, chronic obstructive pulmonary disease; DKA, diabetic ketoacidosis; DPP4i, dipeptidyl peptidase-4 inhibitors; ED, emergency department; eGFR, estimated glomerular filtration rate (estimated using the quadratic GFR equation: GFR = EXP(1.911 + (5.249 / Serum creatinine) - (2.114 / (Serum creatinine^2)) - (0.00686 * Age) - 0.205 (if female))); HbA1c, hemoglobin A1c; HDL, high-density lipoprotein cholesterol; HONK, hyperglycemic hyperosmolar nonketotic state; IQR, inter-quartile-range; MASH, metabolic dysfunction associated steatohepatitis; MASLD, metabolic dysfunction associated steatotic liver disease; MI, myocardial infarction; N, number of participants; NSAIDs, non-steroidal anti-inflammatory drugs; PCSK9, proprotein convertase subtilisin/kexin type 9; PSA, prostate-specific antigen; PTCA, percutaneous transluminal coronary angioplasty;

SGLT2i, sodium-glucose transport protein 2 inhibitors; SD, standard deviation; SMD, standardized mean difference; TIA, transient ischemic attack.
Missing data were handled by assuming absence of a code indicated absence of the condition for most binary covariates. Missing indicators were included for race and region in the propensity score model.

Supplementary Table 13. Baseline characteristics of initiators of tirzepatide vs dulaglutide when applying expanded eligibility criteria before and after propensity score matching, pooled databases. Values are number (percentage) unless otherwise specified.

	Before propensity score matching			After propensity score matching		
Variable	Tirzepatide (n = 82,742)	Dulaglutide (n = 53,347)	SMD	Tirzepatide (n = 39,152)	Dulaglutide (n = 39,152)	SMD
Demographics						
Age; mean (SD) median [IQR]	59.35 ± 11.64	61.52 ± 11.74	0.185	60.38 ± 11.45	60.38 ± 11.72	0.000
Gender						
Male	36,626 (44.3%)	25,181 (47.2%)	0.059	17,995 (46.0%)	17,971 (45.9%)	0.001
Female	46,116 (55,7%)	28,166 (52,8%)	0.059	21,157 (54,0%)	21,181 (54,1%)	0.001
Race						
White	26911 (45.6%)	14596 (43.7%)	0.113	10,954 (44.2%)	10,940 (44.1%)	0.001
Black	7226 (12.3%)	4928 (14.7%)	0.018	3,483 (14.0%)	3,471 (14.0%)	0.001
Unknown / Missing	23995 (40.7%)	13350 (39.9%)	0.090	9,991 (40.3%)	10,009 (40.3%)	0.001
Others	823 (1.4%)	546 (1.6%)	0.003	367 (1.5%)	375 (1.5%)	0.002
Region / State						
Northeast	6,476 (7.8%)	5,855 (11.0%)	0.108	3,689 (9.4%)	3,703 (9.5%)	0.001
Midwest / North central	17,966 (21.7%)	13,864 (26.0%)	0.100	9,574 (24.5%)	9,609 (24.5%)	0.002
South	48,782 (59.0%)	25,387 (47.6%)	0.229	20,441 (52.2%)	20,342 (52.0%)	0.005
West	9,465 (11.4%)	8,213 (15.4%)	0.116	5,430 (13.9%)	5,477 (14.0%)	0.003
Missing	53 (0.1%)	28 (0.1%)	0.005	18 (0.0%)	21 (0.1%)	0.003
Lifestyle risk factors						
Smoking / Tobacco use	15,635 (18.9%)	10,626 (19.9%)	0.026	7,498 (19.2%)	7,465 (19.1%)	0.002
Weight						
Overweight (25.0-29.9)	5,418 (6.5%)	6,151 (11.5%)	0.174	3,601 (9.2%)	3,610 (9.2%)	0.001
Class 1 Obesity (30.0-34.9)	12,207 (14.8%)	9,440 (17.7%)	0.080	6,388 (16.3%)	6,401 (16.3%)	0.001
Class 2 Obesity (35.0-39.9)	20,004 (24.2%)	11,075 (20.8%)	0.082	8,502 (21.7%)	8,501 (21.7%)	0.000
Class 3 Obesity (40.0 and above)	23,522 (28.4%)	13,073 (24.5%)	0.089	9,925 (25.3%)	9,921 (25.3%)	0.000
Unspecified Obesity	21,591 (26.1%)	13,608 (25.5%)	0.013	10,736 (27.4%)	10,719 (27.4%)	0.001
Diabetes complications						
Diabetic retinopathy	5,720 (6.9%)	5,616 (10.5%)	0.128	3,520 (9.0%)	3,540 (9.0%)	0.002
Diabetic neuropathy	17,091 (20.7%)	14,964 (28.1%)	0.173	9,816 (25.1%)	9,731 (24.9%)	0.005
Diabetic nephropathy	13,698 (16.6%)	11,955 (22.4%)	0.148	7,859 (20.1%)	7,673 (19.6%)	0.012
Diabetes with other opthalmic complications	2,082 (2.5%)	1,798 (3.4%)	0.051	1,150 (2.9%)	1,176 (3.0%)	0.004
Diabetes with peripheral circulatory disorders	9,310 (11.3%)	7,718 (14.5%)	0.096	5,044 (12.9%)	4,988 (12.7%)	0.004
Diabetic foot	1,921 (2.3%)	1,752 (3.3%)	0.058	1,106 (2.8%)	1,077 (2.8%)	0.004
Erectile dysfunction	3,716 (4.5%)	2,450 (4.6%)	0.005	1,811 (4.6%)	1,827 (4.7%)	0.002
Hypoglycemia	18,733 (22.6%)	12,697 (23.8%)	0.027	9,112 (23.3%)	9,024 (23.0%)	0.005
Hyperglycemia / DKA / HONK	40,752 (49.3%)	30,231 (56.7%)	0.149	21,187 (54.1%)	21,055 (53.8%)	0.007

Skin infections	8,282 (10.0%)	5,752 (10.8%)	0.025	4,104 (10.5%)	3,990 (10.2%)	0.010
Cardiovascular-related conditions						
Coronary atherosclerosis	14,000 (16.9%)	10,292 (19.3%)	0.062	7,030 (18.0%)	6,973 (17.8%)	0.004
Stable angina	2,965 (3.6%)	2,170 (4.1%)	0.025	1,464 (3.7%)	1,472 (3.8%)	0.001
Unstable angina	1,572 (1.9%)	1,226 (2.3%)	0.028	809 (2.1%)	796 (2.0%)	0.002
Hypertension	70,593 (85.3%)	45,971 (86.2%)	0.025	33,542 (85.7%)	33,459 (85.5%)	0.006
Hypotension	1,873 (2.3%)	1,605 (3.0%)	0.047	1,010 (2.6%)	983 (2.5%)	0.004
Hyperlipidemia	68,565 (82.9%)	44,491 (83.4%)	0.014	32,542 (83.1%)	32,485 (83.0%)	0.004
Acute MI	646 (0.8%)	662 (1.2%)	0.046	390 (1.0%)	396 (1.0%)	0.002
Old MI	2,443 (3.0%)	2,076 (3.9%)	0.052	1,296 (3.3%)	1,288 (3.3%)	0.001
Ischemic stroke	408 (0.5%)	516 (1.0%)	0.056	263 (0.7%)	274 (0.7%)	0.003
TIA	1,087 (1.3%)	913 (1.7%)	0.033	572 (1.5%)	577 (1.5%)	0.001
Cardiac conduction disorder	3,702 (4.5%)	2,857 (5.4%)	0.041	1,862 (4.8%)	1,863 (4.8%)	0.000
Previous cardiac procedure (CABG, PTCA, Stent)	982 (1.2%)	845 (1.6%)	0.034	555 (1.4%)	544 (1.4%)	0.002
PVD diagnosis or surgery	5,751 (7.0%)	4,839 (9.1%)	0.078	3,097 (7.9%)	3,099 (7.9%)	0.000
Atrial fibrillation	6,515 (7.9%)	4,723 (8.9%)	0.035	3,167 (8.1%)	3,160 (8.1%)	0.001
Other cardiac dysrhythmia	14,775 (17.9%)	9,793 (18.4%)	0.013	6,857 (17.5%)	6,842 (17.5%)	0.001
Heart failure	8,466 (10.2%)	6,644 (12.5%)	0.070	4,405 (11.3%)	4,289 (11.0%)	0.009
Acute heart failure	1,913 (2.3%)	1,736 (3.3%)	0.057	1,061 (2.7%)	1,058 (2.7%)	0.000
Cardiomyopathy	3,215 (3.9%)	2,558 (4.8%)	0.045	1,674 (4.3%)	1,671 (4.3%)	0.000
Valve disorders	7,171 (8.7%)	5,027 (9.4%)	0.026	3,444 (8.8%)	3,444 (8.8%)	0.000
Valve replacement	690 (0.8%)	511 (1.0%)	0.013	341 (0.9%)	347 (0.9%)	0.002
Edema	9,721 (11.7%)	6,425 (12.0%)	0.009	4,542 (11.6%)	4,452 (11.4%)	0.007
Venous thromboembolism / Pulmonary embolism	2,363 (2.9%)	1,714 (3.2%)	0.021	1,159 (3.0%)	1,143 (2.9%)	0.002
Pulmonary hypertension	1,915 (2.3%)	1,313 (2.5%)	0.010	911 (2.3%)	906 (2.3%)	0.001
Implantable cardioverter defibrillator	174 (0.2%)	150 (0.3%)	0.014	100 (0.3%)	94 (0.2%)	0.003
Hyperkalemia	1,712 (2.1%)	1,709 (3.2%)	0.071	1,007 (2.6%)	967 (2.5%)	0.007
Cerebrovascular procedure	62 (0.1%)	69 (0.1%)	0.017	36 (0.1%)	39 (0.1%)	0.002
Insertion of pacemakers / removal of cardiac lead	96 (0.1%)	83 (0.2%)	0.011	47 (0.1%)	51 (0.1%)	0.003
Renal-related conditions						
Hypertensive nephropathy	7,631 (9.2%)	6,666 (12.5%)	0.105	4,327 (11.1%)	4,215 (10.8%)	0.009
CKD Stage 1-2	2,919 (3.5%)	2,026 (3.8%)	0.014	1,410 (3.6%)	1,409 (3.6%)	0.000
CKD Stage 3-4	8,085 (9.8%)	6,530 (12.2%)	0.079	4,366 (11.2%)	4,287 (10.9%)	0.006
Unspecified CKD	3,436 (4.2%)	3,106 (5.8%)	0.077	1,953 (5.0%)	1,912 (4.9%)	0.005
Microalbuminuria or proteinuria	4,358 (5.3%)	3,471 (6.5%)	0.053	2,342 (6.0%)	2,317 (5.9%)	0.003
Acute kidney injury	3,642 (4.4%)	3,591 (6.7%)	0.102	2,165 (5.5%)	2,087 (5.3%)	0.009
Urinary tract infections	9,784 (11.8%)	6,616 (12.4%)	0.018	4,630 (11.8%)	4,625 (11.8%)	0.000
Genital infections	2,500 (3.0%)	2,035 (3.8%)	0.044	1,376 (3.5%)	1,359 (3.5%)	0.002
Urolithiasis (Kidney and urinary stone)	3,685 (4.5%)	2,282 (4.3%)	0.009	1,701 (4.3%)	1,664 (4.3%)	0.005

**Other comorbidities**						
COPD	7,648 (9.2%)	5,775 (10.8%)	0.053	3,906 (10.0%)	3,846 (9.8%)	0.005
Asthma	9,774 (11.8%)	5,685 (10.7%)	0.037	4,357 (11.1%)	4,290 (11.0%)	0.005
Obstructive sleep apnea	27,386 (33.1%)	15,039 (28.2%)	0.107	11,636 (29.7%)	11,597 (29.6%)	0.002
Serious bacterial infections	1,940 (2.3%)	1,861 (3.5%)	0.068	1,140 (2.9%)	1,106 (2.8%)	0.005
Pneumonia	3,075 (3.7%)	2,541 (4.8%)	0.052	1,604 (4.1%)	1,576 (4.0%)	0.004
Liver disease	14,011 (16.9%)	8,521 (16.0%)	0.026	6,351 (16.2%)	6,236 (15.9%)	0.008
MASH / MASLD	8,790 (10.6%)	4,630 (8.7%)	0.066	3,692 (9.4%)	3,630 (9.3%)	0.005
Fractures / Falls	3,964 (4.8%)	3,165 (5.9%)	0.051	2,065 (5.3%)	2,021 (5.2%)	0.005
Osteoporosis	2,625 (3.2%)	1,818 (3.4%)	0.013	1,214 (3.1%)	1,255 (3.2%)	0.006
Osteoarthritis	22,487 (27.2%)	14,201 (26.6%)	0.013	10,325 (26.4%)	10,334 (26.4%)	0.001
Depression	16,397 (19.8%)	10,861 (20.4%)	0.014	7,749 (19.8%)	7,679 (19.6%)	0.004
Dementia	1,622 (2.0%)	2,086 (3.9%)	0.116	1,053 (2.7%)	1,041 (2.7%)	0.002
Delirium or psychosis	852 (1.0%)	999 (1.9%)	0.071	531 (1.4%)	510 (1.3%)	0.005
Anxiety	19,164 (23.2%)	10,954 (20.5%)	0.064	8,427 (21.5%)	8,179 (20.9%)	0.015
Sleep disorders	27,068 (32.7%)	16,008 (30.0%)	0.058	11,988 (30.6%)	11,931 (30.5%)	0.003
Anemia	13,755 (16.6%)	9,339 (17.5%)	0.023	6,526 (16.7%)	6,466 (16.5%)	0.004
Influenza	1,885 (2.3%)	862 (1.6%)	0.048	709 (1.8%)	712 (1.8%)	0.001
COVID	9,419 (11.4%)	6,747 (12.6%)	0.039	4,697 (12.0%)	4,693 (12.0%)	0.000
Hyperthyroidism and other thyroid gland disorders	20,901 (25.3%)	12,165 (22.8%)	0.058	9,209 (23.5%)	9,094 (23.2%)	0.007
Hypothyroidism	16,783 (20.3%)	9,708 (18.2%)	0.053	7,418 (18.9%)	7,277 (18.6%)	0.009
Nephrotic syndrome	54 (0.1%)	58 (0.1%)	0.015	33 (0.1%)	30 (0.1%)	0.003
Urinary incontinence	3,790 (4.6%)	2,671 (5.0%)	0.020	1,823 (4.7%)	1,821 (4.7%)	0.000
Biliary disease	125 (0.2%)	133 (0.2%)	0.022	76 (0.2%)	73 (0.2%)	0.002
Pancreatitis	85 (0.1%)	65 (0.1%)	0.006	48 (0.1%)	39 (0.1%)	0.007
Bowel obstruction	107 (0.1%)	72 (0.1%)	0.002	49 (0.1%)	51 (0.1%)	0.001
Gastroparesis	669 (0.8%)	449 (0.8%)	0.004	318 (0.8%)	326 (0.8%)	0.002
**Diabetes medications**						
Number of antidiabetic drugs on CED, mean (SD)	2.07 ± 0.97	2.32 ± 1.00	0.255	2.24 ± 1.01	2.24 ± 0.98	0.006
Concomitant use or initiation of Metformin	40,386 (48.8%)	27,953 (52.4%)	0.072	20,297 (51.8%)	20,367 (52.0%)	0.004
Concomitant use or initiation of Insulins	13,477 (16.3%)	12,653 (23.7%)	0.187	8,197 (20.9%)	8,115 (20.7%)	0.005
Concomitant use or initiation of Sulfonylureas	10,960 (13.2%)	10,963 (20.6%)	0.196	6,974 (17.8%)	6,911 (17.7%)	0.004
Concomitant use or initiation of DPP-4i	4,645 (5.6%)	4,146 (7.8%)	0.086	2,725 (7.0%)	2,691 (6.9%)	0.003
Concomitant use or initiation of SGLT-2i	14,932 (18.0%)	11,325 (21.2%)	0.080	8,143 (20.8%)	8,161 (20.8%)	0.001
Concomitant use or initiation of Any other glucose-lowering drugs	4,193 (5.1%)	3,291 (6.2%)	0.048	2,316 (5.9%)	2,307 (5.9%)	0.001
Past use of Metformin	54,165 (65.5%)	36,958 (69.3%)	0.081	26,869 (68.6%)	26,890 (68.7%)	0.001
Past use of Insulins	18,087 (21.9%)	16,910 (31.7%)	0.224	11,011 (28.1%)	10,896 (27.8%)	0.007
Past use of Sulfonylureas	15,950 (19.3%)	15,386 (28.8%)	0.225	9,990 (25.5%)	9,843 (25.1%)	0.009
Past use of DPP-4i	7,470 (9.0%)	6,696 (12.6%)	0.114	4,391 (11.2%)	4,347 (11.1%)	0.004

Past use of SGLT-2i	20,419 (24.7%)	15,422 (28.9%)	0.096	11,061 (28.3%)	11,049 (28.2%)	0.001
Past use of Any other glucose-lowering drugs	5,912 (7.1%)	4,765 (8.9%)	0.066	3,314 (8.5%)	3,268 (8.3%)	0.004
Other medications						
ACE / ARB	58,717 (71.0%)	39,349 (73.8%)	0.063	28,583 (73.0%)	28,472 (72.7%)	0.006
ARNI	1,293 (1.6%)	865 (1.6%)	0.005	627 (1.6%)	624 (1.6%)	0.001
Thiazides	29,221 (35.3%)	17,662 (33.1%)	0.047	13,220 (33.8%)	13,261 (33.9%)	0.002
Beta-blockers	31,185 (37.7%)	21,595 (40.5%)	0.057	15,200 (38.8%)	15,182 (38.8%)	0.001
Calcium channel blockers	25,269 (30.5%)	17,293 (32.4%)	0.040	12,321 (31.5%)	12,286 (31.4%)	0.002
Digoxin / Digitoxin	370 (0.4%)	331 (0.6%)	0.024	184 (0.5%)	200 (0.5%)	0.006
Loop diuretics	12,576 (15.2%)	8,827 (16.5%)	0.037	6,147 (15.7%)	6,040 (15.4%)	0.008
Other diuretics	7,218 (8.7%)	4,547 (8.5%)	0.007	3,347 (8.5%)	3,342 (8.5%)	0.000
Intravenous diuretics	859 (1.0%)	698 (1.3%)	0.025	437 (1.1%)	446 (1.1%)	0.002
Nitrates	4,058 (4.9%)	3,362 (6.3%)	0.061	2,199 (5.6%)	2,202 (5.6%)	0.000
Anti-arrhythmics	1,662 (2.0%)	1,006 (1.9%)	0.009	697 (1.8%)	746 (1.9%)	0.009
Statins	62,048 (75.0%)	43,450 (81.4%)	0.157	31,016 (79.2%)	31,109 (79.5%)	0.006
PCSK9 inhibitors and other lipid-lowering drugs	11,075 (13.4%)	6,930 (13.0%)	0.012	5,262 (13.4%)	5,154 (13.2%)	0.008
Antiplatelet medications	6,952 (8.4%)	5,637 (10.6%)	0.074	3,764 (9.6%)	3,723 (9.5%)	0.004
Oral anticoagulants	7,202 (8.7%)	5,277 (9.9%)	0.041	3,538 (9.0%)	3,540 (9.0%)	0.000
COPD / Asthma medications	29,752 (36.0%)	17,848 (33.5%)	0.053	13,489 (34.5%)	13,344 (34.1%)	0.008
NSAIDS	28,241 (34.1%)	16,980 (31.8%)	0.049	12,818 (32.7%)	12,802 (32.7%)	0.001
Oral corticosteroids	23,298 (28.2%)	12,077 (22.6%)	0.127	9,494 (24.2%)	9,504 (24.3%)	0.001
Osteoporosis agents (incl. bisphosphonates)	1,679 (2.0%)	1,268 (2.4%)	0.024	856 (2.2%)	871 (2.2%)	0.003
Opioids	24,284 (29.3%)	15,038 (28.2%)	0.026	11,132 (28.4%)	11,088 (28.3%)	0.002
Anti-depressants	32,530 (39.3%)	20,053 (37.6%)	0.035	15,062 (38.5%)	14,840 (37.9%)	0.012
Antipsychotics	3,415 (4.1%)	2,439 (4.6%)	0.022	1,707 (4.4%)	1,642 (4.2%)	0.008
Anxiolytics / hypnotics, benzos	18,439 (22.3%)	10,158 (19.0%)	0.080	7,976 (20.4%)	7,842 (20.0%)	0.009
Dementia medications	687 (0.8%)	866 (1.6%)	0.072	450 (1.1%)	435 (1.1%)	0.004
Urinary tract infections antibiotics	39,307 (47.5%)	23,649 (44.3%)	0.064	17,746 (45.3%)	17,734 (45.3%)	0.001
Laxatives	2,752 (3.3%)	1,786 (3.3%)	0.001	1,278 (3.3%)	1,292 (3.3%)	0.002
Healthcare utilization marker						
Number of distinct medications; mean (SD)	14.16 ± 7.05	14.52 ± 6.92	0.051	14.40 ± 6.95	14.31 ± 6.92	0.013
Number of office visits; mean (SD)	9.28 ± 6.95	9.00 ± 6.78	0.042	9.02 ± 6.57	8.99 ± 6.83	0.005
Number of endocrinologist visits; mean (SD)	0.42 ± 1.35	0.45 ± 1.47	0.026	0.44 ± 1.31	0.44 ± 1.43	0.005
Number of cardiologist visits; mean (SD)	1.30 ± 3.03	1.37 ± 3.32	0.023	1.29 ± 2.97	1.30 ± 3.16	0.002
Number of internal/family medicine visits; mean (SD)	6.07 ± 7.05	6.84 ± 8.69	0.098	6.35 ± 7.79	6.31 ± 7.57	0.005
Number of electrocardiograms (ECG/EKG); mean (SD)	0.90 ± 1.60	0.98 ± 1.73	0.048	0.93 ± 1.60	0.93 ± 1.64	0.000
Number of echocardiograms; mean (SD)	0.26 ± 0.62	0.29 ± 0.69	0.046	0.28 ± 0.63	0.28 ± 0.68	0.000
Out-of-pocket medication cost; mean (SD)	569.35 ± 863.19	576.16 ± 837.62	0.008	578.40 ± 784.43	574.50 ± 857.82	0.005
Unique brand medicines; mean (SD)	14.39 ± 7.25	14.79 ± 7.16	0.056	14.65 ± 7.17	14.56 ± 7.14	0.013

Unique generic medicines; mean (SD)	14.16 ± 7.05	14.52 ± 6.92	0.051	14.40 ± 6.95	14.31 ± 6.92	0.013
Ratio of brand to generic medications; mean (SD)	1.01 ± 0.04	1.02 ± 0.04	0.250	1.02 ± 0.04	1.02 ± 0.04	0.000
**Healthy behavior markers**						
Colonoscopy / Sigmoidoscopy	8,977 (10.8%)	5,300 (9.9%)	0.030	4,034 (10.3%)	4,047 (10.3%)	0.001
Flu Pneumococcal vaccine	23,845 (28.8%)	17,828 (33.4%)	0.099	12,399 (31.7%)	12,410 (31.7%)	0.001
Pap smear	7,622 (9.2%)	3,724 (7.0%)	0.082	3,016 (7.7%)	3,061 (7.8%)	0.004
PSA test	18,449 (22.3%)	10,979 (20.6%)	0.042	8,323 (21.3%)	8,265 (21.1%)	0.004
Fecal occult blood test	2,999 (3.6%)	2,146 (4.0%)	0.021	1,494 (3.8%)	1,469 (3.8%)	0.003
Bone mineral density tests	5,159 (6.2%)	2,929 (5.5%)	0.032	2,118 (5.4%)	2,226 (5.7%)	0.012
Mammograms	22,536 (27.2%)	12,561 (23.5%)	0.085	9,746 (24.9%)	9,830 (25.1%)	0.005
Telemedicine	20,969 (25.3%)	13,447 (25.2%)	0.003	9,743 (24.9%)	9,719 (24.8%)	0.001
**Laboratory and diagnostic tests**						
HbA1c tests; mean (SD)	2.29 ± 1.31	2.47 ± 1.38	0.134	2.41 ± 1.38	2.41 ± 1.32	0.000
Lipid panels; mean (SD)	1.62 ± 1.15	1.54 ± 1.28	0.071	1.57 ± 1.15	1.57 ± 1.18	0.001
Creatinine tests; mean (SD)	1.98 ± 2.67	1.94 ± 2.85	0.011	1.87 ± 2.82	1.87 ± 2.59	0.002
Natriuretic peptide tests; mean (SD)	0.15 ± 0.67	0.17 ± 0.86	0.026	0.16 ± 0.70	0.15 ± 0.77	0.014
Urine tests; mean (SD)	0.99 ± 1.61	0.98 ± 1.70	0.009	0.97 ± 1.60	0.97 ± 1.73	0.000
**Lab values**						
HbA1c (%)*; mean (SD) median [IQR]	7.59 ±1.71 7.10 [6.40, 8.40]	8.23 ±1.84 7.90 [6.90, 9.20]	0.361	7.81 ±1.77 7.40 [6.50, 8.70]	8.13 ±1.83 7.80 [6.80, 9.10]	0.178
Glucose (mg/dl)*; mean (SD) median [IQR]	154.81 ±66.88 137.00 [111.00, 179.00]	709.96 ±73,034.26 158.00 [124.00, 210.00]	0.011	161.44 ±70.21 143.50 [114.00, 189.00]	876.61 ±83,854.72 155.00 [121.00, 206.00]	0.012
Creatinine (mg/dl)*; mean (SD) median [IQR]	1.01 ±1.32 0.89 [0.80, 1.08]	1.06 ±1.07 0.92 [0.80, 1.15]	0.043	1.05 ±1.94 0.91 [0.80, 1.12]	1.04 ±1.14 0.91 [0.80, 1.12]	0.006
Systolic blood pressure (mmHg)*; mean (SD) median [IQR]	129.38 ±22.89 130.00 [120.00, 139.00]	130.47 ±26.86 130.00 [120.00, 140.00]	0.043	129.90 ±22.63 130.00 [120.00, 140.00]	130.13 ±27.13 130.00 [120.00, 140.00]	0.009
Heart rate (1/min)*; mean (SD) median [IQR]	78.57 ±15.04 78.00 [69.00, 87.00]	78.76 ±17.40 78.00 [69.00, 87.00]	0.012	78.37 ±14.56 77.00 [69.00, 87.00]	78.81 ±18.29 78.00 [69.00, 87.00]	0.027
BMI (kg/m2)*; mean (SD) median [IQR]	38.70 ±8.05 37.40 [33.05, 42.77]	36.44 ±7.54 35.15 [31.07, 40.30]	0.290	37.78 ±7.93 36.42 [32.12, 41.66]	36.88 ±7.60 35.60 [31.46, 40.81]	0.116
eGFR (ml/min/1.73m2)*; mean (SD) median [IQR]	2,420.68 ±152,973.25 83.00 [64.00, 98.00]	1,525.25 ±120,389.29 78.00 [58.00, 95.00]	0.007	2,618.84 ±159,419.94 79.00 [60.00, 95.00]	1,886.79 ±134,527.43 79.00 [60.00, 96.00]	0.005
LDL (mg/dl)*; mean (SD) median [IQR]	87.31 ±39.64 83.00 [62.00, 110.00]	84.02 ±39.58 79.00 [59.00, 105.00]	0.083	85.07 ±39.55 81.00 [60.00, 107.00]	84.53 ±39.87 80.00 [59.00, 106.00]	0.013
HDL (mg/dl)*; mean (SD) median [IQR]	46.04 ±13.91 45.00 [37.00, 53.00]	45.02 ±13.75 43.00 [36.00, 53.00]	0.074	45.76 ±13.91 44.00 [37.00, 53.00]	45.04 ±13.77 44.00 [36.00, 52.00]	0.052
Total cholesterol (mg/dl)*; mean (SD) median [IQR]	165.47 ±49.63 161.00 [135.00, 192.00]	162.43 ±50.77 157.00 [131.00, 189.00]	0.061	162.99 ±50.08 158.00 [133.00, 189.00]	163.02 ±51.02 157.00 [131.00, 190.00]	0.001
Triglyceride (mg/dl)*; mean (SD) median [IQR]	175.63 ±149.62 144.00 [104.00, 205.00]	181.79 ±168.10 148.00 [106.00, 210.00]	0.039	176.96 ±151.29 144.00 [103.00, 207.00]	182.12 ±166.70 148.00 [105.00, 210.00]	0.032
**Burden of comorbidities**						
Combined comorbidity score; mean (SD)	1.55 ± 2.14	1.88 ± 2.40	0.144	1.70 ± 2.26	1.68 ± 2.22	0.011
Frailty Score; mean (SD)	0.15 ± 0.05	0.16 ± 0.05	0.161	0.16 ± 0.06	0.16 ± 0.06	0.000
**Baseline hospitalizations and hospital metrics**						

Number of Hospitalizations; mean (SD)	1.22 ± 10.89	2.34 ± 16.25	0.081	1.81 ± 13.73	1.75 ± 14.13	0.004
Any hospitalization within prior 91 days	2,638 (3.2%)	2,697 (5.1%)	0.094	1,555 (4.0%)	1,539 (3.9%)	0.002
Any hospitalization within prior 92-365 days	6,408 (7.7%)	5,380 (10.1%)	0.082	3,401 (8.7%)	3,355 (8.6%)	0.004
Number of hospitalizations (0, 1, 2 or more)						
< 1	74,621 (90.2%)	46,268 (86.7%)	0.108	34,759 (88.8%)	34,798 (88.9%)	0.003
1 - <2	4,713 (5.7%)	3,340 (6.3%)	0.024	2,234 (5.7%)	2,239 (5.7%)	0.001
>= 2	3,408 (4.1%)	3,739 (7.0%)	0.126	2,159 (5.5%)	2,115 (5.4%)	0.005
Heart failure hospitalization	2,021 (2.4%)	1,961 (3.7%)	0.072	1,154 (2.9%)	1,153 (2.9%)	0.000
ED visit	22,106 (26.7%)	17,164 (32.2%)	0.120	11,738 (30.0%)	11,570 (29.6%)	0.009
**Calendar year of cohort entry**						
2022	9,402 (11.4%)	19,979 (37.5%)	0.637	8,863 (22.6%)	8,884 (22.7%)	0.001
2023	43,132 (52.1%)	25,595 (48.0%)	0.083	22,676 (57.9%)	22,685 (57.9%)	0.000
2024	28,830 (34.8%)	7,632 (14.3%)	0.491	7,473 (19.1%)	7,442 (19.0%)	0.002
2025	1,378 (1.7%)	141 (0.3%)	0.144	140 (0.4%)	141 (0.4%)	0.000

Abbreviations: *not used in the propensity score; ACE, angiotensin-converting enzyme inhibitors; ARB, angiotensin receptor blocker; ARNI, angiotensin receptor/neprilysin inhibitor; BMI, body mass index; CABG, coronary artery bypass graft surgery; CED, cohort entry date; CKD, chronic kidney disease; COPD, chronic obstructive pulmonary disease; DKA, diabetic ketoacidosis; DPP4i, dipeptidyl peptidase-4 inhibitors; ED, emergency department; eGFR, estimated glomerular filtration rate (estimated using the quadratic GFR equation: GFR = EXP(1.911 + (5.249 / Serum creatinine) - (2.114 / (Serum creatinine^2)) - (0.00686 * Age) - 0.205 (if female))); HbA1c, hemoglobin A1c; HDL, high-density lipoprotein cholesterol; HONK, hyperglycemic hyperosmolar nonketotic state; IQR, inter-quartile-range; MASH, metabolic dysfunction associated steatohepatitis; MASLD, metabolic dysfunction associated steatotic liver disease; MI, myocardial infarction; N, number of participants; NSAIDs, non-steroidal anti-inflammatory drugs; PCSK9, proprotein convertase subtilisin/kexin type 9; PSA, prostate-specific antigen; PTCA, percutaneous transluminal coronary angioplasty; SGLT2i, sodium-glucose transport protein 2 inhibitors; SD, standard deviation; SMD, standardized mean difference; TIA, transient ischemic attack.

Missing data were handled by assuming absence of a code indicated absence of the condition for most binary covariates. Missing indicators were included for race and region in the propensity score model.

Laboratory values were only available in a subset of the Optum database and were truncated using clinically plausible cut-off values (BMI values >100 or <10 were set to missing; missingness before matching ~54%. Creatinine values >30 were set to missing, and values <0.8 were set to 0.8; missingness before matching ~41%. eGFR values >150 were set to 150, and values =0 were set to missing; missingness before matching ~53%. Glucose values <30 were set to missing; missingness before matching ~41%. HbA1c values ≥20 or ≤2 were set to missing; missingness before matching ~36%. HDL value missingness before matching was ~48%. Heart rate values <30 were set to missing; missingness before matching ~67%. LDL value missingness before matching was~46%. Systolic blood pressure values <30 were set to missing; missingness before matching ~51%. Total cholesterol value missingness before matching was ~48%. Triglyceride value missingness before matching was ~49%).

Race was reported by the respective database: Optum. The Race-Others category includes Asian and Hispanic individuals.

Supplementary Table 14. Baseline characteristics of initiators of tirzepatide vs dulaglutide when applying expanded eligibility criteria before and after propensity score matching. Optum database. Values are number (percentage) unless otherwise specified

	Before propensity score matching			After propensity score matching		
Variable	Tirzepatide (n = 58,955)	Dulaglutide (n = 33,420)	SMD	Tirzepatide (n = 24,795)	Dulaglutide (n = 24,795)	SMD
Demographics						
Age; mean (SD) median [IQR]	61.57 ±11.51 64.00 [54.00, 70.00]	64.56 ±11.07 67.00 [58.00, 72.00]	0.265	63.47 ±11.02 66.00 [56.00, 71.00]	63.49 ±11.23 66.00 [56.00, 71.00]	0.001
Race						
White	26,911 (45.6%)	14,596 (43.7%)	0.040	10,954 (44.2%)	10,940 (44.1%)	0.001
Black	7,226 (12.3%)	4,928 (14.7%)	0.073	3,483 (14.0%)	3,471 (14.0%)	0.001
Unknown / Missing	23,995 (40.7%)	13,350 (39.9%)	0.015	9,991 (40.3%)	10,009 (40.4%)	0.001
Others	823 (1.4%)	546 (1.6%)	0.019	367 (1.5%)	375 (1.5%)	0.003
Region / State						
Northeast	4,411 (7.5%)	3,469 (10.4%)	0.102	2,190 (8.8%)	2,218 (8.9%)	0.004
Midwest / North central	12,719 (21.6%)	7,692 (23.0%)	0.035	5,767 (23.3%)	5,728 (23.1%)	0.004
South	34,090 (57.8%)	15,773 (47.2%)	0.214	12,603 (50.8%)	12,582 (50.7%)	0.002
West	7,700 (13.1%)	6,473 (19.4%)	0.172	4,229 (17.1%)	4,255 (17.2%)	0.003
Missing	35 (0.1%)	13 (0.0%)	0.001	6 (0.0%)	12 (0.0%)	0.012
Lifestyle risk factors						
Smoking / Tobacco use	13,408 (22.7%)	8,434 (25.2%)	0.058	6,039 (24.4%)	6,003 (24.2%)	0.003
Weight						
Overweight (25.0-29.9)	3,573 (6.1%)	3,734 (11.2%)	0.183	2,208 (8.9%)	2,222 (9.0%)	0.002
Class 1 Obesity (30.0-34.9)	10,345 (17.5%)	7,515 (22.5%)	0.124	5,153 (20.8%)	5,164 (20.8%)	0.001
Class 2 Obesity (35.0-39.9)	18,370 (31.2%)	9,753 (29.2%)	0.043	7,499 (30.2%)	7,510 (30.3%)	0.001
Class 3 Obesity (40.0 and above)	18,355 (31.1%)	7,889 (23.6%)	0.169	6,511 (26.3%)	6,468 (26.1%)	0.004
Unspecified Obesity	8,312 (14.1%)	4,529 (13.6%)	0.016	3,424 (13.8%)	3,431 (13.8%)	0.001
Diabetes complications						
Diabetic retinopathy	4,491 (7.6%)	4,065 (12.2%)	0.153	2,586 (10.4%)	2,611 (10.5%)	0.003
Diabetic neuropathy	13,958 (23.7%)	11,222 (33.6%)	0.220	7,500 (30.2%)	7,483 (30.2%)	0.001
Diabetic nephropathy	11,313 (19.2%)	9,023 (27.0%)	0.186	6,105 (24.6%)	5,962 (24.0%)	0.013
Diabetes with other opthalmic complications	2,082 (3.5%)	1,798 (5.4%)	0.09	1,150 (4.6%)	1,176 (4.7%)	0.005
Diabetes with peripheral circulatory disorders	7,667 (13.0%)	5,992 (17.9%)	0.137	3,924 (15.8%)	3,916 (15.8%)	0.001
Diabetic foot	1,608 (2.7%)	1,358 (4.1%)	0.074	875 (3.5%)	851 (3.4%)	0.005
Erectile dysfunction	2,918 (4.9%)	1,708 (5.1%)	0.007	1,271 (5.1%)	1,288 (5.2%)	0.003
Hypoglycemia	14,423 (24.5%)	9,056 (27.1%)	0.060	6,489 (26.2%)	6,437 (26.0%)	0.005
Hyperglycemia / DKA / HONK	29,255 (49.6%)	19,342 (57.9%)	0.166	13,691 (55.2%)	13,595 (54.8%)	0.008
Skin infections	6,255 (10.6%)	3,863 (11.6%)	0.030	2,792 (11.3%)	2,726 (11.0%)	0.008
Cardiovascular-related conditions						

Coronary atherosclerosis	11,680 (19.8%)	7,770 (23.2%)	0.084	5,438 (21.9%)	5,421 (21.9%)	0.002
Stable angina	2,540 (4.3%)	1,664 (5.0%)	0.032	1,158 (4.7%)	1,181 (4.8%)	0.004
Unstable angina	1,301 (2.2%)	883 (2.6%)	0.028	612 (2.5%)	603 (2.4%)	0.002
Hypertension	51,511 (87.4%)	29,914 (89.5%)	0.067	22,048 (88.9%)	21,996 (88.7%)	0.007
Hypotension	1,621 (2.7%)	1,290 (3.9%)	0.062	840 (3.4%)	818 (3.3%)	0.005
Hyperlipidemia	49,978 (84.8%)	28,764 (86.1%)	0.037	21,223 (85.6%)	21,186 (85.4%)	0.004
Acute MI	482 (0.8%)	430 (1.3%)	0.046	272 (1.1%)	262 (1.1%)	0.004
Old MI	2,189 (3.7%)	1,769 (5.3%)	0.076	1,116 (4.5%)	1,111 (4.5%)	0.001
Ischemic stroke	284 (0.5%)	316 (0.9%)	0.055	159 (0.6%)	177 (0.7%)	0.009
TIA	876 (1.5%)	681 (2.0%)	0.042	432 (1.7%)	440 (1.8%)	0.002
Cardiac conduction disorder	3,130 (5.3%)	2,179 (6.5%)	0.051	1,461 (5.9%)	1,475 (5.9%)	0.002
Previous cardiac procedure (CABG, PTCA, Stent)	772 (1.3%)	577 (1.7%)	0.034	403 (1.6%)	385 (1.6%)	0.006
PVD diagnosis or surgery	4,970 (8.4%)	3,923 (11.7%)	0.110	2,547 (10.3%)	2,573 (10.4%)	0.003
Atrial fibrillation	5,456 (9.3%)	3,542 (10.6%)	0.045	2,443 (9.9%)	2,436 (9.8%)	0.001
Other cardiac dysrhythmia	11,624 (19.7%)	6,939 (20.8%)	0.026	4,932 (19.9%)	4,953 (20.0%)	0.002
Heart failure	7,343 (12.5%)	5,287 (15.8%)	0.097	3,615 (14.6%)	3,543 (14.3%)	0.008
Acute heart failure	1,674 (2.8%)	1,374 (4.1%)	0.069	879 (3.5%)	884 (3.6%)	0.001
Cardiomyopathy	2,644 (4.5%)	1,930 (5.8%)	0.059	1,295 (5.2%)	1,292 (5.2%)	0.001
Valve disorders	5,942 (10.1%)	3,785 (11.3%)	0.040	2,630 (10.6%)	2,651 (10.7%)	0.003
Valve replacement	608 (1.0%)	398 (1.2%)	0.015	281 (1.1%)	286 (1.2%)	0.002
Edema	8,052 (13.7%)	4,894 (14.6%)	0.028	3,522 (14.2%)	3,469 (14.0%)	0.006
Venous thromboembolism / Pulmonary embolism	1,880 (3.2%)	1,226 (3.7%)	0.026	832 (3.4%)	821 (3.3%)	0.002
Pulmonary hypertension	1,689 (2.9%)	1,081 (3.2%)	0.022	768 (3.1%)	757 (3.1%)	0.003
Implantable cardioverter defibrillator	142 (0.2%)	105 (0.3%)	0.014	76 (0.3%)	74 (0.3%)	0.001
Hyperkalemia	1,476 (2.5%)	1,379 (4.1%)	0.091	829 (3.3%)	809 (3.3%)	0.005
Cerebrovascular procedure	53 (0.1%)	55 (0.2%)	0.021	28 (0.1%)	33 (0.1%)	0.006
Insertion of pacemakers / removal of cardiac lead	89 (0.2%)	68 (0.2%)	0.012	41 (0.2%)	44 (0.2%)	0.003
**Renal-related conditions**						
Hypertensive nephropathy	6,647 (11.3%)	5,451 (16.3%)	0.146	3,626 (14.6%)	3,537 (14.3%)	0.010
CKD Stage 1-2	2,919 (5.0%)	2,026 (6.1%)	0.049	1,410 (5.7%)	1,409 (5.7%)	0.000
CKD Stage 3-4	8,085 (13.7%)	6,530 (19.5%)	0.157	4,366 (17.6%)	4,287 (17.3%)	0.008
Unspecified CKD	3,084 (5.2%)	2,641 (7.9%)	0.108	1,690 (6.8%)	1,663 (6.7%)	0.004
Microalbuminuria or proteinuria	3,606 (6.1%)	2,630 (7.9%)	0.069	1,802 (7.3%)	1,793 (7.2%)	0.001
Acute kidney injury	3,067 (5.2%)	2,810 (8.4%)	0.128	1,745 (7.0%)	1,692 (6.8%)	0.008
Urinary tract infections	7,575 (12.8%)	4,841 (14.5%)	0.048	3,402 (13.7%)	3,371 (13.6%)	0.004
Genital infections	1,632 (2.8%)	1,217 (3.6%)	0.050	806 (3.3%)	799 (3.2%)	0.002
Urolithiasis (Kidney and urinary stone)	2,751 (4.7%)	1,480 (4.4%)	0.011	1,144 (4.6%)	1,096 (4.4%)	0.009
**Other comorbidities**						
COPD	6,763 (11.5%)	4,782 (14.3%)	0.085	3,301 (13.3%)	3,268 (13.2%)	0.004

Asthma	7,580 (12.9%)	3,955 (11.8%)	0.031	3,066 (12.4%)	3,028 (12.2%)	0.005
Obstructive sleep apnea	20,297 (34.4%)	9,689 (29.0%)	0.117	7,615 (30.7%)	7,614 (30.7%)	0.000
Serious bacterial infections	1,435 (2.4%)	1,244 (3.7%)	0.075	770 (3.1%)	752 (3.0%)	0.004
Pneumonia	2,449 (4.2%)	1,858 (5.6%)	0.065	1,193 (4.8%)	1,178 (4.8%)	0.003
Liver disease	10,714 (18.2%)	5,938 (17.8%)	0.011	4,468 (18.0%)	4,361 (17.6%)	0.011
MASH / MASLD	6,574 (11.2%)	3,047 (9.1%)	0.067	2,473 (10.0%)	2,418 (9.8%)	0.007
Fractures / Falls	3,277 (5.6%)	2,422 (7.2%)	0.069	1,604 (6.5%)	1,562 (6.3%)	0.007
Osteoporosis	2,352 (4.0%)	1,545 (4.6%)	0.031	1,034 (4.2%)	1,069 (4.3%)	0.007
Osteoarthritis	17,956 (30.5%)	10,341 (30.9%)	0.011	7,606 (30.7%)	7,609 (30.7%)	0.000
Depression	12,691 (21.5%)	7,747 (23.2%)	0.040	5,513 (22.2%)	5,453 (22.0%)	0.006
Dementia	1,475 (2.5%)	1,775 (5.3%)	0.145	934 (3.8%)	925 (3.7%)	0.002
Delirium or psychosis	767 (1.3%)	859 (2.6%)	0.092	458 (1.8%)	443 (1.8%)	0.005
Anxiety	14,178 (24.0%)	7,344 (22.0%)	0.049	5,657 (22.8%)	5,471 (22.1%)	0.018
Sleep disorders	21,114 (35.8%)	11,586 (34.7%)	0.024	8,660 (34.9%)	8,585 (34.6%)	0.006
Anemia	10,461 (17.7%)	6,622 (19.8%)	0.053	4,595 (18.5%)	4,594 (18.5%)	0.000
Influenza	1,283 (2.2%)	567 (1.7%)	0.035	437 (1.8%)	458 (1.8%)	0.006
COVID	6,286 (10.7%)	3,995 (12.0%)	0.041	2,804 (11.3%)	2,773 (11.2%)	0.004
Hyperthyroidism and other thyroid gland disorders	15,598 (26.5%)	8,261 (24.7%)	0.040	6,307 (25.4%)	6,213 (25.1%)	0.009
Hypothyroidism	12,630 (21.4%)	6,685 (20.0%)	0.035	5,136 (20.7%)	5,031 (20.3%)	0.010
Nephrotic syndrome	46 (0.1%)	43 (0.1%)	0.016	26 (0.1%)	24 (0.1%)	0.003
Urinary incontinence	3,185 (5.4%)	2,132 (6.4%)	0.042	1,435 (5.8%)	1,448 (5.8%)	0.002
Biliary disease	68 (0.1%)	70 (0.2%)	0.023	38 (0.2%)	39 (0.2%)	0.001
Pancreatitis	57 (0.1%)	38 (0.1%)	0.005	30 (0.1%)	25 (0.1%)	0.006
Bowel obstruction	76 (0.1%)	44 (0.1%)	0.001	28 (0.1%)	32 (0.1%)	0.005
Gastroparesis	541 (0.9%)	333 (1.0%)	0.008	242 (1.0%)	245 (1.0%)	0.001
Diabetes medications						
Number of antidiabetic drugs on CED, mean (SD)	2.08 ±0.96 2.00 [1.00, 3.00]	2.36 ±1.00 2.00 [2.00, 3.00]	0.284	2.28 ±1.01 2.00 [2.00, 3.00]	2.27 ±0.98 2.00 [2.00, 3.00]	0.006
Concomitant use or initiation of Metformin	28,425 (48.2%)	17,289 (51.7%)	0.070	12,694 (51.2%)	12,749 (51.4%)	0.004
Concomitant use or initiation of Insulins	10,264 (17.4%)	8,833 (26.4%)	0.219	5,809 (23.4%)	5,751 (23.2%)	0.006
Concomitant use or initiation of Sulfonylureas	8,369 (14.2%)	7,454 (22.3%)	0.211	4,931 (19.9%)	4,854 (19.6%)	0.008
Concomitant use or initiation of DPP-4i	3,087 (5.2%)	2,479 (7.4%)	0.090	1,642 (6.6%)	1,603 (6.5%)	0.006
Concomitant use or initiation of SGLT-2i	10,133 (17.2%)	6,928 (20.7%)	0.090	5,000 (20.2%)	4,977 (20.1%)	0.002
Concomitant use or initiation of Any other glucose-lowering drugs	3,268 (5.5%)	2,338 (7.0%)	0.060	1,660 (6.7%)	1,660 (6.7%)	0.000
Past use of Metformin	37,945 (64.4%)	22,648 (67.8%)	0.072	16,693 (67.3%)	16,709 (67.4%)	0.001
Past use of Insulins	13,583 (23.0%)	11,589 (34.7%)	0.259	7,694 (31.0%)	7,632 (30.8%)	0.005
Past use of Sulfonylureas	12,080 (20.5%)	10,386 (31.1%)	0.244	7,001 (28.2%)	6,868 (27.7%)	0.012
Past use of DPP-4i	4,981 (8.4%)	3,985 (11.9%)	0.115	2,645 (10.7%)	2,596 (10.5%)	0.006
Past use of SGLT-2i	14,104 (23.9%)	9,538 (28.5%)	0.105	6,921 (27.9%)	6,874 (27.7%)	0.004
Past use of Any other glucose-lowering drugs	4,578 (7.8%)	3,363 (10.1%)	0.081	2,376 (9.6%)	2,347 (9.5%)	0.004

**Other medications**						
ACE / ARB	42,276 (71.7%)	25,033 (74.9%)	0.072	18,460 (74.5%)	18,351 (74.0%)	0.010
ARNI	1,021 (1.7%)	605 (1.8%)	0.006	458 (1.8%)	445 (1.8%)	0.004
Thiazides	20,857 (35.4%)	11,075 (33.1%)	0.047	8,388 (33.8%)	8,418 (34.0%)	0.003
Beta-blockers	23,685 (40.2%)	14,764 (44.2%)	0.081	10,556 (42.6%)	10,590 (42.7%)	0.003
Calcium channel blockers	18,824 (31.9%)	11,543 (34.5%)	0.055	8,328 (33.6%)	8,310 (33.5%)	0.002
Digoxin / Digitoxin	300 (0.5%)	248 (0.7%)	0.030	140 (0.6%)	151 (0.6%)	0.006
Loop diuretics	10,407 (17.7%)	6,693 (20.0%)	0.061	4,743 (19.1%)	4,728 (19.1%)	0.002
Other diuretics	5,445 (9.2%)	3,042 (9.1%)	0.005	2,288 (9.2%)	2,269 (9.2%)	0.003
Intravenous diuretics	746 (1.3%)	545 (1.6%)	0.031	355 (1.4%)	365 (1.5%)	0.003
Nitrates	3,331 (5.7%)	2,469 (7.4%)	0.070	1,672 (6.7%)	1,680 (6.8%)	0.001
Anti-arrhythmics	1,351 (2.3%)	722 (2.2%)	0.009	520 (2.1%)	549 (2.2%)	0.008
Statins	45,331 (76.9%)	28,084 (84.0%)	0.181	20,314 (81.9%)	20,411 (82.3%)	0.010
PCSK9 inhibitors and other lipid-lowering drugs	8,255 (14.0%)	4,452 (13.3%)	0.020	3,481 (14.0%)	3,390 (13.7%)	0.011
Antiplatelet medications	5,279 (9.0%)	3,755 (11.2%)	0.076	2,603 (10.5%)	2,554 (10.3%)	0.006
Oral anticoagulants	5,812 (9.9%)	3,805 (11.4%)	0.050	2,617 (10.6%)	2,616 (10.6%)	0.000
COPD / Asthma medications	21,806 (37.0%)	11,741 (35.1%)	0.039	8,903 (35.9%)	8,831 (35.6%)	0.006
NSAIDS	19,911 (33.8%)	10,685 (32.0%)	0.038	8,068 (32.5%)	8,071 (32.6%)	0.000
Oral corticosteroids	16,517 (28.0%)	7,323 (21.9%)	0.141	5,865 (23.7%)	5,867 (23.7%)	0.000
Osteoporosis agents (incl. bisphosphonates)	1,380 (2.3%)	959 (2.9%)	0.033	648 (2.6%)	669 (2.7%)	0.005
Opioids	18,282 (31.0%)	10,208 (30.5%)	0.010	7,616 (30.7%)	7,594 (30.6%)	0.002
Anti-depressants	23,749 (40.3%)	13,271 (39.7%)	0.012	9,951 (40.1%)	9,810 (39.6%)	0.012
Antipsychotics	2,713 (4.6%)	1,863 (5.6%)	0.044	1,288 (5.2%)	1,235 (5.0%)	0.01
Anxiolytics / hypnotics, benzos	13,427 (22.8%)	6,681 (20.0%)	0.068	5,253 (21.2%)	5,163 (20.8%)	0.009
Dementia medications	632 (1.1%)	713 (2.1%)	0.085	401 (1.6%)	391 (1.6%)	0.003
Urinary tract infections antibiotics	28,224 (47.9%)	15,139 (45.3%)	0.052	11,447 (46.2%)	11,402 (46.0%)	0.004
Laxatives	2,046 (3.5%)	1,259 (3.8%)	0.016	893 (3.6%)	896 (3.6%)	0.001
**Healthcare utilization marker**						
Number of distinct medications; mean (SD) median [IQR]	14.48 ±7.21 13.00 [9.00, 18.00]	15.30 ±7.15 14.00 [10.00, 19.00]	0.114	15.07 ±7.17 14.00 [10.00, 19.00]	14.99 ±7.11 14.00 [10.00, 19.00]	0.012
Number of office visits; mean (SD) median [IQR]	9.67 ±7.19 8.00 [5.00, 13.00]	9.50 ±7.07 8.00 [5.00, 13.00]	0.024	9.55 ±6.86 8.00 [5.00, 13.00]	9.53 ±7.14 8.00 [5.00, 13.00]	0.004
Number of endocrinologist visits; mean (SD) median [IQR]	0.43 ±1.41 0.00 [0.00, 0.00]	0.49 ±1.59 0.00 [0.00, 0.00]	0.044	0.48 ±1.39 0.00 [0.00, 0.00]	0.47 ±1.54 0.00 [0.00, 0.00]	0.002
Number of cardiologist visits; mean (SD) median [IQR]	1.49 ±3.28 0.00 [0.00, 2.00]	1.59 ±3.61 0.00 [0.00, 2.00]	0.029	1.52 ±3.28 0.00 [0.00, 2.00]	1.53 ±3.45 0.00 [0.00, 2.00]	0.003
Number of internal / family medicine visits; mean (SD) median [IQR]	6.68 ±7.50 5.00 [3.00, 8.00]	7.98 ±9.82 5.00 [3.00, 9.00]	0.149	7.31 ±8.53 5.00 [3.00, 9.00]	7.26 ±8.42 5.00 [3.00, 9.00]	0.006
Number of electrocardiograms (ECG/EKG) ; mean (SD) median [IQR]	0.90 ±1.60 0.00 [0.00, 1.00]	0.98 ±1.73 0.00 [0.00, 1.00]	0.053	0.93 ±1.60 0.00 [0.00, 1.00]	0.93 ±1.64 0.00 [0.00, 1.00]	0.003
Number of echocardiograms; mean (SD) median [IQR]	0.26 ±0.62 0.00 [0.00, 0.00]	0.29 ±0.69 0.00 [0.00, 0.00]	0.042	0.28 ±0.63 0.00 [0.00, 0.00]	0.28 ±0.68 0.00 [0.00, 0.00]	0.003
Out-of-pocket medication cost; mean (SD) median [IQR]	581.50 ±786.55 330.72 [122.31, 769.78]	606.26 ±850.27 330.91 [115.80, 821.96]	0.030	603.55 ±740.32 348.00 [125.99, 823.83]	601.11 ±873.75 330.92 [118.32, 812.51]	0.003
Unique brand medicines; mean (SD) median [IQR]	14.71 ±7.42 13.00 [9.00, 19.00]	15.60 ±7.42 14.00 [10.00, 20.00]	0.121	15.35 ±7.41 14.00 [10.00, 19.00]	15.26 ±7.35 14.00 [10.00, 19.00]	0.012
Unique generic medicines; mean (SD) median [IQR]	14.48 ±7.21 13.00 [9.00, 18.00]	15.30 ±7.15 14.00 [10.00, 19.00]	0.114	15.07 ±7.17 14.00 [10.00, 19.00]	14.99 ±7.11 14.00 [10.00, 19.00]	0.012

Ratio of brand to generic medications; mean (SD) median [IQR]	1.01 ±0.04 1.00 [1.00, 1.00]	1.02 ±0.04 1.00 [1.00, 1.00]	0.105	1.02 ±0.04 1.00 [1.00, 1.00]	1.02 ±0.04 1.00 [1.00, 1.00]	0.004
**Healthy behavior markers**						
Colonoscopy / Sigmoidoscopy	6,385 (10.8%)	3,304 (9.9%)	0.031	2,533 (10.2%)	2,527 (10.2%)	0.001
Flu Pneumococcal vaccine	17,681 (30.0%)	11,686 (35.0%)	0.106	8,318 (33.5%)	8,300 (33.5%)	0.002
Pap smear	4,598 (7.8%)	1,813 (5.4%)	0.096	1,458 (5.9%)	1,500 (6.0%)	0.007
PSA test	13,445 (22.8%)	6,876 (20.6%)	0.054	5,311 (21.4%)	5,291 (21.3%)	0.002
Fecal occult blood test	2,181 (3.7%)	1,507 (4.5%)	0.041	1,006 (4.1%)	1,003 (4.0%)	0.001
Bone mineral density tests	4,345 (7.4%)	2,313 (6.9%)	0.017	1,707 (6.9%)	1,754 (7.1%)	0.007
Mammograms	16,016 (27.2%)	7,806 (23.4%)	0.088	6,121 (24.7%)	6,137 (24.8%)	0.001
Telemedicine	14,008 (23.8%)	7,678 (23.0%)	0.019	5,626 (22.7%)	5,582 (22.5%)	0.004
**Laboratory and diagnostic tests**						
HbA1c tests; mean (SD) median [IQR]	2.29 ±1.31 2.00 [1.00, 3.00]	2.47 ±1.38 2.00 [2.00, 3.00]	0.133	2.41 ±1.38 2.00 [2.00, 3.00]	2.41 ±1.32 2.00 [2.00, 3.00]	0.004
Lipid panels; mean (SD) median [IQR]	1.64 ±1.14 1.00 [1.00, 2.00]	1.57 ±1.15 1.00 [1.00, 2.00]	0.065	1.59 ±1.12 1.00 [1.00, 2.00]	1.60 ±1.16 1.00 [1.00, 2.00]	0.002
Creatinine tests; mean (SD) median [IQR]	2.76 ±2.80 2.00 [1.00, 4.00]	3.08 ±3.07 2.00 [1.00, 4.00]	0.110	2.94 ±3.06 2.00 [1.00, 4.00]	2.93 ±2.73 2.00 [1.00, 4.00]	0.003
Natriuretic peptide tests; mean (SD) median [IQR]	0.17 ±0.69 0.00 [0.00, 0.00]	0.20 ±0.83 0.00 [0.00, 0.00]	0.037	0.19 ±0.71 0.00 [0.00, 0.00]	0.18 ±0.72 0.00 [0.00, 0.00]	0.001
Urine tests; mean (SD) median [IQR]	1.03 ±1.62 0.00 [0.00, 1.00]	1.05 ±1.66 0.00 [0.00, 1.00]	0.012	1.03 ±1.61 0.00 [0.00, 1.00]	1.03 ±1.66 0.00 [0.00, 1.00]	0.000
**Lab values**						
HbA1c (%)*; mean (SD) median [IQR]	7.59 ±1.71 7.10 [6.40, 8.40]	8.23 ±1.84 7.90 [6.90, 9.20]	0.361	7.81 ±1.77 7.40 [6.50, 8.70]	8.13 ±1.83 7.80 [6.80, 9.10]	0.178
Glucose (mg/dl)*; mean (SD) median [IQR]	154.81 ±66.88 137.00 [111.00, 179.00]	709.96 ±73,034.26 158.00 [124.00, 210.00]	0.011	161.44 ±70.21 143.50 [114.00, 189.00]	876.61 ±83,854.72 155.00 [121.00, 206.00]	0.012
Creatinine (mg/dl)*; mean (SD) median [IQR]	1.01 ±1.32 0.89 [0.80, 1.08]	1.06 ±1.07 0.92 [0.80, 1.15]	0.043	1.05 ±1.94 0.91 [0.80, 1.12]	1.04 ±1.14 0.91 [0.80, 1.12]	0.006
Systolic blood pressure (mmHg)*; mean (SD) median [IQR]	129.38 ±22.89 130.00 [120.00, 139.00]	130.47 ±26.86 130.00 [120.00, 140.00]	0.043	129.90 ±22.63 130.00 [120.00, 140.00]	130.13 ±27.13 130.00 [120.00, 140.00]	0.009
Heart rate (1/min)*; mean (SD) median [IQR]	78.57 ±15.04 78.00 [69.00, 87.00]	78.76 ±17.40 78.00 [69.00, 87.00]	0.012	78.37 ±14.56 77.00 [69.00, 87.00]	78.81 ±18.29 78.00 [69.00, 87.00]	0.027
BMI (kg/m2)*; mean (SD) median [IQR]	38.70 ±8.05 37.40 [33.05, 42.77]	36.44 ±7.54 35.15 [31.07, 40.30]	0.290	37.78 ±7.93 36.42 [32.12, 41.66]	36.88 ±7.60 35.60 [31.46, 40.81]	0.116
eGFR (ml/min/1.73m2)*; mean (SD) median [IQR]	2,420.68 ±152,973.25 83.00 [64.00, 98.00]	1,525.25 ±120,389.29 78.00 [58.00, 95.00]	0.007	2,618.84 ±159,419.94 79.00 [60.00, 95.00]	1,886.79 ±134,527.43 79.00 [60.00, 96.00]	0.005
LDL (mg/dl)*; mean (SD) median [IQR]	87.31 ±39.64 83.00 [62.00, 110.00]	84.02 ±39.58 79.00 [59.00, 105.00]	0.083	85.07 ±39.55 81.00 [60.00, 107.00]	84.53 ±39.87 80.00 [59.00, 106.00]	0.013
HDL (mg/dl)*; mean (SD) median [IQR]	46.04 ±13.91 45.00 [37.00, 53.00]	45.02 ±13.75 43.00 [36.00, 53.00]	0.074	45.76 ±13.91 44.00 [37.00, 53.00]	45.04 ±13.77 44.00 [36.00, 52.00]	0.052
Total cholesterol (mg/dl)*; mean (SD) median [IQR]	165.47 ±49.63 161.00 [135.00, 192.00]	162.43 ±50.77 157.00 [131.00, 189.00]	0.061	162.99 ±50.08 158.00 [133.00, 189.00]	163.02 ±51.02 157.00 [131.00, 190.00]	0.001
Triglyceride (mg/dl)*; mean (SD) median [IQR]	175.63 ±149.62 144.00 [104.00, 205.00]	181.79 ±168.10 148.00 [106.00, 210.00]	0.039	176.96 ±151.29 144.00 [103.00, 207.00]	182.12 ±166.70 148.00 [105.00, 210.00]	0.032
**Burden of comorbidities**						
Combined comorbidity score; mean (SD) median [IQR]	1.80 ±2.28 1.00 [0.00, 3.00]	2.30 ±2.60 2.00 [0.00, 4.00]	0.204	2.09 ±2.46 1.00 [0.00, 3.00]	2.07 ±2.41 1.00 [0.00, 3.00]	0.009
Frailty Score; mean (SD) median [IQR]	0.16 ±0.05 0.15 [0.13, 0.19]	0.17 ±0.06 0.16 [0.13, 0.20]	0.167	0.17 ±0.06 0.16 [0.13, 0.20]	0.17 ±0.06 0.16 [0.13, 0.20]	0.005
**Baseline hospitalizations and hospital metrics**						

Number of Hospitalizations; mean (SD) median [IQR]	0.16 ±0.58 0.00 [0.00, 0.00]	0.27 ±0.84 0.00 [0.00, 0.00]	0.142	0.21 ±0.69 0.00 [0.00, 0.00]	0.21 ±0.68 0.00 [0.00, 0.00]	0.005
Any hospitalization within prior 91 days	2,078 (3.5%)	1,980 (5.9%)	0.113	1,155 (4.7%)	1,140 (4.6%)	0.003
Any hospitalization within prior 92-365 days	5,169 (8.8%)	3,936 (11.8%)	0.099	2,533 (10.2%)	2,517 (10.2%)	0.002
Number of hospitalizations (0, 1, 2 or more)						
< 1	52,488 (89.0%)	28,313 (84.7%)	0.128	21,564 (87.0%)	21,587 (87.1%)	0.003
1 - <2	4,588 (7.8%)	3,216 (9.6%)	0.065	2,159 (8.7%)	2,162 (8.7%)	0.000
>= 2	1,879 (3.2%)	1,891 (5.7%)	0.120	1,072 (4.3%)	1,046 (4.2%)	0.005
Heart failure hospitalization	1,714 (2.9%)	1,492 (4.5%)	0.083	919 (3.7%)	924 (3.7%)	0.001
ED visit	15,564 (26.4%)	10,963 (32.8%)	0.141	7,599 (30.6%)	7,457 (30.1%)	0.012
**Calendar year of cohort entry**						
2022	5,070 (8.6%)	11,086 (33.2%)	0.634	4,682 (18.9%)	4,704 (19.0%)	0.002
2023	23,677 (40.2%)	14,561 (43.6%)	0.069	12,500 (50.4%)	12,508 (50.4%)	0.001
2024	28,830 (48.9%)	7,632 (22.8%)	0.565	7,473 (30.1%)	7,442 (30.0%)	0.003
2025	1,378 (2.3%)	141 (0.4%)	0.165	140 (0.6%)	141 (0.6%)	0.001

Abbreviations: *not used in the propensity score; ACE, angiotensin-converting enzyme inhibitors; ARB, angiotensin receptor blocker; ARNI, angiotensin receptor/neprilysin inhibitor; BMI, body mass index; CABG, coronary artery bypass graft surgery; CED, cohort entry date; CKD, chronic kidney disease; COPD, chronic obstructive pulmonary disease; DKA, diabetic ketoacidosis; DPP4i, dipeptidyl peptidase-4 inhibitors; ED, emergency department; eGFR, estimated glomerular filtration rate (estimated using the quadratic GFR equation: GFR = EXP(1.911 + (5.249 / Serum creatinine) - (2.114 / (Serum creatinine^2)) - (0.00686 * Age) - 0.205 (if female))); HbA1c, hemoglobin A1c; HDL, high-density lipoprotein cholesterol; HONK, hyperglycemic hyperosmolar nonketotic state; IQR, inter-quartile-range; MASH, metabolic dysfunction associated steatohepatitis; MASLD, metabolic dysfunction associated steatotic liver disease; MI, myocardial infarction; N, number of participants; NSAIDs, non-steroidal anti-inflammatory drugs; PCSK9, proprotein convertase subtilisin/kexin type 9; PSA, prostate-specific antigen; PTCA, percutaneous transluminal coronary angioplasty; SGLT2i, sodium-glucose transport protein 2 inhibitors; SD, standard deviation; SMD, standardized mean difference; TIA, transient ischemic attack.
Missing data were handled by assuming absence of a code indicated absence of the condition for most binary covariates. Missing indicators were included for race and region in the propensity score model.
Laboratory values were only available in a subset of the Optum database and were truncated using clinically plausible cut-off values (BMI values >100 or <10 were set to missing; missingness before matching ~54%. Creatinine values >30 were set to missing, and values <0.8 were set to 0.8; missingness before matching ~41%. eGFR values >150 were set to 150, and values =0 were set to missing; missingness before matching ~53%. Glucose values <30 were set to missing; missingness before matching ~41%. HbA1c values ≥20 or ≤2 were set to missing; missingness before matching ~36%. HDL value missingness before matching was ~48%. Heart rate values <30 were set to missing; missingness before matching ~67%. LDL value missingness before matching was~46%. Systolic blood pressure values <30 were set to missing; missingness before matching ~51%. Total cholesterol value missingness before matching was ~48%. Triglyceride value missingness before matching was ~49%).
The Race-Others category includes Asian and Hispanic individuals.

Supplementary Table 15. Baseline characteristics of initiators of tirzepatide vs dulaglutide when applying expanded eligibility criteria before and after propensity score matching. Marketscan database. Values are number (percentage) unless otherwise specified.

	Before propensity score matching			After propensity score matching		
Variable	Tirzepatide (n = 23,787)	Dulaglutide (n = 19,927)	SMD	Tirzepatide (n = 14,357)	Dulaglutide (n = 14,357)	SMD
**Demographics**						
Age	53.84 ±10.02 54.00 [48.00, 60.00]	56.41 ±11.05 57.00 [49.00, 63.00]	0.244	55.04 ±10.15 55.00 [49.00, 61.00]	55.00 ±10.54 55.00 [48.00, 62.00]	0.003
Gender						
Male	10,568 (44.4%)	9,695 (48.7%)	0.085	6,745 (47.0%)	6,711 (46.7%)	0.005
Female	13,219 (55,6%)	10,232 (51,3%)	0.085	7,612 (53,0%)	7,646 (53,3%)	0.005
Region / State						
Northeast	2,065 (8.7%)	2,386 (12.0%)	0.108	1,499 (10.4%)	1,485 (10.3%)	0.003
Midwest / North central	5,247 (22.1%)	6,172 (31.0%)	0.203	3,807 (26.5%)	3,881 (27.0%)	0.012
South	14,692 (61.8%)	9,614 (48.2%)	0.274	7,838 (54.6%)	7,760 (54.1%)	0.011
West	1,765 (7.4%)	1,740 (8.7%)	0.048	1,201 (8.4%)	1,222 (8.5%)	0.005
Missing	18 (0.1%)	15 (0.1%)	0.000	12 (0.1%)	9 (0.1%)	0.008
**Lifestyle risk factors**						
Smoking / Tobacco use	2,227 (9.4%)	2,192 (11.0%)	0.054	1,459 (10.2%)	1,462 (10.2%)	0.001
Weight						
Overweight (25.0-29.9)	1,845 (7.8%)	2,417 (12.1%)	0.147	1,393 (9.7%)	1,388 (9.7%)	0.001
Class 1 Obesity (30.0-34.9)	1,862 (7.8%)	1,925 (9.7%)	0.065	1,235 (8.6%)	1,237 (8.6%)	0.000
Class 2 Obesity (35.0-39.9)	1,634 (6.9%)	1,322 (6.6%)	0.009	1,003 (7.0%)	991 (6.9%)	0.003
Class 3 Obesity (40.0 and above)	5,167 (21.7%)	5,184 (26.0%)	0.101	3,414 (23.8%)	3,453 (24.1%)	0.006
Unspecified Obesity	13,279 (55.8%)	9,079 (45.6%)	0.206	7,312 (50.9%)	7,288 (50.8%)	0.003
**Diabetes complications**						
Diabetic retinopathy	1,229 (5.2%)	1,551 (7.8%)	0.106	934 (6.5%)	929 (6.5%)	0.001
Diabetic neuropathy	3,133 (13.2%)	3,742 (18.8%)	0.154	2,316 (16.1%)	2,248 (15.7%)	0.013
Diabetic nephropathy	2,385 (10.0%)	2,932 (14.7%)	0.143	1,754 (12.2%)	1,711 (11.9%)	0.009
Diabetes with other opthalmic complications	294 (1.2%)	331 (1.7%)	0.036	221 (1.5%)	214 (1.5%)	0.004
Diabetes with peripheral circulatory disorders	1,643 (6.9%)	1,726 (8.7%)	0.066	1,120 (7.8%)	1,072 (7.5%)	0.013
Diabetic foot	313 (1.3%)	394 (2.0%)	0.052	231 (1.6%)	226 (1.6%)	0.003
Erectile dysfunction	798 (3.4%)	742 (3.7%)	0.020	540 (3.8%)	539 (3.8%)	0.000
Hypoglycemia	4,310 (18.1%)	3,641 (18.3%)	0.004	2,623 (18.3%)	2,587 (18.0%)	0.007
Hyperglycemia / DKA / HONK	11,497 (48.3%)	10,889 (54.6%)	0.127	7,496 (52.2%)	7,460 (52.0%)	0.005
Skin infections	2,027 (8.5%)	1,889 (9.5%)	0.033	1,312 (9.1%)	1,264 (8.8%)	0.012
**Cardiovascular-related conditions**						
Coronary atherosclerosis	2,320 (9.8%)	2,522 (12.7%)	0.092	1,592 (11.1%)	1,552 (10.8%)	0.009
Stable angina	425 (1.8%)	506 (2.5%)	0.052	306 (2.1%)	291 (2.0%)	0.007

Unstable angina	271 (1.1%)	343 (1.7%)	0.049	197 (1.4%)	193 (1.3%)	0.002
Hypertension	19,082 (80.2%)	16,057 (80.6%)	0.009	11,494 (80.1%)	11,463 (79.8%)	0.005
Hypotension	252 (1.1%)	315 (1.6%)	0.046	170 (1.2%)	165 (1.1%)	0.003
Hyperlipidemia	18,587 (78.1%)	15,727 (78.9%)	0.019	11,319 (78.8%)	11,299 (78.7%)	0.003
Acute MI	164 (0.7%)	232 (1.2%)	0.050	118 (0.8%)	134 (0.9%)	0.012
Old MI	254 (1.1%)	307 (1.5%)	0.042	180 (1.3%)	177 (1.2%)	0.002
Ischemic stroke	124 (0.5%)	200 (1.0%)	0.055	104 (0.7%)	97 (0.7%)	0.006
TIA	211 (0.9%)	232 (1.2%)	0.028	140 (1.0%)	137 (1.0%)	0.002
Cardiac conduction disorder	572 (2.4%)	678 (3.4%)	0.059	401 (2.8%)	388 (2.7%)	0.006
Previous cardiac procedure (CABG, PTCA, Stent)	210 (0.9%)	268 (1.3%)	0.044	152 (1.1%)	159 (1.1%)	0.005
PVD diagnosis or surgery	781 (3.3%)	916 (4.6%)	0.068	550 (3.8%)	526 (3.7%)	0.009
Atrial fibrillation	1,059 (4.5%)	1,181 (5.9%)	0.067	724 (5.0%)	724 (5.0%)	0.000
Other cardiac dysrhythmia	3,151 (13.2%)	2,854 (14.3%)	0.031	1,925 (13.4%)	1,889 (13.2%)	0.007
Heart failure	1,123 (4.7%)	1,357 (6.8%)	0.090	790 (5.5%)	746 (5.2%)	0.014
Acute heart failure	239 (1.0%)	362 (1.8%)	0.069	182 (1.3%)	174 (1.2%)	0.005
Cardiomyopathy	571 (2.4%)	628 (3.2%)	0.046	379 (2.6%)	379 (2.6%)	0.000
Valve disorders	1,229 (5.2%)	1,242 (6.2%)	0.046	814 (5.7%)	793 (5.5%)	0.006
Valve replacement	82 (0.3%)	113 (0.6%)	0.033	60 (0.4%)	61 (0.4%)	0.001
Edema	1,669 (7.0%)	1,531 (7.7%)	0.026	1,020 (7.1%)	983 (6.8%)	0.01
Venous thromboembolism / Pulmonary embolism	483 (2.0%)	488 (2.4%)	0.028	327 (2.3%)	322 (2.2%)	0.002
Pulmonary hypertension	226 (1.0%)	232 (1.2%)	0.021	143 (1.0%)	149 (1.0%)	0.004
Implantable cardioverter defibrillator	32 (0.1%)	45 (0.2%)	0.022	24 (0.2%)	20 (0.1%)	0.007
Hyperkalemia	236 (1.0%)	330 (1.7%)	0.058	178 (1.2%)	158 (1.1%)	0.013
Cerebrovascular procedure	9 (0.0%)	14 (0.1%)	0.014	8 (0.1%)	6 (0.0%)	0.006
Insertion of pacemakers / removal of cardiac lead	7 (0.0%)	15 (0.1%)	0.020	6 (0.0%)	7 (0.0%)	0.003
**Renal-related conditions**						
Hypertensive nephropathy	984 (4.1%)	1,215 (6.1%)	0.089	701 (4.9%)	678 (4.7%)	0.007
CKD Stage 1-2	485 (2.0%)	411 (2.1%)	0.002	292 (2.0%)	288 (2.0%)	0.002
CKD Stage 3-4	1,006 (4.2%)	1,440 (7.2%)	0.129	764 (5.3%)	738 (5.1%)	0.008
Unspecified CKD	352 (1.5%)	465 (2.3%)	0.062	263 (1.8%)	249 (1.7%)	0.007
Microalbuminuria or proteinuria	752 (3.2%)	841 (4.2%)	0.056	540 (3.8%)	524 (3.6%)	0.006
Acute kidney injury	575 (2.4%)	781 (3.9%)	0.086	420 (2.9%)	395 (2.8%)	0.010
Urinary tract infections	2,209 (9.3%)	1,775 (8.9%)	0.013	1,228 (8.6%)	1,254 (8.7%)	0.006
Genital infections	868 (3.6%)	818 (4.1%)	0.024	570 (4.0%)	560 (3.9%)	0.004
Urolithiasis (Kidney and urinary stone)	934 (3.9%)	802 (4.0%)	0.005	557 (3.9%)	568 (4.0%)	0.004
**Other comorbidities**						
COPD	885 (3.7%)	993 (5.0%)	0.062	605 (4.2%)	578 (4.0%)	0.009
Asthma	2,194 (9.2%)	1,730 (8.7%)	0.019	1,291 (9.0%)	1,262 (8.8%)	0.007
Obstructive sleep apnea	7,089 (29.8%)	5,350 (26.8%)	0.066	4,021 (28.0%)	3,983 (27.7%)	0.006

Serious bacterial infections	505 (2.1%)	617 (3.1%)	0.061	370 (2.6%)	354 (2.5%)	0.007
Pneumonia	626 (2.6%)	683 (3.4%)	0.046	411 (2.9%)	398 (2.8%)	0.005
Liver disease	3,297 (13.9%)	2,583 (13.0%)	0.026	1,883 (13.1%)	1,875 (13.1%)	0.002
MASH / MASLD	2,216 (9.3%)	1,583 (7.9%)	0.049	1,219 (8.5%)	1,212 (8.4%)	0.002
Fractures / Falls	687 (2.9%)	743 (3.7%)	0.047	461 (3.2%)	459 (3.2%)	0.001
Osteoporosis	273 (1.1%)	273 (1.4%)	0.02	180 (1.3%)	186 (1.3%)	0.004
Osteoarthritis	4,531 (19.0%)	3,860 (19.4%)	0.008	2,719 (18.9%)	2,725 (19.0%)	0.001
Depression	3,706 (15.6%)	3,114 (15.6%)	0.001	2,236 (15.6%)	2,226 (15.5%)	0.002
Dementia	147 (0.6%)	311 (1.6%)	0.091	119 (0.8%)	116 (0.8%)	0.002
Delirium or psychosis	85 (0.4%)	140 (0.7%)	0.048	73 (0.5%)	67 (0.5%)	0.006
Anxiety	4,986 (21.0%)	3,610 (18.1%)	0.072	2,770 (19.3%)	2,708 (18.9%)	0.011
Sleep disorders	5,954 (25.0%)	4,422 (22.2%)	0.067	3,328 (23.2%)	3,346 (23.3%)	0.003
Anemia	3,294 (13.8%)	2,717 (13.6%)	0.006	1,931 (13.4%)	1,872 (13.0%)	0.012
Influenza	602 (2.5%)	295 (1.5%)	0.075	272 (1.9%)	254 (1.8%)	0.009
COVID	3,133 (13.2%)	2,752 (13.8%)	0.019	1,893 (13.2%)	1,920 (13.4%)	0.006
Hyperthyroidism and other thyroid gland disorders	5,303 (22.3%)	3,904 (19.6%)	0.066	2,902 (20.2%)	2,881 (20.1%)	0.004
Hypothyroidism	4,153 (17.5%)	3,023 (15.2%)	0.062	2,282 (15.9%)	2,246 (15.6%)	0.007
Nephrotic syndrome	8 (0.0%)	15 (0.1%)	0.018	7 (0.0%)	6 (0.0%)	0.003
Urinary incontinence	605 (2.5%)	539 (2.7%)	0.010	388 (2.7%)	373 (2.6%)	0.007
Biliary disease	57 (0.2%)	63 (0.3%)	0.015	38 (0.3%)	34 (0.2%)	0.006
Pancreatitis	28 (0.1%)	27 (0.1%)	0.005	18 (0.1%)	14 (0.1%)	0.008
Bowel obstruction	31 (0.1%)	28 (0.1%)	0.003	21 (0.1%)	19 (0.1%)	0.004
Gastroparesis	128 (0.5%)	116 (0.6%)	0.006	76 (0.5%)	81 (0.6%)	0.005
Diabetes medications						
Number of antidiabetic drugs on CED, mean (SD)	2.05 ±0.98 2.00 [1.00, 3.00]	2.26 ±1.01 2.00 [2.00, 3.00]	0.203	2.18 ±1.02 2.00 [1.00, 3.00]	2.18 ±0.98 2.00 [1.00, 3.00]	0.003
Concomitant use or initiation of Metformin	11,961 (50.3%)	10,664 (53.5%)	0.065	7,603 (53.0%)	7,618 (53.1%)	0.002
Concomitant use or initiation of Insulins	3,213 (13.5%)	3,820 (19.2%)	0.154	2,388 (16.6%)	2,364 (16.5%)	0.004
Concomitant use or initiation of Sulfonylureas	2,591 (10.9%)	3,509 (17.6%)	0.193	2,043 (14.2%)	2,057 (14.3%)	0.003
Concomitant use or initiation of DPP-4i	1,558 (6.5%)	1,667 (8.4%)	0.069	1,083 (7.5%)	1,088 (7.6%)	0.001
Concomitant use or initiation of SGLT-2i	4,799 (20.2%)	4,397 (22.1%)	0.046	3,143 (21.9%)	3,184 (22.2%)	0.007
Concomitant use or initiation of Any other glucose-lowering drugs	925 (3.9%)	953 (4.8%)	0.044	656 (4.6%)	647 (4.5%)	0.003
Past use of Metformin	16,220 (68.2%)	14,310 (71.8%)	0.079	10,176 (70.9%)	10,181 (70.9%)	0.001
Past use of Insulins	4,504 (18.9%)	5,321 (26.7%)	0.186	3,317 (23.1%)	3,264 (22.7%)	0.009
Past use of Sulfonylureas	3,870 (16.3%)	5,000 (25.1%)	0.219	2,989 (20.8%)	2,975 (20.7%)	0.002
Past use of DPP-4i	2,489 (10.5%)	2,711 (13.6%)	0.097	1,746 (12.2%)	1,751 (12.2%)	0.001
Past use of SGLT-2i	6,315 (26.5%)	5,884 (29.5%)	0.066	4,140 (28.8%)	4,175 (29.1%)	0.005
Past use of Any other glucose-lowering drugs	1,334 (5.6%)	1,402 (7.0%)	0.059	938 (6.5%)	921 (6.4%)	0.005
Other medications						
ACE / ARB	16,441 (69.1%)	14,316 (71.8%)	0.060	10,123 (70.5%)	10,121 (70.5%)	0.000

ARNI	272 (1.1%)	260 (1.3%)	0.015	169 (1.2%)	179 (1.2%)	0.006
Thiazides	8,364 (35.2%)	6,587 (33.1%)	0.044	4,832 (33.7%)	4,843 (33.7%)	0.002
Beta-blockers	7,500 (31.5%)	6,831 (34.3%)	0.059	4,644 (32.3%)	4,592 (32.0%)	0.008
Calcium channel blockers	6,445 (27.1%)	5,750 (28.9%)	0.039	3,993 (27.8%)	3,976 (27.7%)	0.003
Digoxin / Digitoxin	70 (0.3%)	83 (0.4%)	0.021	44 (0.3%)	49 (0.3%)	0.006
Loop diuretics	2,169 (9.1%)	2,134 (10.7%)	0.053	1,404 (9.8%)	1,312 (9.1%)	0.022
Other diuretics	1,773 (7.5%)	1,505 (7.6%)	0.004	1,059 (7.4%)	1,073 (7.5%)	0.004
Intravenous diuretics	113 (0.5%)	153 (0.8%)	0.037	82 (0.6%)	81 (0.6%)	0.001
Nitrates	727 (3.1%)	893 (4.5%)	0.075	527 (3.7%)	522 (3.6%)	0.002
Anti-arrhythmics	311 (1.3%)	284 (1.4%)	0.010	177 (1.2%)	197 (1.4%)	0.012
Statins	16,717 (70.3%)	15,366 (77.1%)	0.156	10,702 (74.5%)	10,698 (74.5%)	0.001
PCSK9 inhibitors and other lipid-lowering drugs	2,820 (11.9%)	2,478 (12.4%)	0.018	1,781 (12.4%)	1,764 (12.3%)	0.004
Antiplatelet medications	1,673 (7.0%)	1,882 (9.4%)	0.088	1,161 (8.1%)	1,169 (8.1%)	0.002
Oral anticoagulants	1,390 (5.8%)	1,472 (7.4%)	0.062	921 (6.4%)	924 (6.4%)	0.001
COPD / Asthma medications	7,946 (33.4%)	6,107 (30.6%)	0.059	4,586 (31.9%)	4,513 (31.4%)	0.011
NSAIDS	8,330 (35.0%)	6,295 (31.6%)	0.073	4,750 (33.1%)	4,731 (33.0%)	0.003
Oral corticosteroids	6,781 (28.5%)	4,754 (23.9%)	0.106	3,629 (25.3%)	3,637 (25.3%)	0.001
Osteoporosis agents (incl. bisphosphonates)	299 (1.3%)	309 (1.6%)	0.025	208 (1.4%)	202 (1.4%)	0.004
Opioids	6,002 (25.2%)	4,830 (24.2%)	0.023	3,516 (24.5%)	3,494 (24.3%)	0.004
Anti-depressants	8,781 (36.9%)	6,782 (34.0%)	0.060	5,111 (35.6%)	5,030 (35.0%)	0.012
Antipsychotics	702 (3.0%)	576 (2.9%)	0.004	419 (2.9%)	407 (2.8%)	0.005
Anxiolytics / hypnotics, benzos	5,012 (21.1%)	3,477 (17.4%)	0.092	2,723 (19.0%)	2,679 (18.7%)	0.008
Dementia medications	55 (0.2%)	153 (0.8%)	0.076	49 (0.3%)	44 (0.3%)	0.006
Urinary tract infections antibiotics	11,083 (46.6%)	8,510 (42.7%)	0.078	6,299 (43.9%)	6,332 (44.1%)	0.005
Laxatives	706 (3.0%)	527 (2.6%)	0.020	385 (2.7%)	396 (2.8%)	0.005
Healthcare utilization marker						
Number of distinct medications; mean (SD) median [IQR]	13.38 ±6.58 12.00 [9.00, 17.00]	13.21 ±6.31 12.00 [9.00, 17.00]	0.026	13.23 ±6.39 12.00 [9.00, 17.00]	13.13 ±6.41 12.00 [9.00, 16.00]	0.015
Number of office visits; mean (SD) median [IQR]	8.32 ±6.21 7.00 [4.00, 11.00]	8.15 ±6.16 7.00 [4.00, 11.00]	0.027	8.10 ±5.93 7.00 [4.00, 11.00]	8.05 ±6.14 6.00 [4.00, 10.00]	0.009
Number of endocrinologist visits; mean (SD) median [IQR]	0.38 ±1.19 0.00 [0.00, 0.00]	0.39 ±1.24 0.00 [0.00, 0.00]	0.011	0.38 ±1.16 0.00 [0.00, 0.00]	0.38 ±1.21 0.00 [0.00, 0.00]	0.006
Number of cardiologist visits; mean (SD) median [IQR]	0.83 ±2.21 0.00 [0.00, 1.00]	1.01 ±2.72 0.00 [0.00, 1.00]	0.071	0.89 ±2.27 0.00 [0.00, 1.00]	0.89 ±2.53 0.00 [0.00, 1.00]	0.001
Number of internal / family medicine visits; mean (SD) median [IQR]	4.55 ±5.48 3.00 [1.00, 6.00]	4.94 ±5.87 4.00 [2.00, 6.00]	0.069	4.68 ±5.96 3.00 [1.00, 6.00]	4.67 ±5.45 3.00 [1.00, 6.00]	0.002
Number of electrocardiograms (ECG/EKG) ; mean (SD) median [IQR]	0.66 ±1.24 0.00 [0.00, 1.00]	0.76 ±1.46 0.00 [0.00, 1.00]	0.070	0.69 ±1.30 0.00 [0.00, 1.00]	0.68 ±1.35 0.00 [0.00, 1.00]	0.009
Number of echocardiograms; mean (SD) median [IQR]	0.19 ±0.56 0.00 [0.00, 0.00]	0.23 ±0.67 0.00 [0.00, 0.00]	0.065	0.20 ±0.58 0.00 [0.00, 0.00]	0.20 ±0.62 0.00 [0.00, 0.00]	0.004
Out-of-pocket medication cost; mean (SD) median [IQR]	539.23 ±1,028.23 296.03 [110.00, 670.00]	525.68 ±813.50 318.00 [124.20, 672.49]	0.015	534.96 ±853.54 298.20 [112.09, 673.22]	528.54 ±827.61 319.72 [119.28, 671.62]	0.008
Unique brand medicines; mean (SD) median [IQR]	13.60 ±6.75 12.00 [9.00, 17.00]	13.44 ±6.49 12.00 [9.00, 17.00]	0.025	13.44 ±6.57 12.00 [9.00, 17.00]	13.34 ±6.58 12.00 [9.00, 17.00]	0.015
Unique generic medicines; mean (SD) median [IQR]	13.38 ±6.58 12.00 [9.00, 17.00]	13.21 ±6.31 12.00 [9.00, 17.00]	0.026	13.23 ±6.39 12.00 [9.00, 17.00]	13.13 ±6.41 12.00 [9.00, 16.00]	0.015

Ratio of brand to generic medications; mean (SD) median [IQR]	1.01 ±0.04 1.00 [1.00, 1.00]	1.02 ±0.04 1.00 [1.00, 1.00]	0.018	1.01 ±0.04 1.00 [1.00, 1.00]	1.01 ±0.04 1.00 [1.00, 1.00]	0.002
**Healthy behavior markers**						
Colonoscopy / Sigmoidoscopy	2,592 (10.9%)	1,996 (10.0%)	0.029	1,501 (10.5%)	1,520 (10.6%)	0.004
Flu Pneumococcal vaccine	6,164 (25.9%)	6,142 (30.8%)	0.109	4,081 (28.4%)	4,110 (28.6%)	0.004
Pap smear	3,024 (12.7%)	1,911 (9.6%)	0.099	1,558 (10.9%)	1,561 (10.9%)	0.001
PSA test	5,004 (21.0%)	4,103 (20.6%)	0.011	3,012 (21.0%)	2,974 (20.7%)	0.007
Fecal occult blood test	818 (3.4%)	639 (3.2%)	0.013	488 (3.4%)	466 (3.2%)	0.009
Bone mineral density tests	814 (3.4%)	616 (3.1%)	0.019	411 (2.9%)	472 (3.3%)	0.025
Mammograms	6,520 (27.4%)	4,755 (23.9%)	0.081	3,625 (25.2%)	3,693 (25.7%)	0.011
Telemedicine	6,961 (29.3%)	5,769 (29.0%)	0.007	4,117 (28.7%)	4,137 (28.8%)	0.003
**Laboratory and diagnostic tests**						
HbA1c tests; mean (SD) median [IQR]	2.16 ±1.42 2.00 [1.00, 3.00]	2.21 ±1.44 2.00 [1.00, 3.00]	0.033	2.19 ±1.54 2.00 [1.00, 3.00]	2.18 ±1.41 2.00 [1.00, 3.00]	0.004
Lipid panels; mean (SD) median [IQR]	1.58 ±1.18 1.00 [1.00, 2.00]	1.48 ±1.48 1.00 [1.00, 2.00]	0.071	1.53 ±1.20 1.00 [1.00, 2.00]	1.51 ±1.21 1.00 [1.00, 2.00]	0.017
Creatinine tests; mean (SD) median [IQR]	0.03 ±0.25 0.00 [0.00, 0.00]	0.04 ±0.34 0.00 [0.00, 0.00]	0.033	0.03 ±0.28 0.00 [0.00, 0.00]	0.03 ±0.23 0.00 [0.00, 0.00]	0.021
Natriuretic peptide tests; mean (SD) median [IQR]	0.10 ±0.61 0.00 [0.00, 0.00]	0.12 ±0.91 0.00 [0.00, 0.00]	0.036	0.11 ±0.67 0.00 [0.00, 0.00]	0.10 ±0.84 0.00 [0.00, 0.00]	0.005
Urine tests; mean (SD) median [IQR]	0.89 ±1.58 0.00 [0.00, 1.00]	0.85 ±1.76 0.00 [0.00, 1.00]	0.023	0.86 ±1.58 0.00 [0.00, 1.00]	0.86 ±1.83 0.00 [0.00, 1.00]	0.002
**Burden of comorbidities**						
Combined comorbidity score; mean (SD) median [IQR]	0.93 ±1.58 1.00 [0.00, 2.00]	1.17 ±1.81 1.00 [0.00, 2.00]	0.143	1.03 ±1.66 1.00 [0.00, 2.00]	1.00 ±1.64 1.00 [0.00, 2.00]	0.018
Frailty Score; mean (SD) median [IQR]	0.14 ±0.04 0.13 [0.12, 0.16]	0.15 ±0.04 0.14 [0.12, 0.16]	0.123	0.14 ±0.04 0.14 [0.12, 0.16]	0.14 ±0.04 0.14 [0.12, 0.16]	0.019
**Baseline hospitalizations and hospital metrics**						
Number of Hospitalizations; mean (SD) median [IQR]	3.83 ±20.05 0.00 [0.00, 0.00]	5.80 ±26.20 0.00 [0.00, 0.00]	0.085	4.58 ±22.39 0.00 [0.00, 0.00]	4.41 ±23.08 0.00 [0.00, 0.00]	0.007
Any hospitalization within prior 91 days	560 (2.4%)	717 (3.6%)	0.073	400 (2.8%)	399 (2.8%)	0.000
Any hospitalization within prior 92-365 days	1,239 (5.2%)	1,444 (7.2%)	0.084	868 (6.0%)	838 (5.8%)	0.009
Number of hospitalizations (0, 1, 2 or more)						
< 1	22,133 (93.0%)	17,955 (90.1%)	0.106	13,195 (91.9%)	13,211 (92.0%)	0.004
1 - <2	125 (0.5%)	124 (0.6%)	0.013	75 (0.5%)	77 (0.5%)	0.002
>= 2	1,529 (6.4%)	1,848 (9.3%)	0.106	1,087 (7.6%)	1,069 (7.4%)	0.005
Heart failure hospitalization	307 (1.3%)	469 (2.4%)	0.080	235 (1.6%)	229 (1.6%)	0.003
ED visit	6,542 (27.5%)	6,201 (31.1%)	0.080	4,139 (28.8%)	4,113 (28.6%)	0.004
**Calendar year of cohort entry**						
2022	4,332 (18.2%)	8,893 (44.6%)	0.594	4,181 (29.1%)	4,180 (29.1%)	0.000
2023	19,455 (81.8%)	11,034 (55.4%)	0.594	10,176 (70.9%)	10,177 (70.9%)	0.000

Abbreviations: *not used in the propensity score; ACE, angiotensin-converting enzyme inhibitors; ARB, angiotensin receptor blocker; ARNI, angiotensin receptor/neprilysin inhibitor; BMI, body mass index; CABG, coronary artery bypass graft surgery; CED, cohort entry date; CKD, chronic kidney disease; COPD, chronic obstructive pulmonary disease; DKA, diabetic ketoacidosis; DPP4i, dipeptidyl peptidase-4 inhibitors; ED, emergency department; eGFR, estimated glomerular filtration rate (estimated using the quadratic GFR equation: GFR = EXP(1.911 + (5.249 / Serum creatinine) - (2.114 / (Serum creatinine^2)) - (0.00686 * Age) - 0.205 (if female))); HbA1c, hemoglobin A1c; HDL, high-density lipoprotein cholesterol; HONK, hyperglycemic hyperosmolar nonketotic state; IQR, inter-quartile-range; MASH, metabolic dysfunction associated

steatohepatitis; MASLD, metabolic dysfunction associated steatotic liver disease; MI, myocardial infarction; N, number of participants; NSAIDs, non-steroidal anti-inflammatory drugs; PCSK9, proprotein convertase subtilisin/kexin type 9; PSA, prostate-specific antigen; PTCA, percutaneous transluminal coronary angioplasty; SGLT2i, sodium-glucose transport protein 2 inhibitors; SD, standard deviation; SMD, standardized mean difference; TIA, transient ischemic attack.
Missing data were handled by assuming absence of a code indicated absence of the condition for most binary covariates. Missing indicators were included for race and region in the propensity score model.

Supplementary Table 16. Baseline characteristics of initiators of tirzepatide vs semaglutide when applying expanded eligibility criteria before and after propensity score matching, across pooled databases. Values are number (percentage) unless otherwise specified.

	Before propensity score matching			After propensity score matching		
Variable	Tirzepatide (n = 86,446)	Semaglutide (n = 193,396)	SMD	Tirzepatide (n = 86,191)	Semaglutide (n = 86,191)	SMD
Demographics						
Age; mean (SD)	59.19 ± 11.61	60.30 ± 11.59	0.095	59.24 ± 11.59	59.26 ± 11.72	0.002
Gender						
Male	38,277 (44.3%)	84,884 (43.9%)	0.008	38,166 (44.3%)	38,117 (44.2%)	0.001
Female	48,169 (55.7%)	108,512 (56.1%)	0.008	48,025 (55.7%)	48,074 (55.8%)	0.001
Race						
White	27,749 (45.5%)	56,020 (45.0%)	0.068	27,648 (45.4%)	27,589 (45.3%)	0.001
Black	7,565 (12.4%)	17,194 (13.8%)	0.005	7,551 (12.4%)	7,535 (12.4%)	0.001
Unknown / Missing	24,905 (40.8%)	49,199 (39.5%)	0.076	24,779 (40.7%)	24,866 (40.9%)	0.002
Others	870 (1.4%)	2,051 (1.7%)	0.005	868 (1.4%)	856 (1.4%)	0.001
Region / State						
Northeast	6,790 (7.9%)	19,159 (9.9%)	0.072	6,789 (7.9%)	6,688 (7.8%)	0.004
Midwest / North central	18,715 (21.6%)	40,737 (21.1%)	0.014	18,664 (21.7%)	18,693 (21.7%)	0.001
South	51,050 (59.1%)	106,326 (55.0%)	0.082	50,849 (59.0%)	50,808 (58.9%)	0.001
West	9,832 (11.4%)	27,050 (14.0%)	0.079	9,831 (11.4%)	9,944 (11.5%)	0.004
Missing	59 (0.1%)	124 (0.1%)	0.002	58 (0.1%)	58 (0.1%)	0.000
Lifestyle risk factors						
Smoking / Tobacco use	16,247 (18.8%)	37,375 (19.3%)	0.014	16,213 (18.8%)	16,255 (18.9%)	0.001
Weight						
Overweight (25.0-29.9)	5,715 (6.6%)	15,168 (7.8%)	0.048	5,712 (6.6%)	5,723 (6.6%)	0.001
Class 1 Obesity (30.0-34.9)	12,763 (14.8%)	30,060 (15.5%)	0.022	12,744 (14.8%)	12,713 (14.7%)	0.001
Class 2 Obesity (35.0-39.9)	20,686 (23.9%)	42,721 (22.1%)	0.044	20,622 (23.9%)	20,589 (23.9%)	0.001
Class 3 Obesity (40.0 and above)	24,517 (28.4%)	50,827 (26.3%)	0.047	24,374 (28.3%)	24,478 (28.4%)	0.003
Unspecified Obesity	22,765 (26.3%)	54,620 (28.2%)	0.043	22,739 (26.4%)	22,688 (26.3%)	0.001
Diabetes complications						
Diabetic retinopathy	5,985 (6.9%)	14,197 (7.3%)	0.016	5,979 (6.9%)	5,944 (6.9%)	0.002
Diabetic neuropathy	17,775 (20.6%)	42,014 (21.7%)	0.028	17,753 (20.6%)	17,886 (20.8%)	0.004
Diabetic nephropathy	14,183 (16.4%)	34,615 (17.9%)	0.040	14,167 (16.4%)	14,227 (16.5%)	0.002
Diabetes with other opthalmic complications	2,457 (2.8%)	6,442 (3.3%)	0.028	2,455 (2.8%)	2,413 (2.8%)	0.003
Diabetes with peripheral circulatory disorders	9,645 (11.2%)	23,017 (11.9%)	0.023	9,627 (11.2%)	9,683 (11.2%)	0.002
Diabetic foot	1,981 (2.3%)	4,613 (2.4%)	0.006	1,978 (2.3%)	1,957 (2.3%)	0.002
Erectile dysfunction	3,912 (4.5%)	8,585 (4.4%)	0.004	3,900 (4.5%)	3,988 (4.6%)	0.005
Hypoglycemia	19,571 (22.6%)	43,136 (22.3%)	0.008	19,509 (22.6%)	19,400 (22.5%)	0.003
Hyperglycemia / DKA / HONK	42,756 (49.5%)	93,949 (48.6%)	0.018	42,600 (49.4%)	42,539 (49.4%)	0.001

Skin infections	8,578 (9.9%)	18,484 (9.6%)	0.012	8,541 (9.9%)	8,566 (9.9%)	0.001
Cardiovascular-related conditions						
Coronary atherosclerosis	14,430 (16.7%)	34,924 (18.1%)	0.036	14,415 (16.7%)	14,483 (16.8%)	0.002
Stable angina	3,055 (3.5%)	7,739 (4.0%)	0.025	3,052 (3.5%)	3,071 (3.6%)	0.001
Unstable angina	1,613 (1.9%)	4,052 (2.1%)	0.016	1,610 (1.9%)	1,617 (1.9%)	0.001
Hypertension	73,734 (85.3%)	165,368 (85.5%)	0.006	73,529 (85.3%)	73,475 (85.2%)	0.002
Hypotension	1,915 (2.2%)	4,686 (2.4%)	0.014	1,914 (2.2%)	1,865 (2.2%)	0.004
Hyperlipidemia	71,681 (82.9%)	159,984 (82.7%)	0.005	71,467 (82.9%)	71,458 (82.9%)	0.000
Acute MI	672 (0.8%)	2,011 (1.0%)	0.028	671 (0.8%)	692 (0.8%)	0.003
Old MI	2,515 (2.9%)	6,435 (3.3%)	0.024	2,513 (2.9%)	2,506 (2.9%)	0.000
Ischemic stroke	408 (0.5%)	1,315 (0.7%)	0.027	408 (0.5%)	400 (0.5%)	0.001
TIA	1,114 (1.3%)	2,883 (1.5%)	0.017	1,109 (1.3%)	1,132 (1.3%)	0.002
Cardiac conduction disorder	3,783 (4.4%)	9,389 (4.9%)	0.023	3,779 (4.4%)	3,772 (4.4%)	0.000
Previous cardiac procedure (CABG, PTCA, Stent)	1,029 (1.2%)	2,834 (1.5%)	0.024	1,029 (1.2%)	1,065 (1.2%)	0.004
PVD diagnosis or surgery	5,940 (6.9%)	14,416 (7.5%)	0.023	5,935 (6.9%)	5,900 (6.8%)	0.002
Atrial fibrillation	6,677 (7.7%)	16,168 (8.4%)	0.023	6,667 (7.7%)	6,755 (7.8%)	0.004
Other cardiac dysrhythmia	15,232 (17.6%)	35,394 (18.3%)	0.018	15,194 (17.6%)	15,379 (17.8%)	0.006
Heart failure	8,647 (10.0%)	21,656 (11.2%)	0.039	8,641 (10.0%)	8,761 (10.2%)	0.005
Acute heart failure	1,945 (2.2%)	5,252 (2.7%)	0.030	1,943 (2.3%)	1,952 (2.3%)	0.001
Cardiomyopathy	3,293 (3.8%)	8,451 (4.4%)	0.028	3,290 (3.8%)	3,346 (3.9%)	0.003
Valve disorders	7,341 (8.5%)	17,631 (9.1%)	0.022	7,338 (8.5%)	7,343 (8.5%)	0.000
Valve replacement	690 (0.8%)	1,836 (0.9%)	0.016	690 (0.8%)	675 (0.8%)	0.002
Edema	10,047 (11.6%)	22,489 (11.6%)	0.000	10,018 (11.6%)	10,029 (11.6%)	0.000
Venous thromboembolism / Pulmonary embolism	2,421 (2.8%)	5,525 (2.9%)	0.003	2,417 (2.8%)	2,411 (2.8%)	0.000
Pulmonary hypertension	1,974 (2.3%)	4,763 (2.5%)	0.012	1,972 (2.3%)	1,962 (2.3%)	0.001
Implantable cardioverter defibrillator	184 (0.2%)	414 (0.2%)	0.000	184 (0.2%)	203 (0.2%)	0.005
Hyperkalemia	1,778 (2.1%)	4,775 (2.5%)	0.028	1,777 (2.1%)	1,719 (2.0%)	0.005
Cerebrovascular procedure	64 (0.1%)	203 (0.1%)	0.010	64 (0.1%)	52 (0.1%)	0.005
Insertion of pacemakers / removal of cardiac lead	98 (0.1%)	249 (0.1%)	0.004	98 (0.1%)	98 (0.1%)	0.000
Renal-related conditions						
Hypertensive nephropathy	7,817 (9.0%)	19,779 (10.2%)	0.040	7,814 (9.1%)	7,817 (9.1%)	0.000
CKD Stage 1-2	3,529 (4.1%)	8,204 (4.2%)	0.008	3,521 (4.1%)	3,583 (4.2%)	0.004
CKD Stage 3-4	9,320 (10.8%)	23,587 (12.2%)	0.044	9,317 (10.8%)	9,238 (10.7%)	0.003
Unspecified CKD	3,494 (4.0%)	9,015 (4.7%)	0.030	3,491 (4.1%)	3,479 (4.0%)	0.001
Microalbuminuria or proteinuria	4,566 (5.3%)	10,461 (5.4%)	0.006	4,556 (5.3%)	4,452 (5.2%)	0.005
Acute kidney injury	3,731 (4.3%)	9,643 (5.0%)	0.032	3,729 (4.3%)	3,762 (4.4%)	0.002
Urinary tract infections	10,161 (11.8%)	22,908 (11.8%)	0.003	10,137 (11.8%)	10,058 (11.7%)	0.003
Genital infections	2,649 (3.1%)	6,397 (3.3%)	0.014	2,646 (3.1%)	2,620 (3.0%)	0.002
Urolithiasis (Kidney and urinary stone)	3,825 (4.4%)	8,586 (4.4%)	0.001	3,816 (4.4%)	3,805 (4.4%)	0.001

**Other comorbidities**						
COPD	7,862 (9.1%)	19,390 (10.0%)	0.032	7,860 (9.1%)	7,909 (9.2%)	0.002
Asthma	10,167 (11.8%)	22,612 (11.7%)	0.002	10,140 (11.8%)	10,145 (11.8%)	0.000
Obstructive sleep apnea	28,491 (33.0%)	61,012 (31.5%)	0.030	28,394 (32.9%)	28,358 (32.9%)	0.001
Serious bacterial infections	1,987 (2.3%)	4,903 (2.5%)	0.015	1,983 (2.3%)	1,973 (2.3%)	0.001
Pneumonia	3,140 (3.6%)	7,847 (4.1%)	0.022	3,133 (3.6%)	3,114 (3.6%)	0.001
Liver disease	14,618 (16.9%)	31,991 (16.5%)	0.010	14,548 (16.9%)	14,477 (16.8%)	0.002
MASH / MASLD	9,270 (10.7%)	19,290 (10.0%)	0.025	9,214 (10.7%)	9,152 (10.6%)	0.002
Fractures / Falls	4,081 (4.7%)	9,594 (5.0%)	0.011	4,067 (4.7%)	4,196 (4.9%)	0.007
Osteoporosis	2,701 (3.1%)	6,686 (3.5%)	0.019	2,701 (3.1%)	2,734 (3.2%)	0.002
Osteoarthritis	23,309 (27.0%)	53,449 (27.6%)	0.015	23,274 (27.0%)	23,372 (27.1%)	0.003
Depression	17,059 (19.7%)	38,832 (20.1%)	0.009	17,010 (19.7%)	17,046 (19.8%)	0.001
Dementia	1,661 (1.9%)	4,679 (2.4%)	0.034	1,661 (1.9%)	1,662 (1.9%)	0.000
Delirium or psychosis	883 (1.0%)	2,530 (1.3%)	0.027	883 (1.0%)	875 (1.0%)	0.001
Anxiety	19,997 (23.1%)	43,544 (22.5%)	0.015	19,929 (23.1%)	19,771 (22.9%)	0.004
Sleep disorders	28,314 (32.8%)	61,521 (31.8%)	0.020	28,221 (32.7%)	28,233 (32.8%)	0.000
Anemia	14,270 (16.5%)	32,729 (16.9%)	0.011	14,226 (16.5%)	14,207 (16.5%)	0.001
Influenza	1,968 (2.3%)	3,736 (1.9%)	0.024	1,956 (2.3%)	1,969 (2.3%)	0.001
COVID	9,873 (11.4%)	23,440 (12.1%)	0.022	9,847 (11.4%)	9,944 (11.5%)	0.004
Hyperthyroidism and other thyroid gland disorders	21,749 (25.2%)	46,971 (24.3%)	0.020	21,679 (25.2%)	21,772 (25.3%)	0.002
Hypothyroidism	17,425 (20.2%)	37,546 (19.4%)	0.019	17,371 (20.2%)	17,404 (20.2%)	0.001
Nephrotic syndrome	58 (0.1%)	145 (0.1%)	0.003	57 (0.1%)	51 (0.1%)	0.003
Urinary incontinence	3,916 (4.5%)	9,364 (4.8%)	0.015	3,909 (4.5%)	3,867 (4.5%)	0.002
Biliary disease	131 (0.2%)	314 (0.2%)	0.003	129 (0.1%)	123 (0.1%)	0.002
Pancreatitis	84 (0.1%)	199 (0.1%)	0.002	84 (0.1%)	94 (0.1%)	0.004
Bowel obstruction	111 (0.1%)	220 (0.1%)	0.004	110 (0.1%)	122 (0.1%)	0.004
Gastroparesis	710 (0.8%)	1,314 (0.7%)	0.016	696 (0.8%)	713 (0.8%)	0.002
**Diabetes medications**						
Number of antidiabetic drugs on CED, mean (SD)	2.07 ± 0.97	2.10 ± 0.96	0.029	2.07 ± 0.97	2.07 ± 0.96	0.003
Concomitant use or initiation of Metformin	42,185 (48.8%)	97,584 (50.5%)	0.033	42,087 (48.8%)	42,204 (49.0%)	0.003
Concomitant use or initiation of Insulins	13,972 (16.2%)	31,528 (16.3%)	0.004	13,932 (16.2%)	13,906 (16.1%)	0.001
Concomitant use or initiation of Sulfonylureas	11,512 (13.3%)	29,250 (15.1%)	0.052	11,495 (13.3%)	11,418 (13.2%)	0.003
Concomitant use or initiation of DPP-4i	2,903 (3.4%)	7,075 (3.7%)	0.016	2,899 (3.4%)	2,846 (3.3%)	0.003
Concomitant use or initiation of SGLT-2i	15,947 (18.4%)	33,784 (17.5%)	0.025	15,897 (18.4%)	15,928 (18.5%)	0.001
Concomitant use or initiation of Any other glucose-lowering drugs	4,381 (5.1%)	9,604 (5.0%)	0.005	4,370 (5.1%)	4,384 (5.1%)	0.001
Past use of Metformin	56,715 (65.6%)	129,786 (67.1%)	0.032	56,572 (65.6%)	56,653 (65.7%)	0.002
Past use of Insulins	18,734 (21.7%)	41,902 (21.7%)	0.000	18,681 (21.7%)	18,598 (21.6%)	0.002
Past use of Sulfonylureas	16,803 (19.4%)	41,675 (21.5%)	0.052	16,778 (19.5%)	16,762 (19.4%)	0.000
Past use of DPP-4i	4,906 (5.7%)	11,825 (6.1%)	0.019	4,893 (5.7%)	4,785 (5.6%)	0.005

Past use of SGLT-2i	21,786 (25.2%)	45,782 (23.7%)	0.036	21,707 (25.2%)	21,687 (25.2%)	0.001
Past use of Any other glucose-lowering drugs	6,206 (7.2%)	13,478 (7.0%)	0.008	6,190 (7.2%)	6,185 (7.2%)	0.000
**Other medications**						
ACE / ARB	61,455 (71.1%)	138,338 (71.5%)	0.010	61,304 (71.1%)	61,148 (70.9%)	0.004
ARNI	1,349 (1.6%)	3,277 (1.7%)	0.011	1,347 (1.6%)	1,378 (1.6%)	0.003
Thiazides	30,520 (35.3%)	67,654 (35.0%)	0.007	30,433 (35.3%)	30,394 (35.3%)	0.001
Beta-blockers	32,436 (37.5%)	75,709 (39.1%)	0.033	32,384 (37.6%)	32,591 (37.8%)	0.005
Calcium channel blockers	26,248 (30.4%)	61,431 (31.8%)	0.030	26,196 (30.4%)	26,243 (30.4%)	0.001
Digoxin / Digitoxin	378 (0.4%)	903 (0.5%)	0.004	378 (0.4%)	351 (0.4%)	0.005
Loop diuretics	12,895 (14.9%)	30,489 (15.8%)	0.024	12,882 (14.9%)	12,893 (15.0%)	0.000
Other diuretics	7,482 (8.7%)	17,343 (9.0%)	0.011	7,464 (8.7%)	7,526 (8.7%)	0.003
Intravenous diuretics	881 (1.0%)	2,229 (1.2%)	0.013	880 (1.0%)	916 (1.1%)	0.004
Nitrates	4,176 (4.8%)	10,581 (5.5%)	0.029	4,173 (4.8%)	4,135 (4.8%)	0.002
Anti-arrhythmics	1,707 (2.0%)	4,065 (2.1%)	0.009	1,705 (2.0%)	1,707 (2.0%)	0.000
Statins	65,006 (75.2%)	150,068 (77.6%)	0.056	64,885 (75.3%)	64,817 (75.2%)	0.002
PCSK9 inhibitors and other lipid-lowering drugs	11,568 (13.4%)	25,082 (13.0%)	0.012	11,539 (13.4%)	11,571 (13.4%)	0.001
Antiplatelet medications	7,167 (8.3%)	17,989 (9.3%)	0.036	7,159 (8.3%)	7,239 (8.4%)	0.003
Oral anticoagulants	7,413 (8.6%)	17,694 (9.1%)	0.020	7,402 (8.6%)	7,426 (8.6%)	0.001
COPD / Asthma medications	31,052 (35.9%)	69,160 (35.8%)	0.003	30,956 (35.9%)	30,907 (35.9%)	0.001
NSAIDS	29,610 (34.3%)	66,515 (34.4%)	0.003	29,544 (34.3%)	29,645 (34.4%)	0.002
Oral corticosteroids	24,256 (28.1%)	52,010 (26.9%)	0.026	24,161 (28.0%)	24,118 (28.0%)	0.001
Osteoporosis agents (incl. bisphosphonates)	1,779 (2.1%)	4,451 (2.3%)	0.017	1,779 (2.1%)	1,786 (2.1%)	0.001
Opioids	25,286 (29.3%)	56,725 (29.3%)	0.002	25,240 (29.3%)	25,265 (29.3%)	0.001
Anti-depressants	33,913 (39.2%)	75,680 (39.1%)	0.002	33,833 (39.3%)	33,810 (39.2%)	0.001
Antipsychotics	3,578 (4.1%)	8,336 (4.3%)	0.009	3,568 (4.1%)	3,556 (4.1%)	0.001
Anxiolytics / hypnotics, benzos	19,236 (22.3%)	41,628 (21.5%)	0.018	19,181 (22.3%)	19,186 (22.3%)	0.000
Dementia medications	700 (0.8%)	1,980 (1.0%)	0.022	700 (0.8%)	736 (0.9%)	0.005
Urinary tract infections antibiotics	41,069 (47.5%)	89,127 (46.1%)	0.029	40,944 (47.5%)	40,896 (47.4%)	0.001
Laxatives	2,927 (3.4%)	6,709 (3.5%)	0.005	2,925 (3.4%)	2,912 (3.4%)	0.001
**Healthcare utilization marker**						
Number of distinct medications; mean (SD)	14.21 ± 7.05	14.26 ± 6.90	0.006	14.22 ± 7.05	14.22 ± 7.03	0.000
Number of office visits; mean (SD)	9.28 ± 6.94	9.32 ± 6.90	0.005	9.28 ± 6.93	9.27 ± 6.97	0.002
Number of endocrinologist visits; mean (SD)	0.42 ± 1.36	0.38 ± 1.29	0.028	0.42 ± 1.34	0.42 ± 1.37	0.000
Number of cardiologist visits; mean (SD)	1.28 ± 2.99	1.38 ± 3.31	0.032	1.28 ± 2.99	1.29 ± 3.07	0.004
Number of internal/family medicine visits; mean (SD)	6.06 ± 6.99	6.34 ± 7.64	0.039	6.06 ± 7.00	6.04 ± 6.88	0.003
Number of electrocardiograms (ECG/EKG); mean (SD)	0.89 ± 1.93	0.98 ± 2.21	0.043	0.89 ± 1.93	0.89 ± 1.84	0.004
Number of echocardiograms; mean (SD)	0.26 ± 0.93	0.30 ± 1.21	0.037	0.26 ± 0.93	0.27 ± 0.98	0.011
Out-of-pocket medication cost; mean (SD)	585.49 ± 874.07	560.08 ± 830.29	0.030	583.99 ± 831.09	578.25 ± 871.37	0.007
Unique brand medicines; mean (SD)	14.44 ± 7.26	14.53 ± 7.11	0.013	14.45 ± 7.25	14.45 ± 7.21	0.000

Unique generic medicines; mean (SD)	14.21 ± 7.05	14.26 ± 6.90	0.006	14.22 ± 7.05	14.22 ± 7.03	0.000
Ratio of brand to generic medications; mean (SD)	1.01 ± 0.04	1.02 ± 0.04	0.250	1.01 ± 0.04	1.01 ± 0.04	0.000
**Healthy behavior markers**						
Colonoscopy / Sigmoidoscopy	9,395 (10.9%)	21,254 (11.0%)	0.004	9,377 (10.9%)	9,251 (10.7%)	0.005
Flu Pneumococcal vaccine	24,935 (28.8%)	59,392 (30.7%)	0.041	24,895 (28.9%)	24,898 (28.9%)	0.000
Pap smear	8,144 (9.4%)	17,018 (8.8%)	0.022	8,081 (9.4%)	8,064 (9.4%)	0.001
PSA test	19,230 (22.2%)	41,518 (21.5%)	0.019	19,190 (22.3%)	19,128 (22.2%)	0.002
Fecal occult blood test	3,132 (3.6%)	7,756 (4.0%)	0.020	3,130 (3.6%)	3,102 (3.6%)	0.002
Bone mineral density tests	5,370 (6.2%)	12,273 (6.3%)	0.006	5,365 (6.2%)	5,346 (6.2%)	0.001
Mammograms	23,710 (27.4%)	53,352 (27.6%)	0.004	23,658 (27.4%)	23,538 (27.3%)	0.003
Telemedicine	21,938 (25.4%)	51,868 (26.8%)	0.033	21,862 (25.4%)	21,958 (25.5%)	0.003
**Laboratory and diagnostic tests**						
HbA1c tests; mean (SD)	2.27 ± 1.35	2.27 ± 1.38	0.005	2.27 ± 1.35	2.26 ± 1.35	0.007
Lipid panels; mean (SD)	1.63 ± 1.15	1.60 ± 1.21	0.029	1.63 ± 1.15	1.63 ± 1.20	0.000
Creatinine tests; mean (SD)	1.96 ± 2.68	1.89 ± 2.71	0.027	1.96 ± 2.68	1.96 ± 2.58	0.003
Natriuretic peptide tests; mean (SD)	0.15 ± 0.66	0.16 ± 0.78	0.017	0.15 ± 0.66	0.15 ± 0.70	0.000
Urine tests; mean (SD)	0.99 ± 1.61	1.00 ± 1.63	0.005	0.99 ± 1.61	0.98 ± 1.58	0.003
**Lab values**						
HbA1c (%)*; mean (SD) median [IQR]	7.59 ±1.71 7.10 [6.40, 8.40]	7.59 ±1.69 7.20 [6.40, 8.40]	0.002	7.59 ±1.71 7.10 [6.40, 8.40]	7.57 ±1.70 7.10 [6.40, 8.30]	0.012
Glucose (mg/dl)*; mean (SD) median [IQR]	154.91 ±66.87 137.00 [111.00, 179.00]	156.16 ±67.64 138.00 [111.50, 181.00]	0.019	154.88 ±66.82 137.00 [111.00, 179.00]	155.14 ±67.04 137.00 [111.00, 179.00]	0.004
Creatinine (mg/dl)*; mean (SD) median [IQR]	1.01 ±1.33 0.89 [0.80, 1.07]	1.02 ±0.90 0.90 [0.80, 1.10]	0.012	1.01 ±1.33 0.89 [0.80, 1.08]	1.00 ±0.97 0.89 [0.80, 1.08]	0.005
Systolic blood pressure (mmHg)*; mean (SD) median [IQR]	129.34 ±22.75 130.00 [120.00, 139.00]	129.56 ±20.62 130.00 [120.00, 139.00]	0.010	129.33 ±22.75 130.00 [120.00, 139.00]	129.21 ±21.25 130.00 [120.00, 139.00]	0.005
Heart rate (1/min)*; mean (SD) median [IQR]	78.69 ±14.99 78.00 [69.00, 87.00]	78.38 ±48.88 77.00 [69.00, 87.00]	0.008	78.65 ±14.98 78.00 [69.00, 87.00]	78.50 ±13.92 77.00 [69.00, 87.00]	0.011
BMI (kg/m2)*; mean (SD) median [IQR]	38.66 ±8.06 37.31 [32.99, 42.76]	37.69 ±7.70 36.40 [32.30, 41.57]	0.123	38.65 ±8.06 37.30 [32.97, 42.74]	38.19 ±7.77 36.92 [32.80, 42.13]	0.057
eGFR (ml/min/1.73m2)*; mean (SD) median [IQR]	2,664.87 ±160,746.30 83.00 [65.00, 98.00]	1,584.71 ±119,300.02 80.00 [61.00, 95.00]	0.008	2,676.77 ±161,118.82 83.00 [65.00, 98.00]	1,070.00 ±99,497.99 82.00 [64.00, 97.00]	0.012
LDL (mg/dl)*; mean (SD) median [IQR]	87.28 ±39.49 83.00 [62.00, 110.00]	85.77 ±38.91 81.80 [61.00, 108.00]	0.039	87.22 ±39.47 83.00 [62.00, 110.00]	87.22 ±39.48 83.00 [62.00, 110.00]	0.000
HDL (mg/dl)*; mean (SD) median [IQR]	46.00 ±13.84 45.00 [37.00, 53.00]	46.40 ±14.19 45.00 [38.00, 54.00]	0.028	46.02 ±13.85 45.00 [37.00, 53.00]	46.20 ±14.10 45.00 [37.00, 54.00]	0.013
Total cholesterol (mg/dl)*; mean (SD) median [IQR]	165.41 ±49.48 161.00 [135.00, 192.00]	163.48 ±48.54 159.00 [133.00, 190.00]	0.039	165.35 ±49.50 161.00 [135.00, 192.00]	165.31 ±49.04 161.00 [135.00, 193.00]	0.001
Triglyceride (mg/dl)*; mean (SD) median [IQR]	176.03 ±150.57 144.00 [104.00, 205.00]	172.38 ±147.65 143.00 [103.00, 202.00]	0.024	176.00 ±150.72 144.00 [104.00, 205.00]	175.09 ±154.99 144.00 [103.00, 204.00]	0.006
**Burden of comorbidities**						
Combined comorbidity score; mean (SD)	1.53 ± 2.12	1.64 ± 2.22	0.047	1.53 ± 2.13	1.54 ± 2.13	0.005
Frailty Score; mean (SD)	0.15 ± 0.05	0.16 ± 0.06	0.099	0.15 ± 0.05	0.15 ± 0.05	0.000
**Baseline hospitalizations and hospital metrics**						

Number of Hospitalizations; mean (SD)	1.22 ± 11.06	1.73 ± 16.17	0.037	1.22 ± 10.95	1.26 ± 16.98	0.003
Any hospitalization within prior 91 days	2,714 (3.1%)	7,146 (3.7%)	0.031	2,710 (3.1%)	2,715 (3.1%)	0.000
Any hospitalization within prior 92-365 days	6,577 (7.6%)	16,490 (8.5%)	0.034	6,566 (7.6%)	6,590 (7.6%)	0.001
Number of hospitalizations (0, 1, 2 or more)						
< 1	78,085 (90.3%)	172,227 (89.1%)	0.042	77,843 (90.3%)	77,792 (90.3%)	0.002
1 - <2	4,861 (5.6%)	11,260 (5.8%)	0.009	4,853 (5.6%)	4,898 (5.7%)	0.002
>= 2	3,500 (4.0%)	9,909 (5.1%)	0.051	3,495 (4.1%)	3,501 (4.1%)	0.000
Heart failure hospitalization	2,053 (2.4%)	5,710 (3.0%)	0.036	2,051 (2.4%)	2,100 (2.4%)	0.004
ED visit	22,928 (26.5%)	56,075 (29.0%)	0.055	22,894 (26.6%)	23,039 (26.7%)	0.004
**Calendar year of cohort entry**						
2022	10,030 (11.6%)	38,805 (20.1%)	0.233	10,029 (11.6%)	9,803 (11.4%)	0.008
2023	45,550 (52.7%)	101,033 (52.2%)	0.009	45,525 (52.8%)	45,620 (52.9%)	0.002
2024	29,475 (34.1%)	52,182 (27.0%)	0.155	29,382 (34.1%)	29,553 (34.3%)	0.004
2025	1,391 (1.6%)	1,376 (0.7%)	0.084	1,255 (1.5%)	1,215 (1.4%)	0.004

Abbreviations: *not used in the propensity score; ACE, angiotensin-converting enzyme inhibitors; ARB, angiotensin receptor blocker; ARNI, angiotensin receptor/neprilysin inhibitor; BMI, body mass index; CABG, coronary artery bypass graft surgery; CED, cohort entry date; CKD, chronic kidney disease; COPD, chronic obstructive pulmonary disease; DKA, diabetic ketoacidosis; DPP4i, dipeptidyl peptidase-4 inhibitors; ED, emergency department; eGFR, estimated glomerular filtration rate (estimated using the quadratic GFR equation: GFR = EXP(1.911 + (5.249 / Serum creatinine) - (2.114 / (Serum creatinine^2)) - (0.00686 * Age) - 0.205 (if female))); HbA1c, hemoglobin A1c; HDL, high-density lipoprotein cholesterol; HONK, hyperglycemic hyperosmolar nonketotic state; IQR, inter-quartile-range; MASH, metabolic dysfunction associated steatohepatitis; MASLD, metabolic dysfunction associated steatotic liver disease; MI, myocardial infarction; N, number of participants; NSAIDs, non-steroidal anti-inflammatory drugs; PCSK9, proprotein convertase subtilisin/kexin type 9; PSA, prostate-specific antigen; PTCA, percutaneous transluminal coronary angioplasty; SGLT2i, sodium-glucose transport protein 2 inhibitors; SD, standard deviation; SMD, standardized mean difference; TIA, transient ischemic attack.

Missing data were handled by assuming absence of a code indicated absence of the condition for most binary covariates. Missing indicators were included for race and region in the propensity score model.

Laboratory values were only available in a subset of the Optum database and were truncated using clinically plausible cut-off values (BMI values >100 or <10 were set to missing; missingness before matching ~53%. Creatinine values >30 were set to missing, and values <0.8 were set to 0.8; missingness before matching ~40%. eGFR values >150 were set to 150, and values =0 were set to missing; missingness before matching ~51%. Glucose values <30 were set to missing; missingness before matching ~40%. HbA1c values ≥20 or ≤2 were set to missing; missingness before matching ~34%. HDL value missingness before matching was ~47%. Heart rate values <30 were set to missing; missingness before matching ~66%. LDL value missingness before matching was~45%. Systolic blood pressure values <30 were set to missing; missingness before matching ~50%. Total cholesterol value missingness before matching was ~47%. Triglyceride value missingness before matching was ~48%).

Race was reported by the respective database: Optum. The Race-Others category includes Asian and Hispanic individuals.

Supplementary Table 17. Baseline characteristics of initiators of tirzepatide vs semaglutide when applying expanded eligibility criteria before and after propensity score matching. Optum database. Values are number (percentage) unless otherwise specified.

	Before propensity score matching			After propensity score matching		
Variable	Tirzepatide (n = 61,089)	Semaglutide (n = 124,464)	SMD	Tirzepatide (n = 60,846)	Semaglutide (n = 60,846)	SMD
**Demographics**						
Age; mean (SD) median [IQR]	61.44 ±11.51 63.00 [54.00, 70.00]	63.53 ±11.03 66.00 [56.00, 71.00]	0.185	61.51 ±11.46 64.00 [54.00, 70.00]	61.55 ±11.58 64.00 [54.00, 70.00]	0.003
Gender						
Male	27,034 (44.3%)	54,096 (43.5%)	0.016	26,930 (44.3%)	26,915 (44.2%)	0.000
Female	34,055 (55,7%)	70,368 (56,5%)	0.016	33,916 (55,7%)	33,931 (55,8%)	0.000
Race						
White	27,749 (45.4%)	56,020 (45.0%)	0.008	27,648 (45.4%)	27,589 (45.3%)	0.002
Black	7,565 (12.4%)	17,194 (13.8%)	0.042	7,551 (12.4%)	7,535 (12.4%)	0.001
Unknown / Missing	24,905 (40.8%)	49,199 (39.5%)	0.025	24,779 (40.7%)	24,866 (40.9%)	0.003
Others	870 (1.4%)	2,051 (1.6%)	0.018	868 (1.4%)	856 (1.4%)	0.002
Region / State						
Northeast	4,597 (7.5%)	11,458 (9.2%)	0.061	4,596 (7.6%)	4,536 (7.5%)	0.004
Midwest / North central	13,147 (21.5%)	25,578 (20.6%)	0.024	13,099 (21.5%)	13,125 (21.6%)	0.001
South	35,370 (57.9%)	66,844 (53.7%)	0.085	35,178 (57.8%)	35,165 (57.8%)	0.000
West	7,938 (13.0%)	20,532 (16.5%)	0.099	7,937 (13.0%)	7,984 (13.1%)	0.002
Missing	37 (0.1%)	52 (0.0%)	0.008	36 (0.1%)	36 (0.1%)	0.000
**Lifestyle risk factors**						
Smoking / Tobacco use	13,928 (22.8%)	30,626 (24.6%)	0.042	13,894 (22.8%)	13,987 (23.0%)	0.004
Weight						
Overweight (25.0-29.9)	3,744 (6.1%)	9,152 (7.4%)	0.049	3,742 (6.1%)	3,746 (6.2%)	0.000
Class 1 Obesity (30.0-34.9)	10,773 (17.6%)	24,457 (19.6%)	0.052	10,757 (17.7%)	10,743 (17.7%)	0.001
Class 2 Obesity (35.0-39.9)	18,927 (31.0%)	38,112 (30.6%)	0.008	18,863 (31.0%)	18,870 (31.0%)	0.000
Class 3 Obesity (40.0 and above)	18,995 (31.1%)	34,832 (28.0%)	0.068	18,855 (31.0%)	18,873 (31.0%)	0.001
Unspecified Obesity	8,650 (14.2%)	17,911 (14.4%)	0.007	8,629 (14.2%)	8,614 (14.2%)	0.001
**Diabetes complications**						
Diabetic retinopathy	4,649 (7.6%)	10,581 (8.5%)	0.033	4,643 (7.6%)	4,619 (7.6%)	0.001
Diabetic neuropathy	14,434 (23.6%)	32,680 (26.3%)	0.061	14,415 (23.7%)	14,505 (23.8%)	0.003
Diabetic nephropathy	11,637 (19.0%)	27,397 (22.0%)	0.073	11,622 (19.1%)	11,671 (19.2%)	0.002
Diabetes with other opthalmic complications	2,138 (3.5%)	5,448 (4.4%)	0.045	2,136 (3.5%)	2,090 (3.4%)	0.004
Diabetes with peripheral circulatory disorders	7,894 (12.9%)	18,229 (14.6%)	0.050	7,878 (12.9%)	7,913 (13.0%)	0.002
Diabetic foot	1,652 (2.7%)	3,637 (2.9%)	0.013	1,649 (2.7%)	1,646 (2.7%)	0.000
Erectile dysfunction	3,050 (5.0%)	6,091 (4.9%)	0.005	3,039 (5.0%)	3,079 (5.1%)	0.003
Hypoglycemia	14,971 (24.5%)	31,298 (25.1%)	0.015	14,914 (24.5%)	14,798 (24.3%)	0.004

Hyperglycemia / DKA / HONK	30,454 (49.9%)	61,620 (49.5%)	0.007	30,307 (49.8%)	30,262 (49.7%)	0.001
Skin infections	6,441 (10.5%)	12,766 (10.3%)	0.009	6,405 (10.5%)	6,447 (10.6%)	0.002
Cardiovascular-related conditions						
Coronary atherosclerosis	11,978 (19.6%)	27,304 (21.9%)	0.057	11,964 (19.7%)	11,994 (19.7%)	0.001
Stable angina	2,606 (4.3%)	6,228 (5.0%)	0.035	2,604 (4.3%)	2,606 (4.3%)	0.000
Unstable angina	1,325 (2.2%)	3,044 (2.4%)	0.018	1,322 (2.2%)	1,334 (2.2%)	0.001
Hypertension	53,371 (87.4%)	110,335 (88.6%)	0.039	53,178 (87.4%)	53,135 (87.3%)	0.002
Hypotension	1,648 (2.7%)	3,805 (3.1%)	0.022	1,648 (2.7%)	1,614 (2.7%)	0.003
Hyperlipidemia	51,832 (84.8%)	106,296 (85.4%)	0.016	51,629 (84.9%)	51,565 (84.7%)	0.003
Acute MI	490 (0.8%)	1,418 (1.1%)	0.034	490 (0.8%)	511 (0.8%)	0.004
Old MI	2,235 (3.7%)	5,397 (4.3%)	0.035	2,234 (3.7%)	2,210 (3.6%)	0.002
Ischemic stroke	285 (0.5%)	835 (0.7%)	0.027	285 (0.5%)	270 (0.4%)	0.004
TIA	887 (1.5%)	2,172 (1.7%)	0.023	882 (1.4%)	900 (1.5%)	0.002
Cardiac conduction disorder	3,180 (5.2%)	7,504 (6.0%)	0.036	3,176 (5.2%)	3,167 (5.2%)	0.001
Previous cardiac procedure (CABG, PTCA, Stent)	800 (1.3%)	2,068 (1.7%)	0.029	800 (1.3%)	831 (1.4%)	0.004
PVD diagnosis or surgery	5,099 (8.3%)	12,113 (9.7%)	0.048	5,094 (8.4%)	5,096 (8.4%)	0.000
Atrial fibrillation	5,568 (9.1%)	12,842 (10.3%)	0.041	5,559 (9.1%)	5,667 (9.3%)	0.006
Other cardiac dysrhythmia	11,891 (19.5%)	25,862 (20.8%)	0.033	11,855 (19.5%)	11,988 (19.7%)	0.006
Heart failure	7,465 (12.2%)	17,834 (14.3%)	0.062	7,461 (12.3%)	7,571 (12.4%)	0.005
Acute heart failure	1,697 (2.8%)	4,282 (3.4%)	0.038	1,697 (2.8%)	1,716 (2.8%)	0.002
Cardiomyopathy	2,695 (4.4%)	6,554 (5.3%)	0.040	2,692 (4.4%)	2,759 (4.5%)	0.005
Valve disorders	6,049 (9.9%)	13,800 (11.1%)	0.039	6,046 (9.9%)	6,063 (10.0%)	0.001
Valve replacement	605 (1.0%)	1,510 (1.2%)	0.021	605 (1.0%)	597 (1.0%)	0.001
Edema	8,276 (13.5%)	17,516 (14.1%)	0.015	8,247 (13.6%)	8,260 (13.6%)	0.001
Venous thromboembolism / Pulmonary embolism	1,917 (3.1%)	4,042 (3.2%)	0.006	1,913 (3.1%)	1,913 (3.1%)	0.000
Pulmonary hypertension	1,736 (2.8%)	4,009 (3.2%)	0.022	1,734 (2.8%)	1,720 (2.8%)	0.001
Implantable cardioverter defibrillator	151 (0.2%)	299 (0.2%)	0.001	151 (0.2%)	169 (0.3%)	0.006
Hyperkalemia	1,527 (2.5%)	3,888 (3.1%)	0.038	1,526 (2.5%)	1,495 (2.5%)	0.003
Cerebrovascular procedure	55 (0.1%)	172 (0.1%)	0.014	55 (0.1%)	41 (0.1%)	0.008
Insertion of pacemakers / removal of cardiac lead	89 (0.1%)	220 (0.2%)	0.008	89 (0.1%)	83 (0.1%)	0.003
Renal-related conditions						
Hypertensive nephropathy	6,778 (11.1%)	16,767 (13.5%)	0.072	6,776 (11.1%)	6,765 (11.1%)	0.001
CKD Stage 1-2	3,008 (4.9%)	6,855 (5.5%)	0.026	3,001 (4.9%)	3,052 (5.0%)	0.004
CKD Stage 3-4	8,251 (13.5%)	20,201 (16.2%)	0.077	8,249 (13.6%)	8,213 (13.5%)	0.002
Unspecified CKD	3,127 (5.1%)	7,809 (6.3%)	0.050	3,125 (5.1%)	3,105 (5.1%)	0.001
Microalbuminuria or proteinuria	3,741 (6.1%)	8,134 (6.5%)	0.017	3,731 (6.1%)	3,642 (6.0%)	0.006
Acute kidney injury	3,127 (5.1%)	7,820 (6.3%)	0.050	3,126 (5.1%)	3,159 (5.2%)	0.002
Urinary tract infections	7,787 (12.7%)	16,712 (13.4%)	0.020	7,764 (12.8%)	7,692 (12.6%)	0.004
Genital infections	1,724 (2.8%)	3,778 (3.0%)	0.013	1,721 (2.8%)	1,676 (2.8%)	0.004

Urolithiasis (Kidney and urinary stone)	2,818 (4.6%)	5,842 (4.7%)	0.004	2,810 (4.6%)	2,810 (4.6%)	0.000
Other comorbidities						
COPD	6,932 (11.3%)	16,510 (13.3%)	0.058	6,930 (11.4%)	6,976 (11.5%)	0.002
Asthma	7,805 (12.8%)	16,060 (12.9%)	0.004	7,779 (12.8%)	7,760 (12.8%)	0.001
Obstructive sleep apnea	20,910 (34.2%)	40,961 (32.9%)	0.028	20,819 (34.2%)	20,696 (34.0%)	0.004
Serious bacterial infections	1,464 (2.4%)	3,363 (2.7%)	0.019	1,462 (2.4%)	1,447 (2.4%)	0.002
Pneumonia	2,495 (4.1%)	5,894 (4.7%)	0.032	2,489 (4.1%)	2,482 (4.1%)	0.001
Liver disease	11,110 (18.2%)	22,531 (18.1%)	0.002	11,045 (18.2%)	10,998 (18.1%)	0.002
MASH / MASLD	6,897 (11.3%)	12,871 (10.3%)	0.031	6,843 (11.2%)	6,820 (11.2%)	0.001
Fractures / Falls	3,367 (5.5%)	7,543 (6.1%)	0.024	3,353 (5.5%)	3,434 (5.6%)	0.006
Osteoporosis	2,412 (3.9%)	5,807 (4.7%)	0.035	2,412 (4.0%)	2,425 (4.0%)	0.001
Osteoarthritis	18,494 (30.3%)	39,975 (32.1%)	0.040	18,462 (30.3%)	18,569 (30.5%)	0.004
Depression	13,102 (21.4%)	27,793 (22.3%)	0.021	13,056 (21.5%)	13,083 (21.5%)	0.001
Dementia	1,508 (2.5%)	4,092 (3.3%)	0.049	1,508 (2.5%)	1,516 (2.5%)	0.001
Delirium or psychosis	789 (1.3%)	2,150 (1.7%)	0.036	789 (1.3%)	780 (1.3%)	0.001
Anxiety	14,681 (24.0%)	29,246 (23.5%)	0.013	14,614 (24.0%)	14,526 (23.9%)	0.003
Sleep disorders	21,939 (35.9%)	44,883 (36.1%)	0.003	21,854 (35.9%)	21,795 (35.8%)	0.002
Anemia	10,776 (17.6%)	23,300 (18.7%)	0.028	10,734 (17.6%)	10,688 (17.6%)	0.002
Influenza	1,327 (2.2%)	2,349 (1.9%)	0.020	1,316 (2.2%)	1,269 (2.1%)	0.005
COVID	6,549 (10.7%)	13,619 (10.9%)	0.007	6,525 (10.7%)	6,565 (10.8%)	0.002
Hyperthyroidism and other thyroid gland disorders	16,115 (26.4%)	32,597 (26.2%)	0.004	16,050 (26.4%)	16,147 (26.5%)	0.004
Hypothyroidism	13,037 (21.3%)	26,425 (21.2%)	0.003	12,986 (21.3%)	13,019 (21.4%)	0.001
Nephrotic syndrome	49 (0.1%)	107 (0.1%)	0.002	48 (0.1%)	45 (0.1%)	0.002
Urinary incontinence	3,265 (5.3%)	7,488 (6.0%)	0.029	3,258 (5.4%)	3,208 (5.3%)	0.004
Biliary disease	71 (0.1%)	161 (0.1%)	0.004	71 (0.1%)	70 (0.1%)	0.000
Pancreatitis	57 (0.1%)	125 (0.1%)	0.002	57 (0.1%)	57 (0.1%)	0.000
Bowel obstruction	77 (0.1%)	132 (0.1%)	0.006	76 (0.1%)	88 (0.1%)	0.005
Gastroparesis	573 (0.9%)	989 (0.8%)	0.015	560 (0.9%)	561 (0.9%)	0.000
Diabetes medications						
Number of antidiabetic drugs on CED, mean (SD)	2.08 ±0.96 2.00 [1.00, 3.00]	2.12 ±0.96 2.00 [1.00, 3.00]	0.042	2.08 ±0.96 2.00 [1.00, 3.00]	2.08 ±0.95 2.00 [1.00, 3.00]	0.001
Concomitant use or initiation of Metformin	29,476 (48.3%)	61,334 (49.3%)	0.021	29,382 (48.3%)	29,497 (48.5%)	0.004
Concomitant use or initiation of Insulins	10,552 (17.3%)	22,397 (18.0%)	0.019	10,516 (17.3%)	10,511 (17.3%)	0.000
Concomitant use or initiation of Sulfonylureas	8,707 (14.3%)	20,236 (16.3%)	0.056	8,691 (14.3%)	8,588 (14.1%)	0.005
Concomitant use or initiation of DPP-4i	1,273 (2.1%)	2,461 (2.0%)	0.008	1,269 (2.1%)	1,240 (2.0%)	0.003
Concomitant use or initiation of SGLT-2i	10,713 (17.5%)	21,295 (17.1%)	0.011	10,667 (17.5%)	10,714 (17.6%)	0.002
Concomitant use or initiation of Any other glucose-lowering drugs	3,381 (5.5%)	7,079 (5.7%)	0.007	3,370 (5.5%)	3,426 (5.6%)	0.004
Past use of Metformin	39,430 (64.5%)	81,492 (65.5%)	0.019	39,293 (64.6%)	39,413 (64.8%)	0.004
Past use of Insulins	13,940 (22.8%)	29,354 (23.6%)	0.018	13,892 (22.8%)	13,830 (22.7%)	0.002
Past use of Sulfonylureas	12,606 (20.6%)	28,787 (23.1%)	0.060	12,582 (20.7%)	12,541 (20.6%)	0.002

Past use of DPP-4i	2,226 (3.6%)	4,185 (3.4%)	0.015	2,213 (3.6%)	2,193 (3.6%)	0.002
Past use of SGLT-2i	14,884 (24.4%)	29,246 (23.5%)	0.020	14,812 (24.3%)	14,803 (24.3%)	0.000
Past use of Any other glucose-lowering drugs	4,756 (7.8%)	9,773 (7.9%)	0.002	4,741 (7.8%)	4,788 (7.9%)	0.003
Other medications						
ACE / ARB	43,857 (71.8%)	90,657 (72.8%)	0.023	43,713 (71.8%)	43,539 (71.6%)	0.006
ARNI	1,058 (1.7%)	2,404 (1.9%)	0.015	1,057 (1.7%)	1,091 (1.8%)	0.004
Thiazides	21,632 (35.4%)	43,618 (35.0%)	0.008	21,548 (35.4%)	21,494 (35.3%)	0.002
Beta-blockers	24,448 (40.0%)	53,555 (43.0%)	0.061	24,401 (40.1%)	24,576 (40.4%)	0.006
Calcium channel blockers	19,387 (31.7%)	41,981 (33.7%)	0.042	19,338 (31.8%)	19,359 (31.8%)	0.001
Digoxin / Digitoxin	303 (0.5%)	698 (0.6%)	0.009	303 (0.5%)	277 (0.5%)	0.006
Loop diuretics	10,612 (17.4%)	23,870 (19.2%)	0.047	10,601 (17.4%)	10,636 (17.5%)	0.002
Other diuretics	5,595 (9.2%)	12,049 (9.7%)	0.018	5,579 (9.2%)	5,596 (9.2%)	0.001
Intravenous diuretics	762 (1.2%)	1,808 (1.5%)	0.018	761 (1.3%)	795 (1.3%)	0.005
Nitrates	3,410 (5.6%)	8,119 (6.5%)	0.039	3,408 (5.6%)	3,374 (5.5%)	0.002
Anti-arrhythmics	1,374 (2.2%)	3,076 (2.5%)	0.015	1,372 (2.3%)	1,396 (2.3%)	0.003
Statins	47,088 (77.1%)	99,990 (80.3%)	0.080	46,976 (77.2%)	46,958 (77.2%)	0.001
PCSK9 inhibitors and other lipid-lowering drugs	8,538 (14.0%)	17,008 (13.7%)	0.009	8,511 (14.0%)	8,512 (14.0%)	0.000
Antiplatelet medications	5,400 (8.8%)	12,657 (10.2%)	0.045	5,394 (8.9%)	5,475 (9.0%)	0.005
Oral anticoagulants	5,952 (9.7%)	13,469 (10.8%)	0.036	5,943 (9.8%)	6,002 (9.9%)	0.003
COPD / Asthma medications	22,578 (37.0%)	46,266 (37.2%)	0.004	22,489 (37.0%)	22,379 (36.8%)	0.004
NSAIDS	20,681 (33.9%)	42,646 (34.3%)	0.009	20,620 (33.9%)	20,764 (34.1%)	0.005
Oral corticosteroids	17,020 (27.9%)	32,990 (26.5%)	0.03	16,930 (27.8%)	16,853 (27.7%)	0.003
Osteoporosis agents (incl. bisphosphonates)	1,450 (2.4%)	3,548 (2.9%)	0.030	1,450 (2.4%)	1,457 (2.4%)	0.001
Opioids	18,892 (30.9%)	39,600 (31.8%)	0.019	18,851 (31.0%)	18,895 (31.1%)	0.002
Anti-depressants	24,540 (40.2%)	50,808 (40.8%)	0.013	24,465 (40.2%)	24,435 (40.2%)	0.001
Antipsychotics	2,823 (4.6%)	6,244 (5.0%)	0.018	2,813 (4.6%)	2,807 (4.6%)	0.000
Anxiolytics / hypnotics, benzos	13,878 (22.7%)	27,985 (22.5%)	0.006	13,828 (22.7%)	13,848 (22.8%)	0.001
Dementia medications	640 (1.0%)	1,722 (1.4%)	0.031	640 (1.1%)	678 (1.1%)	0.006
Urinary tract infections antibiotics	29,222 (47.8%)	58,609 (47.1%)	0.015	29,106 (47.8%)	29,082 (47.8%)	0.001
Laxatives	2,158 (3.5%)	4,583 (3.7%)	0.008	2,156 (3.5%)	2,106 (3.5%)	0.004
Healthcare utilization marker						
Number of distinct medications; mean (SD) median [IQR]	14.52 ±7.22 13.00 [9.00, 18.00]	14.84 ±7.08 14.00 [10.00, 19.00]	0.045	14.53 ±7.22 13.00 [9.00, 18.00]	14.52 ±7.14 13.00 [9.00, 18.00]	0.001
Number of office visits; mean (SD) median [IQR]	9.67 ±7.18 8.00 [5.00, 13.00]	9.85 ±7.15 8.00 [5.00, 13.00]	0.025	9.67 ±7.18 8.00 [5.00, 13.00]	9.65 ±7.20 8.00 [5.00, 13.00]	0.004
Number of endocrinologist visits; mean (SD) median [IQR]	0.43 ±1.40 0.00 [0.00, 0.00]	0.41 ±1.36 0.00 [0.00, 0.00]	0.016	0.43 ±1.40 0.00 [0.00, 0.00]	0.43 ±1.42 0.00 [0.00, 0.00]	0.005
Number of cardiologist visits; mean (SD) median [IQR]	1.47 ±3.24 0.00 [0.00, 2.00]	1.64 ±3.65 0.00 [0.00, 2.00]	0.048	1.47 ±3.25 0.00 [0.00, 2.00]	1.49 ±3.31 0.00 [0.00, 2.00]	0.005
Number of internal / family medicine visits; mean (SD) median [IQR]	6.68 ±7.45 5.00 [3.00, 8.00]	7.32 ±8.56 5.00 [3.00, 9.00]	0.080	6.69 ±7.46 5.00 [3.00, 8.00]	6.68 ±7.40 5.00 [3.00, 8.00]	0.002
Number of electrocardiograms (ECG/EKG) ; mean (SD) median [IQR]	0.89 ±1.59 0.00 [0.00, 1.00]	0.97 ±1.68 0.00 [0.00, 1.00]	0.049	0.89 ±1.60 0.00 [0.00, 1.00]	0.90 ±1.59 0.00 [0.00, 1.00]	0.008
Number of echocardiograms; mean (SD) median [IQR]	0.25 ±0.60 0.00 [0.00, 0.00]	0.28 ±0.64 0.00 [0.00, 0.00]	0.049	0.25 ±0.60 0.00 [0.00, 0.00]	0.26 ±0.61 0.00 [0.00, 0.00]	0.006

Out-of-pocket medication cost; mean (SD) median [IQR]	595.25 ±793.97 340.16 [124.64, 792.59]	597.62 ±783.12 340.80 [133.10, 792.50]	0.003	595.61 ±792.64 340.78 [124.89, 793.39]	591.39 ±811.04 336.26 [131.10, 778.25]	0.005
Unique brand medicines; mean (SD) median [IQR]	14.75 ±7.43 14.00 [9.00, 19.00]	15.12 ±7.31 14.00 [10.00, 19.00]	0.050	14.76 ±7.43 14.00 [9.00, 19.00]	14.75 ±7.33 14.00 [9.00, 19.00]	0.002
Unique generic medicines; mean (SD) median [IQR]	14.52 ±7.22 13.00 [9.00, 18.00]	14.84 ±7.08 14.00 [10.00, 19.00]	0.045	14.53 ±7.22 13.00 [9.00, 18.00]	14.52 ±7.14 13.00 [9.00, 18.00]	0.001
Ratio of brand to generic medications; mean (SD) median [IQR]	1.01 ±0.04 1.00 [1.00, 1.00]	1.02 ±0.04 1.00 [1.00, 1.00]	0.098	1.01 ±0.04 1.00 [1.00, 1.00]	1.01 ±0.04 1.00 [1.00, 1.00]	0.006
Healthy behavior markers						
Colonoscopy / Sigmoidoscopy	6,647 (10.9%)	13,600 (10.9%)	0.001	6,630 (10.9%)	6,558 (10.8%)	0.004
Flu Pneumococcal vaccine	18,346 (30.0%)	40,281 (32.4%)	0.050	18,308 (30.1%)	18,240 (30.0%)	0.002
Pap smear	4,843 (7.9%)	8,575 (6.9%)	0.040	4,781 (7.9%)	4,707 (7.7%)	0.005
PSA test	13,950 (22.8%)	27,398 (22.0%)	0.020	13,913 (22.9%)	13,832 (22.7%)	0.003
Fecal occult blood test	2,265 (3.7%)	5,260 (4.2%)	0.027	2,263 (3.7%)	2,268 (3.7%)	0.000
Bone mineral density tests	4,505 (7.4%)	9,938 (8.0%)	0.023	4,501 (7.4%)	4,504 (7.4%)	0.000
Mammograms	16,714 (27.4%)	34,465 (27.7%)	0.007	16,664 (27.4%)	16,600 (27.3%)	0.002
Telemedicine	14,492 (23.7%)	29,837 (24.0%)	0.006	14,418 (23.7%)	14,439 (23.7%)	0.001
Laboratory and diagnostic tests						
HbA1c tests; mean (SD) median [IQR]	2.31 ±1.32 2.00 [1.00, 3.00]	2.34 ±1.32 2.00 [1.00, 3.00]	0.020	2.31 ±1.32 2.00 [1.00, 3.00]	2.30 ±1.34 2.00 [1.00, 3.00]	0.004
Lipid panels; mean (SD) median [IQR]	1.65 ±1.14 1.00 [1.00, 2.00]	1.63 ±1.18 1.00 [1.00, 2.00]	0.021	1.65 ±1.14 1.00 [1.00, 2.00]	1.65 ±1.22 1.00 [1.00, 2.00]	0.000
Creatinine tests; mean (SD) median [IQR]	2.76 ±2.82 2.00 [1.00, 4.00]	2.91 ±2.89 2.00 [1.00, 4.00]	0.051	2.76 ±2.82 2.00 [1.00, 4.00]	2.77 ±2.68 2.00 [1.00, 4.00]	0.002
Natriuretic peptide tests; mean (SD) median [IQR]	0.17 ±0.68 0.00 [0.00, 0.00]	0.19 ±0.79 0.00 [0.00, 0.00]	0.026	0.17 ±0.68 0.00 [0.00, 0.00]	0.17 ±0.68 0.00 [0.00, 0.00]	0.003
Urine tests; mean (SD) median [IQR]	1.03 ±1.62 0.00 [0.00, 1.00]	1.05 ±1.64 0.00 [0.00, 2.00]	0.015	1.03 ±1.62 0.00 [0.00, 1.00]	1.02 ±1.59 0.00 [0.00, 1.00]	0.007
Lab values						
HbA1c (%)*; mean (SD) median [IQR]	7.59 ±1.71 7.10 [6.40, 8.40]	7.59 ±1.69 7.20 [6.40, 8.40]	0.002	7.59 ±1.71 7.10 [6.40, 8.40]	7.57 ±1.70 7.10 [6.40, 8.30]	0.012
Glucose (mg/dl)*; mean (SD) median [IQR]	154.91 ±66.87 137.00 [111.00, 179.00]	156.16 ±67.64 138.00 [111.50, 181.00]	0.019	154.88 ±66.82 137.00 [111.00, 179.00]	155.14 ±67.04 137.00 [111.00, 179.00]	0.004
Creatinine (mg/dl)*; mean (SD) median [IQR]	1.01 ±1.33 0.89 [0.80, 1.07]	1.02 ±0.90 0.90 [0.80, 1.10]	0.012	1.01 ±1.33 0.89 [0.80, 1.08]	1.00 ±0.97 0.89 [0.80, 1.08]	0.005
Systolic blood pressure (mmHg)*; mean (SD) median [IQR]	129.34 ±22.75 130.00 [120.00, 139.00]	129.56 ±20.62 130.00 [120.00, 139.00]	0.010	129.33 ±22.75 130.00 [120.00, 139.00]	129.21 ±21.25 130.00 [120.00, 139.00]	0.005
Heart rate (1/min)*; mean (SD) median [IQR]	78.69 ±14.99 78.00 [69.00, 87.00]	78.38 ±48.88 77.00 [69.00, 87.00]	0.008	78.65 ±14.98 78.00 [69.00, 87.00]	78.50 ±13.92 77.00 [69.00, 87.00]	0.011
BMI (kg/m2)*; mean (SD) median [IQR]	38.66 ±8.06 37.31 [32.99, 42.76]	37.69 ±7.70 36.40 [32.30, 41.57]	0.123	38.65 ±8.06 37.30 [32.97, 42.74]	38.19 ±7.77 36.92 [32.80, 42.13]	0.057
eGFR (ml/min/1.73m2)*; mean (SD) median [IQR]	2,664.87 ±160,746.30 83.00 [65.00, 98.00]	1,584.71 ±119,300.02 80.00 [61.00, 95.00]	0.008	2,676.77 ±161,118.82 83.00 [65.00, 98.00]	1,070.00 ±99,497.99 82.00 [64.00, 97.00]	0.012
LDL (mg/dl)*; mean (SD) median [IQR]	87.28 ±39.49 83.00 [62.00, 110.00]	85.77 ±38.91 81.80 [61.00, 108.00]	0.039	87.22 ±39.47 83.00 [62.00, 110.00]	87.22 ±39.48 83.00 [62.00, 110.00]	0.000
HDL (mg/dl)*; mean (SD) median [IQR]	46.00 ±13.84 45.00 [37.00, 53.00]	46.40 ±14.19 45.00 [38.00, 54.00]	0.028	46.02 ±13.85 45.00 [37.00, 53.00]	46.20 ±14.10 45.00 [37.00, 54.00]	0.013
Total cholesterol (mg/dl)*; mean (SD) median [IQR]	165.41 ±49.48 161.00 [135.00, 192.00]	163.48 ±48.54 159.00 [133.00, 190.00]	0.039	165.35 ±49.50 161.00 [135.00, 192.00]	165.31 ±49.04 161.00 [135.00, 193.00]	0.001
Triglyceride (mg/dl)*; mean (SD) median [IQR]	176.03 ±150.57 144.00 [104.00, 205.00]	172.38 ±147.65 143.00 [103.00, 202.00]	0.024	176.00 ±150.72 144.00 [104.00, 205.00]	175.09 ±154.99 144.00 [103.00, 204.00]	0.006

**Burden of comorbidities**						
Combined comorbidity score; mean (SD) median [IQR]	1.79 ±2.26 1.00 [0.00, 3.00]	2.00 ±2.40 1.00 [0.00, 3.00]	0.092	1.79 ±2.27 1.00 [0.00, 3.00]	1.80 ±2.26 1.00 [0.00, 3.00]	0.005
Frailty Score; mean (SD) median [IQR]	0.16 ±0.05 0.15 [0.13, 0.19]	0.17 ±0.06 0.16 [0.13, 0.19]	0.082	0.16 ±0.05 0.15 [0.13, 0.19]	0.16 ±0.05 0.15 [0.13, 0.19]	0.002
**Baseline hospitalizations and hospital metrics**						
Number of Hospitalizations; mean (SD) median [IQR]	0.16 ±0.57 0.00 [0.00, 0.00]	0.20 ±0.68 0.00 [0.00, 0.00]	0.058	0.16 ±0.57 0.00 [0.00, 0.00]	0.16 ±0.59 0.00 [0.00, 0.00]	0.003
Any hospitalization within prior 91 days	2,136 (3.5%)	5,302 (4.3%)	0.040	2,133 (3.5%)	2,125 (3.5%)	0.001
Any hospitalization within prior 92-365 days	5,278 (8.6%)	12,422 (10.0%)	0.046	5,269 (8.7%)	5,301 (8.7%)	0.002
Number of hospitalizations (0, 1, 2 or more)						
< 1	54,457 (89.1%)	108,767 (87.4%)	0.055	54,225 (89.1%)	54,180 (89.0%)	0.002
1 - <2	4,723 (7.7%)	10,816 (8.7%)	0.035	4,715 (7.7%)	4,760 (7.8%)	0.003
>= 2	1,909 (3.1%)	4,881 (3.9%)	0.043	1,906 (3.1%)	1,906 (3.1%)	0.000
Heart failure hospitalization	1,728 (2.8%)	4,506 (3.6%)	0.045	1,727 (2.8%)	1,767 (2.9%)	0.004
ED visit	16,007 (26.2%)	36,192 (29.1%)	0.064	15,977 (26.3%)	16,047 (26.4%)	0.003
**Calendar year of cohort entry**						
2022	5,375 (8.8%)	19,393 (15.6%)	0.208	5,375 (8.8%)	5,161 (8.5%)	0.013
2023	24,848 (40.7%)	51,513 (41.4%)	0.014	24,834 (40.8%)	24,917 (41.0%)	0.003
2024	29,475 (48.2%)	52,182 (41.9%)	0.127	29,382 (48.3%)	29,553 (48.6%)	0.006
2025	1,391 (2.3%)	1,376 (1.1%)	0.091	1,255 (2.1%)	1,215 (2.0%)	0.005

Abbreviations: *not used in the propensity score; ACE, angiotensin-converting enzyme inhibitors; ARB, angiotensin receptor blocker; ARNI, angiotensin receptor/neprilysin inhibitor; BMI, body mass index; CABG, coronary artery bypass graft surgery; CED, cohort entry date; CKD, chronic kidney disease; COPD, chronic obstructive pulmonary disease; DKA, diabetic ketoacidosis; DPP4i, dipeptidyl peptidase-4 inhibitors; ED, emergency department; eGFR, estimated glomerular filtration rate (estimated using the quadratic GFR equation: GFR = EXP(1.911 + (5.249 / Serum creatinine) - (2.114 / (Serum creatinine^2)) - (0.00686 * Age) - 0.205 (if female))); HbA1c, hemoglobin A1c; HDL, high-density lipoprotein cholesterol; HONK, hyperglycemic hyperosmolar nonketotic state; IQR, inter-quartile-range; MASH, metabolic dysfunction associated steatohepatitis; MASLD, metabolic dysfunction associated steatotic liver disease; MI, myocardial infarction; N, number of participants; NSAIDs, non-steroidal anti-inflammatory drugs; PCSK9, proprotein convertase subtilisin/kexin type 9; PSA, prostate-specific antigen; PTCA, percutaneous transluminal coronary angioplasty; SGLT2i, sodium-glucose transport protein 2 inhibitors; SD, standard deviation; SMD, standardized mean difference; TIA, transient ischemic attack.

Missing data were handled by assuming absence of a code indicated absence of the condition for most binary covariates. Missing indicators were included for race and region in the propensity score model.

Laboratory values were only available in a subset of the Optum database and were truncated using clinically plausible cut-off values (BMI values >100 or <10 were set to missing; missingness before matching ~53%. Creatinine values >30 were set to missing, and values <0.8 were set to 0.8; missingness before matching ~40%. eGFR values >150 were set to 150, and values =0 were set to missing; missingness before matching ~51%. Glucose values <30 were set to missing; missingness before matching ~40%. HbA1c values ≥20 or ≤2 were set to missing; missingness before matching ~34%. HDL value missingness before matching was ~47%. Heart rate values <30 were set to missing; missingness before matching ~66%. LDL value missingness before matching was~45%. Systolic blood pressure values <30 were set to missing; missingness before matching ~50%. Total cholesterol value missingness before matching was ~47%. Triglyceride value missingness before matching was ~48%).

The Race-Others category includes Asian and Hispanic individuals.

Supplementary Table 18. Baseline characteristics of initiators of tirzepatide vs semaglutide when applying expanded eligibility criteria before and after propensity score matching. MarketScan database. Values are number (percentage) unless otherwise specified.

	Before propensity score matching			After propensity score matching		
Variable	Tirzepatide (n = 25,357)	Semaglutide (n = 68,932)	SMD	Tirzepatide (n = 25,345)	Semaglutide (n = 25,345)	SMD
Demographics						
Age	53.78 ±9.96 54.00 [48.00, 60.00]	54.46 ±10.20 55.00 [48.00, 61.00]	0.007	53.78 ±9.96 54.00 [48.00, 60.00]	53.75 ±10.10 54.00 [47.00, 60.00]	0.003
Gender						
Male	11,243 (44.3%)	30,788 (44.7%)	0.007	11,236 (44.3%)	11,202 (44.2%)	0.003
Female	14,114 (55,7%)	38,144 (55,3%)	0.007	14,109 (55,7%)	14,143 (55,8%)	0.003
Region / State						
Northeast	2,193 (8.6%)	7,701 (11.2%)	0.085	2,193 (8.7%)	2,152 (8.5%)	0.006
Midwest / North central	5,568 (22.0%)	15,159 (22.0%)	0.001	5,565 (22.0%)	5,568 (22.0%)	0.000
South	15,680 (61.8%)	39,482 (57.3%)	0.093	15,671 (61.8%)	15,643 (61.7%)	0.002
West	1,894 (7.5%)	6,518 (9.5%)	0.071	1,894 (7.5%)	1,960 (7.7%)	0.010
Missing	22 (0.1%)	72 (0.1%)	0.006	22 (0.1%)	22 (0.1%)	0.000
Lifestyle risk factors						
Smoking / Tobacco use	2,319 (9.1%)	6,749 (9.8%)	0.022	2,319 (9.1%)	2,268 (8.9%)	0.007
Weight						
Overweight (25.0-29.9)	1,971 (7.8%)	6,016 (8.7%)	0.035	1,970 (7.8%)	1,977 (7.8%)	0.001
Class 1 Obesity (30.0-34.9)	1,990 (7.8%)	5,603 (8.1%)	0.010	1,987 (7.8%)	1,970 (7.8%)	0.003
Class 2 Obesity (35.0-39.9)	1,759 (6.9%)	4,609 (6.7%)	0.010	1,759 (6.9%)	1,719 (6.8%)	0.006
Class 3 Obesity (40.0 and above)	5,522 (21.8%)	15,995 (23.2%)	0.034	5,519 (21.8%)	5,605 (22.1%)	0.008
Unspecified Obesity	14,115 (55.7%)	36,709 (53.3%)	0.048	14,110 (55.7%)	14,074 (55.5%)	0.003
Diabetes complications						
Diabetic retinopathy	1,336 (5.3%)	3,616 (5.2%)	0.001	1,336 (5.3%)	1,325 (5.2%)	0.002
Diabetic neuropathy	3,341 (13.2%)	9,334 (13.5%)	0.011	3,338 (13.2%)	3,381 (13.3%)	0.005
Diabetic nephropathy	2,546 (10.0%)	7,218 (10.5%)	0.014	2,545 (10.0%)	2,556 (10.1%)	0.001
Diabetes with other opthalmic complications	319 (1.3%)	994 (1.4%)	0.016	319 (1.3%)	323 (1.3%)	0.001
Diabetes with peripheral circulatory disorders	1,751 (6.9%)	4,788 (6.9%)	0.002	1,749 (6.9%)	1,770 (7.0%)	0.003
Diabetic foot	329 (1.3%)	976 (1.4%)	0.010	329 (1.3%)	311 (1.2%)	0.006
Erectile dysfunction	862 (3.4%)	2,494 (3.6%)	0.012	861 (3.4%)	909 (3.6%)	0.010
Hypoglycemia	4,600 (18.1%)	11,838 (17.2%)	0.025	4,595 (18.1%)	4,602 (18.2%)	0.001
Hyperglycemia / DKA / HONK	12,302 (48.5%)	32,329 (46.9%)	0.032	12,293 (48.5%)	12,277 (48.4%)	0.001
Skin infections	2,137 (8.4%)	5,718 (8.3%)	0.005	2,136 (8.4%)	2,119 (8.4%)	0.002
Cardiovascular-related conditions						
Coronary atherosclerosis	2,452 (9.7%)	7,620 (11.1%)	0.045	2,451 (9.7%)	2,489 (9.8%)	0.005
Stable angina	449 (1.8%)	1,511 (2.2%)	0.030	448 (1.8%)	465 (1.8%)	0.005

Unstable angina	288 (1.1%)	1,008 (1.5%)	0.029	288 (1.1%)	283 (1.1%)	0.002
Hypertension	20,363 (80.3%)	55,033 (79.8%)	0.012	20,351 (80.3%)	20,340 (80.3%)	0.001
Hypotension	267 (1.1%)	881 (1.3%)	0.021	266 (1.0%)	251 (1.0%)	0.006
Hyperlipidemia	19,849 (78.3%)	53,688 (77.9%)	0.009	19,838 (78.3%)	19,893 (78.5%)	0.005
Acute MI	182 (0.7%)	593 (0.9%)	0.016	181 (0.7%)	181 (0.7%)	0.000
Old MI	280 (1.1%)	1,038 (1.5%)	0.035	279 (1.1%)	296 (1.2%)	0.006
Ischemic stroke	123 (0.5%)	480 (0.7%)	0.028	123 (0.5%)	130 (0.5%)	0.004
TIA	227 (0.9%)	711 (1.0%)	0.014	227 (0.9%)	232 (0.9%)	0.002
Cardiac conduction disorder	603 (2.4%)	1,885 (2.7%)	0.023	603 (2.4%)	605 (2.4%)	0.001
Previous cardiac procedure (CABG, PTCA, Stent)	229 (0.9%)	766 (1.1%)	0.021	229 (0.9%)	234 (0.9%)	0.002
PVD diagnosis or surgery	841 (3.3%)	2,303 (3.3%)	0.001	841 (3.3%)	804 (3.2%)	0.008
Atrial fibrillation	1,109 (4.4%)	3,326 (4.8%)	0.022	1,108 (4.4%)	1,088 (4.3%)	0.004
Other cardiac dysrhythmia	3,341 (13.2%)	9,532 (13.8%)	0.019	3,339 (13.2%)	3,391 (13.4%)	0.006
Heart failure	1,182 (4.7%)	3,822 (5.5%)	0.040	1,180 (4.7%)	1,190 (4.7%)	0.002
Acute heart failure	248 (1.0%)	970 (1.4%)	0.040	246 (1.0%)	236 (0.9%)	0.004
Cardiomyopathy	598 (2.4%)	1,897 (2.8%)	0.025	598 (2.4%)	587 (2.3%)	0.003
Valve disorders	1,292 (5.1%)	3,831 (5.6%)	0.021	1,292 (5.1%)	1,280 (5.1%)	0.002
Valve replacement	85 (0.3%)	326 (0.5%)	0.022	85 (0.3%)	78 (0.3%)	0.005
Edema	1,771 (7.0%)	4,973 (7.2%)	0.009	1,771 (7.0%)	1,769 (7.0%)	0.000
Venous thromboembolism / Pulmonary embolism	504 (2.0%)	1,483 (2.2%)	0.012	504 (2.0%)	498 (2.0%)	0.002
Pulmonary hypertension	238 (0.9%)	754 (1.1%)	0.015	238 (0.9%)	242 (1.0%)	0.002
Implantable cardioverter defibrillator	33 (0.1%)	115 (0.2%)	0.010	33 (0.1%)	34 (0.1%)	0.001
Hyperkalemia	251 (1.0%)	887 (1.3%)	0.028	251 (1.0%)	224 (0.9%)	0.011
Cerebrovascular procedure	9 (0.0%)	31 (0.0%)	0.005	9 (0.0%)	11 (0.0%)	0.004
Insertion of pacemakers / removal of cardiac lead	9 (0.0%)	29 (0.0%)	0.003	9 (0.0%)	15 (0.1%)	0.011
Renal-related conditions						
Hypertensive nephropathy	1,039 (4.1%)	3,012 (4.4%)	0.014	1,038 (4.1%)	1,052 (4.2%)	0.003
CKD Stage 1-2	521 (2.1%)	1,349 (2.0%)	0.007	520 (2.1%)	531 (2.1%)	0.003
CKD Stage 3-4	1,069 (4.2%)	3,386 (4.9%)	0.033	1,068 (4.2%)	1,025 (4.0%)	0.009
Unspecified CKD	367 (1.4%)	1,206 (1.7%)	0.024	366 (1.4%)	374 (1.5%)	0.003
Microalbuminuria or proteinuria	825 (3.3%)	2,327 (3.4%)	0.007	825 (3.3%)	810 (3.2%)	0.003
Acute kidney injury	604 (2.4%)	1,823 (2.6%)	0.017	603 (2.4%)	603 (2.4%)	0.000
Urinary tract infections	2,374 (9.4%)	6,196 (9.0%)	0.013	2,373 (9.4%)	2,366 (9.3%)	0.001
Genital infections	925 (3.6%)	2,619 (3.8%)	0.008	925 (3.6%)	944 (3.7%)	0.004
Urolithiasis (Kidney and urinary stone)	1,007 (4.0%)	2,744 (4.0%)	0.000	1,006 (4.0%)	995 (3.9%)	0.002
Other comorbidities						
COPD	930 (3.7%)	2,880 (4.2%)	0.026	930 (3.7%)	933 (3.7%)	0.001
Asthma	2,362 (9.3%)	6,552 (9.5%)	0.007	2,361 (9.3%)	2,385 (9.4%)	0.003
Obstructive sleep apnea	7,581 (29.9%)	20,051 (29.1%)	0.018	7,575 (29.9%)	7,662 (30.2%)	0.007

Serious bacterial infections	523 (2.1%)	1,540 (2.2%)	0.012	521 (2.1%)	526 (2.1%)	0.001
Pneumonia	645 (2.5%)	1,953 (2.8%)	0.018	644 (2.5%)	632 (2.5%)	0.003
Liver disease	3,508 (13.8%)	9,460 (13.7%)	0.003	3,503 (13.8%)	3,479 (13.7%)	0.003
MASH / MASLD	2,373 (9.4%)	6,419 (9.3%)	0.002	2,371 (9.4%)	2,332 (9.2%)	0.005
Fractures / Falls	714 (2.8%)	2,051 (3.0%)	0.010	714 (2.8%)	762 (3.0%)	0.011
Osteoporosis	289 (1.1%)	879 (1.3%)	0.012	289 (1.1%)	309 (1.2%)	0.007
Osteoarthritis	4,815 (19.0%)	13,474 (19.5%)	0.014	4,812 (19.0%)	4,803 (19.0%)	0.001
Depression	3,957 (15.6%)	11,039 (16.0%)	0.011	3,954 (15.6%)	3,963 (15.6%)	0.001
Dementia	153 (0.6%)	587 (0.9%)	0.029	153 (0.6%)	146 (0.6%)	0.004
Delirium or psychosis	94 (0.4%)	380 (0.6%)	0.027	94 (0.4%)	95 (0.4%)	0.001
Anxiety	5,316 (21.0%)	14,298 (20.7%)	0.005	5,315 (21.0%)	5,245 (20.7%)	0.007
Sleep disorders	6,375 (25.1%)	16,638 (24.1%)	0.023	6,367 (25.1%)	6,438 (25.4%)	0.006
Anemia	3,494 (13.8%)	9,429 (13.7%)	0.003	3,492 (13.8%)	3,519 (13.9%)	0.003
Influenza	641 (2.5%)	1,387 (2.0%)	0.035	640 (2.5%)	700 (2.8%)	0.015
COVID	3,324 (13.1%)	9,821 (14.2%)	0.033	3,322 (13.1%)	3,379 (13.3%)	0.007
Hyperthyroidism and other thyroid gland disorders	5,634 (22.2%)	14,374 (20.9%)	0.033	5,629 (22.2%)	5,625 (22.2%)	0.000
Hypothyroidism	4,388 (17.3%)	11,121 (16.1%)	0.031	4,385 (17.3%)	4,385 (17.3%)	0.000
Nephrotic syndrome	9 (0.0%)	38 (0.1%)	0.009	9 (0.0%)	6 (0.0%)	0.007
Urinary incontinence	651 (2.6%)	1,876 (2.7%)	0.01	651 (2.6%)	659 (2.6%)	0.002
Biliary disease	60 (0.2%)	153 (0.2%)	0.003	58 (0.2%)	53 (0.2%)	0.004
Pancreatitis	27 (0.1%)	74 (0.1%)	0.000	27 (0.1%)	37 (0.1%)	0.011
Bowel obstruction	34 (0.1%)	88 (0.1%)	0.002	34 (0.1%)	34 (0.1%)	0.000
Gastroparesis	137 (0.5%)	325 (0.5%)	0.01	136 (0.5%)	152 (0.6%)	0.008
Diabetes medications						
Number of antidiabetic drugs on CED, mean (SD)	2.06 ±0.98 2.00 [1.00, 3.00]	2.07 ±0.97 2.00 [1.00, 3.00]	0.017	2.06 ±0.98 2.00 [1.00, 3.00]	2.05 ±0.98 2.00 [1.00, 3.00]	0.003
Concomitant use or initiation of Metformin	12,709 (50.1%)	36,250 (52.6%)	0.049	12,705 (50.1%)	12,707 (50.1%)	0.000
Concomitant use or initiation of Insulins	3,420 (13.5%)	9,131 (13.2%)	0.007	3,416 (13.5%)	3,395 (13.4%)	0.002
Concomitant use or initiation of Sulfonylureas	2,805 (11.1%)	9,014 (13.1%)	0.062	2,804 (11.1%)	2,830 (11.2%)	0.003
Concomitant use or initiation of DPP-4i	1,630 (6.4%)	4,614 (6.7%)	0.011	1,630 (6.4%)	1,606 (6.3%)	0.004
Concomitant use or initiation of SGLT-2i	5,234 (20.6%)	12,489 (18.1%)	0.064	5,230 (20.6%)	5,214 (20.6%)	0.002
Concomitant use or initiation of Any other glucose-lowering drugs	1,000 (3.9%)	2,525 (3.7%)	0.015	1,000 (3.9%)	958 (3.8%)	0.009
Past use of Metformin	17,285 (68.2%)	48,294 (70.1%)	0.041	17,279 (68.2%)	17,240 (68.0%)	0.003
Past use of Insulins	4,794 (18.9%)	12,548 (18.2%)	0.018	4,789 (18.9%)	4,768 (18.8%)	0.002
Past use of Sulfonylureas	4,197 (16.6%)	12,888 (18.7%)	0.056	4,196 (16.6%)	4,221 (16.7%)	0.003
Past use of DPP-4i	2,680 (10.6%)	7,640 (11.1%)	0.017	2,680 (10.6%)	2,592 (10.2%)	0.011
Past use of SGLT-2i	6,902 (27.2%)	16,536 (24.0%)	0.074	6,895 (27.2%)	6,884 (27.2%)	0.001
Past use of Any other glucose-lowering drugs	1,450 (5.7%)	3,705 (5.4%)	0.015	1,449 (5.7%)	1,397 (5.5%)	0.009
Other medications						
ACE / ARB	17,598 (69.4%)	47,681 (69.2%)	0.005	17,591 (69.4%)	17,609 (69.5%)	0.002

ARNI	291 (1.1%)	873 (1.3%)	0.011	290 (1.1%)	287 (1.1%)	0.001
Thiazides	8,888 (35.1%)	24,036 (34.9%)	0.004	8,885 (35.1%)	8,900 (35.1%)	0.001
Beta-blockers	7,988 (31.5%)	22,154 (32.1%)	0.014	7,983 (31.5%)	8,015 (31.6%)	0.003
Calcium channel blockers	6,861 (27.1%)	19,450 (28.2%)	0.026	6,858 (27.1%)	6,884 (27.2%)	0.002
Digoxin / Digitoxin	75 (0.3%)	205 (0.3%)	0.000	75 (0.3%)	74 (0.3%)	0.001
Loop diuretics	2,283 (9.0%)	6,619 (9.6%)	0.021	2,281 (9.0%)	2,257 (8.9%)	0.003
Other diuretics	1,887 (7.4%)	5,294 (7.7%)	0.009	1,885 (7.4%)	1,930 (7.6%)	0.007
Intravenous diuretics	119 (0.5%)	421 (0.6%)	0.019	119 (0.5%)	121 (0.5%)	0.001
Nitrates	766 (3.0%)	2,462 (3.6%)	0.031	765 (3.0%)	761 (3.0%)	0.001
Anti-arrhythmics	333 (1.3%)	989 (1.4%)	0.010	333 (1.3%)	311 (1.2%)	0.008
Statins	17,918 (70.7%)	50,078 (72.6%)	0.044	17,909 (70.7%)	17,859 (70.5%)	0.004
PCSK9 inhibitors and other lipid-lowering drugs	3,030 (11.9%)	8,074 (11.7%)	0.007	3,028 (11.9%)	3,059 (12.1%)	0.004
Antiplatelet medications	1,767 (7.0%)	5,332 (7.7%)	0.029	1,765 (7.0%)	1,764 (7.0%)	0.000
Oral anticoagulants	1,461 (5.8%)	4,225 (6.1%)	0.016	1,459 (5.8%)	1,424 (5.6%)	0.006
COPD / Asthma medications	8,474 (33.4%)	22,894 (33.2%)	0.004	8,467 (33.4%)	8,528 (33.6%)	0.005
NSAIDS	8,929 (35.2%)	23,869 (34.6%)	0.012	8,924 (35.2%)	8,881 (35.0%)	0.004
Oral corticosteroids	7,236 (28.5%)	19,020 (27.6%)	0.021	7,231 (28.5%)	7,265 (28.7%)	0.003
Osteoporosis agents (incl. bisphosphonates)	329 (1.3%)	903 (1.3%)	0.001	329 (1.3%)	329 (1.3%)	0.000
Opioids	6,394 (25.2%)	17,125 (24.8%)	0.009	6,389 (25.2%)	6,370 (25.1%)	0.002
Anti-depressants	9,373 (37.0%)	24,872 (36.1%)	0.018	9,368 (37.0%)	9,375 (37.0%)	0.001
Antipsychotics	755 (3.0%)	2,092 (3.0%)	0.003	755 (3.0%)	749 (3.0%)	0.001
Anxiolytics / hypnotics, benzos	5,358 (21.1%)	13,643 (19.8%)	0.033	5,353 (21.1%)	5,338 (21.1%)	0.001
Dementia medications	60 (0.2%)	258 (0.4%)	0.025	60 (0.2%)	58 (0.2%)	0.002
Urinary tract infections antibiotics	11,847 (46.7%)	30,518 (44.3%)	0.049	11,838 (46.7%)	11,814 (46.6%)	0.002
Laxatives	769 (3.0%)	2,126 (3.1%)	0.003	769 (3.0%)	806 (3.2%)	0.008
**Healthcare utilization marker**						
Number of distinct medications; mean (SD) median [IQR]	13.48 ±6.58 12.00 [9.00, 17.00]	13.21 ±6.43 12.00 [9.00, 17.00]	0.042	13.48 ±6.58 12.00 [9.00, 17.00]	13.51 ±6.70 12.00 [9.00, 17.00]	0.005
Number of office visits; mean (SD) median [IQR]	8.35 ±6.21 7.00 [4.00, 11.00]	8.35 ±6.32 7.00 [4.00, 11.00]	0.000	8.35 ±6.20 7.00 [4.00, 11.00]	8.35 ±6.30 7.00 [4.00, 11.00]	0.001
Number of endocrinologist visits; mean (SD) median [IQR]	0.39 ±1.24 0.00 [0.00, 0.00]	0.33 ±1.14 0.00 [0.00, 0.00]	0.047	0.38 ±1.17 0.00 [0.00, 0.00]	0.38 ±1.23 0.00 [0.00, 0.00]	0.004
Number of cardiologist visits; mean (SD) median [IQR]	0.83 ±2.20 0.00 [0.00, 1.00]	0.92 ±2.53 0.00 [0.00, 1.00]	0.038	0.83 ±2.19 0.00 [0.00, 1.00]	0.82 ±2.33 0.00 [0.00, 1.00]	0.004
Number of internal / family medicine visits; mean (SD) median [IQR]	4.55 ±5.45 3.00 [1.00, 6.00]	4.57 ±5.14 3.00 [1.00, 6.00]	0.004	4.55 ±5.45 3.00 [1.00, 6.00]	4.51 ±5.11 3.00 [1.00, 6.00]	0.007
Number of electrocardiograms (ECG/EKG) ; mean (SD) median [IQR]	0.88 ±2.58 0.00 [0.00, 1.00]	0.99 ±2.93 0.00 [0.00, 1.00]	0.042	0.88 ±2.56 0.00 [0.00, 1.00]	0.88 ±2.33 0.00 [0.00, 1.00]	0.003
Number of echocardiograms; mean (SD) median [IQR]	0.30 ±1.45 0.00 [0.00, 0.00]	0.35 ±1.83 0.00 [0.00, 0.00]	0.034	0.29 ±1.43 0.00 [0.00, 0.00]	0.30 ±1.53 0.00 [0.00, 0.00]	0.006
Out-of-pocket medication cost; mean (SD) median [IQR]	561.98 ±1,041.69 311.20 [114.12, 701.32]	492.30 ±905.36 287.51 [108.25, 618.62]	0.071	556.10 ±916.25 310.80 [114.06, 700.00]	546.71 ±1,000.80 305.58 [113.03, 665.64]	0.010
Unique brand medicines; mean (SD) median [IQR]	13.70 ±6.76 12.00 [9.00, 17.00]	13.47 ±6.61 12.00 [9.00, 17.00]	0.035	13.70 ±6.75 12.00 [9.00, 17.00]	13.72 ±6.87 12.00 [9.00, 17.00]	0.004
Unique generic medicines; mean (SD) median [IQR]	13.48 ±6.58 12.00 [9.00, 17.00]	13.21 ±6.43 12.00 [9.00, 17.00]	0.042	13.48 ±6.58 12.00 [9.00, 17.00]	13.51 ±6.70 12.00 [9.00, 17.00]	0.005

Ratio of brand to generic medications; mean (SD) median [IQR]	1.01 ±0.04 1.00 [1.00, 1.00]	1.02 ±0.04 1.00 [1.00, 1.00]	0.092	1.01 ±0.04 1.00 [1.00, 1.00]	1.01 ±0.04 1.00 [1.00, 1.00]	0.008
**Healthy behavior markers**						
Colonoscopy / Sigmoidoscopy	2,748 (10.8%)	7,654 (11.1%)	0.009	2,747 (10.8%)	2,693 (10.6%)	0.007
Flu Pneumococcal vaccine	6,589 (26.0%)	19,111 (27.7%)	0.039	6,587 (26.0%)	6,658 (26.3%)	0.006
Pap smear	3,301 (13.0%)	8,443 (12.2%)	0.023	3,300 (13.0%)	3,357 (13.2%)	0.007
PSA test	5,280 (20.8%)	14,120 (20.5%)	0.008	5,277 (20.8%)	5,296 (20.9%)	0.002
Fecal occult blood test	867 (3.4%)	2,496 (3.6%)	0.011	867 (3.4%)	834 (3.3%)	0.007
Bone mineral density tests	865 (3.4%)	2,335 (3.4%)	0.001	864 (3.4%)	842 (3.3%)	0.005
Mammograms	6,996 (27.6%)	18,887 (27.4%)	0.004	6,994 (27.6%)	6,938 (27.4%)	0.005
Telemedicine	7,446 (29.4%)	22,031 (32.0%)	0.056	7,444 (29.4%)	7,519 (29.7%)	0.006
**Laboratory and diagnostic tests**						
HbA1c tests; mean (SD) median [IQR]	2.18 ±1.43 2.00 [1.00, 3.00]	2.13 ±1.47 2.00 [1.00, 3.00]	0.034	2.18 ±1.43 2.00 [1.00, 3.00]	2.17 ±1.36 2.00 [1.00, 3.00]	0.012
Lipid panels; mean (SD) median [IQR]	1.59 ±1.18 1.00 [1.00, 2.00]	1.54 ±1.27 1.00 [1.00, 2.00]	0.040	1.59 ±1.18 1.00 [1.00, 2.00]	1.59 ±1.16 1.00 [1.00, 2.00]	0.000
Creatinine tests; mean (SD) median [IQR]	0.03 ±0.24 0.00 [0.00, 0.00]	0.04 ±0.40 0.00 [0.00, 0.00]	0.033	0.03 ±0.24 0.00 [0.00, 0.00]	0.03 ±0.24 0.00 [0.00, 0.00]	0.002
Natriuretic peptide tests; mean (SD) median [IQR]	0.10 ±0.60 0.00 [0.00, 0.00]	0.11 ±0.77 0.00 [0.00, 0.00]	0.027	0.10 ±0.60 0.00 [0.00, 0.00]	0.10 ±0.74 0.00 [0.00, 0.00]	0.008
Urine tests; mean (SD) median [IQR]	0.89 ±1.57 0.00 [0.00, 1.00]	0.90 ±1.60 0.00 [0.00, 1.00]	0.003	0.89 ±1.57 0.00 [0.00, 1.00]	0.90 ±1.56 0.00 [0.00, 1.00]	0.004
**Burden of comorbidities**						
Combined comorbidity score; mean (SD) median [IQR]	0.92 ±1.57 1.00 [0.00, 2.00]	0.98 ±1.67 1.00 [0.00, 2.00]	0.037	0.92 ±1.57 1.00 [0.00, 2.00]	0.93 ±1.61 1.00 [0.00, 2.00]	0.000
Frailty Score; mean (SD) median [IQR]	0.14 ±0.04 0.13 [0.12, 0.16]	0.14 ±0.04 0.13 [0.12, 0.16]	0.030	0.14 ±0.04 0.13 [0.12, 0.16]	0.14 ±0.04 0.13 [0.12, 0.16]	0.000
**Baseline hospitalizations and hospital metrics**						
Number of Hospitalizations; mean (SD) median [IQR]	3.77 ±20.18 0.00 [0.00, 0.00]	4.49 ±26.85 0.00 [0.00, 0.00]	0.030	3.75 ±19.95 0.00 [0.00, 0.00]	3.91 ±31.15 0.00 [0.00, 0.00]	0.006
Any hospitalization within prior 91 days	578 (2.3%)	1,844 (2.7%)	0.025	577 (2.3%)	590 (2.3%)	0.003
Any hospitalization within prior 92-365 days	1,299 (5.1%)	4,068 (5.9%)	0.034	1,297 (5.1%)	1,289 (5.1%)	0.001
Number of hospitalizations (0, 1, 2 or more)						
< 1	23,628 (93.2%)	63,460 (92.1%)	0.043	23,618 (93.2%)	23,612 (93.2%)	0.001
1 - <2	138 (0.5%)	444 (0.6%)	0.013	138 (0.5%)	138 (0.5%)	0.000
>= 2	1,591 (6.3%)	5,028 (7.3%)	0.041	1,589 (6.3%)	1,595 (6.3%)	0.001
Heart failure hospitalization	325 (1.3%)	1,204 (1.7%)	0.038	324 (1.3%)	333 (1.3%)	0.003
ED visit	6,921 (27.3%)	19,883 (28.8%)	0.035	6,917 (27.3%)	6,992 (27.6%)	0.007
**Calendar year of cohort entry**						
2022	4,655 (18.4%)	19,412 (28.2%)	0.234	4,654 (18.4%)	4,642 (18.3%)	0.001
2023	20,702 (81.6%)	49,520 (71.8%)	0.234	20,691 (81.6%)	20,703 (81.7%)	0.001

Abbreviations: *not used in the propensity score; ACE, angiotensin-converting enzyme inhibitors; ARB, angiotensin receptor blocker; ARNI, angiotensin receptor/neprilysin inhibitor; BMI, body mass index; CABG, coronary artery bypass graft surgery; CED, cohort entry date; CKD, chronic kidney disease; COPD, chronic obstructive pulmonary disease; DKA, diabetic ketoacidosis; DPP4i, dipeptidyl peptidase-4 inhibitors; ED, emergency department; eGFR, estimated glomerular filtration rate (estimated using the quadratic GFR equation: GFR = EXP(1.911 + (5.249 / Serum creatinine) - (2.114 / (Serum creatinine^2)) - (0.00686 * Age) - 0.205 (if female))); HbA1c, hemoglobin A1c; HDL, high-density lipoprotein cholesterol; HONK, hyperglycemic hyperosmolar nonketotic state; IQR, inter-quartile-range; MASH, metabolic dysfunction associated

steatohepatitis; MASLD, metabolic dysfunction associated steatotic liver disease; MI, myocardial infarction; N, number of participants; NSAIDs, non-steroidal anti-inflammatory drugs; PCSK9, proprotein convertase subtilisin/kexin type 9; PSA, prostate-specific antigen; PTCA, percutaneous transluminal coronary angioplasty; SGLT2i, sodium-glucose transport protein 2 inhibitors; SD, standard deviation; SMD, standardized mean difference; TIA, transient ischemic attack.
Missing data were handled by assuming absence of a code indicated absence of the condition for most binary covariates. Missing indicators were included for race and region in the propensity score model.

Supplementary Table 19. Primary end point of all-cause mortality, myocardial infarction or stroke among individuals initiating (A) semaglutide vs sitagliptin, (B) tirzepatide vs dulaglutide, or (C) tirzepatide vs semaglutide, reported by eligibility criteria applied and end point.

(A) Semaglutide vs sitagliptin in cohort meeting trial eligibility criteria, end point: 3P-MACE			
	1–year rate, % (95% CI)		
Database	Semaglutide	Sitagliptin	HR (95% CI)
Pooled across databases	3.03 (2.68 to 3.38)	4.35 (3.96 to 4.74)	0.68 (0.60 to 0.77)
Optum	2.93 (2.48 to 3.45)	4.38 (3.88 to 4.93)	0.61 (0.51 to 0.73)
Marketscan	1.74 (1.35 to 2.24)	2.41 (1.89 to 3.06)	0.80 (0.58 to 1.10)
Medicare	4.47 (3.66 to 5.45)	6.16 (5.27 to 7.19)	0.73 (0.59 to 0.90)
(A) Semaglutide vs sitagliptin in cohort meeting expanded eligibility criteria, end point: 3P-MACE and 2P-MACE			
	1–year rate, % (95% CI)		
Database	Semaglutide	Sitagliptin	HR (95% CI)
3P-MACE			
Pooled across databases	*2.15 (1.99 to 2.31)*	*2.90 (2.72 to 3.07)*	*0.71 (0.65 to 0.77)*
Optum	*2.38 (2.14 to 2.65)*	*3.67 (3.38 to 3.98)*	*0.64 (0.57 to 0.72)*
Marketscan	*0.93 (0.80 to 1.09)*	*1.17 (1.01 to 1.35)*	*0.79 (0.65 to 0.96)*
Medicare	*4.74 (4.12 to 5.44)*	*5.38 (4.79 to 6.03)*	*0.78 (0.67 to 0.91)*
2P-MACE			
Pooled across databases	1.49 (1.36 to 1.62)	1.74 (1.60 to 1.88)	0.82 (0.74 to 0.91)
Optum	1.55 (1.36 to 1.77)	1.99 (1.78 to 2.22)	0.78 (0.67 to 0.90)
Marketscan	0.81 (0.69 to 0.96)	0.94 (0.80 to 1.10)	0.84 (0.68 to 1.03)
Medicare	3.13 (2.64 to 3.73)	3.24 (2.78 to 3.77)	0.88 (0.72 to 1.07)
(B) Tirzepatide vs dulaglutide in cohort meeting trial eligibility criteria, end point: 3P-MACE			
	1–year rate, % (95% CI)		
Database	Tirzepatide	Dulaglutide	HR (95% CI)
Pooled across databases	2.84 (2.40 to 3.27)	3.53 (2.99 to 4.06)	0.83 (0.69 to 1.01)
Optum	3.13 (2.65 to 3.70)	4.06 (3.45 to 4.78)	0.79 (0.64 to 0.97)
Marketscan	2.02 (1.38 to 2.95)	1.99 (1.33 to 2.97)	1.08 (0.68 to 1.74)
(B) Tirzepatide vs dulaglutide in cohort meeting expanded eligibility criteria, end point: 3P-MACE			

	1–year rate, % (95% CI)		
Database	Tirzepatide	Dulaglutide	HR (95% CI)
Pooled across databases	1.44 (1.28 to 1.61)	1.75 (1.54 to 1.96)	0.87 (0.75 to 1.01)
Optum	1.81 (1.60 to 2.06)	2.20 (1.93 to 2.52)	0.86 (0.73 to 1.01)
Marketscan	0.81 (0.62 to 1.07)	0.98 (0.75 to 1.27)	0.92 (0.67 to 1.28)
(C) Tirzepatide vs semaglutide, end point: 3P-MACE			
	1–year rate, % (95% CI)		
Database	Tirzepatide	Semaglutide	HR (95% CI)
Pooled across databases	1.33 (1.21 to 1.45)	1.32 (1.19 to 1.46)	1.06 (0.95 to 1.18)
Optum	1.60 (1.46 to 1.75)	1.58 (1.42 to 1.75)	1.05 (0.93 to 1.18)
Marketscan	0.70 (0.55 to 0.89)	0.72 (0.54 to 0.95)	1.13 (0.85 to 1.51)

Supplementary Table 20. Censoring reason for the primary end point among individuals initiating (A) semaglutide vs sitagliptin, (B) tirzepatide vs dulaglutide, or (C) tirzepatide vs semaglutide, reported by eligibility criteria applied and database.

(A) Semaglutide vs sitagliptin in cohort meeting trial eligibility criteria, pooled across databases			
	Count (%)		
Censor reason	Overall	Semaglutide	Sitagliptin
Outcome	1,014 (1.9%)	394 (1.5%)	620 (2.3%)
Start of an additional exposure	1,045 (1.9%)	195 (0.7%)	850 (3.1%)
End of index exposure	25,160 (46.5%)	13,555 (50.1%)	11,605 (42.9%)
Maximum follow-up time	10,438 (19.3%)	5,084 (18.8%)	5,354 (19.8%)
Specified date reached	5,726 (10.6%)	2,836 (10.5%)	2,890 (10.7%)
End of patient data	0 (0.0%)	0 (0.0%)	0 (0.0%)
End of patient enrollment	7,024 (13.0%)	3,544 (13.1%)	3,480 (12.9%)
Censor: Other GLP-1-RAs or DPP4i / Nursing home began	3,659 (6.8%)	1,425 (5.3%)	2,234 (8.3%)
(A) Semaglutide vs sitagliptin in cohort meeting trial eligibility criteria, Optum			
	Count (%)		
Censor reason	Overall	Semaglutide	Sitagliptin
Outcome	519 (1.9%)	188 (1.4%)	331 (2.4%)
Start of an additional exposure	534 (2.0%)	85 (0.6%)	449 (3.3%)
End of index exposure	13,811 (50.8%)	7,447 (54.7%)	6,364 (46.8%)
Maximum follow-up time	5,401 (19.9%)	2,503 (18.4%)	2,898 (21.3%)
Specified date reached	176 (0.6%)	130 (1.0%)	46 (0.3%)
End of patient data	0 (0.0%)	0 (0.0%)	0 (0.0%)
End of patient enrollment	4,909 (18.0%)	2,478 (18.2%)	2,431 (17.9%)
Censor: Other GLP-1-RAs or DPP4i / Nursing home began	1,854 (6.8%)	771 (5.7%)	1,083 (8.0%)
(A) Semaglutide vs sitagliptin in cohort meeting trial eligibility criteria, Marketscan			
	Count (%)		
Censor reason	Overall	Semaglutide	Sitagliptin
Outcome	148 (1.1%)	67 (1.0%)	81 (1.2%)
Start of an additional exposure	349 (2.7%)	39 (0.6%)	310 (4.7%)
End of index exposure	5,482 (41.8%)	2,869 (43.8%)	2,613 (39.9%)

Maximum follow-up time	3,229 (24.6%)	1,736 (26.5%)	1,493 (22.8%)
Specified date reached	1,385 (10.6%)	690 (10.5%)	695 (10.6%)
End of patient data	0 (0.0%)	0 (0.0%)	0 (0.0%)
End of patient enrollment	1,772 (13.5%)	899 (13.7%)	873 (13.3%)
Censor: Other GLP-1-RAs or DPP4i / Nursing home began	743 (5.7%)	254 (3.9%)	489 (7.5%)
Outcome	148 (1.1%)	67 (1.0%)	81 (1.2%)
Start of an additional exposure	349 (2.7%)	39 (0.6%)	310 (4.7%)

(A) Semaglutide vs sitagliptin in cohort meeting trial eligibility criteria, Medicare

	Count (%)		
Censor reason	Overall	Semaglutide	Sitagliptin
Outcome	347 (2.5%)	139 (2.0%)	208 (3.0%)
Start of an additional exposure	162 (1.2%)	71 (1.0%)	91 (1.3%)
End of index exposure	5,867 (42.7%)	3,239 (47.1%)	2,628 (38.2%)
Maximum follow-up time	1,808 (13.1%)	845 (12.3%)	963 (14.0%)
Specified date reached	4,165 (30.3%)	2,016 (29.3%)	2,149 (31.2%)
End of patient data	0 (0.0%)	0 (0.0%)	0 (0.0%)
End of patient enrollment	343 (2.5%)	167 (2.4%)	176 (2.6%)
Censor: Other GLP-1-RAs or DPP4i / Nursing home began	1,062 (7.7%)	400 (5.8%)	662 (9.6%)

(A) Semaglutide vs sitagliptin in cohort meeting expanded eligibility criteria, pooled across databases, 3P-MACE and 2P-MACE

	Count (%)		
Censor reason	Overall	Semaglutide	Sitagliptin
3P-MACE			
Outcome	*2,196 (1.4%)*	*888 (1.1%)*	*1,308 (1.6%)*
Start of an additional exposure	*3,540 (2.2%)*	*545 (0.7%)*	*2,995 (3.8%)*
End of index exposure	*73,038 (45.9%)*	*38,376 (48.3%)*	*34,662 (43.6%)*
Maximum follow-up time	*34,624 (21.8%)*	*17,802 (22.4%)*	*16,822 (21.2%)*
Specified date reached	*12,624 (7.9%)*	*6,493 (8.2%)*	*6,131 (7.7%)*
End of patient data	*0 (0.0%)*	*0 (0.0%)*	*0 (0.0%)*
End of patient enrollment	*21,782 (13.7%)*	*11,249 (14.1%)*	*10,533 (13.2%)*
Censor: Other GLP-1-RAs or DPP4i / Nursing home began	*11,198 (7.0%)*	*4,148 (5.2%)*	*7,050 (8.9%)*
2P-MACE			

Outcome	1,434 (0.9%)	635 (0.8%)	799 (1.0%)
Start of an additional exposure	3,540 (2.2%)	545 (0.7%)	2,995 (3.8%)
End of index exposure	73,038 (45.9%)	38,376 (48.3%)	34,662 (43.6%)
Maximum follow-up time	34,624 (21.8%)	17,802 (22.4%)	16,822 (21.2%)
Specified date reached	12,624 (7.9%)	6,493 (8.2%)	6,131 (7.7%)
End of patient data	0 (0.0%)	0 (0.0%)	0 (0.0%)
End of patient enrollment	21,782 (13.7%)	11,249 (14.1%)	10,533 (13.2%)
Censor: Other GLP-1-RAs or DPP4i / Nursing home began	11,198 (7.0%)	4,148 (5.2%)	7,050 (8.9%)
Death	762 (0.5%)	253 (0.3%)	509 (0.6%)
(A) Semaglutide vs sitagliptin in cohort meeting expanded eligibility criteria, Optum, 3P-MACE and 2P-MACE			
	Count (%)		
Censor reason	Overall	Semaglutide	Sitagliptin
3P-MACE			
Outcome	*1,146 (1.7%)*	*432 (1.3%)*	*714 (2.1%)*
Start of an additional exposure	*1,428 (2.1%)*	*195 (0.6%)*	*1,233 (3.6%)*
End of index exposure	*33,406 (49.2%)*	*17,816 (52.4%)*	*15,590 (45.9%)*
Maximum follow-up time	*14,166 (20.8%)*	*6,848 (20.2%)*	*7,318 (21.5%)*
Specified date reached	*571 (0.8%)*	*424 (1.2%)*	*147 (0.4%)*
End of patient data	*0 (0.0%)*	*0 (0.0%)*	*0 (0.0%)*
End of patient enrollment	*12,210 (18.0%)*	*6,261 (18.4%)*	*5,949 (17.5%)*
Censor: Other GLP-1-RAs or DPP4i / Nursing home began	*5,019 (7.4%)*	*1,997 (5.9%)*	*3,022 (8.9%)*
2P-MACE			
Outcome	697 (1.0%)	297 (0.9%)	400 (1.2%)
Start of an additional exposure	1,428 (2.1%)	195 (0.6%)	1,233 (3.6%)
End of index exposure	33,406 (49.2%)	17,816 (52.4%)	15,590 (45.9%)
Maximum follow-up time	14,166 (20.8%)	6,848 (20.2%)	7,318 (21.5%)
Specified date reached	571 (0.8%)	424 (1.2%)	147 (0.4%)
End of patient data	0 (0.0%)	0 (0.0%)	0 (0.0%)
End of patient enrollment	12,210 (18.0%)	6,261 (18.4%)	5,949 (17.5%)
Censor: Other GLP-1-RAs or DPP4i / Nursing home began	5,019 (7.4%)	1,997 (5.9%)	3,022 (8.9%)
Death	762 (0.5%)	253 (0.3%)	509 (0.6%)

(A) Semaglutide vs sitagliptin in cohort meeting expanded eligibility criteria, Marketscan, 3P-MACE and 2P-MACE			
	Count (%)		
Censor reason	Overall	Semaglutide	Sitagliptin
3P-MACE			
Outcome	*401 (0.6%)*	*182 (0.5%)*	*219 (0.7%)*
Start of an additional exposure	*1,812 (2.7%)*	*222 (0.7%)*	*1,590 (4.8%)*
End of index exposure	*28,839 (43.4%)*	*14,688 (44.3%)*	*14,151 (42.6%)*
Maximum follow-up time	*16,974 (25.6%)*	*9,282 (28.0%)*	*7,692 (23.2%)*
Specified date reached	*5,309 (8.0%)*	*2,783 (8.4%)*	*2,526 (7.6%)*
End of patient data	*0 (0.0%)*	*0 (0.0%)*	*0 (0.0%)*
End of patient enrollment	*8,913 (13.4%)*	*4,662 (14.0%)*	*4,251 (12.8%)*
Censor: Other GLP-1-RAs or DPP4i / Nursing home began	*4,130 (6.2%)*	*1,370 (4.1%)*	*2,760 (8.3%)*
2P-MACE			
Outcome	340 (0.5%)	159 (0.5%)	181 (0.5%)
Start of an additional exposure	1,812 (2.7%)	222 (0.7%)	1,590 (4.8%)
End of index exposure	28,839 (43.4%)	14,688 (44.3%)	14,151 (42.6%)
Maximum follow-up time	16,974 (25.6%)	9,282 (28.0%)	7,692 (23.2%)
Specified date reached	5,309 (8.0%)	2,783 (8.4%)	2,526 (7.6%)
End of patient data	0 (0.0%)	0 (0.0%)	0 (0.0%)
End of patient enrollment	8,913 (13.4%)	4,662 (14.0%)	4,251 (12.8%)
Censor: Other GLP-1-RAs or DPP4i / Nursing home began	4,130 (6.2%)	1,370 (4.1%)	2,760 (8.3%)
Death	61 (0.1%)	23 (0.1%)	38 (0.1%)

(A) Semaglutide vs sitagliptin in cohort meeting expanded eligibility criteria, Medicare, 3P-MACE and 2P-MACE			
	Count (%)		
Censor reason	Overall	Semaglutide	Sitagliptin
3P-MACE			
Outcome	*649 (2.6%)*	*274 (2.2%)*	*375 (3.0%)*
Start of an additional exposure	*300 (1.2%)*	*128 (1.0%)*	*172 (1.4%)*
End of index exposure	*10,793 (43.7%)*	*5,872 (47.6%)*	*4,921 (39.9%)*
Maximum follow-up time	*3,484 (14.1%)*	*1,672 (13.6%)*	*1,812 (14.7%)*
Specified date reached	*6,744 (27.3%)*	*3,286 (26.6%)*	*3,458 (28.0%)*
End of patient data	*0 (0.0%)*	*0 (0.0%)*	*0 (0.0%)*

End of patient enrollment	*659 (2.7%)*	*326 (2.6%)*	*333 (2.7%)*
Censor: Other GLP-1-RAs or DPP4i / Nursing home began	*2,049 (8.3%)*	*781 (6.3%)*	*1,268 (10.3%)*
2P-MACE			
Outcome	397 (1.6%)	179 (1.5%)	218 (1.8%)
Start of an additional exposure	300 (1.2%)	128 (1.0%)	172 (1.4%)
End of index exposure	10,793 (43.7%)	5,872 (47.6%)	4,921 (39.9%)
Maximum follow-up time	3,484 (14.1%)	1,672 (13.6%)	1,812 (14.7%)
Specified date reached	6,744 (27.3%)	3,286 (26.6%)	3,458 (28.0%)
End of patient data	0 (0.0%)	0 (0.0%)	0 (0.0%)
End of patient enrollment	659 (2.7%)	326 (2.6%)	333 (2.7%)
Censor: Other GLP-1-RAs or DPP4i / Nursing home began	2,049 (8.3%)	781 (6.3%)	1,268 (10.3%)
Death	252 (1.0%)	95 (0.8%)	157 (1.3%)
(B) Tirzepatide vs dulaglutide in cohort meeting trial eligibility criteria, pooled across databases			
	Count (%)		
Censor reason	Overall	Tirzepatide	Sitagliptin
Outcome	431 (1.7%)	204 (1.6%)	227 (1.8%)
Start of an additional exposure	729 (2.8%)	107 (0.8%)	622 (4.8%)
End of index exposure	10,218 (39.8%)	4,542 (35.4%)	5,676 (44.3%)
Maximum follow-up time	3,805 (14.8%)	2,379 (18.5%)	1,426 (11.1%)
Specified date reached	3,775 (14.7%)	2,183 (17.0%)	1,592 (12.4%)
End of patient data	0 (0.0%)	0 (0.0%)	0 (0.0%)
End of patient enrollment	5,005 (19.5%)	2,715 (21.2%)	2,290 (17.9%)
Censor: Other GLP-1-RAs / Nursing home began	1,691 (6.6%)	697 (5.4%)	994 (7.7%)
(B) Tirzepatide vs dulaglutide in cohort meeting trial eligibility criteria, Optum			
	Count (%)		
Censor reason	Overall	Tirzepatide	Sitagliptin
Outcome	362 (1.9%)	169 (1.8%)	193 (2.0%)
Start of an additional exposure	533 (2.8%)	76 (0.8%)	457 (4.8%)
End of index exposure	8,491 (44.5%)	3,844 (40.3%)	4,647 (48.8%)
Maximum follow-up time	3,215 (16.9%)	2,028 (21.3%)	1,187 (12.5%)
Specified date reached	464 (2.4%)	284 (3.0%)	180 (1.9%)

End of patient data	0 (0.0%)	0 (0.0%)	0 (0.0%)
End of patient enrollment	4,596 (24.1%)	2,524 (26.5%)	2,072 (21.7%)
Censor: Other GLP-1-RAs / Nursing home began	1,399 (7.3%)	605 (6.3%)	794 (8.3%)
**(B) Tirzepatide vs dulaglutide in cohort meeting trial eligibility criteria, Marketscan**			
	Count (%)		
Censor reason	Overall	Tirzepatide	Semaglutide
Outcome	69 (1.0%)	35 (1.1%)	34 (1.0%)
Start of an additional exposure	196 (3.0%)	31 (0.9%)	165 (5.0%)
End of index exposure	1,727 (26.2%)	698 (21.2%)	1,029 (31.2%)
Maximum follow-up time	590 (8.9%)	351 (10.6%)	239 (7.2%)
Specified date reached	3,311 (50.2%)	1,899 (57.6%)	1,412 (42.8%)
End of patient data	0 (0.0%)	0 (0.0%)	0 (0.0%)
End of patient enrollment	409 (6.2%)	191 (5.8%)	218 (6.6%)
Censor: Other GLP-1-RAs / Nursing home began	292 (4.4%)	92 (2.8%)	200 (6.1%)
**(B) Tirzepatide vs dulaglutide in cohort meeting expanded eligibility criteria, pooled across databases**			
	Count (%)		
Censor reason	Overall	Tirzepatide	Dulaglutide
Outcome	717 (0.9%)	348 (0.9%)	369 (0.9%)
Start of an additional exposure	2,455 (3.1%)	319 (0.8%)	2,136 (5.5%)
End of index exposure	29,072 (37.1%)	12,295 (31.4%)	16,777 (42.9%)
Maximum follow-up time	12,893 (16.5%)	8,129 (20.8%)	4,764 (12.2%)
Specified date reached	15,256 (19.5%)	9,019 (23.0%)	6,237 (15.9%)
End of patient data	0 (0.0%)	0 (0.0%)	0 (0.0%)
End of patient enrollment	13,261 (16.9%)	7,163 (18.3%)	6,098 (15.6%)
Censor: Other GLP-1-RAs / Nursing home began	4,650 (5.9%)	1,879 (4.8%)	2,771 (7.1%)
**(B) Tirzepatide vs dulaglutide in cohort meeting expanded eligibility criteria, Optum**			
	Count (%)		
Censor reason	Overall	Tirzepatide	Dulaglutide
Outcome	571 (1.2%)	279 (1.1%)	292 (1.2%)
Start of an additional exposure	1,558 (3.1%)	205 (0.8%)	1,353 (5.5%)
End of index exposure	21,410 (43.2%)	9,390 (37.9%)	12,020 (48.5%)
Maximum follow-up time	9,786 (19.7%)	6,269 (25.3%)	3,517 (14.2%)

Specified date reached	1,677 (3.4%)	1,030 (4.2%)	647 (2.6%)
End of patient data	0 (0.0%)	0 (0.0%)	0 (0.0%)
End of patient enrollment	11,236 (22.7%)	6,165 (24.9%)	5,071 (20.5%)
Censor: Other GLP-1-RAs / Nursing home began	3,352 (6.8%)	1,457 (5.9%)	1,895 (7.6%)
**(B) Tirzepatide vs dulaglutide in cohort meeting expanded eligibility criteria, Marketscan**			
	Count (%)		
Censor reason	Overall	Tirzepatide	Dulaglutide
Outcome	146 (0.5%)	69 (0.5%)	77 (0.5%)
Start of an additional exposure	897 (3.1%)	114 (0.8%)	783 (5.5%)
End of index exposure	7,662 (26.7%)	2,905 (20.2%)	4,757 (33.1%)
Maximum follow-up time	3,107 (10.8%)	1,860 (13.0%)	1,247 (8.7%)
Specified date reached	13,579 (47.3%)	7,989 (55.6%)	5,590 (38.9%)
End of patient data	0 (0.0%)	0 (0.0%)	0 (0.0%)
End of patient enrollment	2,025 (7.1%)	998 (7.0%)	1,027 (7.2%)
Censor: Other GLP-1-RAs / Nursing home began	1,298 (4.5%)	422 (2.9%)	876 (6.1%)
**(C) Tirzepatide vs semaglutide, pooled across databases**			
	Count (%)		
Censor reason	Overall	Tirzepatide	Semaglutide
Outcome	1,230 (0.7%)	645 (0.7%)	585 (0.7%)
Start of an additional exposure	6,689 (3.9%)	2,891 (3.4%)	3,798 (4.4%)
End of index exposure	57,928 (33.6%)	24,436 (28.4%)	33,492 (38.9%)
Maximum follow-up time	24,262 (14.1%)	14,014 (16.3%)	10,248 (11.9%)
Specified date reached	40,772 (23.7%)	22,785 (26.4%)	17,987 (20.9%)
End of patient data	0 (0.0%)	0 (0.0%)	0 (0.0%)
End of patient enrollment	37,802 (21.9%)	19,716 (22.9%)	18,086 (21.0%)
Censor: Other GLP-1-RAs / Nursing home began	3,699 (2.1%)	1,704 (2.0%)	1,995 (2.3%)
**(C) Tirzepatide vs semaglutide, Optum**			
	Count (%)		
Censor reason	Overall	Tirzepatide	Semaglutide
Outcome	1,048 (0.9%)	550 (0.9%)	498 (0.8%)
Start of an additional exposure	4,917 (4.0%)	2,334 (3.8%)	2,583 (4.2%)
End of index exposure	45,418 (37.3%)	20,006 (32.9%)	25,412 (41.8%)
Maximum follow-up time	20,732 (17.0%)	11,947 (19.6%)	8,785 (14.4%)

Specified date reached	12,062 (9.9%)	6,406 (10.5%)	5,656 (9.3%)
End of patient data	0 (0.0%)	0 (0.0%)	0 (0.0%)
End of patient enrollment	34,812 (28.6%)	18,269 (30.0%)	16,543 (27.2%)
Censor: Other GLP-1-RAs / Nursing home began	2,703 (2.2%)	1,334 (2.2%)	1,369 (2.2%)
(C) Tirzepatide vs semaglutide, Marketscan			
	Count (%)		
Censor reason	Overall	Tirzepatide	Semaglutide
Outcome	182 (0.4%)	95 (0.4%)	87 (0.3%)
Start of an additional exposure	1,772 (3.5%)	557 (2.2%)	1,215 (4.8%)
End of index exposure	12,510 (24.7%)	4,430 (17.5%)	8,080 (31.9%)
Maximum follow-up time	3,530 (7.0%)	2,067 (8.2%)	1,463 (5.8%)
Specified date reached	28,710 (56.6%)	16,379 (64.6%)	12,331 (48.7%)
End of patient data	0 (0.0%)	0 (0.0%)	0 (0.0%)
End of patient enrollment	2,990 (5.9%)	1,447 (5.7%)	1,543 (6.1%)
Censor: Other GLP-1-RAs / Nursing home began	996 (2.0%)	370 (1.5%)	626 (2.5%)

Supplementary Table 21. Secondary end points among individuals initiating (A) semaglutide vs sitagliptin, (B) tirzepatide vs dulaglutide, or (C) tirzepatide vs semaglutide, reported by eligibility criteria applied and database.

(A) Semaglutide vs sitagliptin in cohort meeting trial eligibility criteria, pooled across databases			
	1–year rate, % (95% CI)		
End point	Semaglutide	Sitagliptin	HR (95% CI)
All-cause mortality	1.19 (0.97 to 1.41)	1.90 (1.64 to 2.16)	0.58 (0.48 to 0.71)
Myocardial infarction	1.16 (0.96 to 1.38)	1.65 (1.41 to 1.87)	0.70 (0.57 to 0.86)
Stroke	0.90 (0.72 to 1.08)	1.12 (0.94 to 1.34)	0.82 (0.65 to 1.03)
(A) Semaglutide vs sitagliptin in cohort meeting trial eligibility criteria, Optum			
	1–year rate, % (95% CI)		
End point	Semaglutide	Sitagliptin	HR (95% CI)
All-cause mortality	1.16 (0.89 to 1.50)	2.17 (1.83 to 2.58)	0.47 (0.36 to 0.62)
Myocardial infarction	1.24 (0.92 to 1.58)	1.52 (1.24 to 1.85)	0.72 (0.54 to 0.97)
Stroke	0.64 (0.47 to 0.83)	0.91 (0.69 to 1.20)	0.77 (0.53 to 1.10)
(A) Semaglutide vs sitagliptin in cohort meeting trial eligibility criteria, Marketscan			
	1–year rate, % (95% CI)		
End point	Semaglutide	Sitagliptin	HR (95% CI)
All-cause mortality	0.35 (0.20 to 0.59)	0.33 (0.17 to 0.63)	1.23 (0.56 to 2.70)
Myocardial infarction	0.88 (0.58 to 1.18)	1.38 (0.99 to 1.81)	0.69 (0.44 to 1.08)
Stroke	0.56 (0.33 to 0.80)	0.70 (0.40 to 1.07)	0.88 (0.50 to 1.58)
(A) Semaglutide vs sitagliptin in cohort meeting trial eligibility criteria, Medicare			
	1–year rate, % (95% CI)		
End point	Semaglutide	Sitagliptin	HR (95% CI)
All-cause mortality	2.05 (1.52 to 2.77)	2.85 (2.24 to 3.61)	0.70 (0.51 to 0.97)
Myocardial infarction	1.25 (0.81 to 1.73)	2.17 (1.60 to 2.81)	0.68 (0.46 to 0.98)
Stroke	1.74 (1.17 to 2.29)	1.93 (1.42 to 2.41)	0.84 (0.59 to 1.21)
(A) Semaglutide vs sitagliptin in cohort meeting expanded eligibility criteria, pooled across databases			
	1–year rate, % (95% CI)		

End point	Semaglutide	Sitagliptin	HR (95% CI)
All-cause mortality	*0.77 (0.67 to 0.87)*	*1.32 (1.20 to 1.44)*	*0.55 (0.47 to 0.63)*
Myocardial infarction	0.89 (0.78 to 0.98)	1.08 (0.98 to 1.19)	0.81 (0.70 to 0.92)
Stroke	0.63 (0.55 to 0.73)	0.70 (0.63 to 0.79)	0.84 (0.71 to 0.99)
Hospitalization for heart failure, urgent visit requiring IV diuretics, or all-cause mortality	*2.19 (2.03 to 2.35)*	*3.50 (3.30 to 3.69)*	*0.61 (0.57 to 0.66)*
Hospitalization for heart failure, urgent visit requiring IV diuretics	1.52 (1.39 to 1.64)	2.37 (2.21 to 2.53)	0.64 (0.59 to 0.71)
**(A) Semaglutide vs sitagliptin in cohort meeting expanded eligibility criteria, Optum**			
	1–year rate, % (95% CI)		
End point	Semaglutide	Sitagliptin	HR (95% CI)
All-cause mortality	*0.94 (0.78 to 1.12)*	*1.88 (1.67 to 2.11)*	*0.46 (0.38 to 0.56)*
Myocardial infarction	1.00 (0.85 to 1.14)	1.19 (1.00 to 1.38)	0.86 (0.71 to 1.04)
Stroke	0.57 (0.45 to 0.68)	0.81 (0.69 to 0.95)	0.67 (0.52 to 0.85)
Hospitalization for heart failure, urgent visit requiring IV diuretics, or all-cause mortality	*2.56 (2.31 to 2.82)*	*4.32 (4.01 to 4.65)*	*0.60 (0.54 to 0.67)*
Hospitalization for heart failure, urgent visit requiring IV diuretics	1.75 (1.56 to 1.97)	2.75 (2.50 to 3.01)	0.67 (0.59 to 0.76)
**(A) Semaglutide vs sitagliptin in cohort meeting expanded eligibility criteria, Marketscan**			
	1–year rate, % (95% CI)		
End point	Semaglutide	Sitagliptin	HR (95% CI)
All-cause mortality	*0.14 (0.10 to 0.22)*	*0.25 (0.18 to 0.35)*	*0.61 (0.37 to 0.98)*
Myocardial infarction	0.55 (0.45 to 0.67)	0.68 (0.56 to 0.80)	0.77 (0.60 to 1.00)
Stroke	0.27 (0.19 to 0.34)	0.28 (0.22 to 0.37)	0.95 (0.65 to 1.39)
Hospitalization for heart failure, urgent visit requiring IV diuretics, or all-cause mortality	*0.93 (0.80 to 1.08)*	*1.51 (1.34 to 1.72)*	*0.62 (0.52 to 0.75)*
Hospitalization for heart failure, urgent visit requiring IV diuretics	0.79 (0.67 to 0.93)	1.29 (1.13 to 1.48)	0.62 (0.51 to 0.76)
**(A) Semaglutide vs sitagliptin in cohort meeting expanded eligibility criteria, Medicare**			
	1–year rate, % (95% CI)		
End point	Semaglutide	Sitagliptin	HR (95% CI)
All-cause mortality	*1.97 (1.59 to 2.44)*	*2.62 (2.21 to 3.12)*	*0.69 (0.54 to 0.87)*
Myocardial infarction	1.49 (1.15 to 1.85)	1.84 (1.46 to 2.21)	0.74 (0.56 to 0.97)
Stroke	1.76 (1.38 to 2.20)	1.51 (1.19 to 1.87)	1.06 (0.81 to 1.41)
Hospitalization for heart failure, urgent visit requiring IV diuretics, or all-cause mortality	*4.53 (3.94 to 5.20)*	*6.49 (5.84 to 7.22)*	*0.63 (0.55 to 0.74)*
Hospitalization for heart failure, urgent visit requiring IV diuretics	2.82 (2.36 to 3.37)	4.27 (3.75 to 4.87)	0.61 (0.50 to 0.73)

(B) Tirzepatide vs dulaglutide in cohort meeting trial eligibility criteria, pooled across databases			
	1–year rate, % (95% CI)		
End point	Tirzepatide	Dulaglutide	HR (95% CI)
All-cause mortality	0.81 (0.56 to 1.06)	0.94 (0.66 to 1.22)	0.76 (0.52 to 1.11)
Myocardial infarction	1.31 (1.04 to 1.63)	1.79 (1.37 to 2.17)	0.81 (0.61 to 1.06)
Stroke	0.83 (0.61 to 1.02)	1.01 (0.76 to 1.29)	0.92 (0.65 to 1.29)
(B) Tirzepatide vs dulaglutide in cohort meeting trial eligibility criteria, Optum			
	1–year rate, % (95% CI)		
End point	Tirzepatide	Dulaglutide	HR (95% CI)
All-cause mortality	1.05 (0.77 to 1.43)	1.23 (0.91 to 1.66)	0.75 (0.51 to 1.10)
Myocardial infarction	1.31 (1.03 to 1.65)	1.91 (1.47 to 2.39)	0.77 (0.57 to 1.05)
Stroke	0.92 (0.67 to 1.23)	1.19 (0.82 to 1.62)	0.86 (0.59 to 1.25)
(B) Tirzepatide vs dulaglutide in cohort meeting trial eligibility criteria, Marketscan			
	1–year rate, % (95% CI)		
End point	Tirzepatide	Dulaglutide	HR (95% CI)
All-cause mortality	0.13 (0.03 to 0.58)	0.10 (0.02 to 0.44)	1.05 (0.15 to 7.48)
Myocardial infarction	1.32 (0.71 to 2.09)	1.43 (0.80 to 2.23)	0.96 (0.53 to 1.73)
Stroke	0.58 (0.27 to 0.94)	0.49 (0.21 to 0.83)	1.27 (0.55 to 2.94)
(B) Tirzepatide vs dulaglutide in cohort meeting expanded eligibility criteria, pooled across databases			
	1–year rate, % (95% CI)		
End point	Tirzepatide	Dulaglutide	HR (95% CI)
All-cause mortality	0.48 (0.38 to 0.59)	0.48 (0.38 to 0.59)	0.88 (0.68 to 1.16)
Myocardial infarction	0.69 (0.58 to 0.80)	0.88 (0.74 to 1.04)	0.91 (0.73 to 1.12)
Stroke	0.36 (0.28 to 0.43)	0.48 (0.39 to 0.58)	0.78 (0.59 to 1.03)
Hospitalization for heart failure, urgent visit requiring IV diuretics, or all-cause mortality	1.64 (1.45 to 1.83)	2.07 (1.84 to 2.30)	0.75 (0.65 to 0.86)
(B) Tirzepatide vs dulaglutide in cohort meeting expanded eligibility criteria, Optum			
	1–year rate, % (95% CI)		
End point	Tirzepatide	Dulaglutide	HR (95% CI)

All-cause mortality	0.72 (0.59 to 0.89)	0.71 (0.57 to 0.90)	0.91 (0.69 to 1.20)
Myocardial infarction	0.78 (0.65 to 0.93)	1.01 (0.81 to 1.19)	0.87 (0.68 to 1.11)
Stroke	0.43 (0.32 to 0.53)	0.62 (0.50 to 0.79)	0.73 (0.53 to 1.00)
Hospitalization for heart failure, urgent visit requiring IV diuretics, or all-cause mortality	2.14 (1.89 to 2.41)	2.48 (2.20 to 2.79)	0.78 (0.67 to 0.91)
**(B) Tirzepatide vs dulaglutide in cohort meeting expanded eligibility criteria, Marketscan**			
	1-year-rate, % (95% CI)		
End point	Tirzepatide	Dulaglutide	HR (95% CI)
All-cause mortality	0.07 (0.02 to 0.23)	0.08 (0.04 to 0.16)	0.57 (0.19 to 1.69)
Myocardial infarction	0.54 (0.37 to 0.73)	0.66 (0.43 to 0.86)	1.01 (0.67 to 1.51)
Stroke	0.23 (0.13 to 0.33)	0.24 (0.15 to 0.36)	0.99 (0.54 to 1.79)
Hospitalization for heart failure, urgent visit requiring IV diuretics, or all-cause mortality	0.79 (0.57 to 1.08)	1.39 (1.07 to 1.80)	0.61 (0.44 to 0.85)
**(C) Tirzepatide vs semaglutide, pooled across databases**			
	1-year-rate, % (95% CI)		
End point	Tirzepatide	Semaglutide	HR (95% CI)
All-cause mortality	0.45 (0.38 to 0.53)	0.41 (0.34 to 0.49)	1.03 (0.84 to 1.27)
Myocardial infarction	0.63 (0.55 to 0.71)	0.69 (0.60 to 0.79)	1.03 (0.88 to 1.21)
Stroke	0.31 (0.26 to 0.36)	0.29 (0.23 to 0.35)	1.15 (0.92 to 1.45)
Hospitalization for heart failure, urgent visit requiring IV diuretics, or all-cause mortality	1.45 (1.32 to 1.58)	1.51 (1.38 to 1.65)	0.91 (0.81 to 1.01)
**(C) Tirzepatide vs semaglutide, Optum**			
	1-year-rate, % (95% CI)		
End point	Tirzepatide	Semaglutide	HR (95% CI)
All-cause mortality	0.62 (0.53 to 0.72)	0.57 (0.48 to 0.68)	1.04 (0.84 to 1.27)
Myocardial infarction	0.69 (0.60 to 0.78)	0.73 (0.61 to 0.84)	1.02 (0.85 to 1.21)
Stroke	0.37 (0.30 to 0.45)	0.35 (0.27 to 0.46)	1.11 (0.86 to 1.42)
Hospitalization for heart failure, urgent visit requiring IV diuretics, or all-cause mortality	1.76 (1.60 to 1.92)	1.83 (1.66 to 2.01)	0.92 (0.82 to 1.04)
**(C) Tirzepatide vs semaglutide, Marketscan**			
	1-year-rate, % (95% CI)		
End point	Tirzepatide	Semaglutide	HR (95% CI)
All-cause mortality	0.06 (0.02 to 0.19)	0.04 (0.02 to 0.09)	0.88 (0.30 to 2.63)

Myocardial infarction	0.48 (0.36 to 0.63)	0.59 (0.41 to 0.81)	1.07 (0.76 to 1.50)
Stroke	0.17 (0.09 to 0.25)	0.12 (0.07 to 0.18)	1.43 (0.79 to 2.59)
Hospitalization for heart failure, urgent visit requiring IV diuretics, or all-cause mortality	0.72 (0.54 to 0.95)	0.78 (0.61 to 0.99)	0.83 (0.62 to 1.10)

Supplementary Table 22. Safety end points among individuals initiating (A) semaglutide vs sitagliptin, (B) tirzepatide vs dulaglutide, or (C) tirzepatide vs semaglutide, reported by eligibility criteria applied and database.

(A) Semaglutide vs sitagliptin in cohort meeting expanded eligibility criteria, pooled across databases			
	1–year rate, % (95% CI)		
End point	Semaglutide	Sitagliptin	HR (95% CI)
Urinary tract infection	8.74 (8.44 to 9.04)	8.81 (8.51 to 9.10)	0.98 (0.94 to 1.02)
Serious bacterial infection	2.63 (2.45 to 2.80)	3.40 (3.21 to 3.59)	0.76 (0.70 to 0.82)
Gastrointestinal adverse events	1.46 (1.33 to 1.58)	1.44 (1.32 to 1.56)	1.02 (0.92 to 1.13)
(A) Semaglutide vs sitagliptin in cohort meeting expanded eligibility criteria, Optum			
	1–year rate, % (95% CI)		
End point	Semaglutide	Sitagliptin	HR (95% CI)
Urinary tract infection	10.07 (9.59 to 10.56)	10.16 (9.71 to 10.64)	0.99 (0.94 to 1.06)
Serious bacterial infection	2.74 (2.48 to 3.02)	3.50 (3.23 to 3.80)	0.77 (0.69 to 0.87)
Gastrointestinal adverse events	1.76 (1.56 to 1.97)	1.57 (1.39 to 1.77)	1.11 (0.96 to 1.29)
(A) Semaglutide vs sitagliptin in cohort meeting expanded eligibility criteria, Marketscan			
	1–year rate, % (95% CI)		
End point	Semaglutide	Sitagliptin	HR (95% CI)
Urinary tract infection	6.33 (5.98 to 6.71)	6.36 (5.99 to 6.75)	0.97 (0.90 to 1.05)
Serious bacterial infection	1.99 (1.79 to 2.21)	2.55 (2.31 to 2.81)	0.76 (0.67 to 0.87)
Gastrointestinal adverse events	1.18 (1.03 to 1.35)	1.29 (1.13 to 1.47)	0.90 (0.75 to 1.07)
(A) Semaglutide vs sitagliptin in cohort meeting expanded eligibility criteria, Medicare			
	1–year rate, % (95% CI)		
End point	Semaglutide	Sitagliptin	HR (95% CI)
Urinary tract infection	11.69 (10.79 to 12.67)	11.83 (10.97 to 12.76)	0.96 (0.87 to 1.06)
Serious bacterial infection	4.14 (3.60 to 4.75)	5.54 (4.94 to 6.20)	0.73 (0.62 to 0.85)
Gastrointestinal adverse events	1.42 (1.13 to 1.77)	1.55 (1.24 to 1.94)	1.03 (0.78 to 1.35)
(B) Tirzepatide vs dulaglutide in cohort meeting expanded eligibility criteria, pooled across databases			
	1–year rate, % (95% CI)		

End point	Tirzepatide	Dulaglutide	HR (95% CI)
Urinary tract infection	8.37 (7.96 to 8.78)	8.02 (7.56 to 8.48)	1.04 (0.97 to 1.11)
Serious bacterial infection	2.28 (2.06 to 2.50)	2.51 (2.26 to 2.77)	0.91 (0.81 to 1.03)
Gastrointestinal adverse events	1.52 (1.34 to 1.70)	1.45 (1.26 to 1.64)	1.02 (0.88 to 1.19)
**(B) Tirzepatide vs dulaglutide in cohort meeting expanded eligibility criteria, Optum**			
	1–year rate, % (95% CI)		
End point	Tirzepatide	Dulaglutide	HR (95% CI)
Urinary tract infection	9.22 (8.73 to 9.73)	8.96 (8.39 to 9.56)	1.02 (0.94 to 1.10)
Serious bacterial infection	2.56 (2.30 to 2.85)	2.80 (2.49 to 3.16)	0.95 (0.82 to 1.09)
Gastrointestinal adverse events	1.65 (1.44 to 1.88)	1.71 (1.46 to 1.99)	0.96 (0.80 to 1.15)
**(B) Tirzepatide vs dulaglutide in cohort meeting expanded eligibility criteria, Marketscan**			
	1–year rate, % (95% CI)		
End point	Tirzepatide	Dulaglutide	HR (95% CI)
Urinary tract infection	7.02 (6.34 to 7.78)	6.47 (5.75 to 7.27)	1.09 (0.96 to 1.23)
Serious bacterial infection	1.82 (1.49 to 2.23)	2.06 (1.71 to 2.48)	0.82 (0.65 to 1.03)
Gastrointestinal adverse events	1.32 (1.05 to 1.65)	1.04 (0.81 to 1.33)	1.21 (0.90 to 1.61)
**(C) Tirzepatide vs semaglutide, pooled across databases**			
	1–year rate, % (95% CI)		
End point	Tirzepatide	Semaglutide	HR (95% CI)
Urinary tract infection	8.30 (8.00 to 8.60)	7.92 (7.61 to 8.23)	1.04 (0.99 to 1.08)
Serious bacterial infection	1.85 (1.71 to 1.99)	1.92 (1.76 to 2.08)	1.01 (0.92 to 1.11)
Gastrointestinal adverse events	1.44 (1.31 to 1.56)	1.38 (1.24 to 1.52)	1.12 (1.00 to 1.25)
**(C) Tirzepatide vs semaglutide, Optum**			
	1–year rate, % (95% CI)		
End point	Tirzepatide	Semaglutide	HR (95% CI)
Urinary tract infection	8.62 (8.30 to 8.96)	8.36 (8.01 to 8.72)	1.04 (0.99 to 1.09)
Serious bacterial infection	1.98 (1.82 to 2.15)	2.07 (1.89 to 2.27)	1.03 (0.92 to 1.14)
Gastrointestinal adverse events	1.48 (1.35 to 1.63)	1.38 (1.23 to 1.54)	1.15 (1.01 to 1.31)

(C) Tirzepatide vs semaglutide, Marketscan			
	1–year rate, % (95% CI)		
End point	Tirzepatide	Semaglutide	HR (95% CI)
Urinary tract infection	7.65 (7.04 to 8.30)	6.96 (6.37 to 7.60)	1.03 (0.94 to 1.12)
Serious bacterial infection	1.55 (1.30 to 1.86)	1.59 (1.32 to 1.91)	0.96 (0.79 to 1.17)
Gastrointestinal adverse events	1.33 (1.10 to 1.61)	1.40 (1.13 to 1.73)	1.05 (0.85 to 1.30)

Supplementary Table 23. Sensitivity analyses among individuals initiating (A) semaglutide vs sitagliptin, (B) tirzepatide vs dulaglutide, or (C) tirzepatide vs semaglutide, reported by eligibility criteria applied and database.

(A) Semaglutide vs sitagliptin in cohort meeting trial eligibility criteria, pooled across databases			
	1–year rate, % (95% CI)		
Sensitivity analysis	Semaglutide	Sitagliptin	HR (95% CI)
As-started causal contrast	3.41 (3.15 to 3.66)	4.53 (4.24 to 4.82)	0.74 (0.67 to 0.82)
Lumbar radiculopathy	7.53 (7.04 to 8.02)	6.88 (6.44 to 7.32)	1.06 (0.98 to 1.15)
Abdominal hernia	6.15 (5.69 to 6.60)	6.00 (5.57 to 6.43)	1.06 (0.96 to 1.16)
(A) Semaglutide vs sitagliptin in cohort meeting trial eligibility criteria, Optum			
	1–year rate, % (95% CI)		
Sensitivity analysis	Semaglutide	Sitagliptin	HR (95% CI)
As-started causal contrast	3.27 (2.95 to 3.62)	4.59 (4.22 to 5.00)	0.70 (0.61 to 0.80)
Individuals with HbA1c measurement	1.69 (0.85 to 3.33)	3.36 (2.28 to 4.95)	0.36 (0.18 to 0.70)
Lumbar radiculopathy	8.65 (7.93 to 9.42)	7.36 (6.76 to 8.00)	1.11 (1.00 to 1.24)
Abdominal hernia	6.26 (5.66 to 6.93)	6.34 (5.75 to 6.99)	1.04 (0.91 to 1.17)
(A) Semaglutide vs sitagliptin in cohort meeting trial eligibility criteria, Marketscan			
	1–year rate, % (95% CI)		
Sensitivity analysis	Semaglutide	Sitagliptin	HR (95% CI)
As-started causal contrast	2.19 (1.82 to 2.63)	1.98 (1.64 to 2.40)	1.09 (0.83 to 1.42)
Lumbar radiculopathy	7.34 (6.50 to 8.29)	6.86 (6.04 to 7.78)	1.05 (0.89 to 1.24)
Abdominal hernia	4.82 (4.14 to 5.62)	4.90 (4.19 to 5.74)	1.02 (0.83 to 1.25)
(A) Semaglutide vs sitagliptin in cohort meeting trial eligibility criteria, Medicare			
	1–year rate, % (95% CI)		
Sensitivity analysis	Semaglutide	Sitagliptin	HR (95% CI)
As-started causal contrast	4.84 (4.24 to 5.51)	6.79 (6.09 to 7.58)	0.70 (0.59 to 0.83)
Lumbar radiculopathy	5.66 (4.80 to 6.66)	6.18 (5.35 to 7.14)	0.94 (0.78 to 1.13)
Abdominal hernia	7.22 (6.24 to 8.36)	6.45 (5.57 to 7.46)	1.12 (0.94 to 1.34)
(A) Semaglutide vs sitagliptin in cohort meeting expanded eligibility criteria, pooled across databases			

	1–year rate, % (95% CI)		
Sensitivity analysis	Semaglutide	Sitagliptin	HR (95% CI)
As-started causal contrast, 3P-MACE	*2.55 (2.42 to 2.67)*	*3.13 (3.00 to 3.27)*	*0.79 (0.74 to 0.84)*
As-started causal contrast, 2P-MACE	2.27 (2.15 to 2.39)	2.51 (2.39 to 2.64)	0.89 (0.82 to 0.96)
Lumbar radiculopathy	6.44 (6.18 to 6.69)	6.48 (6.23 to 6.73)	0.98 (0.93 to 1.03)
Abdominal hernia	5.02 (4.79 to 5.25)	4.97 (4.74 to 5.20)	1.04 (0.98 to 1.10)
(A) Semaglutide vs sitagliptin in cohort meeting expanded eligibility criteria, Optum			
	1–year rate, % (95% CI)		
Sensitivity analysis	Semaglutide	Sitagliptin	HR (95% CI)
As-started causal contrast, 3P-MACE	*3.14 (2.94 to 3.35)*	*3.88 (3.66 to 4.11)*	*0.79 (0.73 to 0.87)*
As-started causal contrast, 2P-MACE	3.24 (3.03 to 3.47)	3.54 (3.32 to 3.78)	0.91 (0.81 to 1.02)
Individuals with HbA1c measurement, 3P-MACE	*2.36 (2.02 to 2.74)*	*3.33 (2.95 to 3.76)*	*0.69 (0.58 to 0.82)*
Individuals with HbA1c measurement, 2P-MACE	1.51 (1.25 to 1.82)	1.67 (1.41 to 1.99)	0.90 (0.72 to 1.13)
Lumbar radiculopathy	7.79 (7.37 to 8.24)	7.51 (7.12 to 7.93)	0.99 (0.92 to 1.06)
Abdominal hernia	5.32 (4.97 to 5.70)	5.57 (5.22 to 5.95)	1.00 (0.92 to 1.09)
(A) Semaglutide vs sitagliptin in cohort meeting expanded eligibility criteria, Marketscan			
	1–year rate, % (95% CI)		
Sensitivity analysis	Semaglutide	Sitagliptin	HR (95% CI)
As-started causal contrast, 3P-MACE	*1.10 (0.99 to 1.24)*	*1.22 (1.10 to 1.36)*	*0.89 (0.77 to 1.04)*
As-started causal contrast, 2P-MACE	0.93 (0.82 to 1.05)	1.01 (0.89 to 1.13)	0.91 (0.77 to 1.08)
Lumbar radiculopathy	5.47 (5.14 to 5.82)	5.56 (5.21 to 5.93)	0.99 (0.91 to 1.08)
Abdominal hernia	4.02 (3.74 to 4.33)	3.64 (3.35 to 3.94)	1.12 (1.02 to 1.24)
(A) Semaglutide vs sitagliptin in cohort meeting expanded eligibility criteria, Medicare			
	1–year rate, % (95% CI)		
Sensitivity analysis	Semaglutide	Sitagliptin	HR (95% CI)
As-started causal contrast, 3P-MACE	*4.77 (4.33 to 5.25)*	*6.15 (5.67 to 6.67)*	*0.73 (0.65 to 0.83)*
As-started causal contrast, 2P-MACE	3.20 (2.85 to 3.60)	3.75 (3.37 to 4.17)	0.82 (0.70 to 0.96)
Lumbar radiculopathy	5.57 (4.97 to 6.24)	6.44 (5.80 to 7.15)	0.93 (0.81 to 1.06)
Abdominal hernia	7.00 (6.29 to 7.79)	6.97 (6.28 to 7.74)	1.01 (0.89 to 1.16)

(B) Tirzepatide vs dulaglutide in cohort meeting trial eligibility criteria, pooled across databases			
	1–year rate, % (95% CI)		
Sensitivity analysis	Tirzepatide	Dulaglutide	HR (95% CI)
As-started causal contrast	3.55 (3.16 to 3.93)	4.24 (3.83 to 4.66)	0.84 (0.73 to 0.97)
Lumbar radiculopathy	8.52 (7.74 to 9.29)	8.11 (7.23 to 8.98)	1.06 (0.94 to 1.19)
Abdominal hernia	5.37 (4.75 to 5.98)	5.03 (4.39 to 5.67)	0.99 (0.85 to 1.14)
(B) Tirzepatide vs dulaglutide in cohort meeting trial eligibility criteria, Optum			
	1–year rate, % (95% CI)		
Sensitivity analysis	Tirzepatide	Dulaglutide	HR (95% CI)
As-started causal contrast	4.00 (3.57 to 4.49)	4.78 (4.30 to 5.30)	0.84 (0.72 to 0.98)
Individuals with HbA1c measurement	3.16 (2.31 to 4.31)	4.24 (3.20 to 5.61)	0.66 (0.45 to 0.96)
Lumbar radiculopathy	8.96 (8.13 to 9.87)	8.35 (7.42 to 9.37)	1.08 (0.95 to 1.23)
Abdominal hernia	5.81 (5.14 to 6.55)	5.61 (4.88 to 6.45)	1.00 (0.85 to 1.17)
(B) Tirzepatide vs dulaglutide in cohort meeting trial eligibility criteria, Marketscan			
	1–year rate, % (95% CI)		
Sensitivity analysis	Tirzepatide	Dulaglutide	HR (95% CI)
As-started causal contrast	2.21 (1.61 to 3.04)	2.69 (2.07 to 3.51)	0.85 (0.57 to 1.25)
Lumbar radiculopathy	7.36 (5.84 to 9.26)	7.52 (5.84 to 9.67)	0.98 (0.76 to 1.27)
Abdominal hernia	4.18 (3.07 to 5.67)	3.50 (2.58 to 4.73)	0.93 (0.65 to 1.33)
(B) Tirzepatide vs dulaglutide in cohort meeting expanded eligibility criteria, pooled across databases			
	1–year rate, % (95% CI)		
Sensitivity analysis	Tirzepatide	Dulaglutide	HR (95% CI)
As-started causal contrast	1.88 (1.72 to 2.04)	2.26 (2.09 to 2.43)	0.86 (0.77 to 0.96)
Lumbar radiculopathy	7.24 (6.86 to 7.62)	7.23 (6.79 to 7.67)	1.06 (0.99 to 1.13)
Abdominal hernia	4.65 (4.33 to 4.97)	4.78 (4.42 to 5.14)	0.96 (0.88 to 1.04)
(B) Tirzepatide vs dulaglutide in cohort meeting expanded eligibility criteria, Optum			
	1-year-rate, % (95% CI)		
Sensitivity analysis	Tirzepatide	Dulaglutide	HR (95% CI)
As-started causal contrast	2.45 (2.24 to 2.68)	2.83 (2.61 to 3.08)	0.88 (0.78 to 0.99)

Individuals with HbA1c measurement	2.02 (1.69 to 2.41)	1.95 (1.60 to 2.39)	1.02 (0.80 to 1.29)
Lumbar radiculopathy	8.21 (7.75 to 8.70)	8.03 (7.48 to 8.61)	1.07 (0.99 to 1.16)
Abdominal hernia	5.19 (4.81 to 5.60)	5.49 (5.04 to 5.99)	0.94 (0.85 to 1.04)
**(B) Tirzepatide vs dulaglutide in cohort meeting expanded eligibility criteria, Marketscan**			
	1-year-rate, % (95% CI)		
Sensitivity analysis	Tirzepatide	Dulaglutide	HR (95% CI)
As-started causal contrast	0.91 (0.72 to 1.14)	1.28 (1.06 to 1.53)	0.76 (0.57 to 1.00)
Lumbar radiculopathy	5.66 (5.08 to 6.30)	5.98 (5.31 to 6.73)	1.02 (0.89 to 1.16)
Abdominal hernia	3.77 (3.27 to 4.35)	3.57 (3.08 to 4.14)	1.01 (0.85 to 1.20)
**(C) Tirzepatide vs semaglutide, pooled across databases**			
	1-year-rate, % (95% CI)		
Sensitivity analysis	Tirzepatide	Semaglutide	HR (95% CI)
As-started causal contrast	1.59 (1.49 to 1.70)	1.58 (1.47 to 1.69)	1.04 (0.95 to 1.14)
Lumbar radiculopathy	7.45 (7.18 to 7.72)	7.23 (6.93 to 7.52)	1.03 (0.99 to 1.08)
Abdominal hernia	4.63 (4.40 to 4.86)	4.70 (4.45 to 4.95)	0.97 (0.91 to 1.03)
**(C) Tirzepatide vs semaglutide, Optum**			
	1-year-rate, % (95% CI)		
Sensitivity analysis	Tirzepatide	Semaglutide	HR (95% CI)
As-started causal contrast	1.93 (1.80 to 2.07)	1.92 (1.79 to 2.06)	1.02 (0.93 to 1.12)
Individuals with HbA1c measurement	1.60 (1.40 to 1.83)	1.42 (1.22 to 1.65)	1.08 (0.91 to 1.28)
Lumbar radiculopathy	8.28 (7.96 to 8.60)	7.82 (7.48 to 8.16)	1.05 (1.00 to 1.11)
Abdominal hernia	5.01 (4.76 to 5.28)	4.97 (4.70 to 5.26)	0.99 (0.93 to 1.06)
**(C) Tirzepatide vs semaglutide, Marketscan**			
	1-year-rate, % (95% CI)		
Sensitivity analysis	Tirzepatide	Semaglutide	HR (95% CI)
As-started causal contrast	0.79 (0.65 to 0.97)	0.75 (0.61 to 0.93)	1.22 (0.94 to 1.59)
Lumbar radiculopathy	5.65 (5.19 to 6.16)	5.99 (5.44 to 6.59)	0.98 (0.89 to 1.09)
Abdominal hernia	3.77 (3.34 to 4.25)	4.12 (3.66 to 4.64)	0.89 (0.78 to 1.01)

Supplementary Table 24. Subgroups for the primary end point among individuals initiating (A) semaglutide vs sitagliptin, (B) tirzepatide vs dulaglutide, or (C) tirzepatide vs semaglutide, reported by eligibility criteria applied and database.

(A) Semaglutide vs sitagliptin in cohort meeting expanded eligibility criteria, pooled across databases			
Subgroup	Semaglutide	Sitagliptin	HR (95% CI)
3P-MACE			
Female	*2.01 (1.79 to 2.23)*	*2.80 (2.56 to 3.04)*	*0.67 (0.59 to 0.75)*
Male	*2.44 (2.19 to 2.69)*	*3.13 (2.86 to 3.40)*	*0.74 (0.66 to 0.84)*
Age <65	*0.96 (0.82 to 1.09)*	*1.36 (1.19 to 1.54)*	*0.71 (0.59 to 0.85)*
Age ≥65	*3.36 (3.07 to 3.65)*	*4.30 (4.00 to 4.59)*	*0.70 (0.62 to 0.79)*
Concomitant SGLT2i use	*1.74 (1.40 to 2.08)*	*2.59 (2.17 to 3.01)*	*0.67 (0.53 to 0.84)*
No concomitant SGLT2i use	*2.25 (2.07 to 2.44)*	*2.98 (2.78 to 3.18)*	*0.72 (0.65 to 0.79)*
At high cardiovascular risk	*4.45 (4.04 to 4.86)*	*5.86 (5.42 to 6.29)*	*0.71 (0.64 to 0.79)*
Coronary heart disease	*5.18 (4.60 to 5.75)*	*6.09 (5.53 to 6.64)*	*0.75 (0.66 to 0.85)*
Cardiomyopathy	*6.57 (5.20 to 7.91)*	*7.86 (6.51 to 9.19)*	*0.78 (0.62 to 0.99)*
Valve disorders	*5.54 (4.67 to 6.40)*	*6.20 (5.43 to 6.97)*	*0.77 (0.64 to 0.92)*
Heart failure	*6.78 (5.91 to 7.64)*	*9.47 (8.54 to 10.39)*	*0.65 (0.56 to 0.74)*
High-intensive lipid-lowering therapy	*2.71 (2.34 to 3.08)*	*3.64 (3.25 to 4.02)*	*0.69 (0.59 to 0.80)*
2P-MACE			
Female	1.41 (1.22 to 1.59)	1.79 (1.60 to 1.99)	0.74 (0.64 to 0.85)
Male	1.63 (1.43 to 1.83)	1.76 (1.56 to 1.96)	0.86 (0.74 to 1.00)
Age <65	0.78 (0.66 to 0.91)	0.93 (0.78 to 1.07)	0.83 (0.68 to 1.01)
Age ≥65	2.20 (1.96 to 2.44)	2.46 (2.24 to 2.68)	0.82 (0.72 to 0.92)
Concomitant SGLT2i use	1.24 (0.96 to 1.53)	1.84 (1.48 to 2.20)	0.69 (0.53 to 0.90)
No concomitant SGLT2i use	1.54 (1.39 to 1.69)	1.74 (1.59 to 1.89)	0.84 (0.75 to 0.94)
At high cardiovascular risk	3.10 (2.76 to 3.45)	3.57 (3.24 to 3.91)	0.80 (0.71 to 0.91)
Coronary heart disease	3.53 (3.05 to 4.01)	3.69 (3.25 to 4.12)	0.85 (0.73 to 1.00)
Cardiomyopathy	3.96 (2.94 to 4.97)	4.37 (3.34 to 5.39)	0.87 (0.64 to 1.18)
Valve disorders	3.46 (2.77 to 4.15)	3.75 (3.14 to 4.36)	0.80 (0.64 to 1.01)
Heart failure	4.10 (3.42 to 4.77)	5.12 (4.44 to 5.80)	0.71 (0.59 to 0.86)
High-intensive lipid-lowering therapy	1.96 (1.66 to 2.27)	2.38 (2.07 to 2.69)	0.78 (0.65 to 0.93)
(A) Semaglutide vs sitagliptin in cohort meeting expanded eligibility criteria, Optum, 3P-MACE			

Subgroup	Semaglutide	Sitagliptin	HR (95% CI)
3P-MACE			
Female	*2.27 (1.96 to 2.62)*	*3.45 (3.08 to 3.85)*	*0.65 (0.56 to 0.77)*
Male	*2.88 (2.49 to 3.34)*	*3.83 (3.39 to 4.31)*	*0.72 (0.61 to 0.85)*
Age <65	*1.66 (1.33 to 2.06)*	*2.87 (2.40 to 3.45)*	*0.59 (0.45 to 0.77)*
Age ≥65	*2.90 (2.57 to 3.27)*	*4.03 (3.68 to 4.41)*	*0.68 (0.59 to 0.77)*
Concomitant SGLT2i use	*2.53 (1.96 to 3.25)*	*3.79 (3.11 to 4.63)*	*0.64 (0.48 to 0.86)*
No concomitant SGLT2i use	*2.51 (2.24 to 2.81)*	*3.67 (3.36 to 4.01)*	*0.67 (0.59 to 0.76)*
At high cardiovascular risk	*4.13 (3.62 to 4.70)*	*6.28 (5.69 to 6.92)*	*0.64 (0.55 to 0.74)*
Coronary heart disease	*5.22 (4.48 to 6.07)*	*6.41 (5.68 to 7.23)*	*0.72 (0.61 to 0.86)*
Cardiomyopathy	*6.55 (4.98 to 8.59)*	*8.38 (6.67 to 10.50)*	*0.77 (0.56 to 1.06)*
Valve disorders	*5.41 (4.36 to 6.70)*	*6.74 (5.71 to 7.94)*	*0.69 (0.54 to 0.88)*
Heart failure	*6.72 (5.65 to 7.99)*	*9.16 (8.03 to 10.44)*	*0.65 (0.54 to 0.79)*
High-intensive lipid-lowering therapy	*2.57 (2.13 to 3.11)*	*3.81 (3.31 to 4.39)*	*0.63 (0.51 to 0.78)*
2P-MACE			
Female	1.55 (1.30 to 1.85)	1.95 (1.68 to 2.26)	0.78 (0.64 to 0.96)
Male	1.73 (1.44 to 2.08)	2.05 (1.73 to 2.42)	0.86 (0.69 to 1.08)
Age <65	1.27 (0.99 to 1.63)	1.52 (1.19 to 1.94)	0.82 (0.60 to 1.12)
Age ≥65	1.80 (1.54 to 2.09)	2.14 (1.89 to 2.42)	0.79 (0.67 to 0.94)
Concomitant SGLT2i use	1.61 (1.20 to 2.16)	2.65 (2.08 to 3.37)	0.65 (0.46 to 0.92)
No concomitant SGLT2i use	1.61 (1.40 to 1.86)	1.90 (1.68 to 2.15)	0.84 (0.71 to 0.99)
At high cardiovascular risk	2.66 (2.27 to 3.11)	3.28 (2.87 to 3.74)	0.78 (0.65 to 0.93)
Coronary heart disease	3.26 (2.69 to 3.95)	3.38 (2.87 to 3.99)	0.86 (0.69 to 1.08)
Cardiomyopathy	3.70 (2.62 to 5.23)	4.88 (3.56 to 6.69)	0.85 (0.56 to 1.28)
Valve disorders	3.33 (2.53 to 4.39)	3.93 (3.15 to 4.90)	0.76 (0.56 to 1.04)
Heart failure	3.79 (3.00 to 4.78)	4.21 (3.47 to 5.10)	0.78 (0.60 to 1.02)
High-intensive lipid-lowering therapy	1.70 (1.35 to 2.14)	2.28 (1.90 to 2.74)	0.72 (0.55 to 0.94)
**(A) Semaglutide vs sitagliptin in cohort meeting expanded eligibility criteria, Marketscan**			
	1-year-rate, % (95% CI)		
Subgroup	Semaglutide	Sitagliptin	HR (95% CI)
3P-MACE			
Female	*0.79 (0.62 to 1.00)*	*1.00 (0.80 to 1.25)*	*0.76 (0.56 to 1.03)*

Male	*1.08 (0.88 to 1.33)*	*1.27 (1.05 to 1.53)*	*0.76 (0.59 to 0.99)*
Age <65	*0.74 (0.61 to 0.89)*	*0.89 (0.74 to 1.07)*	*0.82 (0.64 to 1.04)*
Age ≥65	*2.21 (1.67 to 2.91)*	*2.69 (2.08 to 3.47)*	*0.83 (0.59 to 1.18)*
Concomitant SGLT2i use	*0.69 (0.45 to 1.06)*	*1.01 (0.70 to 1.46)*	*0.70 (0.41 to 1.18)*
No concomitant SGLT2i use	*0.96 (0.81 to 1.14)*	*1.19 (1.01 to 1.40)*	*0.76 (0.61 to 0.94)*
At high cardiovascular risk	*2.78 (2.25 to 3.42)*	*2.88 (2.34 to 3.55)*	*0.91 (0.70 to 1.19)*
Coronary heart disease	*2.61 (2.00 to 3.41)*	*2.86 (2.20 to 3.71)*	*0.86 (0.61 to 1.22)*
Cardiomyopathy	*3.59 (2.05 to 6.26)*	*3.70 (2.20 to 6.19)*	*0.73 (0.36 to 1.45)*
Valve disorders	*1.99 (1.26 to 3.14)*	*3.83 (2.73 to 5.36)*	*0.50 (0.29 to 0.84)*
Heart failure	*4.26 (2.96 to 6.12)*	*4.46 (3.28 to 6.07)*	*0.73 (0.47 to 1.14)*
High-intensive lipid-lowering therapy	*1.36 (1.04 to 1.80)*	*1.63 (1.25 to 2.13)*	*0.85 (0.60 to 1.21)*
2P-MACE			
Female	0.67 (0.52 to 0.86)	0.86 (0.68 to 1.10)	0.74 (0.54 to 1.03)
Male	0.95 (0.76 to 1.18)	1.00 (0.81 to 1.23)	0.82 (0.62 to 1.09)
Age <65	0.63 (0.52 to 0.77)	0.74 (0.61 to 0.91)	0.83 (0.64 to 1.08)
Age ≥65	1.97 (1.46 to 2.65)	2.12 (1.59 to 2.82)	0.90 (0.61 to 1.31)
Concomitant SGLT2i use	0.63 (0.40 to 0.99)	0.84 (0.57 to 1.25)	0.73 (0.42 to 1.27)
No concomitant SGLT2i use	0.84 (0.70 to 1.01)	0.96 (0.80 to 1.15)	0.80 (0.63 to 1.01)
At high cardiovascular risk	2.51 (2.01 to 3.13)	2.55 (2.04 to 3.18)	0.92 (0.69 to 1.22)
Coronary heart disease	2.41 (1.82 to 3.18)	2.46 (1.86 to 3.25)	0.90 (0.62 to 1.29)
Cardiomyopathy	3.37 (1.88 to 6.02)	3.43 (2.00 to 5.86)	0.71 (0.35 to 1.46)
Valve disorders	1.65 (0.98 to 2.78)	3.00 (2.08 to 4.34)	0.46 (0.25 to 0.83)
Heart failure	3.81 (2.57 to 5.62)	4.06 (2.93 to 5.63)	0.73 (0.45 to 1.16)
High-intensive lipid-lowering therapy	1.24 (0.93 to 1.66)	1.37 (1.03 to 1.82)	0.86 (0.60 to 1.26)
**(A) Semaglutide vs sitagliptin in cohort meeting expanded eligibility criteria, Medicare**			
	1-year-rate, % (95% CI)		
Subgroup	Semaglutide	Sitagliptin	HR (95% CI)
3P-MACE			
Female	*4.23 (3.42 to 5.22)*	*5.30 (4.51 to 6.23)*	*0.65 (0.52 to 0.81)*
Male	*5.26 (4.35 to 6.35)*	*6.76 (5.80 to 7.87)*	*0.77 (0.62 to 0.96)*
Age ≥65	*4.71 (4.10 to 5.41)*	*5.47 (4.88 to 6.12)*	*0.76 (0.65 to 0.89)*
Concomitant SGLT2i use	*3.42 (2.18 to 5.35)*	*5.06 (3.41 to 7.50)*	*0.74 (0.44 to 1.26)*

No concomitant SGLT2i use	*4.75 (4.10 to 5.51)*	*5.54 (4.90 to 6.26)*	*0.78 (0.66 to 0.92)*
At high cardiovascular risk	*6.99 (5.93 to 8.23)*	*8.10 (7.09 to 9.24)*	*0.75 (0.63 to 0.90)*
Coronary heart disease	*8.01 (6.58 to 9.73)*	*8.92 (7.59 to 10.47)*	*0.77 (0.62 to 0.96)*
Cardiomyopathy	*9.42 (6.49 to 13.56)*	*10.62 (7.89 to 14.22)*	*0.82 (0.54 to 1.24)*
Valve disorders	*9.36 (7.25 to 12.05)*	*7.41 (5.91 to 9.28)*	*1.04 (0.77 to 1.41)*
Heart failure	*8.54 (6.85 to 10.62)*	*13.22 (11.24 to 15.50)*	*0.62 (0.49 to 0.79)*
High-intensive lipid-lowering therapy	*5.63 (4.41 to 7.18)*	*7.00 (5.80 to 8.45)*	*0.71 (0.54 to 0.92)*
2P-MACE			
Female	2.84 (2.19 to 3.69)	3.59 (2.94 to 4.38)	0.66 (0.50 to 0.87)
Male	3.37 (2.64 to 4.30)	3.34 (2.70 to 4.13)	0.91 (0.69 to 1.21)
Age ≥65	3.10 (2.61 to 3.69)	3.27 (2.83 to 3.78)	0.83 (0.68 to 1.00)
Concomitant SGLT2i use	2.61 (1.50 to 4.51)	3.22 (1.91 to 5.38)	0.77 (0.40 to 1.48)
No concomitant SGLT2i use	3.07 (2.55 to 3.71)	3.26 (2.78 to 3.83)	0.87 (0.70 to 1.07)
At high cardiovascular risk	4.79 (3.90 to 5.87)	5.41 (4.59 to 6.37)	0.77 (0.62 to 0.97)
Coronary heart disease	5.49 (4.30 to 7.00)	5.78 (4.69 to 7.11)	0.82 (0.62 to 1.07)
Cardiomyopathy	5.08 (3.09 to 8.28)	4.14 (2.64 to 6.47)	1.04 (0.57 to 1.89)
Valve disorders	5.58 (3.98 to 7.80)	4.16 (3.04 to 5.67)	1.12 (0.75 to 1.66)
Heart failure	4.95 (3.71 to 6.59)	7.61 (6.13 to 9.42)	0.62 (0.45 to 0.85)
High-intensive lipid-lowering therapy	4.05 (3.05 to 5.36)	4.60 (3.64 to 5.81)	0.81 (0.59 to 1.12)
**(B) Tirzepatide vs dulaglutide in cohort meeting expanded eligibility criteria, pooled across databases**			
	1-year-rate, % (95% CI)		
Subgroup	Tirzepatide	Dulaglutide	HR (95% CI)
Female	1.47 (1.24 to 1.71)	1.51 (1.23 to 1.78)	1.05 (0.85 to 1.29)
Male	1.56 (1.31 to 1.82)	1.99 (1.68 to 2.31)	0.80 (0.66 to 0.99)
Age <65	0.80 (0.64 to 0.95)	1.04 (0.82 to 1.25)	0.86 (0.67 to 1.10)
Age ≥65	2.50 (2.13 to 2.86)	2.88 (2.44 to 3.32)	0.87 (0.72 to 1.04)
Concomitant SGLT2i use	1.57 (1.21 to 1.93)	2.07 (1.58 to 2.56)	0.90 (0.67 to 1.20)
No concomitant SGLT2i use	1.63 (1.42 to 1.83)	1.67 (1.44 to 1.90)	0.96 (0.81 to 1.13)
At high cardiovascular risk	3.16 (2.70 to 3.62)	3.94 (3.33 to 4.55)	0.88 (0.73 to 1.07)
Coronary heart disease	3.53 (2.91 to 4.16)	4.45 (3.63 to 5.27)	0.89 (0.71 to 1.11)
Cardiomyopathy	4.01 (2.62 to 5.37)	4.56 (2.95 to 6.13)	0.88 (0.57 to 1.37)
Valve disorders	3.46 (2.60 to 4.31)	5.68 (4.34 to 7.01)	0.69 (0.50 to 0.94)

Heart failure	5.01 (3.99 to 6.03)	5.86 (4.62 to 7.09)	0.87 (0.68 to 1.12)
High-intensive lipid-lowering therapy	1.99 (1.61 to 2.37)	2.25 (1.80 to 2.70)	0.91 (0.71 to 1.17)
**(B) Tirzepatide vs dulaglutide in cohort meeting expanded eligibility criteria, Optum**			
	1-year-rate, % (95% CI)		
Subgroup	Tirzepatide	Dulaglutide	HR (95% CI)
Female	1.89 (1.59 to 2.23)	1.86 (1.52 to 2.27)	1.07 (0.85 to 1.35)
Male	2.08 (1.75 to 2.48)	2.46 (2.05 to 2.95)	0.85 (0.68 to 1.07)
Age <65	1.13 (0.90 to 1.41)	1.43 (1.12 to 1.83)	0.88 (0.65 to 1.20)
Age ≥65	2.59 (2.22 to 3.02)	2.86 (2.42 to 3.37)	0.89 (0.73 to 1.08)
Concomitant SGLT2i use	2.09 (1.62 to 2.69)	2.68 (2.05 to 3.50)	0.90 (0.65 to 1.25)
No concomitant SGLT2i use	2.14 (1.87 to 2.45)	1.98 (1.69 to 2.32)	1.04 (0.87 to 1.26)
At high cardiovascular risk	3.58 (3.07 to 4.18)	4.39 (3.72 to 5.18)	0.87 (0.71 to 1.07)
Coronary heart disease	3.67 (3.03 to 4.44)	4.55 (3.70 to 5.59)	0.90 (0.70 to 1.16)
Cardiomyopathy	4.11 (2.81 to 6.00)	5.00 (3.42 to 7.28)	0.80 (0.49 to 1.30)
Valve disorders	3.93 (3.03 to 5.08)	6.41 (4.97 to 8.25)	0.71 (0.51 to 1.00)
Heart failure	5.38 (4.33 to 6.67)	5.91 (4.74 to 7.37)	0.86 (0.66 to 1.13)
High-intensive lipid-lowering therapy	2.28 (1.84 to 2.82)	2.61 (2.09 to 3.26)	0.87 (0.66 to 1.15)
**(B) Tirzepatide vs dulaglutide in cohort meeting expanded eligibility criteria, Marketscan**			
	1-year-rate, % (95% CI)		
Subgroup	Tirzepatide	Dulaglutide	HR (95% CI)
Female	0.77 (0.52 to 1.15)	0.88 (0.58 to 1.34)	0.95 (0.58 to 1.55)
Male	0.68 (0.45 to 1.02)	1.22 (0.89 to 1.68)	0.63 (0.39 to 1.00)
Age <65	0.50 (0.34 to 0.73)	0.69 (0.47 to 0.99)	0.81 (0.52 to 1.27)
Age ≥65	1.87 (1.17 to 2.99)	3.07 (2.12 to 4.42)	0.71 (0.41 to 1.23)
Concomitant SGLT2i use	0.76 (0.45 to 1.28)	1.12 (0.68 to 1.84)	0.89 (0.46 to 1.72)
No concomitant SGLT2i use	0.71 (0.51 to 1.00)	1.13 (0.85 to 1.51)	0.68 (0.47 to 1.00)
At high cardiovascular risk	1.92 (1.29 to 2.85)	2.55 (1.68 to 3.87)	0.97 (0.60 to 1.57)
Coronary heart disease	3.13 (2.00 to 4.90)	4.13 (2.74 to 6.20)	0.84 (0.51 to 1.41)
Cardiomyopathy	3.90 (1.83 to 8.20)	2.74 (1.19 to 6.23)	1.39 (0.48 to 4.00)
Valve disorders	1.78 (0.79 to 4.01)	3.06 (1.73 to 5.38)	0.55 (0.22 to 1.35)
Heart failure	3.42 (1.96 to 5.92)	5.59 (2.98 to 10.38)	0.97 (0.48 to 1.94)

High-intensive lipid-lowering therapy	1.38 (0.90 to 2.10)	1.49 (0.96 to 2.30)	1.10 (0.64 to 1.89)
**(C) Tirzepatide vs semaglutide, pooled across databases**			
	1-year-rate, % (95% CI)		
Subgroup	Tirzepatide	Semaglutide	HR (95% CI)
Female	1.22 (1.07 to 1.37)	1.11 (0.96 to 1.27)	1.13 (0.96 to 1.32)
Male	1.48 (1.29 to 1.66)	1.54 (1.32 to 1.76)	1.06 (0.90 to 1.25)
Age <65	0.87 (0.75 to 0.99)	0.79 (0.67 to 0.92)	1.17 (0.98 to 1.40)
Age ≥65	2.22 (1.95 to 2.48)	2.10 (1.80 to 2.40)	1.12 (0.96 to 1.30)
Concomitant SGLT2i use	1.53 (1.24 to 1.82)	1.37 (1.08 to 1.67)	1.22 (0.95 to 1.56)
No concomitant SGLT2i use	1.29 (1.16 to 1.41)	1.16 (1.02 to 1.29)	1.15 (1.01 to 1.30)
At high cardiovascular risk	3.11 (2.76 to 3.46)	3.09 (2.67 to 3.51)	1.11 (0.96 to 1.27)
Coronary heart disease	3.23 (2.78 to 3.69	3.23 (2.72 to 3.73)	1.05 (0.88 to 1.25)
Cardiomyopathy	3.93 (2.91 to 4.95)	4.69 (3.34 to 6.03)	0.98 (0.71 to 1.36)
Valve disorders	3.01 (2.43 to 3.60)	3.35 (2.60 to 4.09)	1.04 (0.81 to 1.34)
Heart failure	4.54 (3.78 to 5.29)	4.38 (3.59 to 5.16)	1.01 (0.82 to 1.24)
High-intensive lipid-lowering therapy	1.91 (1.62 to 2.20)	1.99 (1.65 to 2.32)	1.04 (0.86 to 1.26)
**(C) Tirzepatide vs semaglutide, Optum**			
	1-year-rate, % (95% CI)		
Subgroup	Tirzepatide	Semaglutide	HR (95% CI)
Female	1.47 (1.29 to 1.67)	1.39 (1.20 to 1.62)	1.09 (0.92 to 1.29)
Male	1.77 (1.54 to 2.02)	1.83 (1.57 to 2.14)	1.06 (0.89 to 1.27)
Age <65	1.09 (0.94 to 1.27)	1.04 (0.88 to 1.24)	1.15 (0.94 to 1.41)
Age ≥65	2.26 (2.00 to 2.55)	2.05 (1.78 to 2.37)	1.09 (0.94 to 1.28)
Concomitant SGLT2i use	1.79 (1.47 to 2.19)	1.75 (1.39 to 2.20)	1.17 (0.89 to 1.53)
No concomitant SGLT2i use	1.56 (1.40 to 1.73)	1.40 (1.24 to 1.59)	1.14 (0.99 to 1.31)
At high cardiovascular risk	3.39 (3.01 to 3.81)	3.24 (2.82 to 3.73)	1.09 (0.94 to 1.27)
Coronary heart disease	3.24 (2.79 to 3.77)	3.57 (3.02 to 4.20)	0.98 (0.81 to 1.18)
Cardiomyopathy	4.31 (3.27 to 5.66)	5.02 (3.73 to 6.73)	0.96 (0.68 to 1.36)
Valve disorders	3.34 (2.73 to 4.08)	3.87 (3.07 to 4.86)	1.02 (0.79 to 1.33)
Heart failure	4.73 (3.99 to 5.59)	4.57 (3.82 to 5.46)	0.98 (0.79 to 1.21)
High-intensive lipid-lowering therapy	2.14 (1.82 to 2.53)	2.31 (1.93 to 2.76)	1.00 (0.81 to 1.23)

(C) Tirzepatide vs semaglutide, Marketscan			
	1-year-rate, % (95% CI)		
Subgroup	Tirzepatide	Semaglutide	HR (95% CI)
Female	0.65 (0.47 to 0.90)	0.44 (0.31 to 0.62)	1.37 (0.90 to 2.08)
Male	0.77 (0.54 to 1.11)	0.85 (0.58 to 1.26)	1.07 (0.70 to 1.64)
Age <65	0.57 (0.42 to 0.76)	0.45 (0.32 to 0.63)	1.24 (0.87 to 1.77)
Age ≥65	1.83 (1.21 to 2.76)	2.68 (1.45 to 4.95)	1.59 (0.87 to 2.88)
Concomitant SGLT2i use	1.01 (0.62 to 1.63)	0.60 (0.33 to 1.07)	1.47 (0.80 to 2.69)
No concomitant SGLT2i use	0.62 (0.46 to 0.82)	0.55 (0.39 to 0.76)	1.18 (0.83 to 1.66)
At high cardiovascular risk	2.00 (1.41 to 2.83)	2.45 (1.59 to 3.76)	1.23 (0.82 to 1.86)
Coronary heart disease	3.23 (2.18 to 4.78)	1.55 (1.01 to 2.39)	1.75 (1.04 to 2.94)
Cardiomyopathy	2.34 (1.13 to 4.81)	3.17 (1.15 to 8.60)	1.14 (0.44 to 2.97)
Valve disorders	1.46 (0.74 to 2.86)	0.85 (0.42 to 1.73)	1.36 (0.54 to 3.46)
Heart failure	3.51 (1.87 to 6.54)	3.19 (1.44 to 7.01)	1.37 (0.70 to 2.70)
High-intensive lipid-lowering therapy	1.25 (0.83 to 1.87)	1.05 (0.64 to 1.72)	1.32 (0.80 to 2.19)

Supplementary Table 31. Power calculations for superiority and non-inferiority studies

(A) Semaglutide vs sitagliptin in cohort meeting trial eligibility criteria				
Superiority analysis: Composite of myocardial infarction, stroke, or all-cause mortality	Optum	Marketscan	Medicare	Pooled
Number of patients				
Reference	13,602	6,554	6,877	27,033
Exposed	13,602	6,554	6,877	27,033
Risk per 1,000 patients	19.08	11.29	25.23	18.76
Expected HR	0.70	0.70	0.70	0.70
Alpha (2-sided)	0.05	0.05	0.05	0.05
Number of events expected	519	148	347	1,014
Power	0.98	0.58	0.91	1.00
(A) Semaglutide vs sitagliptin in cohort meeting expanded eligibility criteria				
Superiority analysis: Composite of myocardial infarction or stroke	Optum	Marketscan	Medicare	Pooled
Number of patients				
Reference	33,973	33,189	12,339	79,501
Exposed	33,973	33,189	12,339	79,501
Risk per 1,000 patients	10.26	5.12	16.09	9.02
Expected HR	0.70	0.70	0.70	0.70
Alpha (2-sided)	0.05	0.05	0.05	0.05
Number of events expected	697	340	397	1,434
Power	1.00	0.91	0.94	1.00
(B) Tirzepatide vs dulaglutide in cohort meeting trial eligibility criteria				
Noninferiority analysis: Composite of myocardial infarction, stroke, or all-cause mortality	Optum	Marketscan	Medicare	Pooled
Number of patients matched				
Reference	9,530	3,297		12,827
Exposed	9,530	3,297		12,827
Risk per 1,000 patients	18.99	10.47		16.80
Assumed HR from RCT	0.8	0.8		0.8

Alpha (2-sided)	0.05	0.05		0.05
Non-inferiority margin	1.05	1.05		1.05
Number of events expected	362	69		431
Power	0.73	0.20		0.81
**(B) Tirzepatide vs dulaglutide in cohort meeting expanded eligibility criteria**				
**Noninferiority analysis: Composite of myocardial infarction, stroke, or all-cause mortality**	**Optum**	**Marketscan**	**Medicare**	**Pooled**
Number of patients matched				
Reference	24,795	14,357		39,152
Exposed	24,795	14,357		39,152
Risk per 1,000 patients	11.52	5.09		9.16
Assumed HR from RCT	0.8	0.8		0.8
Alpha (2-sided)	0.05	0.05		0.05
Non-inferiority margin	1.05	1.05		1.05
Number of events expected	571	146		717
Power	0.90	0.38		0.95
**(C) Tirzepatide vs semaglutide in cohort meeting expanded eligibility criteria**				
**Noninferiority analysis: Composite of myocardial infarction, stroke, or all-cause mortality**	**Optum**	**Marketscan**	**Medicare**	**Pooled**
Number of patients matched				
Reference	60,846	25,345		86,191
Exposed	60,846	25,345		86,191
Risk per 1,000 patients	8.61	3.59		7.13
Assumed HR from RCT	0.9	0.9		0.9
Alpha (2-sided)	0.05	0.05		0.05
Non-inferiority margin	1.10	1.10		1.10
Number of events expected	1,048	182		1,230
Power	0.90	0.27		0.94

Supplementary Table 30. Primary end points before and after propensity score matching

Comparison	Eligibility criteria	Adjustment method	HR (95% CI)
Semaglutide vs sitagliptin	SUSTAIN-6 eligibility	Unadjusted	0.48 (0.45 to 0.51)
		Propensity-score matched	0.68 (0.60 to 0.77)
Semaglutide vs sitagliptin	expanded eligibility	Unadjusted	0.49 (0.46 to 0.52) (myocardial infaction, stroke, all-cause mortality)<br><br>0.58 (0.54 to 0.62) (myocardial infaction, stroke)
		Propensity-score matched	0.71 (0.65 to 0.77) (myocardial infaction, stroke, all-cause mortality)<br><br>0.82 (0.74 to 0.91) (myocardial infaction, stroke)
Tirzepatide vs dulaglutide	SURPASS-CVOT eligibility	Unadjusted	0.57 (0.49 to 0.66)
		Propensity-score matched	0.83 (0.69 to 1.01)
Tirzepatide vs dulaglutide	expanded eligibility	Unadjusted	0.57 (0.51 to 0.64)
		Propensity-score matched	0.87 (0.75 to 1.01)
Tirzepatide vs semaglutide	expanded eligibility	Unadjusted	0.96 (0.88 to 1.06)
		Propensity-score matched	1.06 (0.95 to 1.18)

Supplementary Table 25. 2-year on-treatment causal contrast *post-hoc* analysis

(A) Semaglutide vs sitagliptin in cohort meeting trial eligibility criteria, pooled across databases			
	1–year rate, % (95% CI)		
	Semaglutide	Sitagliptin	HR (95% CI)
Semaglutide vs sitagliptin in cohort meeting trial eligibility criteria, pooled across databases	5.57 (4.66 to 6.47)	7.86 (7.13 to 8.58)	0.65 (0.59 to 0.72)
Semaglutide vs sitagliptin in cohort meeting trial eligibility criteria, Optum	4.85 (4.18 to 5.63)	7.64 (6.86 to 8.49)	0.61 (0.53 to 0.69)
Semaglutide vs sitagliptin in cohort meeting trial eligibility criteria, Marketscan	2.95 (2.25 to 3.85)	5.58 (4.38 to 7.09)	0.69 (0.52 to 0.93)
Semaglutide vs sitagliptin in cohort meeting trial eligibility criteria, Medicare	10.24 (7.04 to 14.75)	10.73 (8.64 to 13.28)	0.75 (0.61 to 0.92)
Semaglutide vs sitagliptin in cohort meeting expanded eligibility criteria, pooled across databases, 3P-MACE	*3.71 (3.40 to 4.01)*	*5.37 (4.96 to 5.78)*	*0.71 (0.66 to 0.77)*
Semaglutide vs sitagliptin in cohort meeting expanded eligibility criteria, pooled across databases, 2P-MACE	2.46 (2.22 to 2.70)	3.19 (2.89 to 3.48)	0.81 (0.73 to 0.89)
Semaglutide vs sitagliptin in cohort meeting expanded eligibility criteria, Optum, 3P-MACE	*4.61 (4.14 to 5.13)*	*6.63 (6.07 to 7.24)*	*0.68 (0.61 to 0.76)*
2- Semaglutide vs sitagliptin in cohort meeting expanded eligibility criteria, Optum, 2P-MACE	2.94 (2.57 to 3.36)	3.72 (3.30 to 4.19)	0.80 (0.70 to 0.91)
Semaglutide vs sitagliptin in cohort meeting expanded eligibility criteria, Marketscan, 3P-MACE	*1.50 (1.27 to 1.76)*	*2.20 (1.88 to 2.58)*	*0.75 (0.62 to 0.89)*
Semaglutide vs sitagliptin in cohort meeting expanded eligibility criteria, Marketscan, 2P-MACE	1.29 (1.08 to 1.54)	1.76 (1.47 to 2.10)	0.79 (0.65 to 0.96)
Semaglutide vs sitagliptin in cohort meeting expanded eligibility criteria, Medicare, 3P-MACE	*6.97 (5.84 to 8.31)*	*10.13 (8.50 to 12.05)*	*0.76 (0.65 to 0.88)*
Semaglutide vs sitagliptin in cohort meeting expanded eligibility criteria, Medicare, 2P-MACE	4.24 (3.42 to 5.24)	5.54 (4.48 to 6.83)	0.84 (0.70 to 1.02)
Tirzepatide vs dulaglutide in cohort meeting trial eligibility criteria, pooled across databases	4.65 (3.80 to 5.50)	7.29 (5.50 to 9.05)	0.79 (0.66 to 0.94)
Tirzepatide vs dulaglutide in cohort meeting trial eligibility criteria, Optum	5.56 (4.55 to 6.77)	8.23 (6.35 to 10.64)	0.76 (0.63 to 0.92)
Tirzepatide vs dulaglutide in cohort meeting trial eligibility criteria, Marketscan	2.02 (1.38 to 2.95)	4.52 (2.26 to 8.94)	1.01 (0.64 to 1.61)
Tirzepatide vs dulaglutide in cohort meeting expanded eligibility criteria, pooled across databases	2.28 (1.96 to 2.60)	3.44 (2.77 to 4.11)	0.84 (0.73 to 0.96)
Tirzepatide vs dulaglutide in cohort meeting expanded eligibility criteria, Optum	3.05 (2.61 to 3.56)	4.69 (3.78 to 5.82)	0.82 (0.70 to 0.96)
Tirzepatide vs dulaglutide in cohort meeting expanded eligibility criteria, Marketscan	0.96 (0.70 to 1.30)	1.26 (0.91 to 1.74)	0.90 (0.66 to 1.24)
Tirzepatide vs semaglutide, pooled across databases	2.13 (1.88 to 2.38)	2.33 (1.99 to 2.67)	1.04 (0.94 to 1.16)
Tirzepatide vs semaglutide, Optum	2.68 (2.36 to 3.04)	2.78 (2.42 to 3.20)	1.03 (0.92 to 1.16)
Tirzepatide vs semaglutide, Marketscan	0.83 (0.61 to 1.11)	1.25 (0.73 to 2.15)	1.12 (0.84 to 1.50)
