# Supplementary material for: Cardiovascular outcomes of semaglutide and tirzepatide for patients with type 2 diabetes in clinical practice
Source: Nat Med. 2025 Nov 9;32(1):342–52. doi: 10.1038/s41591-025-04102-x (PMC12823426; doi:10.1038/s41591-025-04102-x)
Supplement: Supplementary file 2 — Reporting Summary [file 41591_2025_4102_MOESM2_ESM.pdf]

Corresponding author(s): Nils Krüger, MD
Last updated by author(s): Nov 3, 2025

Reporting Summary

Nature Portfolio wishes to improve the reproducibility of the work that we publish. This form provides structure for consistency and transparency in reporting. For further information on Nature Portfolio policies, see our Editorial Policies and the Editorial Policy Checklist.

Statistics

For all statistical analyses, confirm that the following items are present in the figure legend, table legend, main text, or Methods section.

n/a	Confirmed	
☐	☒	The exact sample size (n) for each experimental group/condition, given as a discrete number and unit of measurement
☐	☒	A statement on whether measurements were taken from distinct samples or whether the same sample was measured repeatedly
☒	☐	The statistical test(s) used AND whether they are one- or two-sided<br>*Only common tests should be described solely by name; describe more complex techniques in the Methods section.*
☐	☒	A description of all covariates tested
☒	☐	A description of any assumptions or corrections, such as tests of normality and adjustment for multiple comparisons
☐	☒	A full description of the statistical parameters including central tendency (e.g. means) or other basic estimates (e.g. regression coefficient) AND variation (e.g. standard deviation) or associated estimates of uncertainty (e.g. confidence intervals)
☐	☒	For null hypothesis testing, the test statistic (e.g. F, t, r) with confidence intervals, effect sizes, degrees of freedom and P value noted<br>*Give P values as exact values whenever suitable.*
☒	☐	For Bayesian analysis, information on the choice of priors and Markov chain Monte Carlo settings
☒	☐	For hierarchical and complex designs, identification of the appropriate level for tests and full reporting of outcomes
☒	☐	Estimates of effect sizes (e.g. Cohen's d, Pearson's r), indicating how they were calculated

Our web collection on statistics for biologists contains articles on many of the points above.

Software and code

Policy information about availability of computer code

Data collection	Routinely collected healthcare claims data was licensed via data use agreements for Medicare, Optum Clinformatics, and Merative MarketScan data
Data analysis	Aetion Evidence Platform, Python, R

For manuscripts utilizing custom algorithms or software that are central to the research but not yet described in published literature, software must be made available to editors and reviewers. We strongly encourage code deposition in a community repository (e.g. GitHub). See the Nature Portfolio guidelines for submitting code & software for further information.

Data

Policy information about availability of data

All manuscripts must include a data availability statement. This statement should provide the following information, where applicable:

- Accession codes, unique identifiers, or web links for publicly available datasets
- A description of any restrictions on data availability
- For clinical datasets or third party data, please ensure that the statement adheres to our policy

Data use agreements and licensing agreements do not allow sharing of patient-level claims data with third parties. However, data can be requested at the vendors directly (Optum Clinformatics, connected@optum.com; Medicare, resdac@umn.edu; Merative MarketScan, marketscan.support@merative.com).

Research involving human participants, their data, or biological material

Policy information about studies with human participants or human data. See also policy information about sex, gender (identity/presentation), and sexual orientation and race, ethnicity and racism.

Reporting on sex and gender	We report descriptive characteristics of our analytic cohort, including self-reported gender. Our table 1 describing baseline characteristics describes the count and percent of patients who are self-reported female or self-reported male.
Reporting on race, ethnicity, or other socially relevant groupings	We report descriptive characteristics such as self-reported race in our table 1. The self-reported race is included in our propensity score model and is balanced between exposure groups in the analytic cohort that produced the primary results. The self reported race categories include:<br>White; n (%)<br>Black; n (%)<br>Unknown / Missing; n (%)<br>Others; n (%)
Population characteristics	Patients with obesity and type 2 diabetes at elevated cardiovascular risk
Recruitment	Patients were identified from routinely collected healthcare claims data in a secondary data analysis. No patients were directly contacted or recruited.
Ethics oversight	The study was approved by the Mass General Brigham Institutional Review Board

Note that full information on the approval of the study protocol must also be provided in the manuscript.

Field-specific reporting

Please select the one below that is the best fit for your research. If you are not sure, read the appropriate sections before making your selection.

☒ Life sciences ☐ Behavioural & social sciences ☐ Ecological, evolutionary & environmental sciences

For a reference copy of the document with all sections, see nature.com/documents/nr-reporting-summary-flat.pdf

Life sciences study design

All studies must disclose on these points even when the disclosure is negative.

Sample size	Sample size and power calculations were included in the protocol that was pre-registered prior to conducting analyses. The assumptions of the power calculations are detailed in the protocols.
Data exclusions	Exclusions for the benchmarking studies emulated the eligibility criteria for the SUSTAIN-6 and SUPRASS-CVOT trials. Exclusions were relaxed for the expanded study populations. These criteria were pre-specified in the pre-registered protocols.
Replication	The benchmarking studies were attempts to replicate the SUSTAIN-6 and SURPASS-CVOT trials using non-randomized healthcare data. The trial results were replicated with the exception of the all-cause mortality outcome for the SUSTAIN-6 benchmarking emulation. This may have been related to potential residual confounding for this secondary end point that could reflect preferential prescribing in patients for whom clinicians anticipated limited life expectancy. Insights from this lack of replication informed the study in expanded populations and the head-to-head comparison.
Randomization	This was a non-randomized study that made secondary use of routinely collected healthcare claims data.
Blinding	There was no blinding in this non-randomized study that made secondary use of routinely collected healthcare claims data.

Reporting for specific materials, systems and methods

We require information from authors about some types of materials, experimental systems and methods used in many studies. Here, indicate whether each material, system or method listed is relevant to your study. If you are not sure if a list item applies to your research, read the appropriate section before selecting a response.

Materials & experimental systems

n/a	Involved in the study
☒	☐ Antibodies
☒	☐ Eukaryotic cell lines
☒	☐ Palaeontology and archaeology
☒	☐ Animals and other organisms
☐	☒ Clinical data
☒	☐ Dual use research of concern
☒	☐ Plants

Methods

n/a	Involved in the study
☒	☐ ChIP-seq
☒	☐ Flow cytometry
☒	☐ MRI-based neuroimaging

Clinical data

Policy information about clinical studies

All manuscripts should comply with the ICMJE guidelines for publication of clinical research and a completed CONSORT checklist must be included with all submissions.

Clinical trial registration	NCT06659744, NCT07088718, NCT07096063
Study protocol	See ClinicalTrials.gov
Data collection	Three secondary healthcare claims databases, including data from Medicare Parts A, B, and D (2018 through 2020), Optum Clinformatics Data Mart (2018 through Feburary 2025), and Merative MarketScan (2018 through 2023).
Outcomes	The primary and secondary outcome measures were pre-specified in protocols that were pre-registered on clinicaltrials.gov. The end point algorithms were previously validated and showed a sensitivity of over 99% for mortality in the National Death Index, a positive predictive value of 94% for myocardial infarction, and 95% for stroke, and 98% for heart failure hospitalization.

Plants

Seed stocks	No seed stocks were involved in this study.
Novel plant genotypes	No novel plant genotypes were involved in this study.
Authentication	No authentication of seed stocks or novel plant genotypes were relevant for this study.
